# The palladium-catalysed modular annulative synthesis of cyclic sulfonimidamides

**DOI:** 10.1039/d6sc02864k

**Published:** 2026-05-19

**Authors:** Kangtao Zhu, Agamemnon E. Crumpton, Michael C. Willis

**Affiliations:** a Department of Chemistry, University of Oxford, Chemistry Research Laboratory Oxford OX1 3TA UK michael.willis@chem.ox.ac.uk

## Abstract

Acyclic sulfonimidamides have gained increasing attention in medicinal chemistry programs; however, examples of their cyclic counterparts are much rarer, reflecting the dearth of convenient synthetic methods to access these unusual heterocycles. Herein, we report a modular one-pot Pd-catalysed method for the synthesis of cyclic sulfonimidamides from the combination of *ortho*-halo benzaldehydes and acyclic sulfonimidamides. The protocol includes a broad scope for both reaction partners and tolerates a diverse range of functional groups, placing substituents at every position of the ring system. The chemistry can be extended to achieve access to alternative cyclic S(iv) and S(iv) functional groups, including sulfinamides, sulfonimidoyl fluorides, and sulfonimidate esters. Preliminary findings on the functionalisation of the cyclic sulfonimidamide products are included. We also demonstrate that enantiomerically enriched cylic sulfonimidamdes can be prepared when starting from an enantiopure building block. Additionally, we report the first synthesis of cyclic sulfondiimidamides *via* a related Chan–Lam coupling.

## Abstract 2

A modular one-pot Pd-catalysed method for the synthesis of structurally unique benzo-fused cyclic sulfonimidamides from the combination of *ortho*-halo benzaldehydes and acyclic sulfonimidamides is reported.

## Graphical abstract

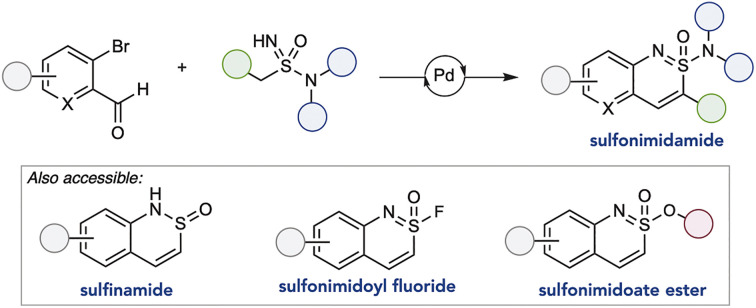

The favourable structural and physicochemical properties of sulfonamides, such as their polarity and hydrogen-bonding capacity, underpin their widespread use in medicinal chemistry programmes.^1–3^ Cyclic sulfonamides are also common; the conformational rigidity and well-defined spatial orientation of substituents make them attractive scaffolds. Notably, 2,1-benzothiazines – a prominent class of cyclic sulfonamides – have attracted attention, with applications as focal adhesion kinase (FAK) inhibitors,^4^ Dengue virus NS5 RdRp inhibitors,^5^ and interleukin-8 receptor antagonists (Fig. 1A and B).^6^ Other cyclic sulfonamide architectures have also been explored as medicinal agents.^7–9^ The mono aza derivatives of sulfonamides, sulfonimidamides,^10,11^ are gaining traction as potent bioactive molecules.^12–14^ They are now commonplace in the patent literature,^15–17^ with several examples advancing to clinical trials.^18^ Despite this progress, examples of cyclic sulfonimidamides,^19–25^ particularly benzo-fused variants, remain rare.^26–28^

**Fig. 1:**
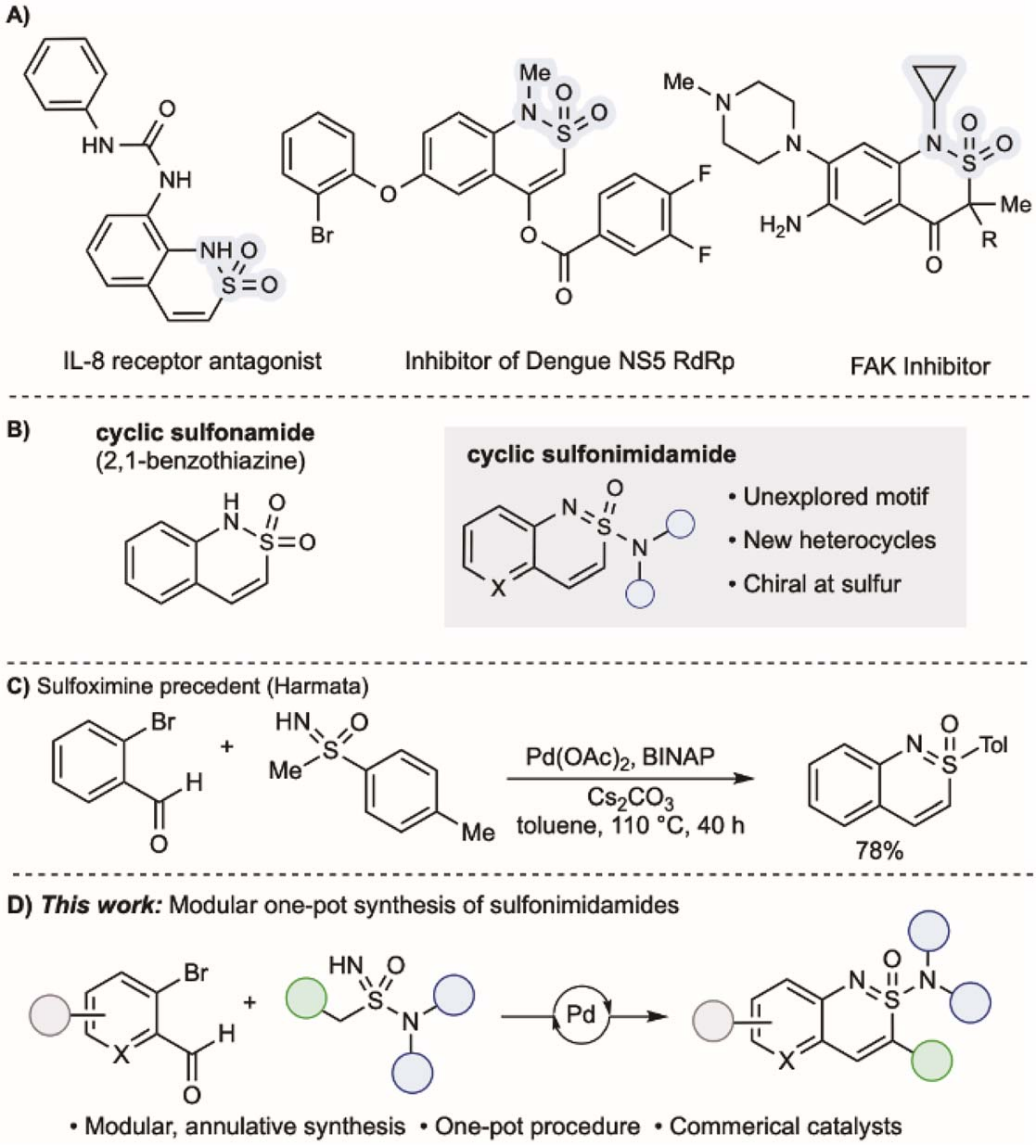
(A) Examples of bioactive cyclic sulfonamides. (B) 2,1-Benzothiazines and cyclic sulfonimidamides. (C) Cyclic sulfoximine precedent from Harmata. (D) This work: the modular Pd-catalysed synthesis of sulfonimidamides.

To address this shortcoming, and recognising the potential value of benzo-fused cyclic sulfonimidamides could make in exploring new chemical space, we conceived of a concise synthesis based on the union of acyclic NH-sulfonimidamides with *ortho*-halo-substituted benzaldehydes using palladium catalysis (Fig. 1D). Importantly, the required acyclic sulfonimidamide substrates would be available in a single step (see Scheme 1A). We now report the successful execution of this plan, and describe an efficient route to cyclic sulfonimidamides; the reactions are broad in scope, tolerate a variety of functional groups and employ commercial catalyst components. Modifications to the protocol allows access to related cyclic sulfinamides, sulfonimidoyl chlorides and fluorides, and sulfonimidoate esters.

**Scheme 1:**
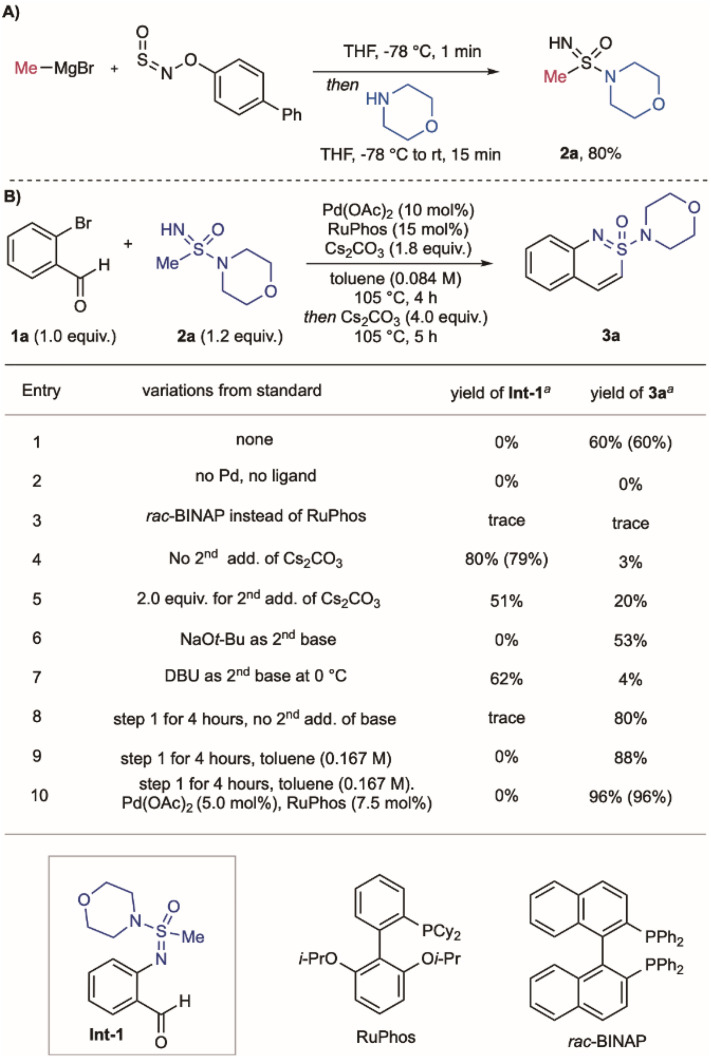
(A) Synthesis of sulfonimidamide 2a using the BiPhONSO reagent. (B). Conditions for the synthesis of cyclic sulfonimidamide 3a: ^*a*^ Yields determined by quantitative ^1^H NMR spectroscopy of the crude reaction mixture using methyl 3,5-dinitrobenzoate as the internal standard. Isolated yields are in parentheses.

Our reaction design requires palladium-catalysed *N*-arylation of the acyclic sulfonimidamides,^29,30^ followed by an intramolecular aldol-type condensation. An important precedent was provided by Harmata,^31–34^ who had achieved related reactions with sulfoximines (Fig. 1C).^35^ The use of sulfonimidamides in these processes is unknown, and has the challenge of using more complex substrates and the reduced acidity of the relevant alkyl protons. An important design consideration was that the acyclic sulfonimidamide substrates should be readily available, and that both the carbon and amidyl substituents should be easily varied. To achieve this modularity, we assembled the required cyclisation substrates using the commercially available BiPhONSO reagent^36^ in combination with organometallic reagents and amines;^37^ an example synthesis, shown for sulfonimidamide 2a, is shown in Scheme 1A. To investigate the proposed coupling/cyclisation, we examined the combination of 2-bromobenzaldehyde 1a with sulfonimidamide 2a, leading to cyclic sulfonimidamide 3a. The process is a one-pot, two-step sequence, with the first C–N coupling involving Pd(OAc)_2_ and RuPhos as catalysts, Cs_2_CO_3_ as base, in toluene at 105 °C for 4 h; the cyclisation requires additional Cs_2_CO_3_ and stirring for 5 h, and delivers the cyclic sulfonimidamide 3a in 60% yield (Scheme 1B, entry 1). Control experiments established that palladium and ligand are essential (entry 2). (Rac)-BINAP, which was used for the sulfoximine chemistry,^31^ was inefficient for C–N bond formation, with starting materials being recovered (entry 3). The presence of a second base was essential for the cyclization step, with 4.0 equivalents proving optimal (entries 4 and 5); alternative bases led to diminished yields (entries 6 and 7). For reactions with inefficient cyclisation steps, the non-cyclic *N*-arylated intermediate Int-1 could be isolated. Notably, doubling the reaction concentration significantly improved both the *N*-arylation and cyclization steps, resulting in an 88% yield of 3a (entry 8). Finally, reducing the catalyst loading by half further enhanced the overall efficiency, delivering 3a in 96% yield (entry 9), and provided the optimised reaction conditions.

With the optimised reaction conditions in hand, we then investigated the scope with respect to both reaction components (Scheme 2). Variation of the halo-benzaldehydes was explored first, with acyclic sulfonimidamide 2a remaining constant. Although the majority of substrates maintained an *ortho*-Br substituent, we established that the chloro-variant could be used with minimal loss of efficiency (3a).^38,39^ Both electron-donating and electron-withdrawing substituents *para* to the bromo-group were well tolerated in this two-step transformation, including methoxy (3b), chloro (3c), nitro (3d) and fluoro (3e) groups. The structure of sulfonimidamide 3c was confirmed by X-ray analysis, showing the near-planarity of the fused ring system.^40^ Fluorine substituents could be placed at multiple positions around the benzo-ring (3e, 3f, 3g). A substrate featuring a methyl group positioned next to the reacting Br-group was successfully used, albeit with reduced efficiency (3h). Dioxolane substitution could also be included (3i). Heteroaromatic ortho-halo aldehydes were also competent substrates, providing interesting pyridine- and thiophene-fused products (3j–3l). An *ortho*-bromobenzophenone-derived substrate could be employed, providing the corresponding 4-phenyl-substituted sulfonimidamide (3m) in a good yield. Using an *ortho*-bromobenzonitrile substrate in combination of sulfonimidamide 2a was unsuccessful (3n).

**Scheme 2:**
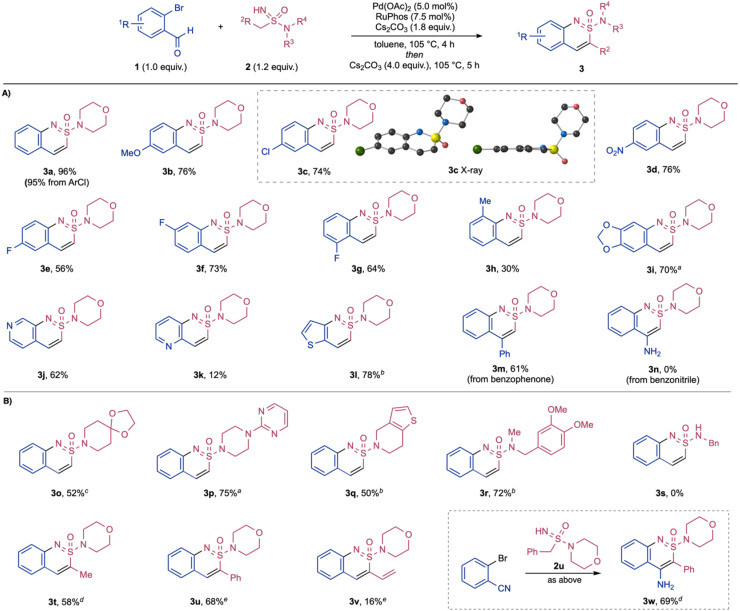
Synthesis of cyclic sulfonimidamides 3. (A) Variation of the halo-benzaldehyde fragment. (B) Variation of the acyclic sulfonimidamide component. Reaction conditions: *o*-halobenaldehyde (1, 1.0 equiv.), acyclic sulfonimidamide (2, 1.2 equiv.), Pd(OAc)_2_ (5.0 mol%), RuPhos (7.5 mol%), Cs_2_CO_3_ (1.8 equiv.), toluene, 105 °C, 4 h; then Cs_2_CO_3_ (4.0 equiv.), 105 °C, 5 h. ^*a*^ 24 h for second step. ^*b*^ 18 h for second step. ^*c*^ 72 h for second step. ^*d*^ 135 °C for second step. ^*e*^ 18 h for first step and without the second addition of Cs_2_CO_3_.

Variation of the acyclic sulfonimidamide was then evaluated, starting the with the amidyl group. A range of both cyclic and acyclic amines could be incorporated (3o–3r). In particular, the piperazine group present in the antiparkinsonian medication Piribedil, and the piperidine fragment from Clopidogrel (an antiplatelet medicine) were successfully incorporated (3p and 3q). Acyclic sulfonimidamides including a primary amidyl group could not be used (3s). The carbon substituent of the sulfonimidamide substrates was then varied, with morpholine held constant as the amine component. Higher reaction temperatures were required in the annulation of ethyl-substituted substrate (3t); in contrast, this was not needed for the phenyl and vinyl substituted examples (3u, 3v). Of note, although the reaction of 2-bromobenzonitrile with sulfonimidamide 2a was unsuccessful, the nitrile substrate could be usefully combined with phenyl-substituted sulfonimidamide 2u, affording a heterocyclic sulfonimidamide bearing an additional 4-amino functionality (3w). The success in using sulfonimidamide 2u is attributed to the increased acidity of the of the methylene group, relative to the methyl group in 2a.

As noted above, acyclic sulfonimidamides derived from primary amines were unsuccessful substrates, with the initial Pd-catalysed C–N bond formation the challenge. To address this issue, we conceived an alternative strategy that involves the final step addition of an amine (or alternative nucleophile) to an activated cyclic substrate that includes a suitable leaving group, *i.e.*, a cyclic sulfonimidoyl chloride (8 → 9, Scheme 3B). To access sulfonimidoyl chloride 8 we targeted cyclic sulfinamide 6 as the precursor, and this was available from the union of *o*-bromobenzaldehyde and methyl *t*-butylsulfoxime 4 using our developed reaction conditions, followed by removal of the *t*-butyl group using TFA (Scheme 3A).^41,42^ Sulfinamide 6 was readily converted into the corresponding sulfonamide (7) using *m*-CPBA, or into sulfonimidoyl chloride 8 by treatment with TCCA.^43,44^ The sulfonimidoyl chloride was challenging to isolate, but could be simply combined with primary amines to deliver the targeted cyclic sulfonimidamides. Using this approach, various primary amines, including primary alkyl (9a, 9b), secondary alkyl (9c), and tertiary alkyl (9d) were successfully introduced. Additionally, aniline and ammonia proved to be competent nucleophiles (9e, 9f). Introduction of an enantiomerically enriched α-methylbenylamine led to the formation of two separable diastereomeric cyclic sulfonimidamides (9c, 9c′). The stereochemistry at the S(vi) centre was confirmed X-ray diffraction, and subsequent deprotection of the α-methylbenzyl group with triflic acid furnished the corresponding enantiomerically pure primary cyclic sulfonimidamide 9f (Scheme 3C). Beyond primary amines, the use of diverse nucleophiles post-chlorination was possible; for example, a secondary amine was incorporated in good yield (3a), and treatment with silver fluoride provided access to the cyclized sulfonimidoyl fluoride (10). Similarly, nucleophilic substitution with sodium phenoxide delivered the corresponding sulfonimidate ester (11). Both the sulfonimidoyl fluoride and the sulfonimidate ester are isolable, stable intermediates for downstream transformations, such as SuFEx chemistry.^45–48^

**Scheme 3:**
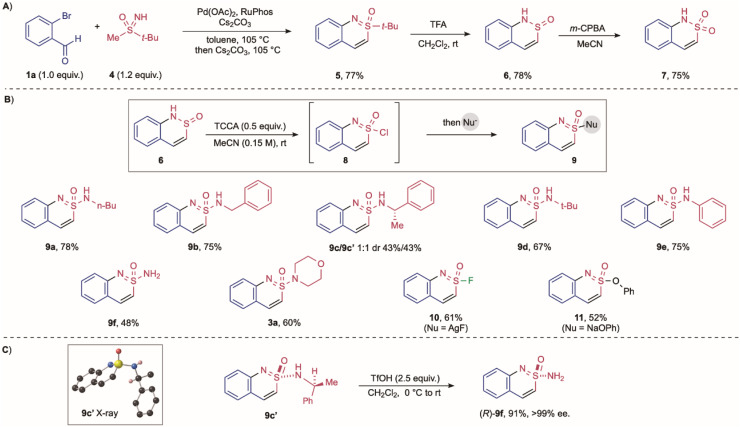
Synthesis of cyclic sulfonimidamides 9 derived from primary acyclic sulfonimidamides. (A) Preparation of cyclic sulfinamide 6. (B) Preparation of cyclic sulfonimidamides 9 from sulfonimidoyl chloride 8; preparation of sulfonimidoyl fluoride 10 and sulfonimidate ester 11. (C) X-ray of sulfonimidamide 9c′ and the preparation of (*R*)-9f.

We next turned our attention to the preparation of cyclic sulfondiimidamides, which are the double aza analogues of sulfonamides.^49^ In these structures, variation of the two *N*-substituents offers the potential for precise tuning of their acid–base properties as well as the hydrogen-bonding donor and acceptor characteristics.^50,51^ Initially, attempts at simply switching the acyclic sulfonimidamide substrate for an acyclic sulfondiimidamide, and using the developed palladium-catalysed protocol described above, proved unsuccessful. The issue was decomposition of the sulfondiimidamide substrate at the elevated reaction temperatures needed. Encouragingly, a Chan–Lam coupling approach, using boronic acids in place of aryl halides, proved to be a viable alternative.^52,53^ Reaction between *o*-formylphenyl boronic acid and sulfondiimidamide 12, using conditions previously identified for the *N*-arylation of sulfondiimidamides^50^ (Cu(CH_3_CN)_4_PF_6_ and an oxygen atmosphere), provided *N*-aryl sulfondiimidamide 13 in 75% yield (Scheme 4).^54^ Dehydrative-cyclisation was achieved by treating 13 with NaO*t*-Bu in toluene, to deliver benzo-fused cyclic sulfondiimidamide 14 in 73% yield. This Chan–Lam protocol was also applicable to the preparation of cyclic sulfonimidamides, albeit with lower overall efficiency, compared to the Pd-based route (see SI for details).

**Scheme 4:**
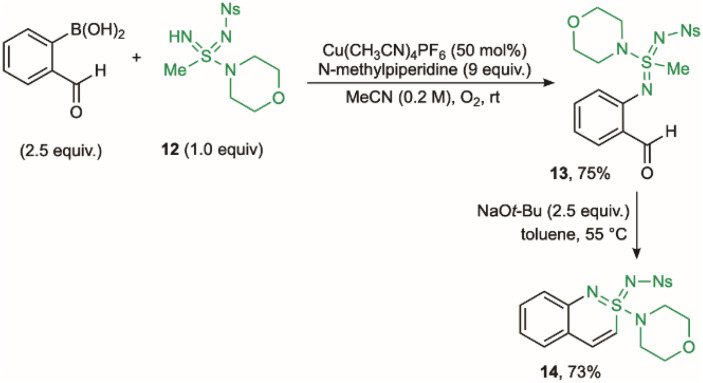
Synthesis of benzo-fused cyclic sulfondiimidamide 14.

With a range of benzo-fused cyclic sulfonimidamides available, we conducted preliminary experiments on the functionalization of these new scaffolds. Treatment of cyclic sulfonimidamide 3a with NBS induced bromination, providing C-6 bromo-derivative (15) in 88% yield (Scheme 5). If the C-6 position is blocked, as in methoxy derivative 3b, the same reaction conditions deliver a mixture of brominated products (16a–16d), including those functionalised in the sulfonimidamide ring. Efficient functionalisation at C-3 could be achieved using a lithiation/electrophile trapping sequence.^55^ For example, treatment of sulfonimidamide 3a with *n*-butyllithium, followed by trapping with TIPS-Cl, delivered C-3 silyl derivative 17 in 81% yield.

**Scheme 5:**
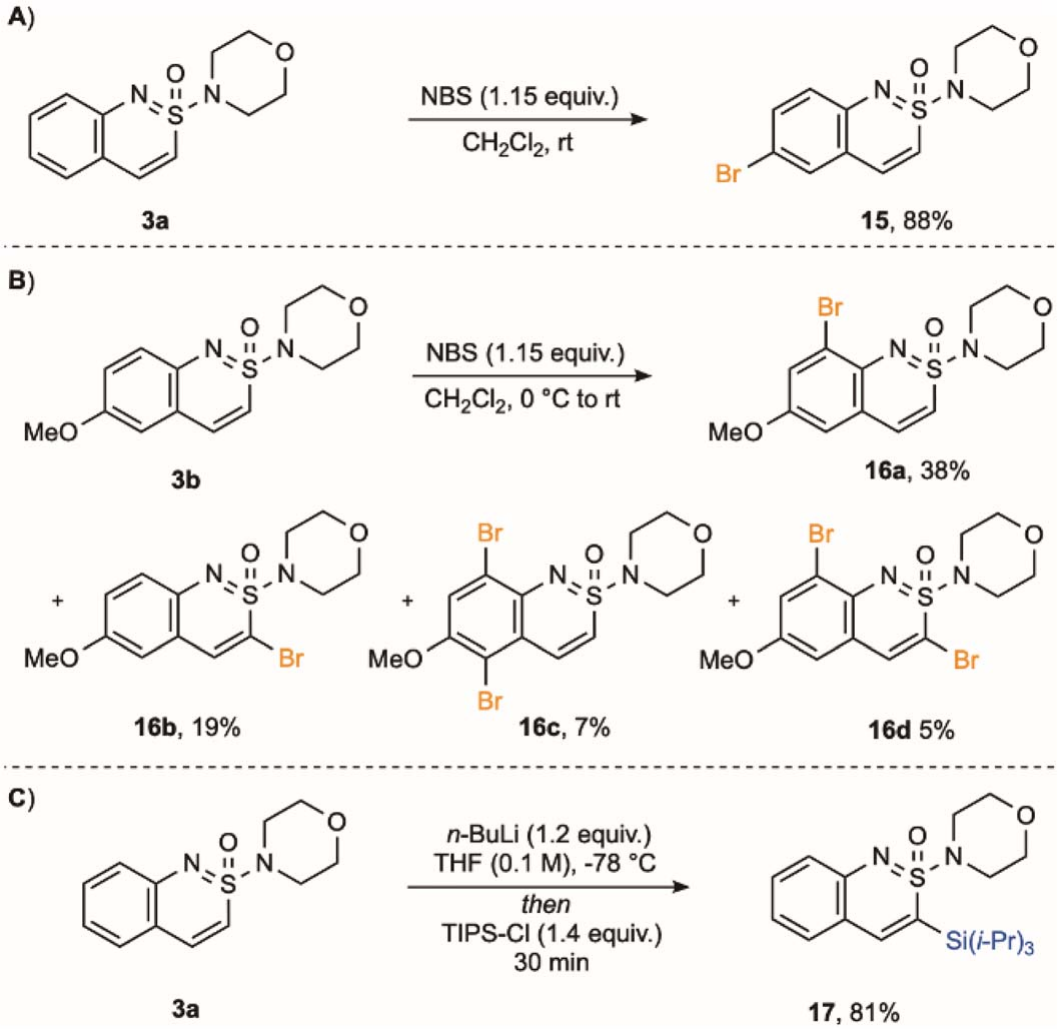
(A) Bromination of sulfonimidamide 3a. (B) Bromination of sulfonimidamide 3b. (C) Lithiation/silylation of sulfonimidamide 3a.

In conclusion, we have developed an efficient one-pot protocol for the preparation of benzo-fused cyclic sulfonimidamides. The method combines acyclic NH-sulfonimidamides with *ortho*-halobenzaldehydes, using palladium catalysis under basic conditions. Good variation of both reaction partners is possible, and substituents can be placed at all positions of fused-ring products. A complementary route uses a cyclic sulfonimidoyl chloride intermediate, and allows access to the corresponding sulfonimidamides, sulfonimidoyl fluorides, and sulfonimidate esters. Finally, a benzo-fused sulfondiimidamide was also prepared using a related route, but with a copper-catalysed Chan-Lam *N*-arylation as the key step. Collectively, these cyclic S(vi)-derivatives possess potential tunable hydrogen-bonding motifs, acid/base properties, and heterocyclic topologies, making them promising candidates for further exploration in medicinal chemistry and chemical biology.

## Author contributions

K. Z. and M. C. W. designed the study, K. Z. conducted all the experiments. A. E. C. performed the X-ray determination for compound 9c′. M. C. W. directed the project. All authors wrote the manuscript.

## Conflicts of interest

The authors declare no conflicts of interest.

## Supplementary Material

SC-OLF-D6SC02864K-s001

SC-OLF-D6SC02864K-s002

## Data Availability

CCDC 2536631 and 2536670 contain the supplementary crystallographic data for this paper.^56*a*,*b*^ The data supporting this article have been included as part of the supplementary information (SI). Supplementary information is available. See DOI: https://doi.org/10.1039/d6sc02864k.
