# Supplementary material for: The palladium-catalysed modular annulative synthesis of cyclic sulfonimidamides
Source: Chem Sci. 2026 May 19;17(25):12551–7. doi: 10.1039/d6sc02864k (PMC13184836; doi:10.1039/d6sc02864k)
Supplement: SC-017-D6SC02864K-s001 [file SC-017-D6SC02864K-s001.pdf]

Supplementary Information (SI) for Chemical Science.
This journal is © The Royal Society of Chemistry 2026

Table of Contents

1. General consideration 2
2. Optimisations 4
  2.1 Ligand screening and equivalence modification 4
  2.2 Second base screening in cyclisation 5
  2.3 Further optimization on *N*-arylation 6
  2.4 *N*-arylation and cyclisation in one pot 6
  2.5 Unsuccessful substrates 7
3. Experimental procedures and characterisations 8
  3.1 Preparation of BiPhONSO 8
  3.2 Preparation of Sulfonimidamide 10
  3.3 Preparation of cyclised sulfonimidamide (secondary amine) 17
  3.4 Preparation of N-aryl sulfonimidamide 34
  3.5 Preparation of cyclic sulfonimidamides (primary amine) 36
  3.6 Derivatisation of sulfinamide 46
  3.7 Chan-Lam couping and cyclisation of sulfondiimidamide 48
  3.8 Derivatisation of sulfonimidamides 51
4. Reference 56
5. NMR spectra 57
6. HPLC-Data 116

1. General consideration

Reactions were performed under inert nitrogen atmosphere with anhydrous solvent unless otherwise stated. All glassware was oven dried over 100 ℃ and cooled to room temperature under positive pressure of nitrogen. Reactions were monitored by TLC using aluminium backed silica plates. Plates were visualized under ultraviolet light (254 nm) and/or staining with $KMnO_4$/Ninhydrin. The cooling of reaction mixture to -78 ℃ was achieved using a dry ice-acetone bath. Cooling of reaction mixtures to 0 °C was achieved using an ice-water bath.

Unless otherwise stated, all chemicals were purchased from commercial sources (Sigma Aldrich, Fluorochem, Fisher Scientific, Alfa-Aesar or Apollo Scientific) and were used without further purification. Anhydrous solvents were purified by filtration through dried alumina columns using the University of Oxford internal solvent drying system (Innovative Technology Inc. PS-400-7) and sparged with nitrogen before use. Toluene was obtained from the solvent drying system and stored under nitrogen with activated 4 Å molecular sieves. The solvent was degassed with nitrogen for 30 min before use. All inert gases were sourced from the University of Oxford internal supplies and dried through $CaCl_2$ drying columns. 'Petrol ether' refers to the fraction of petroleum ether which boils in the range 40-60 °C. 'Brine' refers to a saturated aqueous solution of sodium chloride. Flash chromatorgraphy was carried out using Geduran Si 60, 40–63-micron silica gel. 'Petrol' refers to the fraction of petroleum ether which boils in the range 40-60 °C.

^{1}H NMR spectra were obtained on a Bruker AVIII400 (400MHz) spectrometer using the residual solvent as an internal standard. ^{13}C-NMR spectra were obtained on a Bruker AVIII 400 (101 MHz) using the residual solvent as an internal standard. ^{19}F-NMR spectra were obtained on a Bruker AVIII400 (377 MHz) spectrometer. Chemical shifts (δ) were reported in parts per million (ppm) with the multiplicities of the spectra reported as following: singlet (s); broad singlet (br.s); doublet (d); triplet (t); quartet (q); pentet (pent); sextet (sext); heptet (hept); multiplet (m); apparent (app.) Coupling constants (J) were given in Hertz (Hz).

High resolution mass spectrometry measurements were recorded on a Bruker Daltronics MicroTOF (ESI) spectrometer by the internal service at Chemistry Research Laboratory, University of Oxford. Samples for mass spectra were prepared as 1 mg/mL solution in MeOH (LRMS, HRMS-ESI).

Infrared spectra were recorded on a Bruker Tensor 27 Fourier Transform spectrometer with an internal range 600-4000 cm^{-1} and all absorption maximum (ν_{max}) are given in wavenumbers (cm^{-1}).

Melting points were determined using a Stuart Scientific Melting Point Apparatus SMP1 and are reported uncorrected.

Optical rotations were measured on a Schmidt Haensch UniPol L2000 polarimeter at 589 nm, 25 °C. $[\boldsymbol{\alpha}]_{\mathbf{D}}^{\mathbf{25}}$ is expressed in deg cm^3 g^{-1} dm^{-1} and c is expressed in g 100 cm^{-3}. The enantiomeric excess (*ee*) was determined by chiral stationary phase HPLC in a Dionex P680 chromatogram with a Dionex UVD170U detector (λ_{max} = 225, 250, 275 or 300 nm) using a flow rate of 0.7 - 0.8 mL min^{-1} with a Daicel Chiral pack IC column. The eluent used and retention times (t_{major} and t_{minor}) are given in parentheses.

2. Optimisations

2.1 Ligand screening and equivalence modification

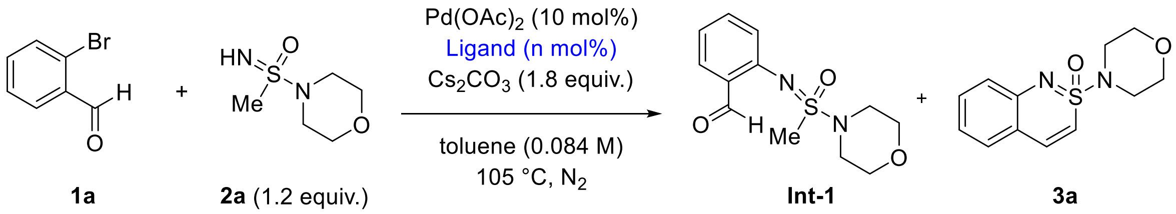

Entry	n (mol%)	Ligand	Time	NMR yield Int-1 [a]	NMR yield 3a [a]
1	15	BINAP	40 h	trace	trace
2	15	RuPhos	40 h	8%	52%
3	15	BrettPhos	40 h	trace	0%
4	15	RuPhos	17 h	80% (79%)	3%
5	10	RuPhos	17 h	48%	16%
6[b]	15	RuPhos	17 h	12%	31%
7[c]	15	RuPhos	17 h	trace	0%
8	20	RuPhos	17 h	50%	13%

Reaction carried out on 0.167 mmol scale. a: ^{1}H NMR yields determined using methyl 3,5-dinitrobenzoate as the internal standard. Isolated yields in parenthesis; b: 2.5 eq of Cs_2CO_3 was used; c: 1.8 eq of K_2CO_3 was used.

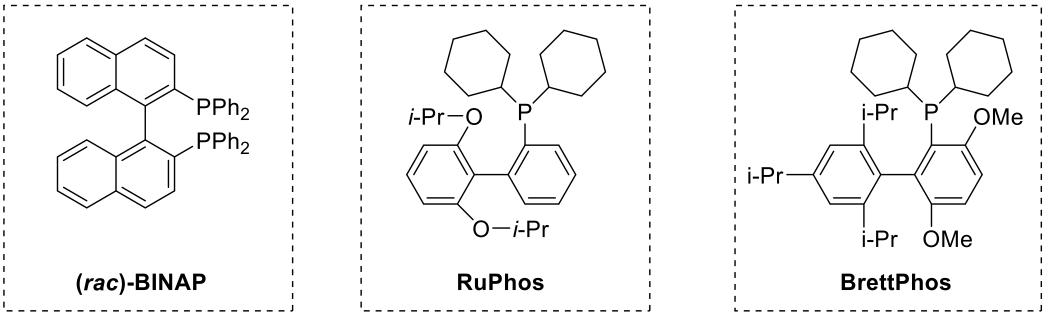

2.2 Second base screening in cyclisation

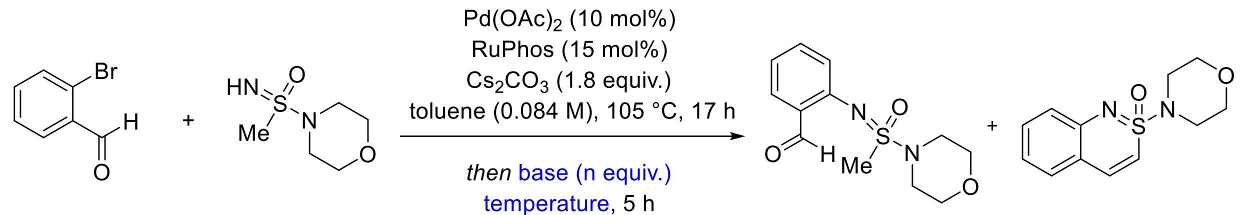

Entry	*Base*	*Equiv.*	*Temperature/ °C*	*NMR yield Int-1* [a]	*NMR yield 3a* [a]
1	LiHMDS	2.0	0 to r.t.	32%	6%
2	DBU	2.0	0 to r.t.	62%	4%
3	NaO*t*-Bu	2.0	0 to r.t.	0%	18%
4	NaO*t*-Bu	2.0	105	0%	53%
5	KO*t*-Bu	2.0	0 to r.t.	0%	9%
6	NaH	2.0	0 to r.t.	27%	11%
7	Cs_2CO_3	2.0	0 to r.t.	78%	4%
8	Cs_2CO_3	2.0	90	68%	5%
9	Cs_2CO_3	2.0	105	51%	20%
10	Cs_2CO_3	3.0	105	15%	53%
11	Cs_2CO_3	4.0	105	0%	60% (60%)
12[b]	Cs_2CO_3	4.0	105	0%	0%
13[c]	Cs_2CO_3	4.0	105	trace	trace

Reaction carried out on 0.167 mmol scale. a: ^{1}H NMR yields determined using methyl 3,5-dinitrobenozate as the internal standard; Isolated yields are in parentheses; b: no Pd source and ligand presented; c: *rac*-BINAP was used instead of RuPhos.

2.3 Further optimization on *N*-arylation

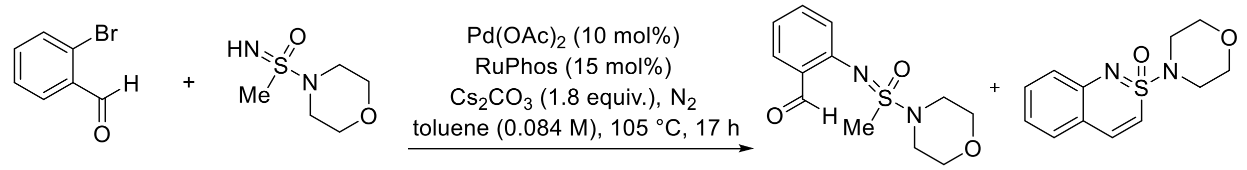

1a (1.0 equiv.) **2a** (1.2 equiv.) **Int-1** **3a**

Entry	***Variation from above***	***NMR yield Int-1*** [a]	***NMR yield 3a*** [a]
1	none	80% (79%)	3%
2	4 h reaction time	80%	trace

Reaction carried out on 0.167 mmol scale. a: ^{1}H NMR yields determined using methyl 3,5-dinitrobenozate as the internal standard;

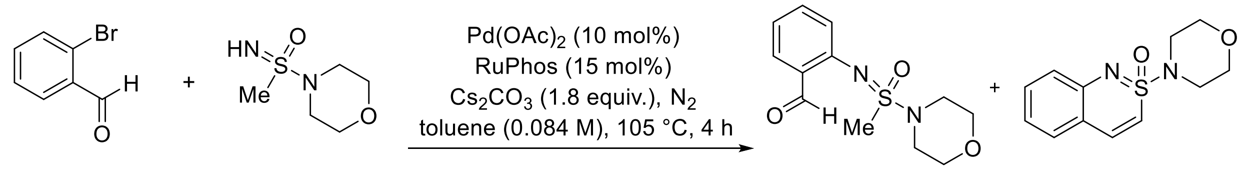

1a (1.0 equiv.) **2a** (1.2 equiv.) **Int-1** **3a**

Entry	***Variation from above***	***NMR yield Int-1*** [a]	***NMR yield 3a*** [a]
1	none	80%	trace
2	Dioxane instead of toluene	66%	11%
3	$Pd_2(dba)_3$ instead of $Pd(OAc)_2$	66%	11%
4	0.167 M of Toluene	93%	trace

Reaction carried out on 0.167 mmol scale. a: ^{1}H NMR yields determined using methyl 3,5-dinitrobenozate as the internal standard;

2.4 *N*-arylation and cyclisation in one pot

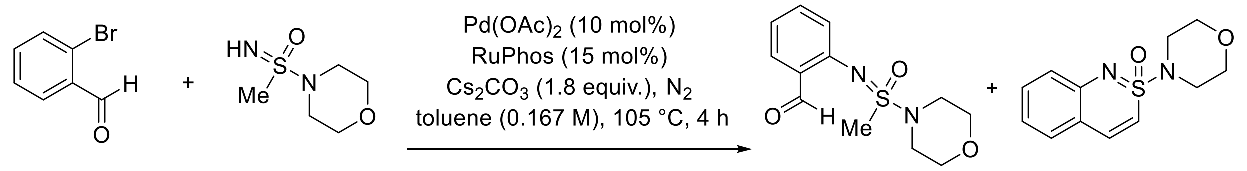

1a (1.0 equiv.) **2a** (1.2 equiv.) *then* Cs_2CO_3 (4 equiv.) 5 h, 105 °C **Int-1** **3a**

Entry	***Variation from above***	***NMR yield Int-1*** [a]	***NMR yield 3a*** [a]
1	none	0%	88%
2	5 mol% $Pd(OAc)_2$ and 7.5 mol% RuPhos	0%	96% (96%)
3	5 mol% $Pd(OAc)_2$ and 7.5 mol% RuPhos<br>No addition of Cs_2CO_3 (4 eq.)	99%	0%

Reaction carried out on 0.167 mmol scale. a: ^{1}H NMR yields determined using methyl 3,5-dinitrobenozate as the internal standard; Isolated yields are in parentheses.

2.5 Unsuccessful substrates

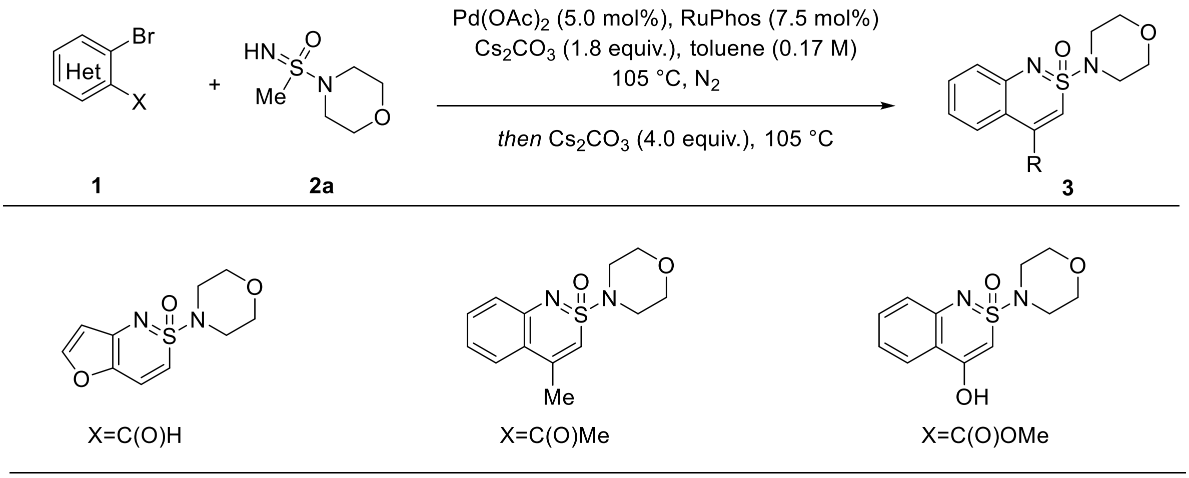

Isolated yield. Reactions performed on 0.17 mmol scale: bromobenzaldehyde (1.0 equiv.), sulfonimidamide (1.2 equiv.), $Pd(OAc)_2$ (5.0 mol%), RuPhos (7.5 mol%), Cs_2CO_3 (1.8 equiv.), Toluene (0.17 M); *then* Cs_2CO_3 (4.0 equiv.).

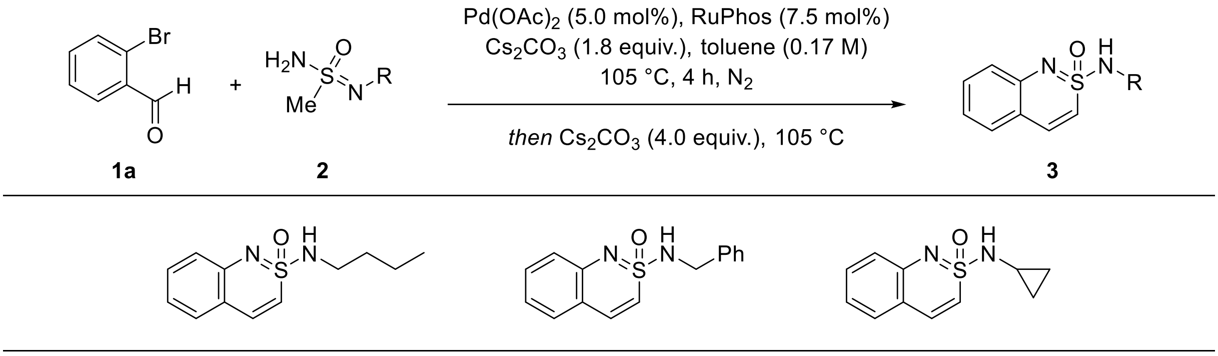

Isolated yields. Reactions performed on 0.17 mmol scale: bromobenzaldehyde (1.0 equiv.), sulfonimidamide (1.2 equiv.), $Pd(OAc)_2$ (5.0 mol%), RuPhos (7.5 mol%), Cs_2CO_3 (1.8 equiv.), toluene (0.167 M); *then* Cs_2CO_3 (4.0 equiv.);

3. Experimental procedures and characterisations

3.1 Preparation of BiPhONSO

_O_-([1,1'-biphenyl]-4-yl) hydroxylamine (BiPhONH$_2$) (SI-1)

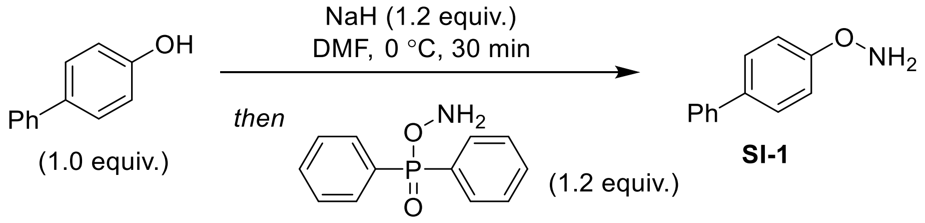

To a stirred solution of [1,1'-biphenyl]-4-ol (3.40 g, 20 mmol, 1.0 equiv.) in DMF (150 ml, 0.13 M) under nitrogen protection, sodium hydride (60% in mineral oil, 960 mg, 24 mmol, 1.2 equiv.) was added by 2 portions at 0 °C. The mixture was stirred at 0 °C for 30 min. Then (aminooxy)diphenylphosphine oxide (5.60 g, 24 mmol, 1.2 equiv.) was added by 3 portions at 0 °C and reaction mixture was stirred further at rt for 20 h. Once completed (judged by TLC), the mixture was diluted with water at 0 °C and the aqueous layer was extracted with EtOAc for 3 times. Combined organic layer was washed by brine twice, dried over anhydrous Na_2SO_4, filtered and concentrated under reduced pressure. The resultant crude mixture was dissolved in Et_2O and washed by NaOH (aq.) by twice. Organic layer was washed by brine, dried over anhydrous Na_2SO_4, filtered and concentrated under reduced pressure to afford O-([1,1'-biphenyl]-4-yl) hydroxylamine **SI-1** as a white solid (2.83 g, 76%).

^{1}H NMR (400 MHz, CDCl$_3$): δ 7.62 – 7.53 (m, 2H), 7.52 (d, J = 8.8 Hz, 2H), 7.42 (t, J = 7.7 Hz, 2H), 7.31 (t, J = 7.3 Hz, 1H), 7.21 (d, J = 8.9 Hz, 1H), 5.89 (br.s, 2H).

^{13}C NMR (101 MHz, CDCl$_3$): δ 160.7, 140.7, 134.0, 128.5, 127.8, 126.6, 126.5, 113.3.

HRMS (ESI +, m/z) Calcd for $C_{12}H_{12}NO^+$ $[M+H]^+$ 186.0913 found 186.0924.

Data is consistent with the literature.[1]

(([1,1'-biphenyl]-4-yloxy)imino)-λ⁴-sulfanone (BiPhONSO) (SI-2)

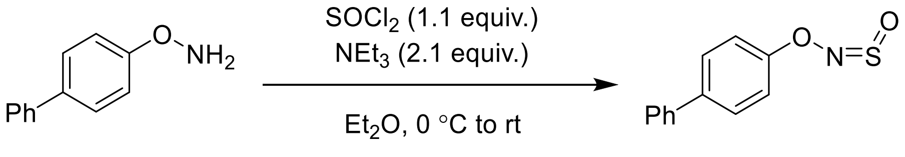

SI-1 **SI-2**

This procedure was adopted from the literature[1]. **SI-1** (1.20 g, 6.5 mmol, 1.0 equiv.) was added to a reaction flask and evacuated and back-filled with nitrogen three times. The compound was dissolved in degassed anhydrous diethyl ether (33 mL, 0.2 M) and placed in an ice bath at 0° C. Anhydrous triethylamine (1.90 mL, 13.7 mmol, 2.1 equiv.) was added. Thionyl chloride (0.52 mL, 7.2 mmol, 1.1 equiv.) was added dropwise. The reaction was stirred at 0 °C for 15 min, then taken out of the ice bath and stirred for a further 15 min. The reaction mixture was filtered through Celite (washed with diethyl ether). The solvent was concentrated in vacuo to afford **SI-2** as a gold solid (1.47 g, 98%).

^{1}H NMR (400 MHz, $CDCl_3$): δ 7.65 – 7.59 (m, 2H), 7.59 – 7.54 (m, 2H), 7.49 – 7.39 (m, 2H), 7.38 – 7.32 (m, 3H).

^{13}C NMR (101 MHz, $CDCl_3$): δ 158.2, 140.1, 138.4, 129.0, 128.5, 127.6, 127.1, 114.9.

HRMS (ESI +, m/z) Calcd for $C_{12}H_9NO_2SNa^+$ $[M+Na]^+$ 254.0246 found 254.0248.

Data is consistent with the literature.[1]

3.2 Preparation of Sulfonimidamide

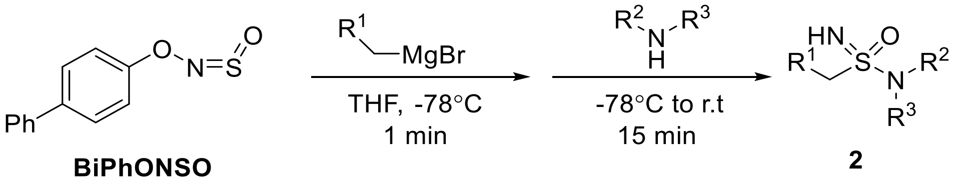

General Procedure A: This procedure was adopted from the literature[1]. BiPhONSO (1.0 equiv.) was added to an oven-dried reaction tube. The reaction tube was evacuated and back-filled with nitrogen three times before anhydrous THF (0.15 M) was added, and the solution was cooled to -78 °C. The first organometallic reagent (1.0 equiv.) was added dropwise over 30 seconds. The amine (1.5 equiv.) was added 1 min later. The reaction was immediately warmed to room temperature by being placed in a water bath and stirred for 15 min. The reaction mixture was then quenched with 2-propanol and purified directly by column chromatography to afford the desired sulfonimidamide **2**.

The procedure is adapted from literature.[1]

4-(*S*-Methylsulfonimidoyl)morpholine (2a)

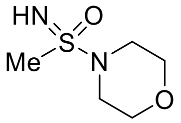

Prepared according to **general procedure A** using BiPhONSO (347 mg, 1.5 mmol, 1.0 equiv.), morpholine (0.20 ml, 2.3 mmol, 1.5 equiv.), MeMgBr (0.60 ml, 2.5 M in THF, 1.0 equiv.) and THF (10 ml, 0.15 M). Purification by flash column chromatography (SiO_2, ethyl acetate to 5% MeOH in ethyl acetate) afforded **2a** as a white solid (196 mg, 80%).

m.p = 54 – 56 °C.

^{1}H NMR (400 MHz, $CDCl_3$): δ 3.75 (t, *J* = 4.6 Hz, 4H), 3.21 (t, *J* = 3.7 Hz, 4H), 2.80 (s, 3H), 2.13 – 1.96 (br. s, 1H).

^{13}C NMR (101 MHz, $CDCl_3$): δ 66.8, 47.2, 34.2.

IR: ν_{max} (neat, cm^{-1}) = 3263, 1456, 1250, 1112, 933, 763.

HRMS (ESI +, m/z) Calcd for $C_5H_{12}N_2O_2SNa^+$ $[M+Na]^+$ 187.0512 found 187.0513.

Data is consistent with the literature[2]

8-(*S*-methylsulfonimidoyl)-1,4-dioxa-8-azaspiro[4.5]decane (2o)

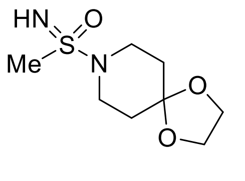

Prepared according to **general procedure A** using BiPhONSO (139 mg, 0.6 mmol, 1.0 equiv.), 1,4-Dioxa-8-azaspiro[4.5]decane (0.12 ml, 0.9 mmol, 1.5 equiv.), MeMgBr (0.24 ml, 2.5 M in THF, 1.0 equiv.) and THF (4 ml, 0.15 M). Purification by flash column chromatography (SiO_2, ethyl acetate to 3% MeOH in ethyl acetate) afforded **2o** as a white solid (81.1 mg, 61%).

m.p = 106 – 108 °C.

^{1}H NMR (400 MHz, $CDCl_3$): δ 3.98 (s, 4H), 3.38 (t, *J* = 5.8 Hz, 4H), 2.82 (s, 3H), 2.10 (br. s, 1H,), 1.81 (t, *J* = 5.8 Hz, 4H).

^{13}C NMR (101 MHz, $CDCl_3$): δ 106.5, 64.6, 45.5, 35.1.

IR: ν_{max} (neat, cm^{-1}) = 3263, 2963, 1469, 1251, 1145, 1045, 942, 762.

HRMS (ESI +, m/z) Calcd for $C_8H_{17}N_2O_3S^+$ $[M+H]^+$ 221.0954 found 221.0960.

2-(4-(*S*-methylsulfonimidoyl)piperazin-1-yl)pyrimidine (2p)

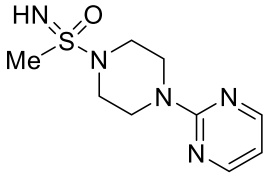

Prepared according to **general procedure A** using BiPhONSO (139 mg, 0.6 mmol, 1.0 equiv.), 1-(2-Pyrimidyl)piperazine (0.13 ml, 0.9 mmol, 1.5 equiv.), MeMgBr (0.24 ml, 2.5 M in THF, 1.0

equiv.) and THF (4 ml, 0.15 M). Purification by flash column chromatography (SiO_2, ethyl acetate to 3% MeOH in ethyl acetate) afforded **2p** as a white solid (77.6 mg, 54%).

m.p = 122 – 124 °C.

^{1}H NMR (400 MHz, $CDCl_3$): δ 8.33 (d, J = 4.8 Hz, 2H), 6.55 (t, J = 4.8 Hz, 1H), 3.98 – 3.91 (m, 4H), 3.30 (t, J = 5.0 Hz, 4H), 2.80 (s, 3H), 2.18 (br. s, 1H).

^{13}C NMR (101 MHz, $CDCl_3$): δ 161.6, 158.0, 110.7, 47.0, 43.8, 34.7.

IR: ν_{max} (neat, cm^{-1}) = 3295, 2855, 1587, 1508, 1450, 1264, 912.

HRMS (ESI +, m/z) Calcd for $C_9H_{16}N_5OS^+$ $[M+H]^+$ 242.1070 found 242.1077.

5-(*S*-methylsulfonimidoyl)-4,5,6,7-tetrahydrothieno[3,2-*c*]pyridine (2q)

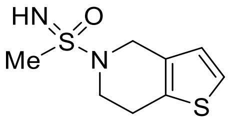

Prepared according to **general procedure A** using BiPhONSO (139 mg, 0.6 mmol, 1.0 equiv.), 4,5,6,7-Tetrahydrothieno-[3,2-C]Pyridine (0.11 ml, 0.9 mmol, 1.5 equiv.), MeMgBr (0.24 ml, 2.5 M in THF, 1.0 equiv.) and THF (4 ml, 0.15 M). Purification by flash column chromatography (SiO_2, pentane/ethyl acetate, 1:2 to 1:5) afforded **2q** as a white solid (93.3 mg, 72%).

m.p = 92 – 94 °C.

^{1}H NMR (400 MHz, $CDCl_3$): δ 7.14 (d, *J* = 5.2 Hz, 1H), 6.77 (d, *J* = 5.2 Hz, 1H), 4.44 – 4.39 (m, 2H), 3.72 – 3.56 (m, 2H), 2.96 (t, *J* = 4.9 Hz, 2H), 2.85 (s, 3H), 2.23 (br. s, 1H).

^{13}C NMR (101 MHz, $CDCl_3$): δ 133.0, 131.6, 124.9, 123.9, 46.9, 44.7, 37.0, 25.6.

IR: ν_{max} (neat, cm^{-1}) = 3275, 2933, 1252, 1237, 988, 926, 766.

HRMS (ESI +, m/z) Calcd for $C_8H_{13}N_2OS_2^+$ $[M+H]^+$ 217.0464 found 217.0468.

***N*-(3,4-dimethoxybenzyl)-*N*-methylmethanesulfonimidamide (2r)**

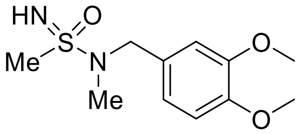

Prepared according to **general procedure A** using BiPhONSO (139 mg, 0.6 mmol, 1.0 equiv.), 3,4-dimethoxy-N-methylbenzylamine (0.17 ml, 0.9 mmol, 1.5 equiv.), MeMgBr (0.24 ml, 2.5 M in THF, 1.0 equiv.) and THF (4 ml, 0.15 M). Purification by flash column chromatography (SiO_2, ethyl acetate to 2% MeOH in ethyl acetate) afforded **2r** as a pink solid (106.4 mg, 69%).

m.p = 76 – 78 °C.

^{1}H NMR (400 MHz, $CDCl_3$): δ 6.89 – 6.80 (m, 3H), 4.27 (d, *J* = 2.4 Hz, 2H), 3.89 (s, 3H), 3.88 (s, 3H), 2.85 (s, 3H), 2.79 (s, 3H), 2.19 (br. s, 1H).

^{13}C NMR (101 MHz, $CDCl_3$): δ 149.4, 149.0, 128.9, 120.9, 111.4, 111.2, 56.13, 56.10, 55.0, 36.0, 35.6.

IR: ν_{max} (neat, cm^{-1}) = 3281, 2934, 1517, 1261, 1028, 912, 774.

HRMS (ESI +, m/z) Calcd for $C_{11}H_{19}N_2O_3S^+$ $[M+H]^+$ 259.1111 found 259.1122.

N'-benzylmethanesulfonimidamide (2s)

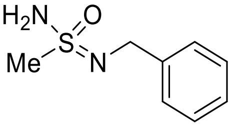

Prepared according to **general procedure A** using BiPhONSO (139 mg, 0.6 mmol, 1.0 equiv.), *n*-benzylamine (100 µL, 0.9 mmol, 1.5 equiv.), MeMgBr (0.24 ml, 2.5 M in Et_2O, 1.0 equiv.) and THF (4 ml, 0.15 M). Purification by flash column chromatography (SiO_2, ethyl acetate to 3% MeOH in ethyl acetate) afforded **2s** as colourless oil (68.0 mg, 62%).

^{1}H NMR (400 MHz, $CDCl_3$): δ 7.42 – 7.29 (m, 5H, Ar-*H*), 4.31 (s, 2H, NCH_2), 3.46 – 3.15 (brs, 2H, NH_2), 2.94 (s, 3H, SCH_3).

^{13}C NMR (101 MHz, $CDCl_3$): δ 137.4, 129.1, 128.1, 127.9, 48.2, 42.4.

IR: ν_{max} (neat, cm^{-1}) = 3261, 1496, 1250, 1027, 743.

HRMS (ESI +, m/z) Calcd for $C_8H_{13}N_2OS^+$ $[M+H]^+$ 185.0743 found 185.0745.

4-(ethylsulfonimidoyl)morpholine (2t)

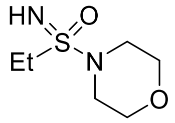

Prepared according to **general procedure A** using BiPhONSO (139 mg, 0.6 mmol, 1.0 equiv.), morpholine (0.08 ml, 0.9 mmol, 1.5 equiv.), EtMgBr (0.75 ml, 0.8 M in THF, 1.0 equiv.) and THF (4 ml, 0.15 M). Purification by flash column chromatography (SiO_2, ethyl acetate to 3% MeOH in ethyl acetate) afforded **2t** as brown oil (66.0 mg, 62%).

^{1}H NMR (400 MHz, $CDCl_3$): δ 3.69 (t, *J* = 4.7 Hz, 4H), 3.32 – 3.18 (m, 4H), 2.99 (dq, *J* = 13.7, 7.5 Hz, 1H), 2.87 (dq, *J* = 13.6, 7.4 Hz, 1H), 2.18 (br. s, 1H), 1.37 (t, *J* = 7.4 Hz, 3H).

^{13}C NMR (101 MHz, $CDCl_3$): δ 67.0, 46.9, 43.4, 8.2.

IR: ν_{max} (neat, cm^{-1}) = 3465, 1454, 1264, 1113, 939, 733.

HRMS (ESI +, m/z) Calcd for $C_6H_{14}N_2O_2SNa^+$ $[M+Na]^+$ 201.0668 found 201.0672.

4-(S-benzylsulfonimidoyl)morpholine (2u)

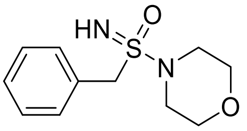

Prepared according to **general procedure A** using BiPhONSO (139 mg, 0.6 mmol, 1.0 equiv.), morpholine (0.08 ml, 0.9 mmol, 1.5 equiv.), Benzylmagnesium chloride solution (0.35 ml, 1.7 M in THF, 1.0 equiv.) and THF (4 ml, 1.5 M). Purification by flash column chromatography (SiO_2, pentane/ethyl acetate, 1:3 to 0:1) afforded **2u** as white solid (91.1 mg, 63%).

^{1}H NMR (400 MHz, $CDCl_3$): δ 7.49 – 7.34 (m, 5H, Ar-*H*), 4.27 (d, *J* = 13.5 Hz, 1H), 4.23 (d, *J* = 13.5 Hz, 1H), 3.70 – 3.56 (m, 4H), 3.17 (qdd, *J* = 12.2, 5.7, 3.7 Hz, 4H), 2.19 (br. s, 1H).

^{13}C NMR (101 MHz, $CDCl_3$): δ 131.1, 129.1, 128.9, 128.8, 67.1, 56.7, 46.9.

HRMS (ESI +, m/z) Calcd for $C_{11}H_{17}N_2O_2S$ $[M+H]^+$ 241.1005 found 241.1003.

Data is consistent with the literature.[1]

4-(allylsulfonimidoyl)morpholine (2v)

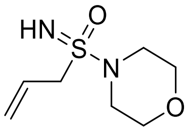

Prepared according to **general procedure A** using BiPhONSO (139 mg, 0.6 mmol, 1.0 equiv.), morpholine (0.08 ml, 0.9 mmol, 1.5 equiv.), allylmagnesium bromide solution (0.80 ml, 0.9 M in Et_2O, 1.2 equiv.) and THF (4 ml, 1.5 M). Purification by flash column chromatography (SiO_2, pentane/ethyl acetate, 1:3 to 0:1) afforded **2v** as yellow oil (45.1 mg, 40%).

^{1}H NMR (400 MHz, CDCl$_3$): δ 5.95 (ddt, *J* = 16.4, 10.5, 7.3 Hz, 1H), 5.43 – 5.32 (m, 2H), 3.78 – 3.70 (m, 2H), 3.68 (t, *J* = 4.7 Hz, 4H), 3.32 (td, *J* = 4.3, 2.3 Hz, 4H), 2.25 (br. s, 1H).

^{13}C NMR (101 MHz, CDCl$_3$): δ 126.3, 123.6, 67.1. 54.5, 47.0.

IR: v_{max} (neat, cm^{-1}) = 3368, 2972, 1614, 1380, 1110, 952.

HRMS (ESI +, m/z) Calcd for $C_7H_{14}N_2O_2SNa^+$ $[M+Na]^+$ 213.0668 found 213.0668.

***N*'-cyclopropylmethanesulfonimidamide (SI-3)**

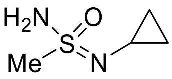

Prepared according to **general procedure A** using BiPhONSO (139 mg, 0.6 mmol, 1.0 equiv.), cyclopropyamine (62 µL, 0.9 mmol, 1.5 equiv.), MeMgBr (0.24 ml, 2.5 M in THF,1.0 equiv.) and THF (4 ml, 0.15 M). Purification by flash column chromatography (SiO_2, ethyl acetate to 4% MeOH in ethyl acetate) afforded ***N*'-cyclopropylmethanesulfonimidamide** as brown oil (58.5 mg, 73%).

^{1}H NMR (400 MHz, CDCl$_3$): δ 3.61 (br. s, 2H), 3.06 (s, 3H), 2.61 – 2.50 (m, 1H), 0.75 – 0.58 (m, 4H).

^{13}C NMR (101 MHz, $CDCl_3$): δ 41.5, 25.4, 6.7, 6.4.

IR: ν_{max} (neat, cm^{-1}) = 3259, 1325, 1248, 1062, 653.

HRMS (ESI +, m/z) Calcd for $C_4H_{10}N_2OSNa^+$ $[M+Na]^+$ 157.0406 found 157.0411.

***N*'-butylmethanesulfonimidamide (SI-4)**

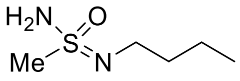

Prepared according to **general procedure A** using BiPhONSO (139 mg, 0.6 mmol, 1.0 equiv.), *n*-butylamine (90 µL, 0.9 mmol, 1.5 equiv.), MeMgBr (0.24 ml, 2.5 M in Et_2O, 1.0 equiv.) and THF (4 ml, 0.15 M). Purification by flash column chromatography (SiO_2, ethyl acetate to 5% MeOH in ethyl acetate) afforded ***N*'-butylmethanesulfonimidamide** as colourless oil (30.0 mg, 33%).

^{1}H NMR (400 MHz, $CDCl_3$): δ 3.68 (brs, 2H, NH_2), 3.07 (t, J = 7.1 Hz, 2H, NCH_2), 2.98 (s, 3H, SCH_3), 1.56 – 1.44 (m, 2H, NCH_2CH_2), 1.41 – 1.28 (m, 2H, $NCH_2CH_2CH_2$), 0.90 (t, J = 7.3 Hz, 3H, $NCH_2CH_2CH_2CH_3$).

^{13}C NMR (101 MHz, $CDCl_3$): δ 43.8, 41.4, 32.3, 19.9, 13.7.

IR: ν_{max} (neat, cm^{-1}) = 3284, 2960, 1375, 1244, 1047, 735.

HRMS (ESI +, m/z) Calcd for $C_5H_{15}N_2OS^+$ $[M+H]^+$ 151.0900 found 151.0899.

Data is consistent with the literature.[6]

3.3 Preparation of cyclised sulfonimidamide (secondary amine)

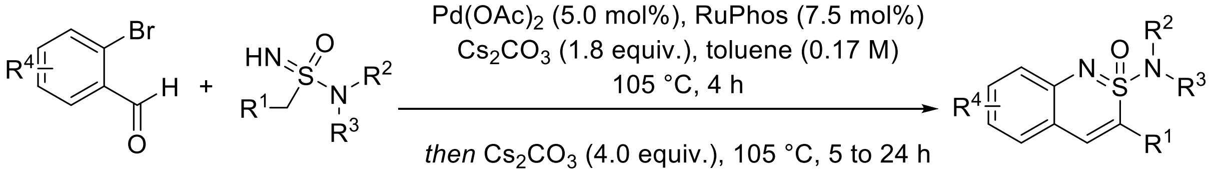

1 (1.0 eq) **2** (1.2 eq) **3**

General Procedure B: An oven-dried 10 mL microwave vial with a stirring bar was charged with bromobenzaldehyde **1** (1.0 equiv., if solid), sulfonimidamide **2** (1.2 equiv.), $Pd(OAc)_2$ (5.0 mol%), RuPhos (7.5 mol%) and Cs_2CO_3 (1.8 equiv.). The vial was evacuated and back-filled with nitrogen gas three times before addition of degassed, anhydrous toluene (0.167 M). The mixture was heated at 105 °C for 4 hours before being cooled to room temperature. Cs_2CO_3 (4.0 equiv.) was then added and vial was sealed again. The reaction mixture was continued to stir at 105 °C for 5 to 24 hours. Once judged complete by TLC, the reaction mixture was cooled to room temperature and ethyl acetate (10 mL) was added. After filtration through filter paper and removal of solvent *in vacuo*, the resultant crude mixture was purified by column chromatography to give the desired cyclised sulfonimidamide product **3**.

Note:

1. If bromobenzaldehyde was liquid, it was added to the reaction mixture after addition of toluene.
2. Caesium carbonate can be dried by heating 120 °C under high-vacuum overnight.
3. A steel block was used for heating the reaction, but oil bath was suggested in larger scale (0.87 mmol).
4. The quality of bromobenzaldehyde is also important. Samples containing bromobenzoic acid were unproductive and were not used.

2-Morpholinobenzo[*c*][1,2]thiazine 2-oxide (3a)

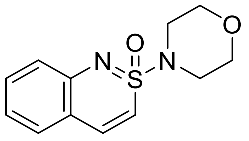

Prepared according to **general procedure B** using 2-bromobenzaldehyde **1a** (20 µL, 0.17 mmol, 1.0 equiv.), 4-(*S*-methylsulfonimidoyl) morpholine **2a** (32.8 mg, 0.20 mmol, 1.2 equiv.), $Pd(OAc)_2$ (1.9 mg, 0.008 mmol, 5.0 mol%), RuPhos (5.8 mg, 0.0125 mmol, 7.5 mol%), Cs_2CO_3 (97.7 mg, 0.30 mmol, 1.8 equiv.) and anhydrous toluene (1 mL, 0.17 M), followed by second addition of Cs_2CO_3 (217.2 mg, 0.67 mmol, 4.0 equiv.) and stirring for 5 h. Purification by flash column chromatography (SiO_2, pentane/ethyl acetate, 3:1 to 1:1) afforded **3a** as an orange solid (39.8 mg, 96%).

m.p = 78 – 80 °C.

^{1}H NMR (400 MHz, $CDCl_3$): δ 7.73 (d, *J* = 10.0 Hz, 1H), 7.41 (ddd, *J* = 8.5, 7.1, 1.6 Hz, 1H), 7.32 (dd, *J* = 7.8, 1.6 Hz, 1H), 7.25 (dd, *J* = 8.4, 1.1 Hz, 1H), 6.99 (ddd, *J* = 8.0, 7.1, 1.2 Hz, 1H), 6.20 (d, *J* = 10.0 Hz, 1H), 3.82 – 3.67 (m, 4H), 3.07 (t, *J* = 4.9 Hz, 4H).

^{13}C NMR (101 MHz, $CDCl_3$): δ 146.6, 142.7, 132.0, 129.5, 123.9, 120.4, 116.2, 105.8, 66.6, 45.7.

IR: ν_{max} (neat, cm^{-1}) = 3056, 2863, 1611, 1346, 1297, 1260, 1115, 1074, 942, 778, 739.

HRMS (ESI +, m/z) Calcd for $C_{12}H_{14}N_2O_2SNa^+$ $[M+Na]^+$ 273.0668 found 273.0676.

2-Morpholinobenzo[*c*][1,2]thiazine 2-oxide (3a) (from ArCl)

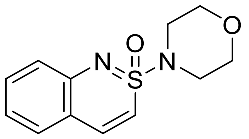

Prepared according to **general procedure B** using 2-chlorobenzaldehyde (19 µL, 0.17 mmol, 1.0 equiv.), 4-(*S*-methylsulfonimidoyl) morpholine **2a** (32.8 mg, 0.20 mmol, 1.2 equiv.), $Pd(OAc)_2$ (1.9 mg, 0.008 mmol, 5.0 mol%), RuPhos (5.8 mg, 0.0125 mmol, 7.5 mol%), Cs_2CO_3 (97.7 mg, 0.30 mmol, 1.8 equiv.) and anhydrous toluene (1 mL, 0.17 M), followed by second addition of Cs_2CO_3 (217.2 mg, 0.67 mmol, 4.0 equiv.) and stirring for 17 h. Purification by flash column chromatography (SiO_2, pentane/ethyl acetate, 3:1 to 1:1) afforded **3a** as an orange solid (39.5 mg, 95%). (**3a** Data is consistent with the previous data.)

6-Methoxy-2-morpholinobenzo[*c*][1,2]thiazine 2-oxide (3b)

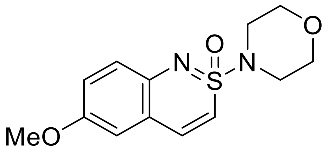

Prepared according to **general procedure B** using 2-bromo-5-methoxybenzaldehyde **1b** (71.6 mg, 0.33 mmol, 1.0 equiv.), 4-(*S*-methylsulfonimidoyl)morpholine **2a** (65.6 mg, 0.40 mmol, 1.2 equiv.), $Pd(OAc)_2$ (3.7 mg, 0.017 mmol, 5.0 mol%), RuPhos (11.7 mg, 0.025 mmol, 7.5 mol%), Cs_2CO_3 (195.4 mg, 0.60 mmol, 1.8 equiv.) and anhydrous toluene (2 mL, 0.17 M), followed by second addition of Cs_2CO_3 (434.4 mg, 1.3 mmol, 4.0 equiv.) and stirring for 5 h. Purification by flash column chromatography (SiO_2, pentane/ethyl acetate, 3:1 to 1:1) afforded **3b** as a yellow solid (70.5 mg, 76%).

m.p = 96 – 98 °C.

^{1}H NMR (400 MHz, $CDCl_3$): δ 7.68 (d, J = 9.9 Hz, 1H), 7.20 (d, J = 9.0 Hz, 1H), 7.07 (dd, J = 9.0, 3.0 Hz, 1H), 6.77 (d, J = 3.0 Hz, 1H), 6.19 (d, J = 9.9 Hz, 1H), 3.80 (s, 3H), 3.78 – 3.68 (m, 4H), 3.07 (t, J = 4.8 Hz, 4H).

^{13}C NMR (101 MHz, $CDCl_3$): δ 153.4, 142.1, 140.9, 124.9, 121.2, 116.0, 110.7, 105.9, 66.7, 55.9, 45.8.

IR: ν_{max} (neat, cm^{-1}) = 3062, 2947, 1592, 1541, 1299, 1239, 1115, 941, 741.

HRMS (ESI +, m/z) Calcd for $C_{13}H_{17}N_2O_3S^+$ $[M+H]^+$ 281.0954 found 281.0963.

6-Chloro-2-morpholinobenzo[*c*][1,2]thiazine 2-oxide (3c)

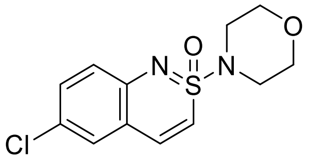

Prepared according to **general procedure B** using 2-bromo-5-chlorobenzaldehyde **1c** (36.6 mg, 0.17 mmol, 1.0 equiv.), 4-(*S*-methylsulfonimidoyl)morpholine **2a** (32.8 mg, 0.20 mmol, 1.2 equiv.), $Pd(OAc)_2$ (1.9 mg, 0.008 mmol, 5.0 mol%), RuPhos (5.8 mg, 0.0125 mmol, 7.5 mol%), Cs_2CO_3 (97.7 mg, 0.30 mmol, 1.8 equiv.) and anhydrous toluene (1 mL, 0.17 M), followed by second addition of Cs_2CO_3 (217.2 mg, 0.67 mmol, 4.0 equiv.) and stirring for 5 h. Purification

by flash column chromatography (SiO_2, pentane/ethyl acetate, 3:1 to 1:1) afforded **3c** as a pale-yellow solid (35.1 mg, 74%).

m.p = 112 – 114 °C.

^{1}H NMR (400 MHz, CDCl$_3$): δ 7.65 (d, *J* = 10.0 Hz, 1H), δ 7.34 (dd, *J* = 8.8, 2.5 Hz, 1H), 7.30 (d, *J* = 2.5 Hz, 1H), 7.18 (d, *J* = 8.8 Hz, 1H), 6.24 (d, *J* = 10.0 Hz, 1H), 3.81 – 3.67 (m, 4H), 3.06 (t, *J* = 4.8 Hz, 4H).

^{13}C NMR (101 MHz, CDCl$_3$): δ 145.0, 141.4, 132.0, 128.2, 125.3, 125.1, 116.8, 107.0, 66.6, 45.7.

IR: ν_{max} (neat, cm^{-1}) = 3057, 1606, 1361, 1298, 1238, 1115, 1074, 943, 764, 739.

HRMS (ESI +, m/z) Calcd for $C_{12}H_{13}ClN_2O_2SNa^+$ $[M+Na]^+$ 307.0279 found 307.0286.

2-Morpholino-6-nitrobenzo[*c*][1,2]thiazine 2-oxide (3d)

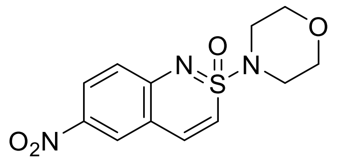

Prepared according to **general procedure B** using 2-bromo-5-nitrobenzaldehyde **1d** (38.3 mg, 0.17 mmol, 1.0 equiv.), 4-(*S*-methylsulfonimidoyl)morpholine **2a** (32.8 mg, 0.20 mmol, 1.2 equiv.), $Pd(OAc)_2$ (1.9 mg, 0.008 mmol, 5.0 mol%), RuPhos (5.8 mg, 0.0125 mmol, 7.5 mol%), Cs_2CO_3 (97.7 mg, 0.30 mmol, 1.8 equiv.) and anhydrous toluene (1 mL, 0.17 M), followed by second addition of Cs_2CO_3 (217.2 mg, 0.67 mmol, 4.0 equiv.) and stirring for 5 h. Purification by flash column chromatography (SiO_2, pentane/ethyl acetate, 3:1 to 1:2) afforded **3d** as an orange solid (37.5 mg, 76%).

m.p = 190 – 192 °C.

^{1}H NMR (400 MHz, CDCl$_3$): δ 8.31 (d, *J* = 2.7 Hz, 1H), 8.24 (dd, *J* = 9.2, 2.7 Hz, 1H), 7.82 (d, *J* = 10.1 Hz, 1H), 7.29 (d, *J* = 9.2 Hz, 1H), 6.38 (d, *J* = 10.1 Hz, 1H), 3.76 (q, *J* = 4.3 Hz, 4H), 3.12 – 3.08 (m, 4H).

^{13}C NMR (101 MHz, CDCl$_3$): δ 151.6, 142.1, 140.8, 126.4, 126.1, 124.6, 114.8, 108.5, 66.5, 45.7.

IR: ν_{max} (neat, cm^{-1}) = 3064, 2925, 1609, 1507, 1346, 1312, 1259, 1093, 1003, 945, 748.

HRMS (ESI +, m/z) Calcd for $C_{12}H_{13}N_3O_4SNa^+$ $[M+Na]^+$ 318.0519 found 318.0524.

6-Fluoro-2-morpholinobenzo[*c*][1,2]thiazine 2-oxide (3e)

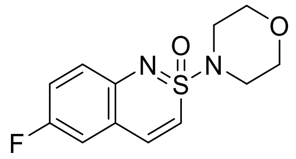

Prepared according to **general procedure B** using 2-bromo-5-fluorobenzaldehyde **1e** (33.8 mg, 0.17 mmol, 1.0 equiv.), 4-(*S*-methylsulfonimidoyl)morpholine **2a** (32.8 mg, 0.20 mmol, 1.2 equiv.), $Pd(OAc)_2$ (1.9 mg, 0.008 mmol, 5.0 mol%), RuPhos (5.8 mg, 0.0125 mmol, 7.5 mol%), Cs_2CO_3 (97.7 mg, 0.30 mmol, 1.8 equiv.) and anhydrous toluene (1 mL, 0.17 M), followed by second addition of Cs_2CO_3 (217.2 mg, 0.67 mmol, 4.0 equiv.) and stirring for 5 h. Purification by flash column chromatography (SiO_2, pentane/ethyl acetate, 3:1 to 1:1) afforded **3e** as a red solid (25.0 mg, 56%).

m.p = 90 – 92 °C.

^{1}H NMR (400 MHz, $CDCl_3$): δ 7.67 (d, *J* = 10.0 Hz, 1H), δ 7.22 (dd, *J* = 9.0, 5.0 Hz, 1H), 7.16 (ddd, *J* = 9.0, 8.6, 2.9 Hz, 1H), 7.01 (dd, J = 8.5, 2.9 Hz, 1H), 6.25 (d, J = 10.0 Hz, 1H), 3.82 – 3.66 (m, 4H), 3.08 (t, J = 4.8 Hz, 4H).

^{13}C NMR (101 MHz, $CDCl_3$): δ 156.6 (d, *J* = 239.2 Hz), 142.8, 141.5 (d, *J* = 3.3 Hz), 125.3 (d, *J* = 7.9 Hz), 120.1 (d, *J* = 23.6 Hz), 116.0 (d, *J* = 8.6 Hz), 113.7 (d, *J* = 22.5 Hz), 107.0, 66.6, 45.8.

^{19}F NMR (377 MHz, $CDCl_3$): δ -123.38.

IR: ν_{max} (neat, cm^{-1}) = 3038, 2881, 1599, 1544, 1471, 1304, 1248, 1152, 949, 747.

HRMS (ESI +, m/z) Calcd for $C_{12}H_{13}FN_2O_2SNa^+$ $[M+Na]^+$ 291.0574 found 291.0574.

7-Fluoro-2-morpholinobenzo[*c*][1,2]thiazine 2-oxide (3f)

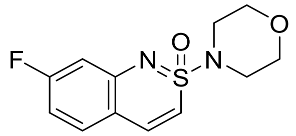

Prepared according to **general procedure B** using 2-bromo-4-fluorobenzaldehyde **1f** (33.8 mg, 0.17 mmol, 1.0 equiv.), 4-(*S*-methylsulfonimidoyl)morpholine **2a** (32.8 mg, 0.20 mmol, 1.2 equiv.), $Pd(OAc)_2$ (1.9 mg, 0.008 mmol, 5.0 mol%), RuPhos (5.8 mg, 0.0125 mmol, 7.5 mol%), Cs_2CO_3 (97.7 mg, 0.30 mmol, 1.8 equiv.) and anhydrous toluene (1 mL, 0.17 M), followed by second addition of Cs_2CO_3 (217.2 mg, 0.67 mmol, 4.0 equiv.) and stirring for 5 h. Purification by flash column chromatography (SiO_2, pentane/ethyl acetate, 3:1 to 1:1) afforded **3f** as a white solid (32.7 mg, 73%).

m.p = 90 – 92 °C.

^{1}H NMR (400 MHz, CDCl$_3$): δ 7.69 (d, *J* = 10.0 Hz, 1H), 7.28 (dd, *J* = 8.5, 6.3 Hz, 1H), 6.90 (dd, *J* = 10.7, 2.5 Hz, 1H), 6.72 (td, *J* = 8.5, 2.5 Hz, 1H), 6.14 (d, *J* = 10.0 Hz, 1H), 3.81 – 3.67 (m, 4H,), 3.06 (t, *J* = 4.9 Hz, 4H).

^{13}C NMR (101 MHz, CDCl$_3$): δ 165.0 (d, *J* = 250.9 Hz), 148.5 (d, *J* = 13.8 Hz), 142.0, 131.4 (d, *J* = 11.1 Hz), 113.0 (d, *J* = 1.9 Hz), 109.4 (d, *J* = 23.0 Hz), 109.1 (d, *J* = 23.6 Hz), 104.6 (d, *J* = 3.2 Hz), 66.56, 45.64.

^{19}F NMR (377 MHz, CDCl$_3$): δ -107.00 (ddd, *J* = 10.6, 8.3, 6.3 Hz).

IR: ν_{max} (neat, cm^{-1}) = 3061, 1621, 1544, 1297, 1254, 1213, 1114, 942, 770, 737.

HRMS (ESI +, m/z) Calcd for $C_{12}H_{13}FN_2O_2SNa^+$ $[M+Na]^+$ 291.0574 found 291.0576.

5-Fluoro-2-morpholinobenzo[*c*][1,2]thiazine 2-oxide (3g)

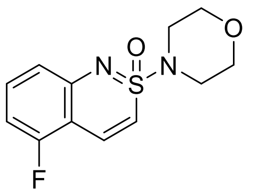

Prepared according to **general procedure B** using 2-bromo-6-fluorobenzaldehyde **1g** (33.8 mg, 0.17 mmol, 1.0 equiv.), 4-(*S*-methylsulfonimidoyl)morpholine **2a** (32.8 mg, 0.20 mmol, 1.2 equiv.), $Pd(OAc)_2$ (1.9 mg, 0.008 mmol, 5.0 mol%), RuPhos (5.8 mg, 0.0125 mmol, 7.5 mol%), Cs_2CO_3 (97.7 mg, 0.30 mmol, 1.8 equiv.) and anhydrous toluene (1 mL, 0.17 M), followed by second addition of Cs_2CO_3 (217.2 mg, 0.67 mmol, 4.0 equiv.) and stirring for 5 h. Purification

by flash column chromatography (SiO_2, pentane/ethyl acetate, 3:1 to 1:1) afforded **3g** as an off-white solid (28.7 mg, 64%).

m.p = 84 – 86 °C.

^{1}H NMR (400 MHz, $CDCl_3$): δ 8.05 (d, J = 10.2 Hz, 1H), 7.32 (td, *J* = 8.2, 6.5 Hz, 1H), 7.02 (d, J = 8.2 Hz, 1H), 6.67 (dd, J = 9.8, 8.2 Hz, 1H), 6.24 (d, J = 10.2 Hz, 1H), 3.82 – 3.66 (m, 4H), 3.08 (t, J = 4.2 Hz, 4H).

^{13}C NMR (101 MHz, $CDCl_3$): δ 159.8 (d, *J* = 253.4 Hz), 147.7, 134.9 (d, *J* = 6.6 Hz), 132.3 (d, *J* = 10.7 Hz), 119.6 (d, *J* = 3.5 Hz), 106.2, 106.0 (d, *J* = 3.0 Hz), 105.3 (d, *J* = 20.5 Hz), 66.6, 45.7.

^{19}F NMR (377 MHz, $CDCl_3$): δ -122.08 (dd, J = 9.9, 6.5 Hz).

IR: ν_{max} (neat, cm^{-1}) = 3059, 1626, 1450, 1355, 1304, 1244, 1115, 1085, 948, 763, 737.

HRMS (ESI +, m/z) Calcd for $C_{12}H_{13}FN_2O_2SNa^+$ $[M+Na]^+$ 291.0574 found 291.0583.

8-methyl-2-morpholinobenzo[*c*][1,2]thiazine 2-oxide (3h)

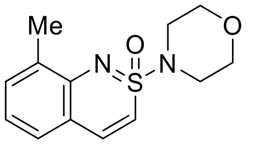

Prepared according to **general procedure B** using 2-bromo-3-methylbenzaldehyde **1h** (33.4 mg, 0.17 mmol, 1.0 equiv.), 4-(*S*-methylsulfonimidoyl)morpholine **2a** (32.8 mg, 0.20 mmol, 1.2 equiv.), $Pd(OAc)_2$ (1.9 mg, 0.008 mmol, 5.0 mol%), RuPhos (5.8 mg, 0.0125 mmol, 7.5 mol%), Cs_2CO_3 (97.7 mg, 0.30 mmol, 1.8 equiv.) and anhydrous toluene (1 mL, 0.17 M), followed by second addition of Cs_2CO_3 (217.2 mg, 0.67 mmol, 4.0 equiv.) and stirring for 5 h. Purification by flash column chromatography (SiO_2, pentane/ethyl acetate, 3:1 to 1:1) afforded **3h** as yellow gum (13.2 mg, 30%).

^{1}H NMR (400 MHz, $CDCl_3$): δ 7.72 (d, *J* = 9.9 Hz, 1H), 7.31 (d, *J* = 7.2 Hz, 1H), 7.18 (d, *J* = 7.8 Hz, 1H), 6.91 (t, *J* = 7.5 Hz, 1H), 6.17 (d, *J* = 9.9 Hz, 1H), 3.74 (q, *J* = 4.1 Hz, 4H), 3.05 (t, *J* = 4.7 Hz, 4H), 2.42 (s, 3H).

^{13}C NMR (101 MHz, $CDCl_3$): δ 145.4, 143.1, 132.6, 131.6, 127.3, 119.7, 115.7, 105.1, 66.6, 45.7, 17.9.

IR: ν_{max} (neat, cm^{-1}) = 2925, 1649, 1453, 1340, 1296, 1113, 1070, 939, 765.

HRMS (ESI +, m/z) Calcd for $C_{13}H_{16}N_2O_2SK^+$ $[M+K]^+$ 303.0564 found 303.0540.

2-Morpholino-[1,3]dioxolo[4',5':4,5]benzo[1,2-*c*][1,2]thiazine 2-oxide (3i)

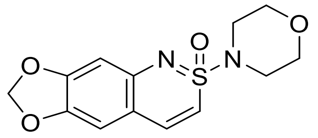

Prepared according to **general procedure B** using 6-Bromopiperonal **1i** (38.2 mg, 0.17 mmol, 1.0 equiv.), 4-(*S*-methylsulfonimidoyl)morpholine **2a** (32.8 mg, 0.20 mmol, 1.2 equiv.), $Pd(OAc)_2$ (1.9 mg, 0.008 mmol, 5.0 mol%), RuPhos (5.8 mg, 0.0125 mmol, 7.5 mol%), Cs_2CO_3 (97.7 mg, 0.30 mmol, 1.8 equiv.) and anhydrous toluene (1 mL, 0.17 M), followed by second addition of Cs_2CO_3 (217.2 mg, 0.67 mmol, 4.0 equiv.) and stirring for 24 h. Purification by flash column chromatography (SiO_2, pentane/ethyl acetate, 3:1 to 1:1) afforded **3i** as a yellow solid (34.1 mg, 70%).

m.p = 168 – 170 °C.

^{1}H NMR (400 MHz, $CDCl_3$): δ 7.57 (d, *J* = 9.9 Hz, 1H), 6.69 (s, 1H), 6.67 (s, 1H), 5.97 (d, *J* = 9.9 Hz, 1H), 5.95 (s, 2H), 3.80 – 3.66 (m, 4H), 3.03 (t, 4H).

^{13}C NMR (101 MHz, $CDCl_3$): δ 151.5, 144.3, 142.4, 142.0, 109.9, 106.2, 103.3, 101.5, 100.7, 66.6, 45.7.

IR: ν_{max} (neat, cm^{-1}) = 3060, 1617, 1556, 1478, 1290, 1250, 1039, 972, 940, 768, 741.

HRMS (ESI +, m/z) Calcd for $C_{13}H_{14}N_2O_4SNa^+$ $[M+Na]^+$ 317.0567 found 317.0574.

2-Morpholinopyrido[3,4-*c*][1,2]thiazine 2-oxide (3j)

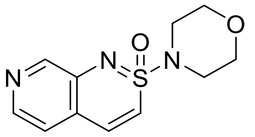

Prepared according to **general procedure B** using 3-bromo-4-formylpyridine **1j** (31.0 mg, 0.17 mmol, 1.0 equiv.), 4-(*S*-methylsulfonimidoyl)morpholine **2a** (32.8 mg, 0.20 mmol, 1.2 equiv.), $Pd(OAc)_2$ (1.9 mg, 0.008 mmol, 5.0 mol%), RuPhos (5.8 mg, 0.0125 mmol, 7.5 mol%), Cs_2CO_3 (97.7 mg, 0.30 mmol, 1.8 equiv.) and anhydrous toluene (1 mL, 0.17 M), followed by second addition of Cs_2CO_3 (217.2 mg, 0.67 mmol, 4.0 equiv.) and stirring for 5 h. Purification by flash column chromatography (SiO_2, pentane/ethyl acetate, 1:1 to pure ethyl acetate) afforded **3j** as a white solid (25.8 mg, 62%).

m.p = 142 – 144 °C.

^{1}H NMR (400 MHz, $CDCl_3$): δ 8.66 (s, 1H), 8.21 (d, *J* = 5.1 Hz, 1H), 7.71 (d, *J* = 10.0 Hz, 1H), 7.17 (d, *J* = 5.1 Hz, 1H), 6.44 (d, *J* = 10.0 Hz, 1H), 3.83 – 3.68 (m, 4H), 3.10 (t, *J* = 4.8 Hz, 4H).

^{13}C NMR (101 MHz, $CDCl_3$): δ 147.3, 141.3, 140.4, 140.1, 121.1, 119.9, 111.8, 66.6, 45.8.

IR: ν_{max} (neat, cm^{-1}) = 3051, 2924, 1602, 1410, 1337, 1261, 1240, 1114, 1076, 1003, 945, 749, 735, 613.

HRMS (ESI +, m/z) Calcd for $C_{11}H_{14}N_3O_2S^+$ $[M+H]^+$ 252.0801 found 252.0810.

2-Morpholinopyrido[3,2-*c*][1,2]thiazine 2-oxide (3k)

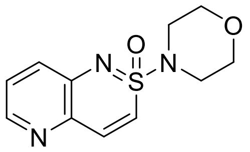

Prepared according to **general procedure B** using 3-bromo-2-formylpyridine **1k** (31.0 mg, 0.17 mmol, 1.0 equiv.), 4-(S-methylsulfonimidoyl)morpholine **2a** (32.8 mg, 0.20 mmol, 1.2 equiv.), $Pd(OAc)_2$ (1.9 mg, 0.008 mmol, 5.0 mol%), RuPhos (5.8 mg, 0.0125 mmol, 7.5 mol%), Cs_2CO_3 (97.7 mg, 0.30 mmol, 1.8 equiv.) and anhydrous toluene (1 mL, 0.17 M), followed by second addition of Cs_2CO_3 (217.2 mg, 0.67 mmol, 4.0 equiv.) and stirring for 5 h. Purification by flash column chromatography (SiO_2, pentane/ethyl acetate, 2:1 to pure ethyl acetate) afforded **3k** as yellow oil (5.0 mg, 12%).

^{1}H NMR (400 MHz, $CDCl_3$): δ 8.35 (dd, *J* = 4.4, 1.5 Hz, 1H), 7.97 (d, *J* = 10.0 Hz, 1H), 7.56 (dd, *J* = 8.4, 1.5 Hz, 1H), 7.31 (dd, *J* = 8.4, 4.4 Hz, 1H), 6.42 (d, *J* = 10.0 Hz, 1H), 3.85 – 3.67 (m, 4H,), 3.10 (t, *J* = 4.8 Hz, 4H).

^{13}C NMR (101 MHz, $CDCl_3$): δ 143.5, 143.23, 143.21, 134.3, 131.5, 125.9, 110.1, 66.6, 45.8.

IR: ν_{max} (neat, cm^{-1}) = 1593, 1431, 1338, 1243, 1115, 1006, 944, 745.

HRMS (ESI +, m/z) Calcd for $C_{11}H_{13}N_3O_2SNa^+$ $[M+Na]^+$ 274.0621 found 274.0621.

2-Morpholinothieno[3,2-*c*][1,2]thiazine 2-oxide (3l)

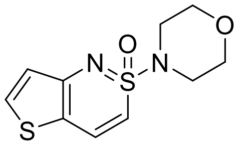

Prepared according to **general procedure B** using 3-Bromothiophene-2-Carboxaldehyde **1l** (18.0 µL, 0.17 mmol, 1.0 equiv.), 4-(S-methylsulfonimidoyl)morpholine **2a** (32.8 mg, 0.20 mmol, 1.2 equiv.), $Pd(OAc)_2$ (1.9 mg, 0.008 mmol, 5.0 mol%), RuPhos (5.8 mg, 0.0125 mmol, 7.5 mol%), Cs_2CO_3 (97.7 mg, 0.30 mmol, 1.8 equiv.) and anhydrous toluene (1 mL, 0.17 M), followed by second addition of Cs_2CO_3 (217.2 mg, 0.67 mmol, 4.0 equiv.) and stirring for 18 h. Purification by flash column chromatography (SiO_2, pentane/ethyl acetate, 4:1 to 1:1) afforded **3l** as yellow oil (33.2 mg, 78%).

^{1}H NMR (400 MHz, $CDCl_3$): δ 7.73 (dd, *J* = 9.9, 0.7 Hz, 1H), 7.46 (d, *J* = 5.4 Hz, 1H), 6.93 (dd, *J* = 5.4, 0.7 Hz, 1H), 5.78 (dd, *J* = 9.9, 0.7 Hz, 1H), δ 3.83 – 3.65 (m, 4H), 3.14 – 3.00 (m, 4H).

^{13}C NMR (101 MHz, $CDCl_3$): δ 151.4, 135.5, 130.6, 123.9, 111.1, 95.8, 66.6, 45.9.

IR: ν_{max} (neat, cm^{-1}) = 3067, 2857, 1638, 1561, 1488, 1394, 1294, 1114, 1075, 946, 756, 710, 635.

HRMS (ESI +, m/z) Calcd $C_{10}H_{12}N_2O_2S_2Na^+$ $[M+Na]^+$ 279.0232 found 279.0234.

2-Morpholino-4-phenylbenzo[*c*][1,2]thiazine 2-oxide (3m)

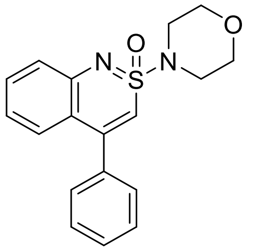

Prepared according to **general procedure B** using 2-Bromobenzophenone **1m** (43.3 mg, 0.17 mmol, 1.0 equiv.), 4-(*S*-methylsulfonimidoyl)morpholine **2a** (32.8 mg, 0.20 mmol, 1.2 equiv.), $Pd(OAc)_2$ (1.9 mg, 0.008 mmol, 5.0 mol%), RuPhos (5.8 mg, 0.0125 mmol, 7.5 mol%), Cs_2CO_3 (97.7 mg, 0.30 mmol, 1.8 equiv.) and anhydrous toluene (1 mL, 0.17 M), followed by second addition of Cs_2CO_3 (217.2 mg, 0.67 mmol, 4.0 equiv.) and stirring for 5 h. Purification by flash column chromatography (SiO_2, pentane/ethyl acetate, 5:1 to 2:1) afforded **3m** as a white solid (32.9 mg, 61%).

m.p = 86 – 88 °C.

^{1}H NMR (400 MHz, CDCl$_3$): δ 7.49 (m, 3H), 7.40 (m, 3H), 7.36 – 7.31 (m, 1H), 7.30 – 7.25 (m, 1H), 6.91 (ddd, *J* = 8.2, 6.9, 1.4 Hz, 1H), 6.14 (s, 1H), 3.84 – 3.70 (m, 4H), 3.14 (t, *J* = 4.8 Hz, 4H).

^{13}C NMR (101 MHz, CDCl$_3$): δ 155.4, 147.2, 137.4, 131.9, 129.2, 128.9, 128.8, 128.1, 124.5, 120.1, 116.8, 104.3, 66.6, 45.9.

IR: ν_{max} (neat, cm^{-1}) = 3038, 2925, 1531, 1344, 1259, 1115, 944, 755, 638.

HRMS (ESI +, m/z) Calcd $C_{18}H_{19}N_2O_2S^+$ $[M+H]^+$ 327.1162 found 327.1166.

2-(1,4-dioxa-8-azaspiro[4.5]decan-8-yl)benzo[*c*][1,2]thiazine 2-oxide (3o)

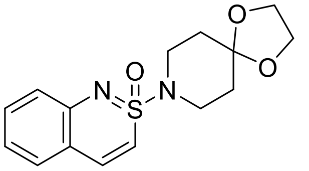

Prepared according to **general procedure B** using 2-bromobenzaldehyde **1a** (20 µL, 0.17 mmol, 1.0 equiv.), 8-(*S*-methylsulfonimidoyl)-1,4-dioxa-8-azaspiro[4.5]decane **2o** (44.0 mg, 0.20 mmol, 1.2 equiv.), $Pd(OAc)_2$ (1.9 mg, 0.008 mmol, 5.0 mol%), RuPhos (5.8 mg, 0.0125 mmol, 7.5 mol%), Cs_2CO_3 (97.7 mg, 0.30 mmol, 1.8 equiv.) and anhydrous toluene (1 mL, 0.17 M),

followed by second addition of Cs_2CO_3 (217.2 mg, 0.67 mmol, 4.0 equiv.) and stirring for 72 h. Purification by flash column chromatography (SiO_2, pentane/ethyl acetate, 3:1 to 1:1) afforded **3o** as yellow gum (26.5 mg, 52%).

^{1}H NMR (400 MHz, $CDCl_3$): δ 7.66 (d, *J* = 10.0 Hz, 1H), 7.38 (ddd, *J* = 8.5, 7.1, 1.6 Hz, 1H), 7.30 (dd, *J* = 8.0, 1.6 Hz, 1H), 7.23 (dd, *J* = 8.5, 1.2 Hz, 1H), 6.97 (ddd, *J* = 8.0, 7.1, 1.2 Hz, 1H), 6.19 (d, *J* = 10.0 Hz, 1H), 3.94 (s, 4H), 3.30 – 3.14 (m, 4H), 1.85 – 1.69 (m, 4H).

^{13}C NMR (101 MHz, $CDCl_3$): δ 146.6, 141.7, 131.7, 129.3, 123.8, 120.1, 116.1, 107.0, 106.5, 64.6, 44.1, 34.9.

IR: ν_{max} (neat, cm^{-1}) = 3055, 1610, 1296, 1230, 1145, 1046, 940, 736.

HRMS (ESI +, m/z) Calcd for $C_{15}H_{18}N_2O_3SNa^+$ $[M+Na]^+$ 329.0930 found 329.0936.

2-(4-(pyrimidin-2-yl)piperazin-1-yl)benzo[*c*][1,2]thiazine 2-oxide (3p)

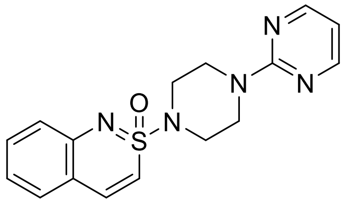

Prepared according to **general procedure B** using 2-bromobenzaldehyde **1a** (20 µL, 0.17 mmol, 1.0 equiv.), 2-(4-(*S*-methylsulfonimidoyl)piperazin-1-yl)pyrimidine **2p** (48.2 mg, 0.20 mmol, 1.2 equiv), $Pd(OAc)_2$ (1.9 mg, 0.008 mmol, 5.0 mol%), RuPhos (5.8 mg, 0.0125 mmol, 7.5 mol%), Cs_2CO_3 (97.7 mg, 0.30 mmol, 1.8 equiv.) and anhydrous toluene (1 mL, 0.17 M), followed by second addition of Cs_2CO_3 (217.2 mg, 0.67 mmol, 4.0 equiv.) and stirring for 24 h. Purification by flash column chromatography (SiO_2, pentane/ethyl acetate, 3:1 to 1:1) afforded **3p** as a white solid (40.7 mg, 75%).

m.p = 172 – 174 °C.

^{1}H NMR (400 MHz, $CDCl_3$): δ 8.30 (d, *J* = 4.8 Hz, 2H), 7.72 (d, *J* = 10.0 Hz, 1H), 7.41 (ddd, *J* = 8.6, 7.1, 1.6 Hz, 1H), 7.33 (dd, *J* = 8.0, 1.6 Hz, 1H), 7.26 (dd, *J* = 8.6, 1.2 Hz, 1H)7.00 (ddd, *J* = 8.0, 7.2, 1.2 Hz, 1H), 6.53 (t, *J* = 4.8 Hz, 1H), 6.20 (d, *J* = 10.0 Hz, 1H), 4.01 – 3.85 (m, 4H), 3.15 (t, *J* = 5.1 Hz, 4H).

^{13}C NMR (101 MHz, CDCl$_3$): δ 161.5, 158.0, 146.6, 142.4, 131.9, 129.5, 123.9, 120.3, 116.2, 110.7, 106.3, 45.6, 43.7.

IR: ν_{max} (neat, cm^{-1}) = 3055, 1589, 1484, 1359, 1268, 957.

HRMS (ESI +, m/z) Calcd for $C_{16}H_{18}N_5OS^+$ [M+H]$^+$ 328.1227 found 328.1228.

2-(6,7-dihydrothieno[3,2-c]pyridin-5(4H)-yl)benzo[*c*][1,2]thiazine 2-oxide (3q)

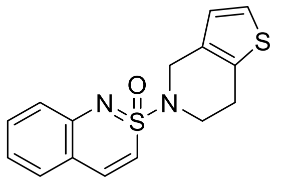

Prepared according to **general procedure B** using 2-bromobenzaldehyde **1a** (20 µL, 0.17 mmol, 1.0 equiv.), 5-(*S*-methylsulfonimidoyl)-4,5,6,7-tetrahydrothieno[3,2-c]pyridine **2q** (43.2 mg, 0.20 mmol), Pd(OAc)$_2$ (1.9 mg, 5.0 mol%), RuPhos (5.8 mg, 7.5 mol%), Cs_2CO_3 (97.7 mg, 0.30 mmol) and anhydrous toluene (1 mL), followed by second addition of Cs_2CO_3 (217.2 mg, 0.67 mmol) and stirring for 18 h. Purification by flash column chromatography (SiO_2, pentane/ethyl acetate, 5:1 to 3:1) afforded **3q** as a white solid (25.1 mg, 50%).

m.p = 92 – 94 °C.

^{1}H NMR (400 MHz, CDCl$_3$): δ 7.67 (d, *J* = 10.0 Hz, 1H), 7.42 (ddd, *J* = 8.6, 7.1, 1.6 Hz, 1H), 7.33 (dd, *J* = 8.0, 1.6 Hz, 1H), 7.30 – 7.22 (m, 1H), 7.12 (d, *J* = 5.0 Hz, 1H), 7.01 (ddd, *J* = 8.0, 7.1, 1.2 Hz, 1H), 6.68 (d, *J* = 5.0 Hz, 1H), 6.15 (d, *J* = 10.0 Hz, 1H), 4.34 (dt, *J* = 16.0, 2.0 Hz, 1H), 4.10 (dt, *J* = 16.0, 2.0 Hz, 1H), 3.62 (dt, *J* = 13.0, 6.0 Hz, 1H), 3.46 (dt, *J* = 13.0, 6.0 Hz, 1H), 2.92 (td, *J* = 6.0, 2.0 Hz, 2H).

^{13}C NMR (101 MHz, CDCl$_3$): δ 146.5, 141.7, 133.1, 131.7, 131.4, 129.3, 124.9, 123.9, 123.7, 120.2, 116.0, 107.8, 45.7, 43.9, 25.5.

IR: ν_{max} (neat, cm^{-1}) = 2940, 1609, 1323, 1236, 990, 917, 752.

HRMS (ESI +, m/z) Calcd for $C_{15}H_{14}N_2OS_2Na^+$ [M+Na]$^+$ 325.0440 found 325.0438.

2-((3,4-dimethoxybenzyl)(methyl)amino)-2l4-benzo[*c*][1,2]thiazine 2-oxide (3r)

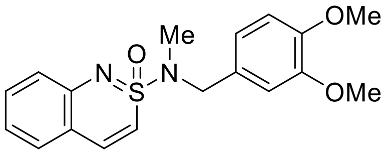

Prepared according to **general procedure B** using 2-bromobenzaldehyde **1a** (20 µL, 0.17 mmol, 1.0 equiv.), *N*-(3,4-dimethoxybenzyl)-*N*-methylmethanesulfonimidamide **2r** (51.6 mg, 0.20 mmol, 1.2 equiv.), $Pd(OAc)_2$ (1.9 mg, 0.008 mmol, 5.0 mol%), RuPhos (5.8 mg, 0.0125 mmol, 7.5 mol%), Cs_2CO_3 (97.7 mg, 0.30 mmol, 1.8 equiv.) and anhydrous toluene (1 mL, 0.17 M), followed by second addition of Cs_2CO_3 (217.2 mg, 0.67 mmol, 4.0 equiv.) and stirring for 18 h. Purification by flash column chromatography (SiO_2, pentane/ethyl acetate, 3:1 to 1:1) afforded **3r** as a yellow gum (41.0 mg, 72%).

^{1}H NMR (400 MHz, CDCl$_3$): δ 7.65 (d, *J* = 9.9 Hz, 1H), 7.38 (ddd, *J* = 8.5, 7.0, 1.6 Hz, 1H), 7.30 (dd, *J* = 7.8, 1.6 Hz, 1H), 7.24 (dd, *J* = 8.5, 1.2 Hz, 1H), 7.01 – 6.92 (m, 2H), 6.87 (dd, *J* = 8.1, 1.9 Hz, 1H), 6.82 (d, *J* = 8.1 Hz, 1H), 6.13 (d, *J* = 9.9 Hz, 1H), 4.24 (d, *J* = 14.5 Hz, 1H), 4.16 (d, *J* = 14.5 Hz, 1H), 3.91 (s, 3H), 3.87 (s, 3H), 2.56 (s, 3H).

^{13}C NMR (101 MHz, CDCl$_3$): δ 149.3, 148.9, 146.6, 141.5, 131.6, 129.3, 128.7, 123.8, 121.0, 120.1, 116.0, 111.7, 111.0, 107.6, 56.1, 56.0, 54.0, 34.1.

IR: ν_{max} (neat, cm^{-1}) = 3059, 1610, 1517, 1297, 1236, 1033, 914, 777.

HRMS (ESI +, m/z) Calcd for $C_{18}H_{21}N_2O_3S^+$ $[M+H]^+$ 345.1267 found 345.1268.

3-methyl-2-morpholinobenzo[*c*][1,2]thiazine 2-oxide (3t)

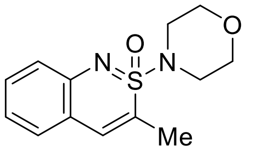

Prepared according to **general procedure B** using 2-bromobenzaldehyde **1a** (20 µL, 0.17 mmol, 1.0 equiv.), 4-(ethylsulfonimidoyl)morpholine **2t** (35.6 mg, 0.20 mmol, 1.2 equiv.), $Pd(OAc)_2$ (1.9 mg, 0.008 mmol, 5.0 mol%), RuPhos (5.8 mg, 0.0125 mmol, 7.5 mol%), Cs_2CO_3 (97.7 mg, 0.30 mmol, 1.8 equiv.) and anhydrous toluene (1 mL, 0.17 M), followed by second addition of

Cs_2CO_3 (217.2 mg, 0.67 mmol, 4.0 equiv.) and stirring for 24 h at 135 °C. Purification by flash column chromatography (SiO_2, pentane/ethyl acetate, 3:1 to 1:1) afforded **3t** as a yellow gum (25.7 mg, 58%).

^{1}H NMR (400 MHz, $CDCl_3$): δ 7.37 (d, *J* = 1.5 Hz, 1H), 7.33 (ddd, *J* = 8.6, 7.1, 1.6 Hz, 1H), 7.25 – 7.18 (m, 2H), 6.95 (ddd, *J* = 7.8, 7.1, 1.2 Hz, 1H), 3.79 – 3.65 (m, 4H), 3.19 – 3.04 (m, 4H), 2.28 (d, *J* = 1.5 Hz, 3H).

^{13}C NMR (101 MHz, $CDCl_3$): δ 145.3, 139.4, 130.4, 128.2, 122.8, 119.8, 116.9, 66.5, 46.0 ,15.5. (One signal missing)

IR: ν_{max} (neat, cm^{-1}) = 2963, 1621, 1346, 1302, 1236, 1113, 1008, 945, 729.

HRMS (ESI +, m/z) Calcd for $C_{13}H_{15}N_2O_2SNa^+$ $[M+Na]^+$ 287.0825 found 287.0831.

2-morpholino-3-phenylbenzo[*c*][1,2]thiazine 2-oxide (3u)

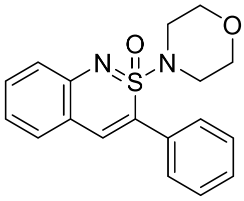

Prepared according to **general procedure B** using 2-bromobenzaldehyde **1a** (20 µL, 0.17 mmol, 1.0 equiv.), 4-(*S*-benzylsulfonimidoyl)morpholine **2u** (48.2 mg, 0.20 mmol, 1.2 equiv.), $Pd(OAc)_2$ (1.9 mg, 0.008 mmol, 5.0 mol%), RuPhos (5.8 mg, 0.0125 mmol, 7.5 mol%), Cs_2CO_3 (97.7 mg, 0.30 mmol, 1.8 equiv.) and anhydrous toluene (1 mL, 0.17 M), the reaction mixture was stirred for 18 h at 105 °C without the second addition of Cs_2CO_3. Purification by flash column chromatography (SiO_2, pentane/ethyl acetate, 6:1 to 3:1) afforded **3u** as a white solid (37.0 mg, 68%).

m.p = 110 – 112 °C.

^{1}H NMR (400 MHz, $CDCl_3$): δ 7.77 – 7.67 (m, 2H), 7.65 (s, 1H), 7.52 – 7.44 (m, 3H), 7.43 – 7.34 (m, 2H), 7.32 – 7.25 (m, 1H), 7.02 (ddd, *J* = 8.0, 7.1, 1.2 Hz, 1H), 3.50 (ddd, *J* = 5.6, 3.8, 1.6 Hz, 4H), 3.04 (dddd, *J* = 12.4, 5.5, 4.0, 1.0 Hz, 2H), 2.82 – 2.72 (m, 2H).

^{13}C NMR (101 MHz, $CDCl_3$): δ 145.8, 139.3, 133.3, 131.4, 129.7, 129.5, 129.3, 129.0, 123.3, 122.5, 120.5, 117.4, 66.6, 46.7.

IR: ν_{max} (neat, cm^{-1}) = 2965, 1610, 1451, 1389, 1302, 1229, 1112, 951, 730.

HRMS (ESI +, m/z) Calcd for $C_{18}H_{18}N_2O_2SNa^+$ $[M+Na]^+$ 349.0981 found 349.0982.

2-morpholino-3-vinylbenzo[*c*][1,2]thiazine 2-oxide (3v)

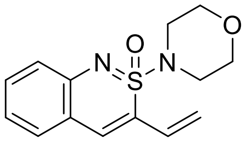

Prepared according to **general procedure B** using 2-bromobenzaldehyde **1a** (20 µL, 0.17 mmol, 1.0 equiv.), 4-(allylsulfonimidoyl)morpholine **2v** (38.0 mg, 0.20 mmol, 1.2 equiv.), $Pd(OAc)_2$ (1.9 mg, 0.008 mmol, 5.0 mol%), RuPhos (5.8 mg, 0.0125mmol, 7.5 mol%), Cs_2CO_3 (97.7 mg, 0.30 mmol, 1.8 equiv.) and anhydrous toluene (1 mL, 0.17 M), the reaction mixture was stirred for 18 h at 105 °C without the second addition of Cs_2CO_3. Purification by flash column chromatography (SiO_2, pentane/ethyl acetate, 6:1 to 3:1) afforded **3v** as a yellow gum (7.5 mg, 16%).

^{1}H NMR (400 MHz, $CDCl_3$): δ 7.60 (s, 1H), 7.37 (ddd, *J* = 8.5, 7.1, 1.6 Hz, 1H), 7.31 (dd, *J* = 7.8, 1.6 Hz, 1H), 7.22 (dd, *J* = 8.2, 1.1 Hz, 1H), 6.98 (ddd, *J* = 8.1, 7.1, 1.2 Hz, 1H), 6.56 (dd, *J* = 17.5, 11.2 Hz, 1H), 5.91 (d, *J* = 17.5 Hz, 1H), 5.47 (d, *J* = 11.3 Hz, 1H), 3.70 (qdd, *J* = 11.6, 6.1, 3.4 Hz, 4H), 3.09 (qdd, *J* = 12.3, 6.1, 3.4 Hz, 4H).

^{13}C NMR (101 MHz, $CDCl_3$): δ 146.1, 137.9, 131.6, 129.4, 128.8, 123.2, 120.5, 119.3, 119.1, 117.2, 66.7, 46.5.

IR: ν_{max} (neat, cm^{-1}) = 2921, 2859, 1608, 1351, 1305, 1237, 1114, 947, 735.

HRMS (ESI +, m/z) Calcd for $C_{14}H_{16}N_2O_2SNa^+$ $[M+Na]^+$ 299.0825 found 299.0824.

4-amino-2-morpholino-3-phenylbenzo[*c*][1,2]thiazine 2-oxide (3w)

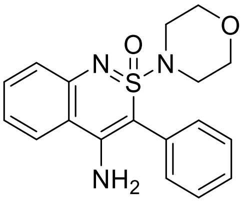

Prepared according to **general procedure B** using 2-bromobenzonitrile (30.3 mg, 0.17 mmol, 1.0 equiv.), 4-(S-benzylsulfonimidoyl)morpholine **2u** (48.2 mg, 0.20 mmol, 1.2 equiv.), $Pd(OAc)_2$ (1.9 mg, 0.008 mmol, 5.0 mol%), RuPhos (5.8 mg, 0.0125 mmol, 7.5 mol%), Cs_2CO_3 (97.7 mg, 0.30 mmol, 1.8 equiv.) and anhydrous toluene (1 mL, 0.17 M), followed by second addition of Cs_2CO_3 (217.2 mg, 0.67 mmol, 4.0 equiv.) and stirring for 24 h at 135 °C. Purification by flash column chromatography (SiO_2, pentane/ethyl acetate, 6:1 to 1:1) afforded **3w** as a white solid (39.0 mg, 69%).

m.p = 84 – 86 °C.

^{1}H NMR (400 MHz, $CDCl_3$): δ 7.62 – 7.41 (m, 5H), 7.38 (d, *J* = 7.6 Hz, 2H), 7.28 (dd, *J* = 8.3, 1.3 Hz, 1H), 7.00 (ddd, *J* = 8.3, 7.0, 1.3 Hz, 1H), 4.56 (s, 2H), 3.61 – 3.46 (m, 4H), 3.03 (ddd, *J* = 12.4, 6.2, 3.5 Hz, 2H), 2.75 – 2.65 (m, 2H).

^{13}C NMR (101 MHz, $CDCl_3$): δ 149.1, 146.3, 132.5, 131.4, 129.9, 129.5, 129.3, 124.8, 121.7, 119.6, 111.4, 96.8, 66.7, 46.2.

IR: ν_{max} (neat, cm^{-1}) = 3378, 2860, 1610, 1348, 1260, 1113, 941, 715.

HRMS (ESI +, m/z) Calcd $C_{18}H_{20}N_3O_2S^+$ $[M+H]^+$ 342.1271 found 342.1277.

3.4 Preparation of N-aryl sulfonimidamide

2-((methyl(morpholino)(oxo)-λ[6]-sulfaneylidene)amino)benzaldehyde (Int-1)

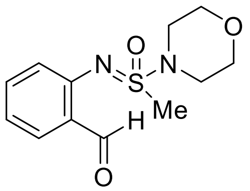

Prepared according to **general procedure B** with the following modification: second addition of Cs_2CO_3 not performed, using 2-bromobenzaldehyde **1a** (20 µL, 0.17 mmol, 1.0 equiv.), 4-(*S*-methylsulfonimidoyl)morpholine **2a** (32.8 mg, 0.20 mmol, 1,2 equiv.), $Pd(OAc)_2$ (1.9 mg, 0.008 mmol, 5.0 mol%), RuPhos (5.8 mg, 0.0125 mmol, 7.5 mol%), Cs_2CO_3 (97.7 mg, 0.30 mmol, 1.8 equiv.) and anhydrous toluene (1 mL, 0.17 M). Purification by flash column chromatography (SiO_2, pentane/ethyl acetate, 3:1 to 1:2) afforded **Int-1** as yellow solid (44.2 mg, 99%).

m.p = 98 – 100 °C.

^{1}H NMR (400 MHz, $CDCl_3$): δ 10.59 (s, 1H), 7.81 – 7.73 (m, 1H), 7.44 – 7.33 (m, 2H), 7.07 – 6.95 (m, 1H), 3.77 – 3.61 (m, 4H), 3.35 – 3.11 (m, 4H), 3.03 (s, 3H).

^{13}C NMR (101 MHz, $CDCl_3$): δ 192.3, 147.4, 135.0, 129.0, 128.1, 123.0, 122.1, 66.5, 46.7, 37.0.

IR: ν_{max} (neat, cm^{-1}) = 2926, 1683, 1595, 1475, 1286, 1260, 1113, 935, 790.

HRMS (ESI +, m/z) Calcd $C_{12}H_{16}N_2O_3SNa$ $[M+Na]^+$ 291.0774 found 291.0772.

2-((methyl(morpholino)(oxo)- λ[6]-sulfaneylidene)amino)benzonitrile (SI-5)

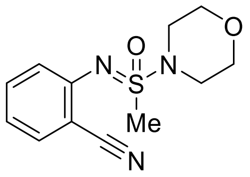

Prepared according to **general procedure B** using 2-bromo-benzonitrile (30.3 mg, 0.17 mmol, 1.0 equiv.), 4-(*S*-methylsulfonimidoyl)morpholine **2a** (32.8 mg, 0.20 mmol), $Pd(OAc)_2$ (1.9 mg, 0.008 mmol, 5.0 mol%), RuPhos (5.8 mg, 0.0125 mmol, 7.5 mol%), Cs_2CO_3 (97.7 mg, 0.30 mmol, 1.8 equiv.) and anhydrous toluene (1 mL, 0.17 M). Purification by flash column

chromatography (SiO_2, pentane/ethyl acetate, 3:1 to 1:1) afforded **SI-5** as orange oil (30.7 mg, 69%).

^{1}H NMR (400 MHz, $CDCl_3$): δ 7.54 – 7.47 (m, 1H), 7.44 – 7.33 (m, 2H), 6.98 (ddd, *J* = 7.7, 6.3, 2.2 Hz, 1H), 3.76 – 3.60 (m, 4H), 3.35 – 3.20 (m, 4H), 3.05 (s, 3H).

^{13}C NMR (101 MHz, $CDCl_3$): δ 147.7, 133.4, 133.4, 122.7, 121.9, 118.4, 107.7, 66.5, 46.8, 36.8.

IR: ν_{max} (neat, cm^{-1}) = 2926, 2224, 1593, 1483, 1310, 1231, 1113, 1075, 935, 785.

HRMS (ESI +, m/z) Calcd $C_{12}H_{15}N_3O_2SNa$ $[M+Na]^+$ 288.0777 found 288.0779.

3.5 Preparation of cyclic sulfonimidamides (primary amine)

tert-butyl(imino)(methyl)- λ⁶-sulfanone (4)

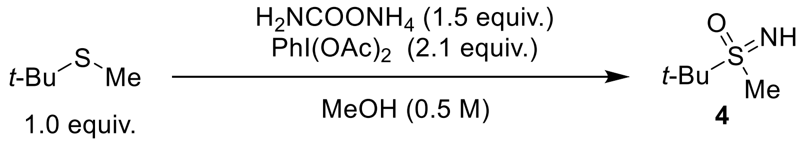

This procedure was adopted from the literature[3], to a flask containing a stirrer bar was added successively, *t*-Butyl methyl sulfide (1.9 mL, 15 mmol, 1.0 equiv.), ammonium carbamate (1.76 g, 22.5 mmol, 1.5 equiv.) and then MeOH (30 mL, 0.5 M). PIDA (10.15 g, 31.5 mmol, 2.1 equiv.) was added in one portion and the reaction was stirred for 30 min. The solvent was removed under reduced pressure and the crude product was purified by flash chromatography (SiO_2, ethyl acetate to 6% MeOH in ethyl acetate) afforded **4** as a white solid (1.53 g, 76%).

^{1}H NMR (400 MHz, $CDCl_3$): δ 2.88 (s, 3H), 2.05 (s, 1H), 1.44 (s, 9H).

^{13}C NMR (101 MHz, $CDCl_3$): δ 60.3, 36.2, 24.0.

HRMS (ESI +, m/z) Calcd for $C_5H_{13}NOSNa^+$ $[M+Na]^+$ 158.0610 found 158.0610.

Data is consistent with the literature.[3]

2-(_tert_-butyl)-2 λ⁴-benzo[_c_][1,2]thiazine 2-oxide (5)

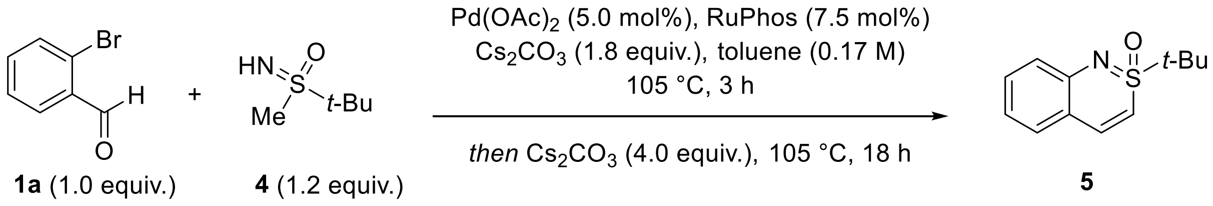

An oven-dried 100 mL round bottom flask with a stirring bar was charged with 2-bromo benzaldehyde **1a** (0.49 mL, 4.2 mmol, 1.0 equiv.), *t*-Butyl methyl sulfoximine **4** (676 mg, 5.0 mmol, 1.2 equiv.), $Pd(OAc)_2$ (46.8 mg, 0.21 mmol, 5.0 mol%), RuPhos (146 mg, 0.31 mmol, 7.5 mol%) and Cs_2CO_3 (2.44g, 7.5 mmol, 1.8 equiv.). The flask was evacuated and back-filled with nitrogen gas three times before addition of degassed, anhydrous toluene (25 ml, 0.167 M).

The mixture was heated at 105 °C for 3 hours before being cooled to room temperature. Cs_2CO_3 (5.43 g, 16.7 mmol, 4.0 equiv.) was then added and flask was sealed again. The reaction mixture was continued to stir at 105 °C for 18 hours. Once completed (judged by TLC), the reaction mixture was cooled to room temperature and ethyl acetate was added. After filtration through filter paper and removal of solvent *in vacuo*, the resultant crude mixture was purified by column chromatography (SiO_2, pentane/ethyl acetate, 6:1 to 3:1) afforded **5** as a yellow solid (709 mg, 77%).

m.p = 114 – 116 °C.

^{1}H NMR (400 MHz, $CDCl_3$): δ 7.69 (d, *J* = 10.0 Hz, 1H), 7.38 (ddd, *J* = 8.5, 7.1, 1.6 Hz, 1H), 7.30 – 7.19 (m, 2H), 6.92 (ddd, *J* = 8.0, 7.1, 1.2 Hz, 1H), 6.36 (d, *J* = 10.0 Hz, 1H), 1.46 (s, 9H).

^{13}C NMR (101 MHz, $CDCl_3$): δ 146.4, 141.9, 132.1, 129.9, 124.0, 119.4, 116.5, 101.9, 62.1, 23.2.

IR: ν_{max} (neat, cm^{-1}) = 3062, 1611, 1342, 1294, 1212, 995, 728.

HRMS (ESI +, m/z) Calcd for $C_{12}H_{15}NOSNa^+$ $[M+Na]^+$ 244.0767 found 244.0753.

1*H*-benzo[*c*][1,2]thiazine 2-oxide (6)

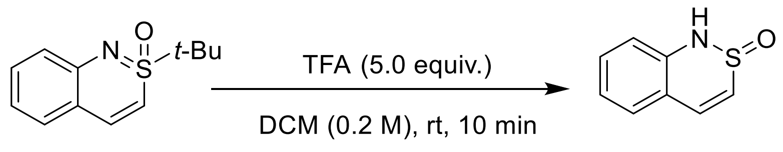

5 (1.0 equiv.) **6**

To a solution of compound **5** (442 mg, 2.0 mmol, 1.0 equiv.) in DCM (10 mL, 0.2 M) was added TFA (0.77 mL, 10 mmol, 5.0 equiv.) at room temperature. After stirring for 10 min, the reaction mixture was quenched with sat. aq. $NaHCO_3$. The mixture was extracted with EtOAc three times. The combined organic layers were washed with brine, dried over Na_2SO_4 and then concentrated. The crude mixture was purified by column chromatography on silica gel (SiO_2, pentane/ethyl acetate, 1:1 to 1:3) afforded product **6** as a white solid (258 mg, 78%).

m.p = 150 – 152 °C.

^{1}H NMR (400 MHz, $CDCl_3$): δ 7.84 (s, 1H), 7.44 (dd, *J* = 7.8, 1.5 Hz, 1H), 7.39 – 7.33 (m, 2H, Ar-*H*), 7.13 (td, *J* = 7.5, 1.1 Hz, 1H), 7.02 (d, *J* = 8.1 Hz, 1H), 6.92 (dd, *J* = 9.8, 2.5 Hz, 1H).

^{13}C NMR (101 MHz, CDCl$_3$): δ 134.3, 131.2, 129.8, 129.6, 123.0, 122.7, 119.6, 118.3.

IR: ν_{max} (neat, cm^{-1}) = 3119, 2851, 1613, 1456, 1036, 916, 746.

HRMS (ESI +, m/z) Calcd for $C_8H_7NOSK^+$ [M+K]$^+$ 203.9880 found 203.9871.

1H-benzo[*c*][1,2]thiazine 2,2-dioxide (7)

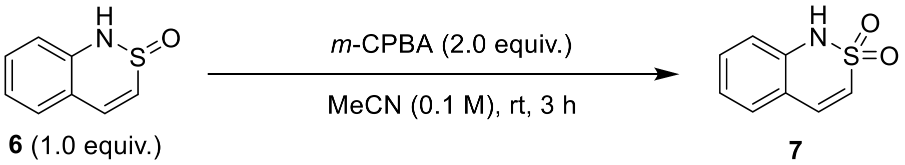

Cyclised sulfinamide **6** (16.5 mg, 0.1 mmol, 1.0 equiv.) was weighed into an oven-dried 10 mL reaction vial, sealed, evacuated and back-filled with N_2 three times. Dry acetonitrile (1 mL, 0.1 M) was then added. *m*-CPBA (34.5 mg, 0.2 mmol, 2.0 equiv.) was added under a cone of nitrogen, the vial re-sealed and the reaction was stirred at room temperature for 3 h. Once completed (determined by TLC), the reaction mixture was then diluted with EtOAc and quenched with saturated aqueous solution of sodium thiosulfate, The aqueous phase was extracted with EtOAc for three times. The combined organic layers were then dried over anhydrous Na_2SO_4, filtered and concentrated *in vacuo*. The resulting reaction mixture was purified by column chromatography (pentane/ethyl acetate, 4:1) to give the desired product **7** as a white solid (13.5 mg, 75%).

m.p = 134 – 136 °C.

^{1}H NMR (400 MHz, CDCl$_3$): δ 7.61 (s, 1H), 7.40 (ddd, *J* = 9.2, 7.6, 1.9 Hz, 2H), 7.27 (d, *J* = 10.4 Hz, 1H), 7.16 (td, *J* = 7.6, 1.1 Hz, 1H), 7.02 (d, *J* = 8.0 Hz, 1H), 6.76 (dd, *J* = 10.5, 2.4 Hz, 1H).

^{13}C NMR (101 MHz, CDCl$_3$): δ 137.4, 135.5, 131.5, 129.9, 123.7, 122.5, 119.4, 118.0.

IR: ν_{max} (neat, cm^{-1}) = 3187, 1613, 1458, 1298, 1150, 936, 769.

HRMS (ESI +, m/z) Calcd for $C_8H_7NO_2SK^+$ [M+K]$^+$ 219.9829 found 219.9831.

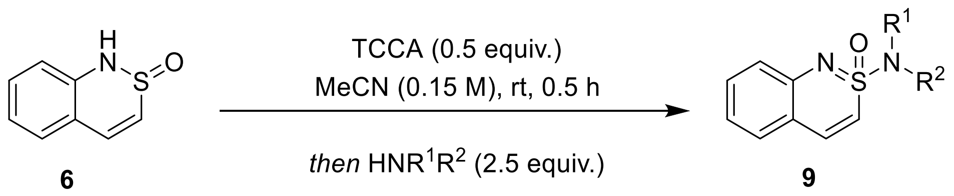

General Procedure C: Cyclised sulfinamide **6** (1.0 equiv.) was weighed into an oven-dried 10 mL reaction vial, sealed, evacuated and back-filled with N_2 three times. Dry acetonitrile (0.15 M) was then added. Trichloroisocyanuric acid (0.5 equiv.) was added under a cone of nitrogen, the vial re-sealed and the reaction was stirred at room temperature for 30 mins. Amine (2.5 equiv.) was then added and the reaction was stirred at room temperature. After completion of the reaction (determined by TLC), the reaction mixture was concentrated in vacuo, and the crude reaction mixture was purified by column chromatography to give the desired product **9**.

2-(butylamino)-2λ^4-benzo[*c*][1,2]thiazine 2-oxide (9a)

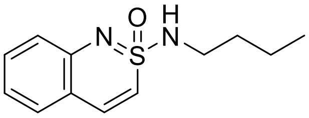

Prepared according to **general procedure C** using cyclised sulfinamide **6** (24.8 mg, 0.15 mmol, 1.0 equiv.), trichloroisocyanuric acid (17.4 mg, 0.075 mmol, 0.5 equiv.), MeCN (1 mL, 0.15 M) and n-butyl amine (40 µL, 0.38 mmol, 2.5 equiv.). After stirring for 1 hour, the crude mixture was purified by column chromatography (SiO_2, pentane/ethyl acetate, 3:1) to afford **9a** as a brown solid (27.8 mg, 78%).

m.p = 68 – 70 °C.

^{1}H NMR (400 MHz, $CDCl_3$): δ 7.65 (d, *J* = 9.9 Hz, 1H), 7.40 (ddd, *J* = 8.5, 7.1, 1.6 Hz, 1H), 7.32 (dd, *J* = 7.8, 1.6 Hz, 1H), 7.29 – 7.22 (m, 1H), 6.99 (ddd, *J* = 8.0, 7.1, 1.2 Hz, 1H), 6.30 (d, *J* = 9.9 Hz, 1H), 4.84 (t, *J* = 6.0 Hz, 1H), 2.92 – 2.79 (m, 1H), 2.72 (dtd, *J* = 12.9, 7.1, 6.0 Hz, 1H,), 1.57 – 1.41 (m, 2H), 1.40 – 1.23 (m, 2H), 0.86 (t, *J* = 7.3 Hz, 3H).

^{13}C NMR (101 MHz, $CDCl_3$): δ 146.2, 141,0, 131.5, 129.2, 123.9, 120.1, 116.0, 109.5, 43.1, 31.8, 19.9, 13.7.

IR: ν_{max} (neat, cm^{-1}) = 3263, 2959, 1611, 1345, 1296, 1213, 777.

HRMS (ESI +, m/z) Calcd for $C_{12}H_{16}N_2OSK^+$ $[M+K]^+$ 275.0615 found 275.0624.

2-(benzylamino)-2λ⁴-benzo[*c*][1,2]thiazine 2-oxide (9b)

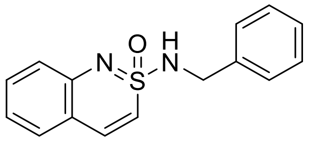

Prepared according to **general procedure C** using cyclised sulfinamide **6** (24.8 mg, 0.15 mmol, 1.0 equiv.), trichloroisocyanuric acid (17.4 mg, 0.075 mmol, 0.5 equiv.), MeCN (1 mL, 0.15 M) and benzylamine (40 µL, 0.38 mmol, 2.5 equiv.). After stirring for 1 hour, the crude mixture was purified by column chromatography (SiO_2, pentane/ethyl acetate, 4:1 to 2:1) to afford **9b** as a white solid (30.3 mg, 75%).

m.p = 100 – 102 °C.

^{1}H NMR (400 MHz, $CDCl_3$): δ 7.59 (d, *J* = 9.9 Hz, 1H), 7.41 (ddd, *J* = 8.5, 7.1, 1.6 Hz, 1H), 7.34 – 7.23 (m, 7H), 7.01 (ddd, *J* = 8.1, 7.1, 1.2 Hz, 1H), 6.25 (d, *J* = 9.9 Hz, 1H), 5.21 (t, *J* = 6.0 Hz, 1H), 4.10 (dd, *J* = 14.4, 6.0 Hz, 1H), 3.91 (dd, *J* = 14.3, 5.8 Hz, 1H).

^{13}C NMR (101 MHz, $CDCl_3$): δ 146.1, 141.0, 136.8, 131.6, 129.3, 128.8, 128.1, 128.0, 123.9, 120.3, 116.1, 109.7, 47.4.

IR: ν_{max} (neat, cm^{-1}) = 3275, 1611, 1345, 1296, 1209, 1038, 776, 701.

HRMS (ESI +, m/z) Calcd for $C_{15}H_{14}N_2OSK^+$ $[M+K]^+$ 309.0458 found 309.0451.

2-(((S)-1-phenylethyl)amino)-2 λ⁴-benzo[*c*][1,2]thiazine 2-oxide

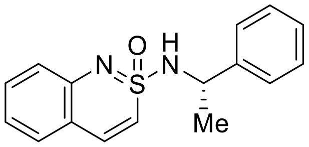

Prepared according to **general procedure C** using cyclised sulfinamide **6** (74.3 mg, 0.45 mmol, 1.0 equiv.), trichloroisocyanuric acid (52.3 mg, 0.23 mmol, 0.5 equiv.), MeCN (3 mL, 0.15 M) and (*S*)-1-phenylethan-1-amine (0.15 mL, 1.13 mmol, 2.5 equiv.). After stirring for 2 hours, the crude mixture was purified by column chromatography (SiO_2, 1% ethyl acetate in DCM) to afford **9c** as a white solid (54.4 mg, 43%) and **9c'** as a white solid (54.3 mg, 43%)

(*S*)-2-(((*S*)-1-phenylethyl)amino)-2λ^4-benzo[*c*][1,2]thiazine 2-oxide (9c)

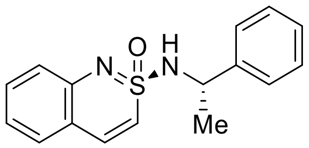

m.p = 102 – 104 °C.

^{1}H NMR (400 MHz, CDCl$_3$): δ 7.51 (d, *J* = 10.0 Hz, 1H), 7.40 – 7.33 (m, 5H), 7.33 – 7.27 (m, 2H), 7.18 (dd, *J* = 8.3, 1.2 Hz, 1H), 6.99 (ddd, *J* = 8.1, 7.1, 1.2 Hz, 1H), 6.10 (d, *J* = 9.9 Hz, 1H), 4.98 (d, *J* = 7.3 Hz, 1H), 4.42 (p, *J* = 7.0 Hz, 1H), 1.46 (d, *J* = 6.9 Hz, 3H).

^{13}C NMR (101 MHz, CDCl$_3$): δ 145.7, 142.7, 139.6, 131.5, 129.2, 128.9, 128.0, 126.6, 123.9, 120.3, 116.1, 111.4, 54.1, 22.9.

IR: ν_{max} (neat, cm^{-1}) = 3239, 1614, 1350, 1303, 1038, 775.

HRMS (ESI +, m/z) Calcd for $C_{16}H_{16}N_2OSK^+$ $[M+K]^+$ 323.0615 found 323.0615.

$[\alpha]_D^{25}$**:** +41.8° (c=1.0, $CHCl_3$).

ee was determined by HPLC using a Chiralpak IC column (n-hexane/i-PrOH, 85:15, flow rate: 0.8 mL/min.)

T_{major} = 32.61 min, T_{minor} = 35.42 min (99% ee).

(*R*)-2-(((*S*)-1-phenylethyl)amino)-2λ^4-benzo[*c*][1,2]thiazine 2-oxide (9c')

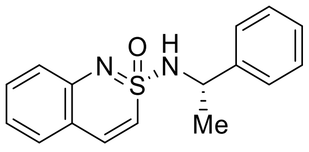

m.p = 100 – 102 °C.

^{1}H NMR (400 MHz, $CDCl_3$): δ 7.38 (ddd, *J* = 8.6, 7.1, 1.6 Hz, 1H), 7.29 – 7.25 (m, 1H), 7.23 (d, *J* = 6.9 Hz, 1H), 7.20 – 7.13 (m, 4H), 7.02 (qd, *J* = 3.7, 1.4 Hz, 2H), 6.95 (ddd, *J* = 8.2, 7.1, 1.2 Hz, 1H), 5.88 (d, *J* = 9.9 Hz, 1H), 5.16 (d, *J* = 6.5 Hz, 1H), 4.13 (p, *J* = 6.7 Hz, 1H), 1.50 (d, *J* = 6.9 Hz, 3H).

^{13}C NMR (101 MHz, $CDCl_3$): δ 146.1, 142.6, 140.1, 131.5, 129.2, 128.6 (2C), 127.8, 126.5 (2C), 123.9, 120.2, 116.0, 110.2, 54.1, 24.0.

IR: ν_{max} (neat, cm^{-1}) = 3255, 1611, 1345, 1296, 1038, 775.

HRMS (ESI +, m/z) Calcd for $C_{16}H_{16}N_2OSK^+$ $[M+K]^+$ 323.0615 found 323.0616.

$[\alpha]_D^{25}$: -292° (c=1.0, $CHCl_3$).

ee was determined by HPLC using a Chiralpak IC column (n-hexane/i-PrOH, 85:15, flow rate: 0.8 mL/min.)

T_{major} = 32.75 min, T_{minor} = 35.36 min (99% ee).

2-(tert-butylamino)-2 λ^4-benzo[*c*][1,2]thiazine 2-oxide (9d)

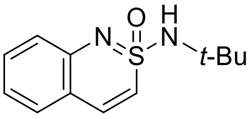

Prepared according to **general procedure C** using cyclised sulfinamide **6** (24.8 mg, 0.15 mmol, 1.0 equiv.), trichloroisocyanuric acid (17.4 mg, 0.075 mmol, 0.5 equiv.), MeCN (1 mL, 0.15 M) and *t*-butylamine (40 µL, 0.38 mmol, 2.5 equiv.). After stirring for 2.5 hours, the crude mixture was purified by column chromatography (SiO_2, pentane/ethyl acetate, 6:1 to 2:1) to afford **9d** as a white solid (23.8 mg, 67%).

m.p = 166 – 168 °C.

^{1}H NMR (400 MHz, $CDCl_3$): δ 7.51 (d, *J* = 9.9 Hz, 1H), 7.39 (ddd, *J* = 8.4, 7.1, 1.6 Hz, 1H), 7.31 (dd, *J* = 7.8, 1.6 Hz, 1H), 7.23 (d, *J* = 8.3 Hz, 1H), 6.98 (t, *J* = 7.5 Hz, 1H), 6.39 (d, *J* = 9.9 Hz, 1H), 4.79 (brs, 1H), 1.23 (s, 9H).

^{13}C NMR (101 MHz, CDCl$_3$): δ 145.1, 138.6, 131.4, 129.1, 123.9, 120.1, 116.1, 113.1, 55.4, 30.4.

IR: ν_{max} (neat, cm^{-1}) = 3262, 1610, 1367, 1296, 1177, 1022, 757.

HRMS (ESI +, m/z) Calcd for $C_{12}H_{17}N_2OS^+$ [M+H]$^+$ 237.1056 found 237.1055.

2-(phenylamino)-2λ^4-benzo[*c*][1,2]thiazine 2-oxide (9e)

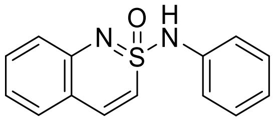

Prepared according to **general procedure C** using cyclised sulfinamide **6** (24.8 mg, 0.15 mmol, 1.0 equiv.), trichloroisocyanuric acid (17.4 mg, 0.075 mmol, 0.5 equiv.), MeCN (1 mL, 0.15 M) and aniline (35 µL, 0.38 mmol, 2.5 equiv.). After stirring for 2 hours, the crude mixture was purified by column chromatography (SiO_2, pentane/ethyl acetate, 5:1) to afford **9e** as a yellow solid (28.8 mg, 75%).

m.p = 108 – 110 °C.

^{1}H NMR (400 MHz, CDCl$_3$): δ 7.72 (d, *J* = 9.9 Hz, 1H), 7.44 (ddd, *J* = 8.5, 7.1, 1.6 Hz, 1H), 7.40 (s, 1H), 7.36 (dd, *J* = 7.8, 1.6 Hz, 1H), 7.29 (d, *J* = 8.3 Hz, 1H), 7.17 – 7.09 (m, 2H), 7.08 – 6.98 (m, 2H), 6.75 – 6.65 (m, 2H), 6.44 (d, *J* = 9.9 Hz, 1H).

^{13}C NMR (101 MHz, CDCl$_3$): δ 145.7, 142.4, 137.4, 132.1, 129.5, 129.4, 124.7, 124.2, 120.8, 120.2, 116.2, 109.5.

IR: ν_{max} (neat, cm^{-1}) = 3233, 1611, 1346, 1296, 1209, 1038, 753.

HRMS (ESI +, m/z) Calcd for $C_{14}H_{13}N_2OS^+$ [M+H]$^+$ 257.0743 found 257.0747.

2-amino-2λ^4-benzo[*c*][1,2]thiazine 2-oxide (9f)

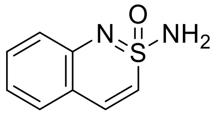

Prepared according to **general procedure C** using cyclised sulfinamide **6** (24.8 mg, 0.15 mmol, 1.0 equiv.), trichloroisocyanuric acid (17.4 mg, 0.075 mmol, 0.5 equiv.), MeCN (1 mL, 0.15 M) and ammonia solution (190 µL, 2 M in IPA, 0.38 mmol, 2.5 equiv.). After stirring for 2 hours, the crude mixture was purified by column chromatography (SiO_2, pentane/ethyl acetate, 3:1 to 1:1) to afford **9f** as a white solid (13.0 mg, 48%).

m.p = 180 – 182 °C.

^{1}H NMR (400 MHz, CD_3CN): δ 7.62 (d, *J* = 9.9 Hz, 1H), 7.43 – 7.32 (m, 2H), 7.13 – 7.06 (m, 1H), 6.97 (td, *J* = 7.4, 1.2 Hz, 1H), 6.62 (d, *J* = 9.9 Hz, 1H), 5.62 (s, 2H).

^{13}C NMR (101 MHz, CD_3CN): δ 146.3, 139.0, 132.0, 130.2, 123.9, 120.5, 117.3, 114.2.

IR: ν_{max} (neat, cm^{-1}) = 3325, 2926, 1614, 1345, 1302, 1228, 1013, 745.

HRMS (ESI +, m/z) Calcd for $C_8H_8N_2OSNa^+$ $[M+Na]^+$ 203.0250 found 203.0234.

2-morpholinobenzo[*c*][1,2]thiazine 2-oxide (3a)

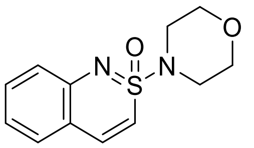

Prepared according to **general procedure C** using cyclised sulfinamide **6** (24.8 mg, 0.15 mmol, 1.0 equiv.), trichloroisocyanuric acid (17.4 mg, 0.075 mmol, 0.5 equiv.), MeCN (1 mL, 0.15 M) and morpholine (32 µL, 0.38 mmol, 2.5 equiv.). After stirring for 1 hour, the crude mixture was purified by column chromatography (SiO_2, pentane/ethyl acetate, 3:1 to 1:1) to afford **3a** as a yellow solid (22.5 mg, 60%).

3a is consistent with the previous data.

(R)-2-amino-2 λ^4-benzo[*c*][1,2]thiazine 2-oxide ((R)-9f)

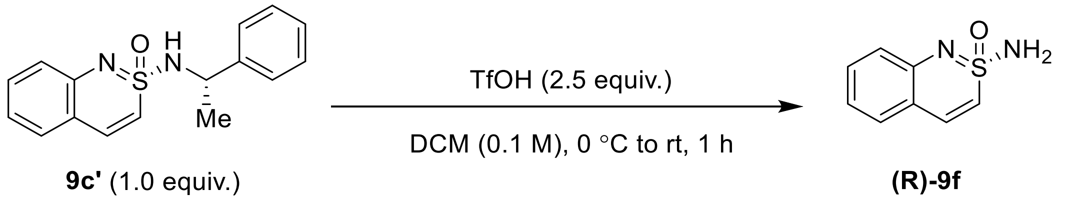

Trifluoromethanesulfonic acid (22 μL, 0.25 mmol, 2.5 equiv.) was added dropwise into the solution of **9c'** (28.4 mg, 0.1 mmol, 1.0 equiv.) in DCM (1 mL, 0.1 M) over 1 min at 0 °C. The reaction mixture was then warmed to room temperature and stirred for 1 h. Then the reaction was quenched with sat. aq. $NaHCO_3$. The mixture was extracted with DCM three times. The combined organic layers were dried over Na_2SO_4 and then concentrated. The crude mixture was purified by column chromatography on silica gel (SiO_2, pentane/ethyl acetate, 5:1 to 4:1) afforded products **(R)-9f** as a white solid (16.3 mg, 91%, 99% *ee*.).

(R)-9f Data is consistent with the previous data.

$[\alpha]_D^{25}$: +8.96° (c=1.0, $CHCl_3$)

ee was determined by HPLC using a Chiralpak IC column (n-hexane/i-PrOH, 70:30, flow rate: 0.7 mL/min.)

T_{major} = 25.02 min, T_{minor} = 50.15 min (99% ee).

3.6 Derivatisation of sulfinamide

2-fluoro-2λ4-benzo[*c*][1,2]thiazine 2-oxide (10)

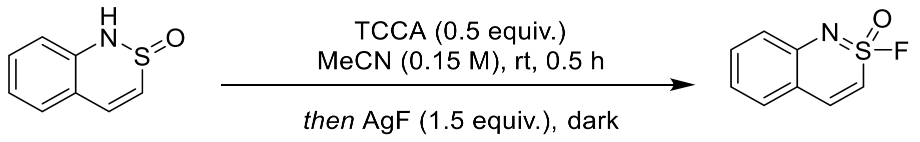

6 (1.0 equiv.) **10**

Cyclised sulfinamide **6** (24.8 mg, 0.15 mmol, 1.0 equiv.) was weighed into an oven-dried 10 mL reaction vial, sealed, evacuated and back-filled with N_2 three times. Dry acetonitrile (1 mL, 0.15 M) was then added. Trichloroisocyanuric acid (17.4 mg, 0.075 mmol, 0.5 equiv.) was added under a cone of nitrogen, the vial re-sealed and the reaction was stirred at room temperature for 30 mins. AgF (28.5 mg, 0.225 mmol, 1.5 equiv.) was then added and the reaction was stirred at room temperature in dark fume hood. After stirring for 18 h (determined by TLC), the reaction mixture was concentrated in vacuo, and the crude reaction mixture was purified by column chromatography (pentane/ethyl acetate, 6:1) to give the desired product **10** as a white solid (16.8 mg, 61%).

m.p = 94 – 96 °C.

^{1}H NMR (400 MHz, $CDCl_3$): δ 7.98 (dd, *J* = 10.0, 6.5 Hz, 1H), 7.58 (ddd, *J* = 8.5, 7.2, 1.6 Hz, 1H), 7.51 (dd, *J* = 7.9, 1.6 Hz, 1H), 7.38 (d, *J* = 8.3 Hz, 1H), 7.26 – 7.18 (m, 1H), 6.74 (dd, *J* = 10.0, 4.7 Hz, 1H).

^{19}F NMR (565 MHz, $CDCl_3$): δ 131.28.

^{13}C NMR (101 MHz, $CDCl_3$): δ 146.8 (d, *J* = 2.3 Hz), 144.9 (d, *J* = 7.5 Hz), 133.5 (d, *J* = 2.4 Hz), 130.1 (d, *J* = 2.9 Hz), 124.7 (d, *J* = 1.9 Hz), 123.2 (d, *J* = 3.1 Hz), 117.9, 107.4 (d, *J* = 29.5 Hz).

IR: ν_{max} (neat, cm^{-1}) = 3079, 1612, 1536, 1371, 1316, 1056, 778.

HRMS (ESI +, m/z) Calcd for $C_8H_7FNOS^+$ $[M+H]^+$ 184.0227 found 184.0240.

2-phenoxy-2λ4-benzo[*c*][1,2]thiazine 2-oxide (11)

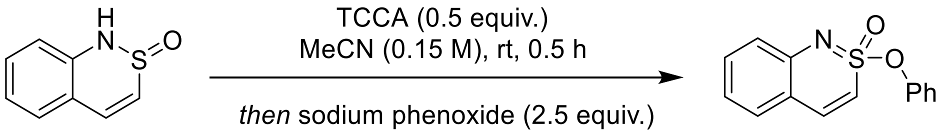

6 (1.0 equiv.) **11**

Cyclised sulfinamide **6** (24.8 mg, 0.15 mmol, 1.0 equiv.) was weighed into an oven-dried 10 mL reaction vial, sealed, evacuated and back-filled with N_2 three times. Dry acetonitrile (1 mL, 0.15 M) was then added. Trichloroisocyanuric acid (17.4 mg, 0.075 mmol, 0.5 equiv.) was added under a cone of nitrogen, the vial re-sealed and the reaction was stirred at room temperature for 30 mins. Sodium phenoxide (43.5 mg, 0.375 mmol, 2.5 equiv.) was then added and the reaction was stirred at room temperature. After stirring for 1 h (determined by TLC), the reaction mixture was concentrated in vacuo, and the crude reaction mixture was purified by column chromatography (pentane/ethyl acetate, 5:1) to give the desired product **11** as a white solid (20.0 mg, 52%).

m.p = 56 – 58 °C.

^{1}H NMR (400 MHz, $CDCl_3$): δ 7.73 (d, *J* = 10.0 Hz, 1H, SC=C*H*), 7.40 (ddd, *J* = 8.6, 7.1, 1.6 Hz, 1H, Ar-*H*), 7.25 – 7.21 (m, 2H, Ar-*H*), 7.18 (dd, *J* = 7.2, 0.9 Hz, 2H, Ar-*H*), 7.15 – 7.08 (m, 3H, Ar-*H*), 6.97 (ddd, *J* = 8.2, 7.2, 1.2 Hz, 1H, Ar-*H*), 6.53 (d, *J* = 10.0 Hz, 1H, SC*H*).

^{13}C NMR (101 MHz, $CDCl_3$): δ 149.3, 146.2, 145.1, 132.6, 129.6, 129.5 (2C), 127.2, 123.7, 123.1 (2C), 121.3, 116.9, 106.9.

IR: ν_{max} (neat, cm^{-1}) = 3067, 1613, 1367, 1305, 1022, 846, 782.

HRMS (ESI +, m/z) Calcd for $C_{14}H_{12}NO_2S^+$ $[M+H]^+$ 258.0583 found 258.0583.

3.7 Chan-Lam couping and cyclisation of sulfondiimidamide

Chan-Lam coupling on 2a

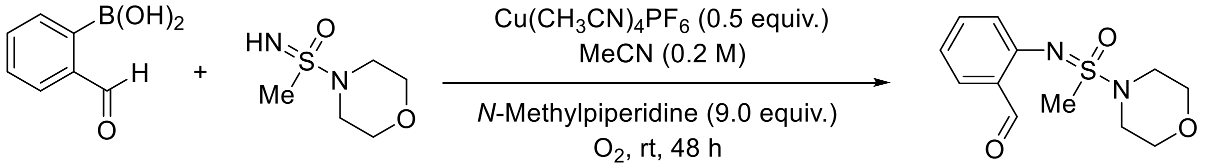

(2.5 equiv.) **2a** (1.0 equiv) **Int-1**

Sulfondimidamide **2a** (32.8 mg, 0.2 mmol, 1.0 equiv.), 2-formylphenylboronic acid (75.0 mg, 0.5 mmol, 2.5 equiv.), $Cu(CH_3CN)_4PF_6$ (37.3 mg, 0.1 mmol, 0.5 equiv.), *N*-methylpiperidine (0.22 mL, 1.8 mmol, 9.0 equiv.) were dissolved in anhydrous MeCN (1 mL, 0.2 M) in an oven-dried 10 mL microwave vial with a stirring bar and under oxygen atmosphere. The reaction was stirred at room temperature for 48 hour until completion (judged by TLC). Then the reaction mixture was quenched with sat. aq. NaCl solution. The product was extracted with ethyl acetate. The combined organic layers were dried over anhydrous Na_2SO_4 and concentrated under reduced pressure. Purification by flash column chromatography (SiO_2, pentane/ethyl acetate, 3:1 to 1:2) afforded **Int-1** as yellow solid (23.0 mg, 43%).

Int-1 data is consistent with the previous data.

Synthesis of 3a using NaO*t*Bu

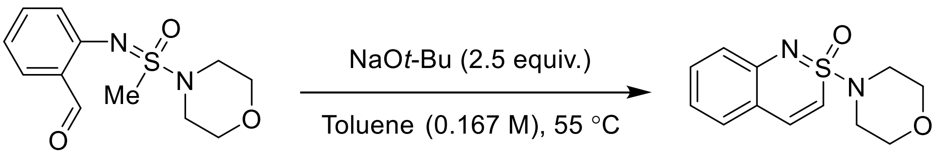

Int-1 **3a**

An oven-dried 10 mL microwave vial with a stirring bar was charged with N-aryl sulfonimidamide (26.8 mg, 0.1 mmol, 1.0 equiv.), NaO*t*-Bu (24.0 mg, 0.25 mmol, 2.5 equiv.). The vial was evacuated and back-filled with nitrogen gas three times before addition of degassed, anhydrous toluene (0.5 mL, 0.2 M). After stirring for 2 hours at 55 °C, reaction mixture was quenched with sat. aq. NaCl solution. The product was extracted with CH_2Cl_2.

The combined organic layers were dried over anhydrous Na_2SO_4 and concentrated under vacuo to afford cyclic sulfonimidamide **3a** as an orange solid (15.1 mg, 60%).

3a data is consistent with the previous data.

***N*-(((2-formylphenyl)imino)(methyl)(morpholino)-λ⁶-sulfaneylidene)-4-nitrobenzenesulfonamide (13)**

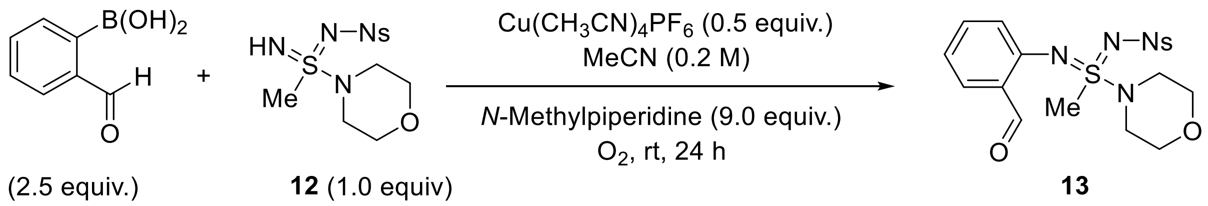

Sulfondiimidamide **12** (174 mg, 0.5 mmol, 1.0 equiv.), 2-formylphenylboronic acid (187 mg, 1.25 mmol, 2.5 equiv.), $Cu(CH_3CN)_4PF_6$ (93.2 mg, 0.25 mmol, 0.5 equiv.), *N*-methylpiperidine (0.55 mL, 4.5 mmol, 9.0 equiv.) were dissolved in anhydrous MeCN (2.5 mL, 0.2 M) in an oven-dried 10 mL microwave vial with a stirring bar and under oxygen atmosphere. The reaction was stirred at room temperature for 24 h until completion (judged by TLC). Then the reaction mixture was quenched with sat. aq. NaCl solution. The product was extracted with ethyl acetate. The combined organic layers were dried over anhydrous Na_2SO_4 and concentrated under reduced pressure. The crude product was purified by flash column chromatography (pentane/ethyl acetate, 2:1 to 1:3) to afford sulfondiimidamide **13** as a yellow solid (171 mg, 75%).

m.p = 168 – 170 °C.

^{1}H NMR (400 MHz, CDCl$_3$): δ 10.33 (s, 1H), 7.81 (s, 4H), 7.52 (dd, *J* = 7.8, 1.7 Hz, 1H), 7.08 (td, *J* = 7.7, 1.7 Hz, 1H), 6.97 (d, *J* = 8.1 Hz, 1H), 6.89 (t, *J* = 7.5 Hz, 1H), 3.90 – 3.71 (m, 4H), 3.55 (s, 3H), 3.49 (ddd, *J* = 11.7, 6.2, 3.1 Hz, 2H), 3.21 (ddd, *J* = 11.7, 6.3, 3.0 Hz, 2H).

^{13}C NMR (101 MHz, CDCl$_3$): δ 191.0, 149.5, 147.8, 144.7, 134.5, 129.1, 128.0, 127.9, 123.7, 123.1, 121.4, 66.3, 46.3, 38.9.

IR: ν_{max} (neat, cm^{-1}) = 3021, 1686, 1594, 1477, 1194, 1049, 903, 771.

HRMS (ESI +, m/z) Calcd for $C_{18}H_{20}N_4O_6S_2Na^+$ $[M+Na]^+$ 475.0717 found 475.0712.

The procedure is adapted from literature.[4]

***N*-(2-morpholino-2λ⁶-benzo[*c*][1,2]thiazin-2-ylidene)-4-nitrobenzenesulfonamide (14)**

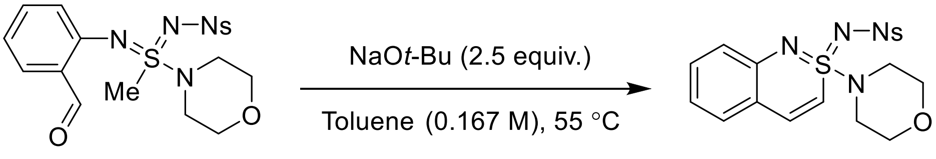

13 (1.0 equiv.) **14**

An oven-dried 10 mL microwave vial with a stirring bar was charged with sulfondiimidamide **13** (38.0 mg, 0.084 mmol, 1.0 equiv.), NaO*t*-Bu (20.2 mg, 0.21 mmol, 2.5 equiv.). The vial was evacuated and back-filled with nitrogen gas three times before addition of degassed, anhydrous toluene (0.5 mL, 0.167 M). After stirring for 2 hours at 55 °C, reaction mixture was quenched with sat. aq. NaCl solution. The product was extracted with CH_2Cl_2. The combined organic layers were dried over anhydrous Na_2SO_4 and concentrated under vacuo to afford sulfondiimidamide **14** as an orange solid (26.7 mg, 73%).

m.p = 198 – 200 °C.

^{1}H NMR (400 MHz, $CDCl_3$): δ 8.18 – 8.10 (m, 2H), 7.96 (d, *J* = 9.8 Hz, 1H), 7.88 – 7.83 (m, 2H), 7.42 (ddd, *J* = 8.6, 7.2, 1.6 Hz, 1H), 7.36 (dd, *J* = 7.9, 1.5 Hz, 1H), 7.08 (td, *J* = 7.6, 1.1 Hz, 1H), 6.91 (d, *J* = 8.3 Hz, 1H), 6.28 (d, *J* = 9.8 Hz, 1H), 3.73 (dt, *J* = 6.0, 3.5 Hz, 4H), 3.11 (dd, *J* = 6.1, 3.7 Hz, 4H).

^{13}C NMR (101 MHz, $CDCl_3$): δ 149.4, 148.7, 145.3, 145.1, 132.8, 129.9, 128.1, 123.4, 123.2, 121.6, 115.1, 103.7, 66.0, 44.7.

IR: ν_{max} (neat, cm^{-1}) = 3055, 1609, 1529, 1351, 1160, 1072, 926, 739.

HRMS (ESI +, m/z) Calcd for $C_{18}H_{18}N_4O_5S_2Na^+$ $[M+Na]^+$ 457.0611 found 457.0611.

3.8 Derivatisation of sulfonimidamides

6-bromo-2-morpholinobenzo[*c*][1,2]thiazine 2-oxide (15)

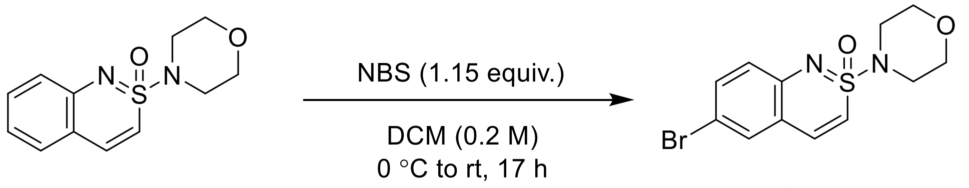

An oven-dried 10 mL microwave vial with a stirring bar was charged with compound **3a** (50.0 mg, 0.2 mmol, 1.0 equiv.) and *N*-Bromosuccinimide (40.9 mg, 0.23 mmol, 1.15 equiv). Then anhydrous DCM (1 ml, 0.2 M) was added at 0 °C. After stirring for 30 min at 0 °C, reaction mixture was warmed to room temperature and stirred for 17 h. Crude mixture was purified by flash column chromatography (SiO_2, pentane/ethyl acetate, 3:1 to 1:1) afforded **15** as a white solid (58.0 mg, 88%).

m.p = 96 – 98 °C.

^{1}H NMR (400 MHz, $CDCl_3$): δ 7.64 (d, *J* = 10.1 Hz, 1H), 7.48 – 7.42 (m, 2H), 7.16 – 7.09 (m, 1H), 6.23 (d, *J* = 10.0 Hz, 1H), 3.81 – 3.67 (m, 4H), 3.06 (t, *J* = 4.8 Hz, 4H).

^{13}C NMR (101 MHz, $CDCl_3$): δ 145.5, 141.3, 134.7, 131.3, 125.6, 117.4, 112.2, 106.9, 66.6, 45.7.

IR: ν_{max} (neat, cm^{-1}) = 3061, 1600, 1357, 1296, 1260, 1113, 941, 737, 652

HRMS (ESI +, m/z) Calcd for $C_{12}H_{13}{}^{79}BrN_2O_2SNa^+$ $[M+Na]^+$ 350.9773 found 350.9763.

Bromination of cyclic sulfonimidamide 3b

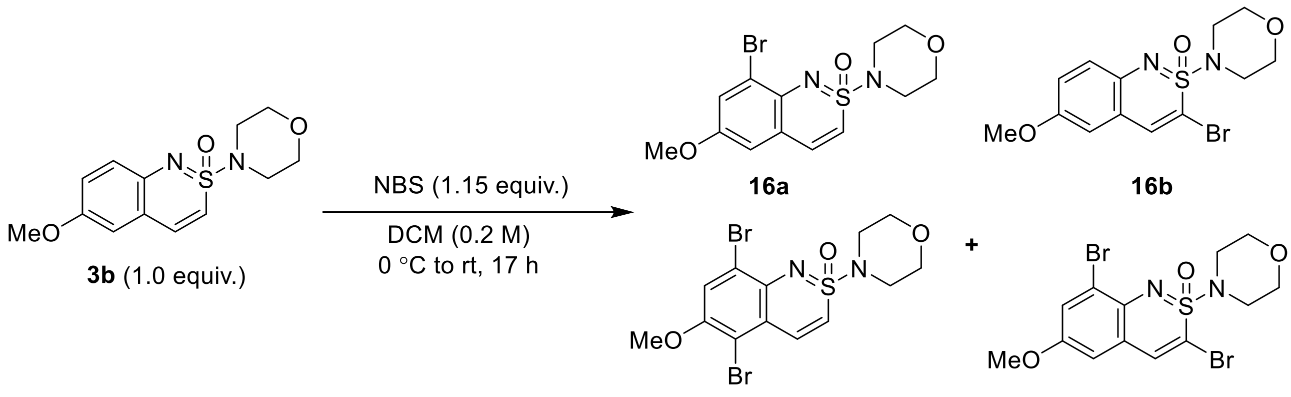

An oven-dried 10 mL microwave vial with a stirring bar was charged with compound **3b** (56.0 mg, 0.2 mmol, 1.0 equiv.) and *N*-Bromosuccinimide (40.9 mg, 0.23 mmol, 1.15 equiv.). Then anhydrous DCM (1 ml, 0.2 M) was added at 0 °C. After stirring for 30 min at 0 °C, reaction mixture was warmed to room temperature and stirred for 17 h.

8-bromo-6-methoxy-2-morpholinobenzo[*c*][1,2]thiazine 2-oxide (16a)

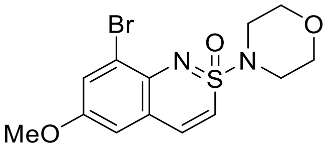

Crude mixture was purified by flash column chromatography (SiO_2, pentane/ethyl acetate, 3:1 to 1:1) afforded **16a** as a white solid (27.3 mg, 38%).

m.p = 120 – 122 °C.

^{1}H NMR (400 MHz, $CDCl_3$): δ 7.63 (d, *J* = 9.9 Hz, 1H), 7.41 (d, *J* = 2.8 Hz, 1H), 6.78 (d, *J* = 2.8 Hz, 1H), 6.22 (d, *J* = 9.9 Hz, 1H), 3.79 (s, 3H), 3.78 – 3.70 (m, 4H), 3.07 (t, *J* = 4.8 Hz, 4H).

^{13}C NMR (101 MHz, $CDCl_3$): δ 153.0, 142.0, 138.7, 124.1, 118.0, 116.8, 111.2, 107.3, 66.5, 56.1, 45.8.

IR: ν_{max} (neat, cm^{-1}) = 2997, 1582, 1273, 1158, 942, 733, 630.

HRMS (ESI +, m/z) Calcd for $C_{13}H_{16}{}^{79}BrN_2O_3S^+$ $[M+H]^+$ 359.0060 found 359.0062.

3-bromo-6-methoxy-2-morpholinobenzo[c][1,2]thiazine 2-oxide (16b)

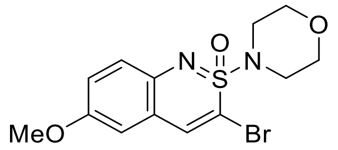

Crude mixture was purified by flash column chromatography (SiO_2, pentane/ethyl acetate, 3:1 to 2:1) afforded **16b** as a yellow solid (13.6 mg, 19%).

m.p = 118 – 120 °C.

^{1}H NMR (400 MHz, $CDCl_3$): δ 7.82 (s, 1H), 7.18 (d, *J* = 9.1 Hz, 1H), 7.06 (dd, *J* = 9.0, 2.9 Hz, 1H), 6.71 (d, *J* = 2.9 Hz, 1H), 3.84 – 3.76 (m, 5H), 3.72 (ddd, *J* = 11.6, 6.3, 3.2 Hz, 2H), 3.30 (ddd, *J* = 12.4, 6.1, 3.1 Hz, 2H), 3.22 (ddd, *J* = 12.4, 6.2, 3.3 Hz, 2H).

^{13}C NMR (101 MHz, $CDCl_3$): δ 153.8, 143.0, 139.4, 124.7, 120.9, 118.4, 109.8, 102.3, 66.8, 55.9, 47.1.

IR: ν_{max} (neat, cm^{-1}) = 2938, 1540, 1303, 1046, 959, 735, 636.

HRMS (ESI +, m/z) Calcd for $C_{13}H_{15}{}^{79}BrN_2O_3SK^+$ $[M+K]^+$ 396.9618 found 396.9608.

5,8-dibromo-6-methoxy-2-morpholinobenzo[*c*][1,2]thiazine 2-oxide (16c)

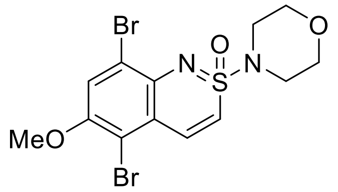

Crude mixture was purified by flash column chromatography (SiO_2, 2% ethyl acetate in DCM) afforded **16c** as a yellow solid (6.0 mg, 7%).

m.p = 142 – 144 °C.

^{1}H NMR (400 MHz, $CDCl_3$): δ 8.31 (d, *J* = 10.3 Hz, 1H), 7.45 (s, 1H), 6.29 (d, *J* = 10.3 Hz, 1H), 3.90 (s, 3H), 3.84 – 3.69 (m, 4H), 3.16 – 3.03 (m, 4H).

^{13}C NMR (101 MHz, $CDCl_3$): δ 149.8, 141.1, 139.7, 121.1, 117.2, 116.6, 111.6, 108.8, 66.6, 57.7, 45.9.

IR: ν_{max} (neat, cm^{-1}) = 3050, 1577, 1269, 1073, 938, 741, 623.

HRMS (ESI +, m/z) Calcd for $C_{13}H_{14}{}^{79}Br_2N_2O_3SK^+$ $[M+K]^+$ 474.8724 found 474.8710.

3,8-dibromo-6-methoxy-2-morpholinobenzo[*c*][1,2]thiazine 2-oxide (16d)

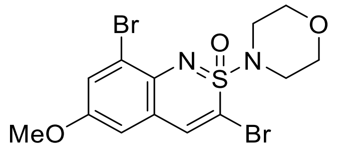

Crude mixture was purified by flash column chromatography (SiO_2, 2% ethyl acetate in DCM) afforded **16d** as a yellow solid (4.0 mg, 5%).

m.p = 110 – 112 °C.

^{1}H NMR (400 MHz, $CDCl_3$): δ 7.78 (s, 1H), 7.39 (d, *J* = 2.8 Hz, 1H), 6.72 (d, *J* = 2.8 Hz, 1H), 3.85 – 3.69 (m, 7H), 3.35 – 3.18 (m, 4H).

^{13}C NMR (101 MHz, $CDCl_3$): δ 153.5, 142.6, 137.5, 123.8, 119.0, 117.8, 110.3, 104.1, 66.7, 56.1, 47.0.

IR: ν_{max} (neat, cm^{-1}) = 2981, 1582, 1274, 1079, 978, 734, 642.

HRMS (ESI +, m/z) Calcd for $C_{13}H_{14}{}^{79}Br_2N_2O_3SK^+$ $[M+K]^+$ 474.8724 found 474.8726.

2-morpholino-3-(triisopropylsilyl)benzo[c][1,2]thiazine 2-oxide (17)

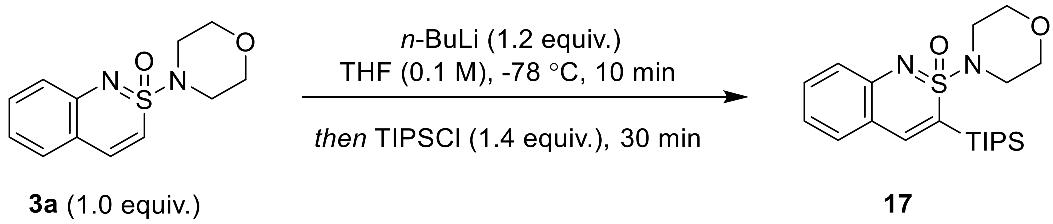

An oven-dried 10 mL microwave vial with a stirring bar was charged with cyclic sulfonimidamide **3a** (25.0 mg, 0.1 mmol, 1.0 equiv.). The vial was evacuated and back-filled with nitrogen gas three times before addition of degassed, anhydrous THF (1.0 mL, 0.1 M). The reaction mixture was cooled to -78 °C and then *n*-BuLi (70 µL, 1.72 M in hexane, 0.12 mmol, 1.2 equiv.) was added to cooled mixture dropwise. After 10 min, TIPSCl (30 µL, 0.14 mmol, 1.4 equiv.) was added. The mixture was stirred for 30 min at -78 °C. Once completed, the reaction was quenched with sat. NH_4Cl and extracted with DCM for 3 times. Combined organic layer was dried by Na_2SO_4, filtered and concentrated under reduced pressure. The crude product was purified by flash column chromatography (pentane/ethyl acetate, 10:1 to 8:1) to afford cyclic sulfonimidamide **17** as a yellow solid (33.0 mg, 81%).

m.p = 100 – 102 °C.

^{1}H NMR (400 MHz, $CDCl_3$): δ 7.82 (s, 1H), 7.39 (ddd, *J* = 8.4, 7.0, 1.6 Hz, 1H), 7.28 (dd, *J* = 7.8, 1.5 Hz, 1H), 7.19 (dd, *J* = 8.3, 1.1 Hz, 1H), 6.93 (ddd, *J* = 8.0, 7.0, 1.2 Hz, 1H), 3.76 – 3.61 (m, 4H), 3.01 (ddd, *J* = 12.6, 6.4, 3.3 Hz, 2H), 2.90 (ddd, *J* = 12.6, 6.3, 3.3 Hz, 2H), 1.51 (hept, *J* = 7.5 Hz, 3H), 1.17 (d, *J* = 7.5 Hz, 18H).

^{13}C NMR (101 MHz, $CDCl_3$): δ 152.1, 147.2, 132.0, 129.4, 122.6, 119.0, 115.5, 112.7, 65.8, 44.7, 18.5, 11.5.

IR: ν_{max} (neat, cm^{-1}) = 2948, 2867, 1608, 1321, 1229, 999, 923, 732.

HRMS (ESI +, m/z) Calcd for $C_{21}H_{34}N_2O_2SSiNa^+$ $[M+Na]^+$ 429.2003 found 429.2005.

The procedure is adapted from literature.[5]

4. Reference

1. T. Q. Davies, M. Tilby, J. Ren, N. A. Parker, D. Skolc, A. Hall, F. Duarte, M. C. Willis, *J. Am. Chem. Soc.* **2020**, *142*, 15445−15453.

2. C. R. Johnson, O. Lavergne, *J. Org. Chem.* **1989**, *54*, 986–988.

3. Chem. Commun, **2017**, *53*, 2064-2067.

4. Z. Zhang, M. C. Willis. Chem, **2022**, *8*, 1137–1146.

5. Synthesis, **2013**, *45,* 1785-1790.

6. *J. Org. Chem.* **1979**, *44*, 13, 2055–2061

5. NMR spectra

O-([1,1'-biphenyl]-4-yl) hydroxylamine (BiPhONH$_2$)(SI-1)

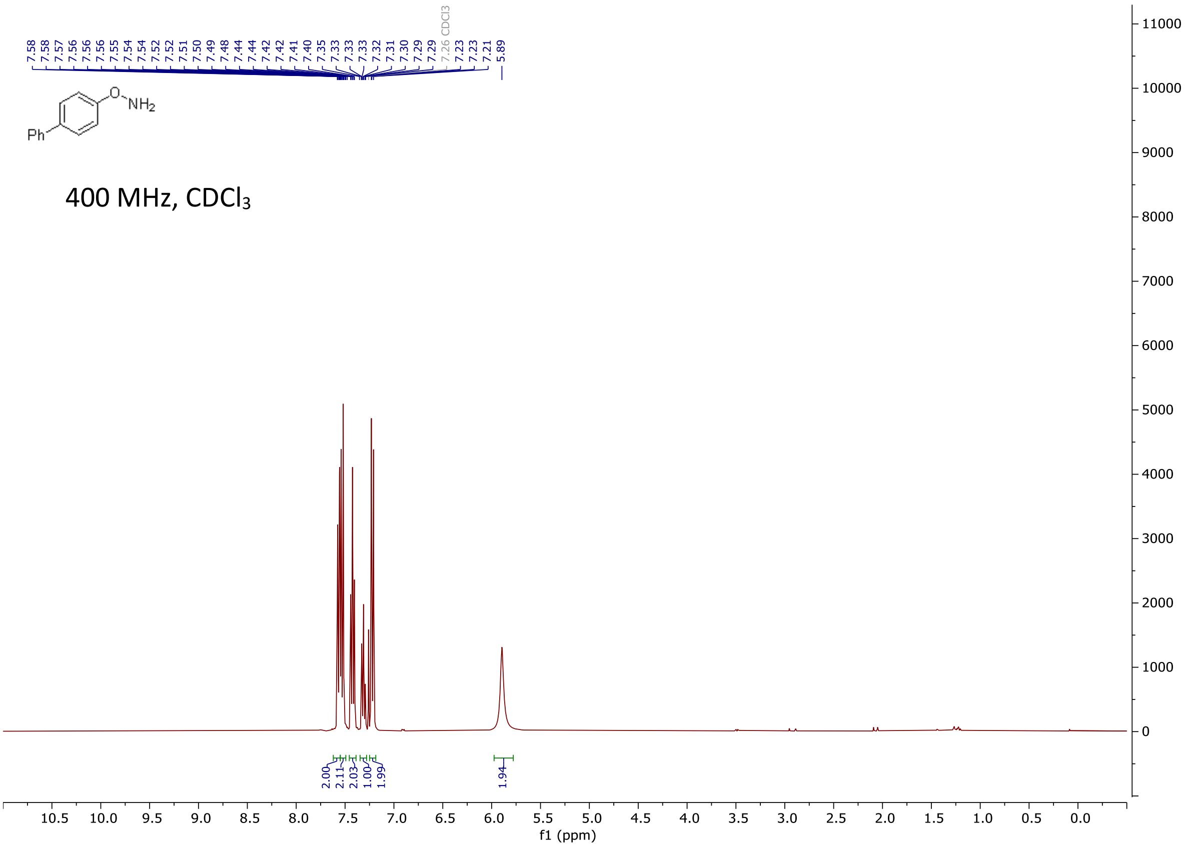

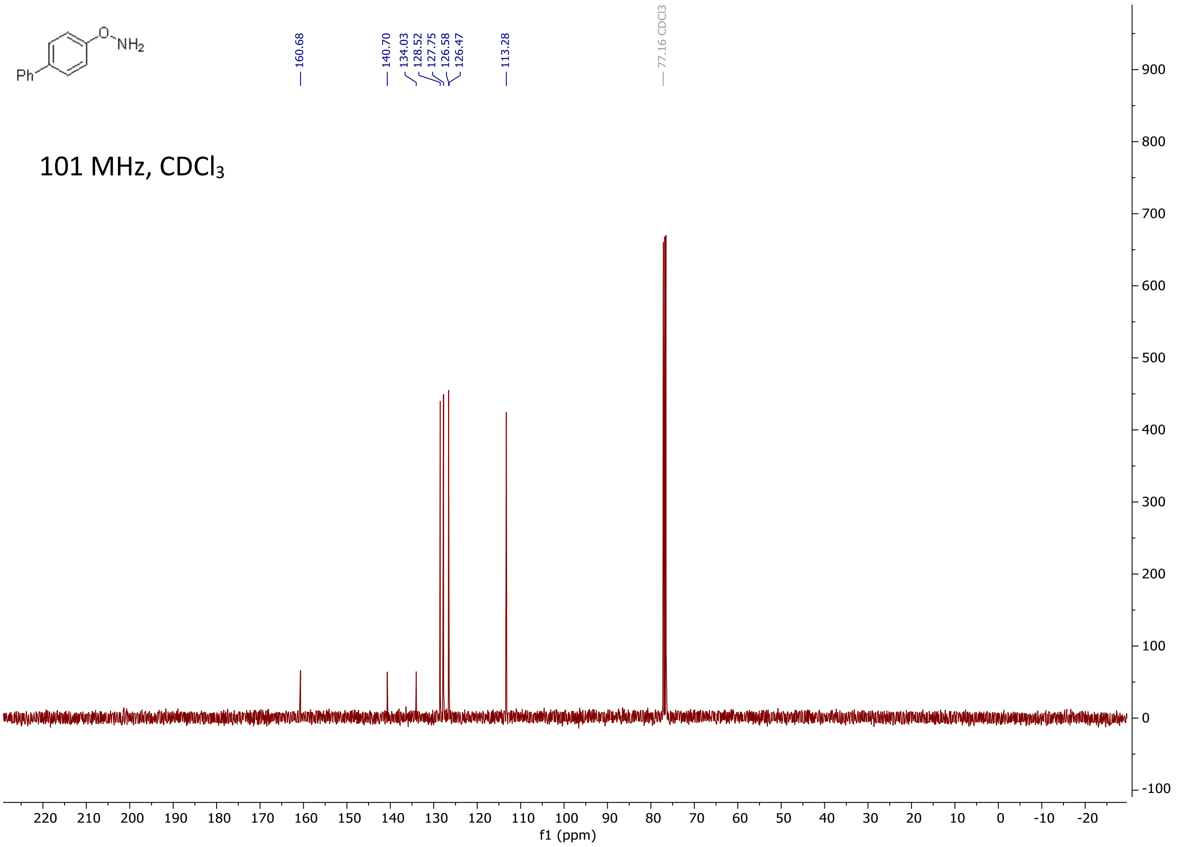

(([1,1'-biphenyl]-4-yloxy)imino)-λ⁴-sulfanone (BiPhONSO) (SI-2)

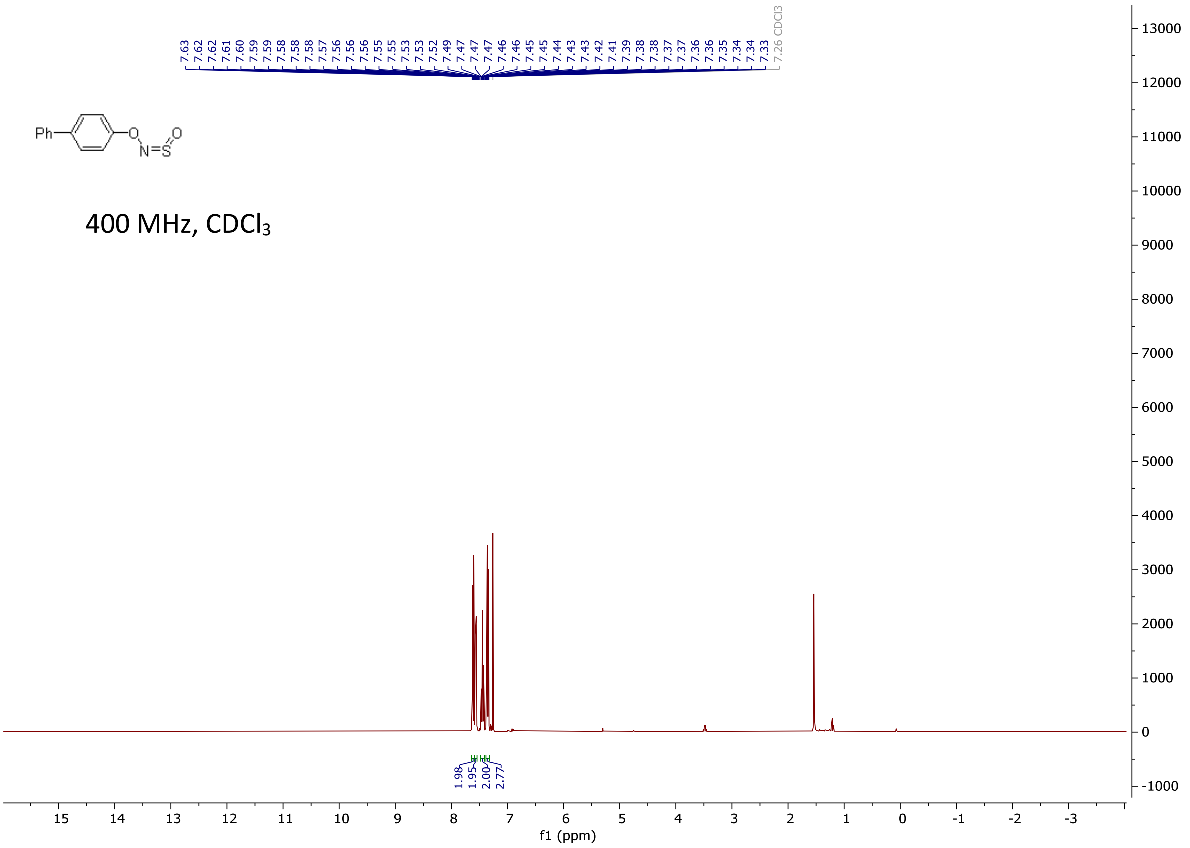

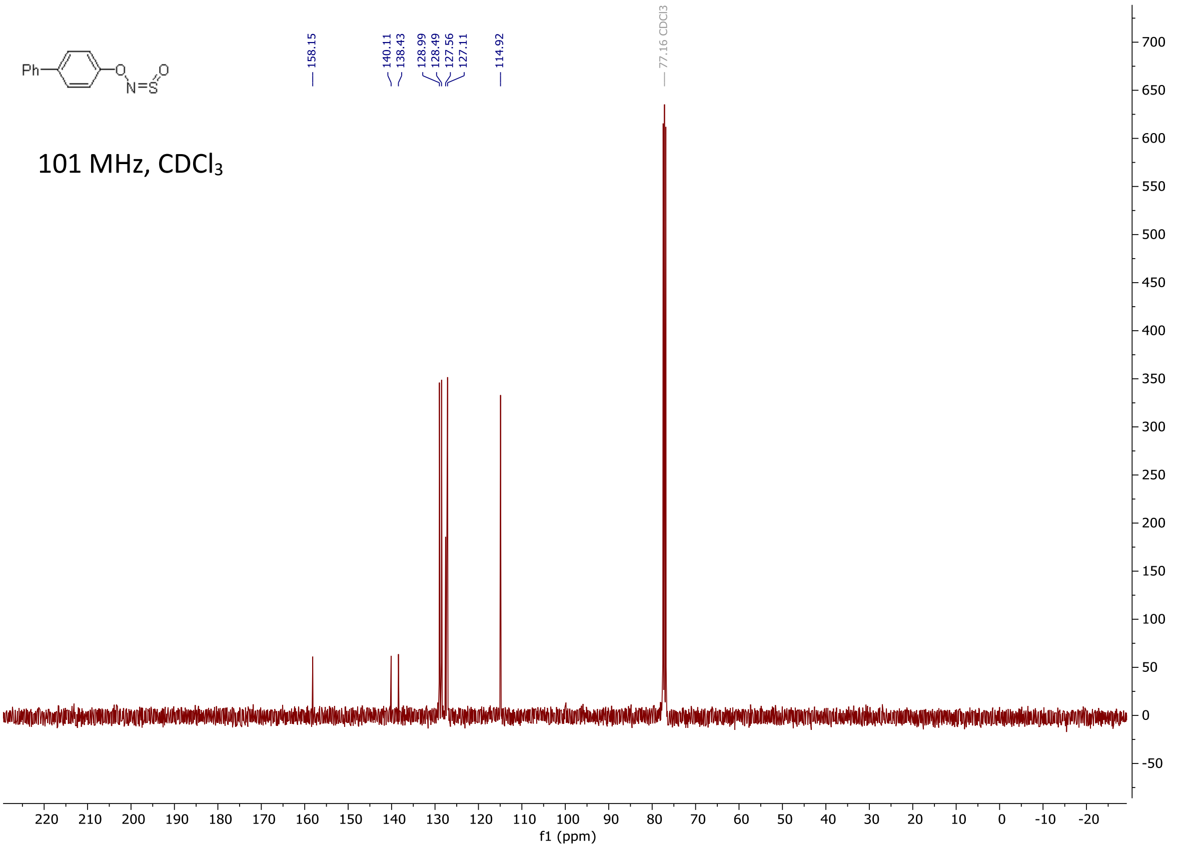

4-(*S*-Methylsulfonimidoyl)morpholine (2a)

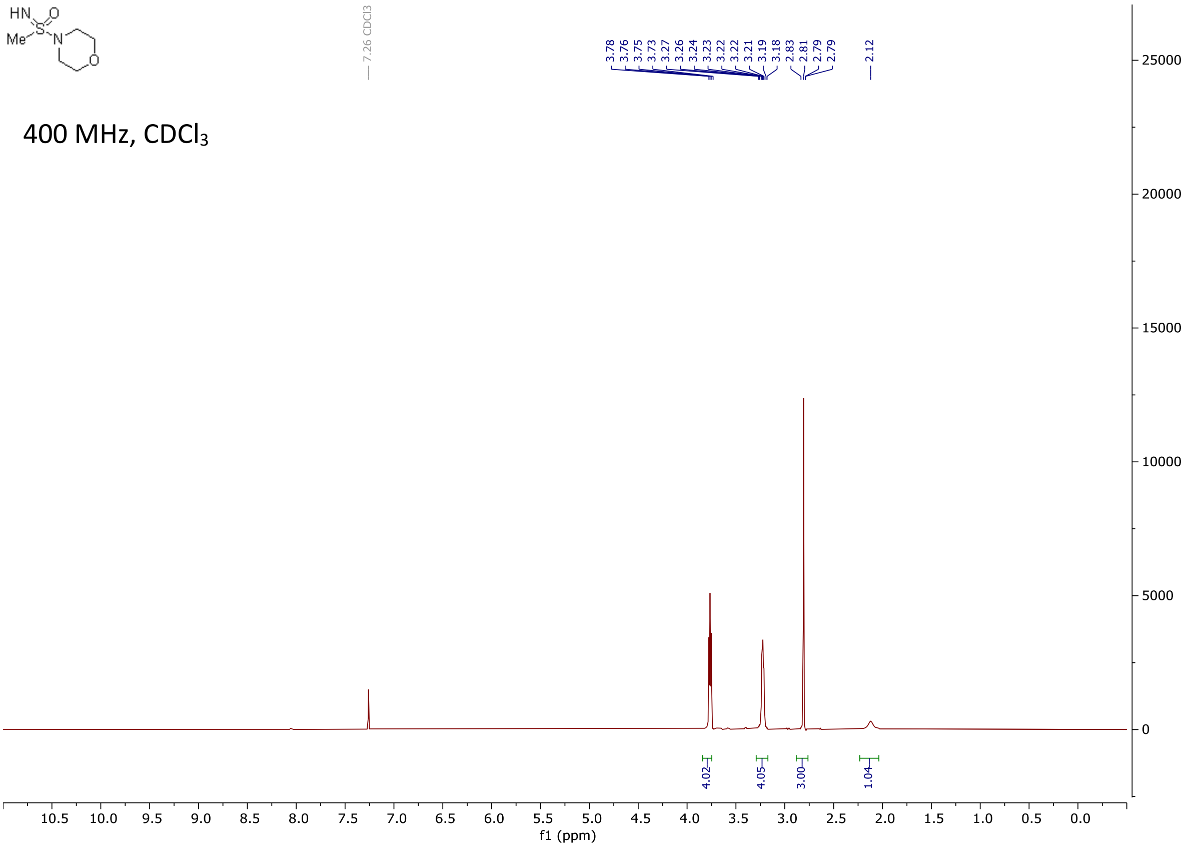

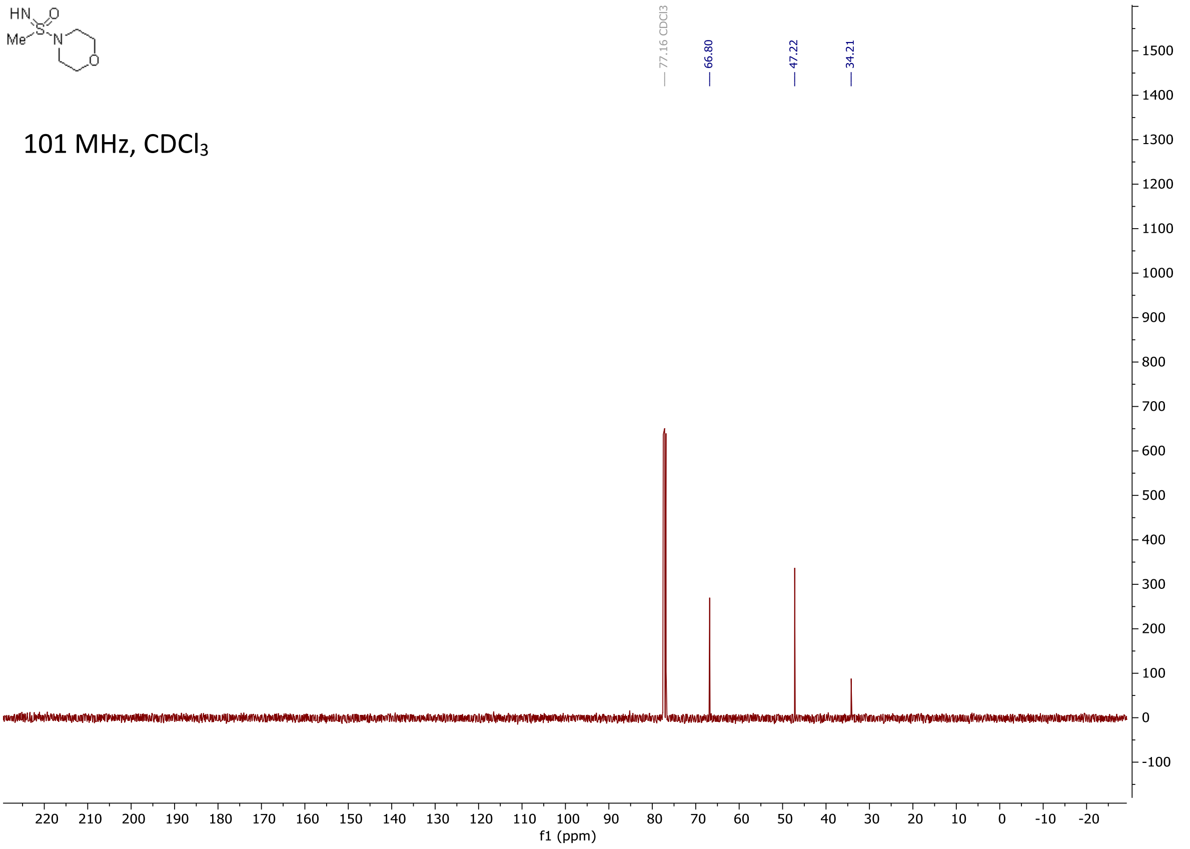

8-(*S*-methylsulfonimidoyl)-1,4-dioxa-8-azaspiro[4.5]decane (2o)

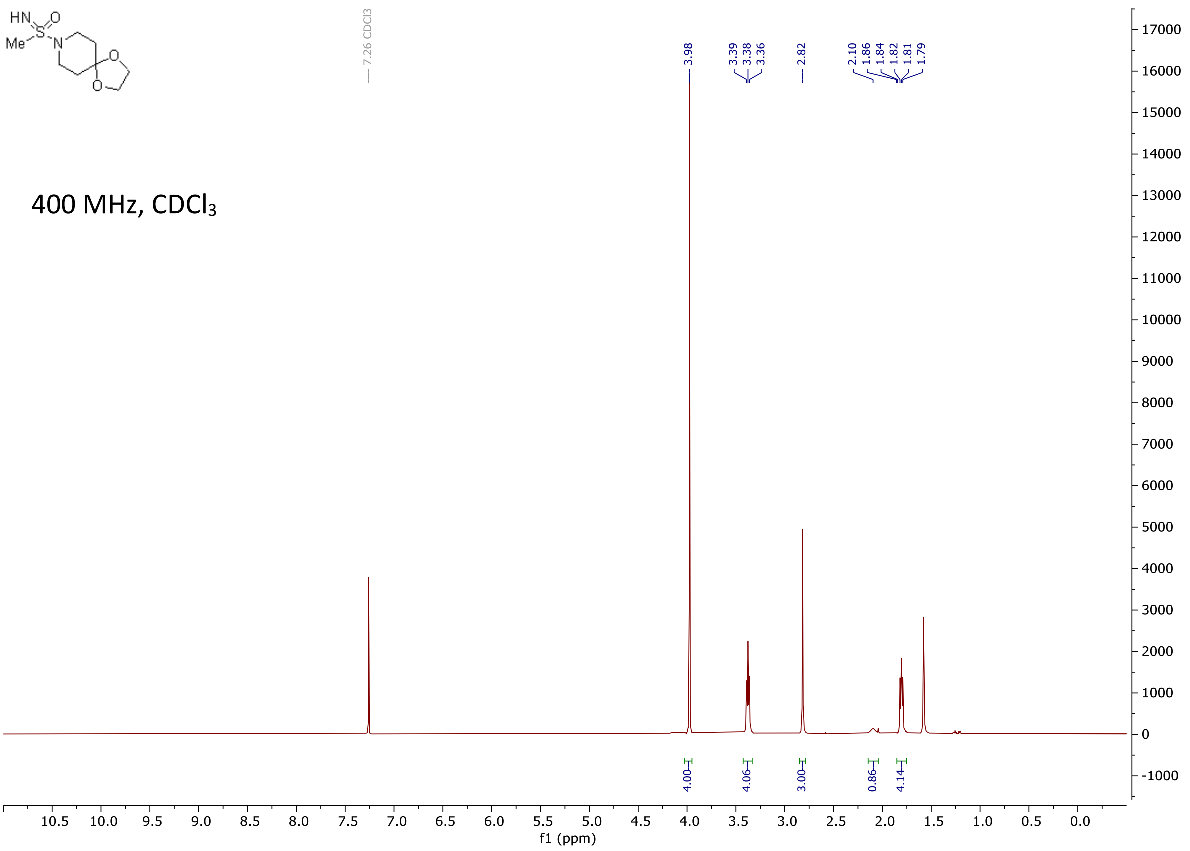

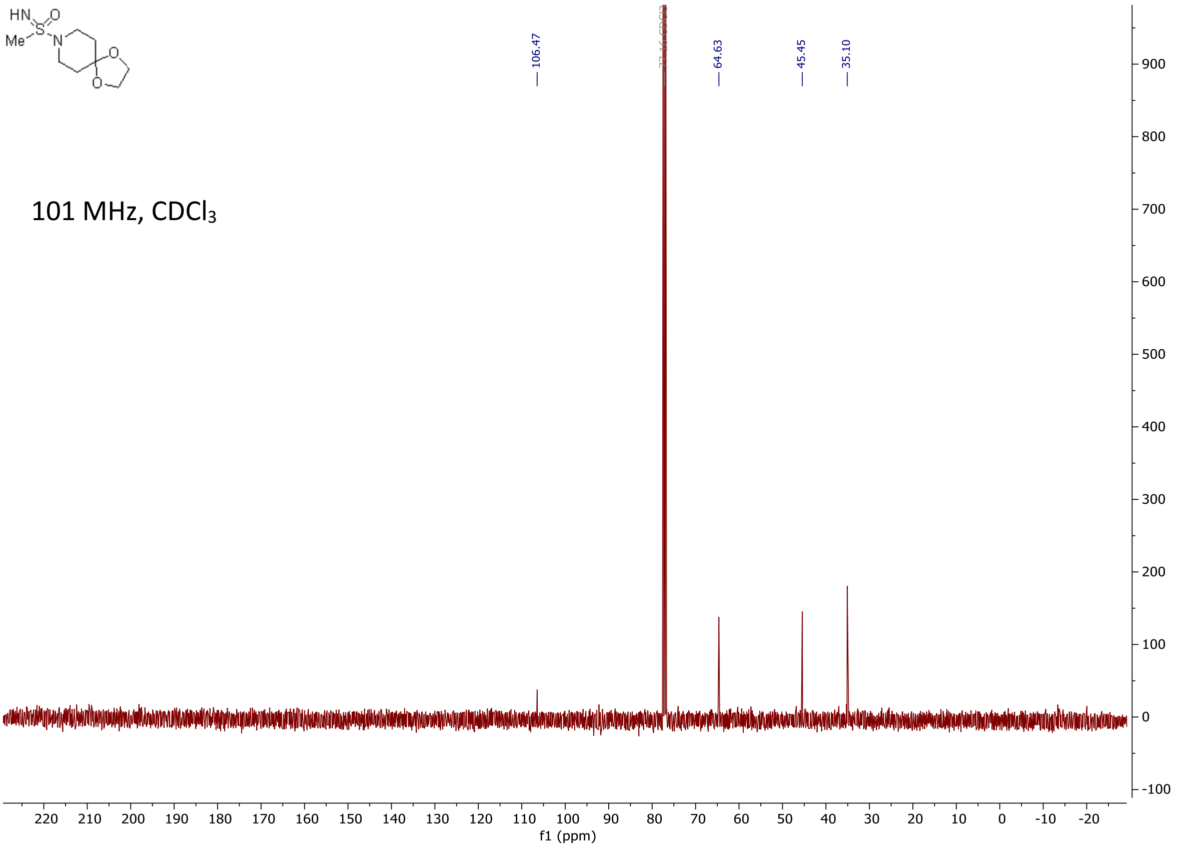

2-(4-(*S*-methylsulfonimidoyl)piperazin-1-yl)pyrimidine (2p)

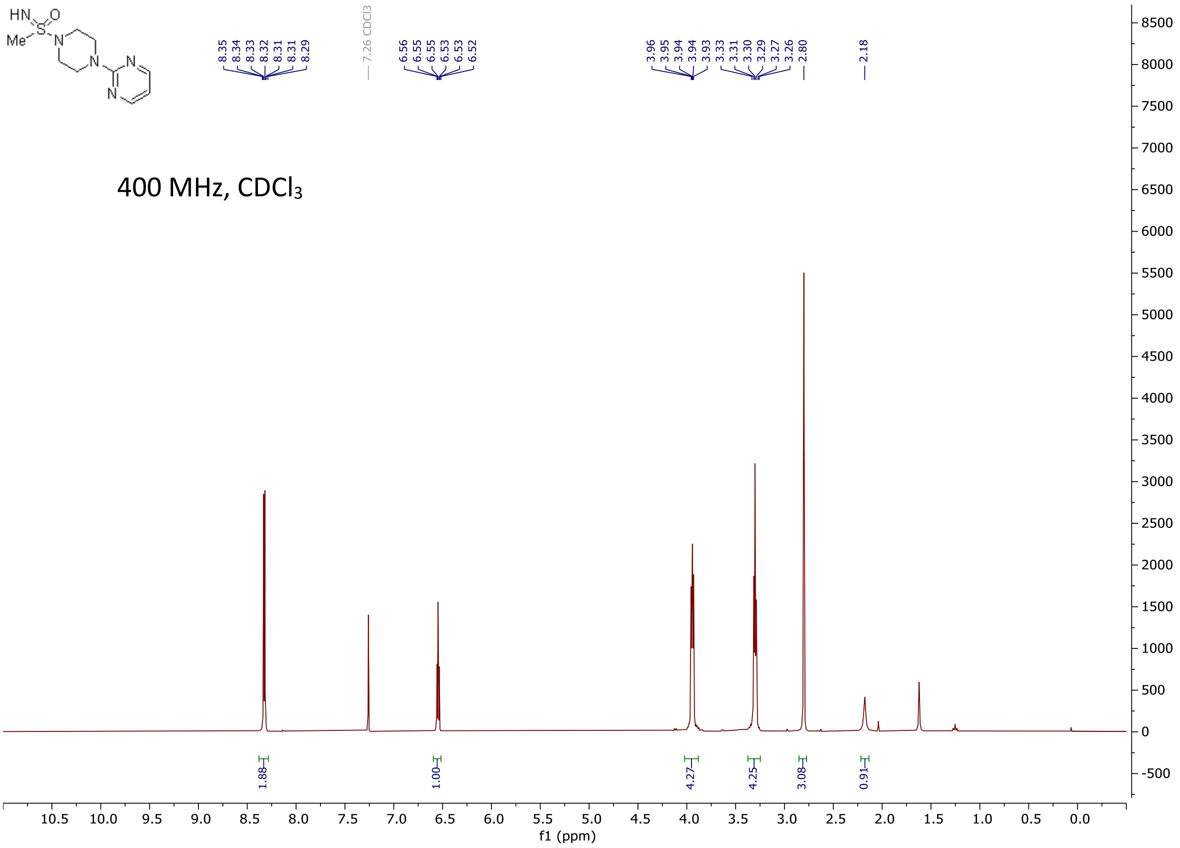

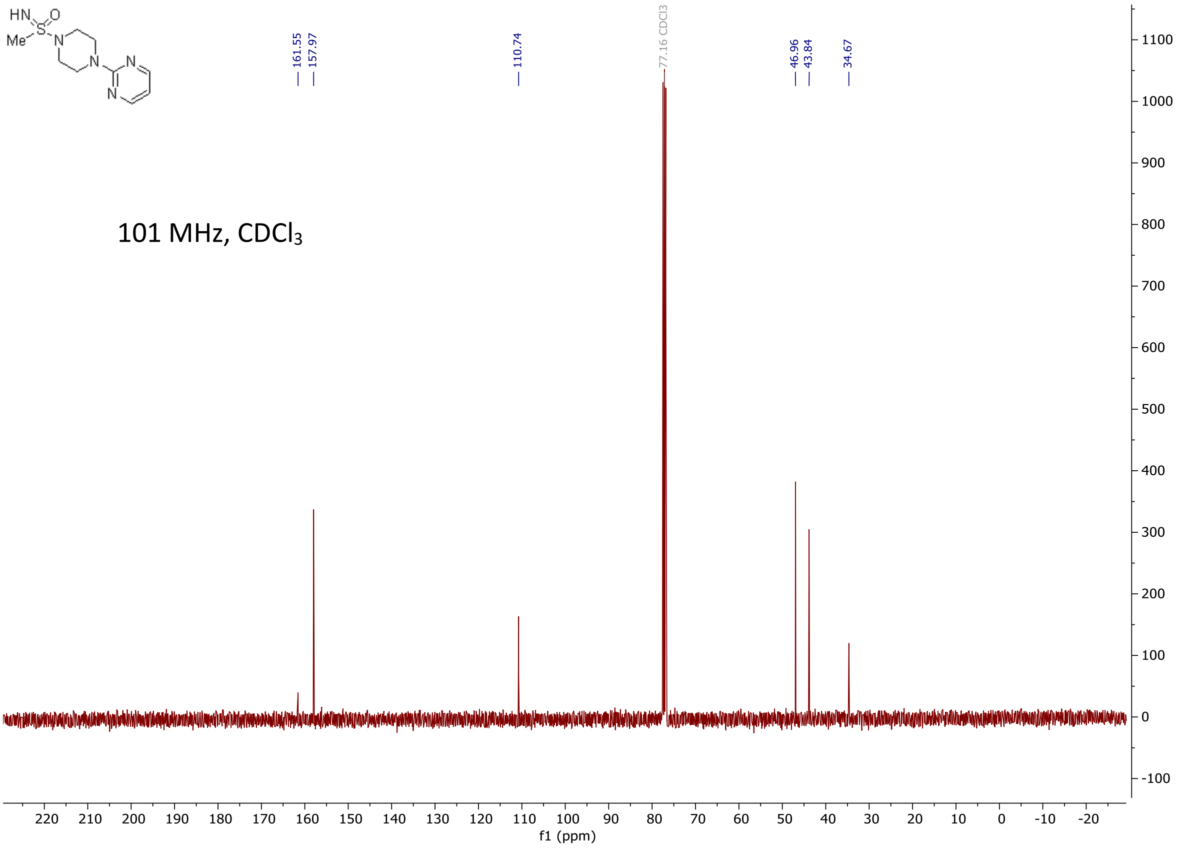

5-(*S*-methylsulfonimidoyl)-4,5,6,7-tetrahydrothieno[3,2-*c*]pyridine (2q)

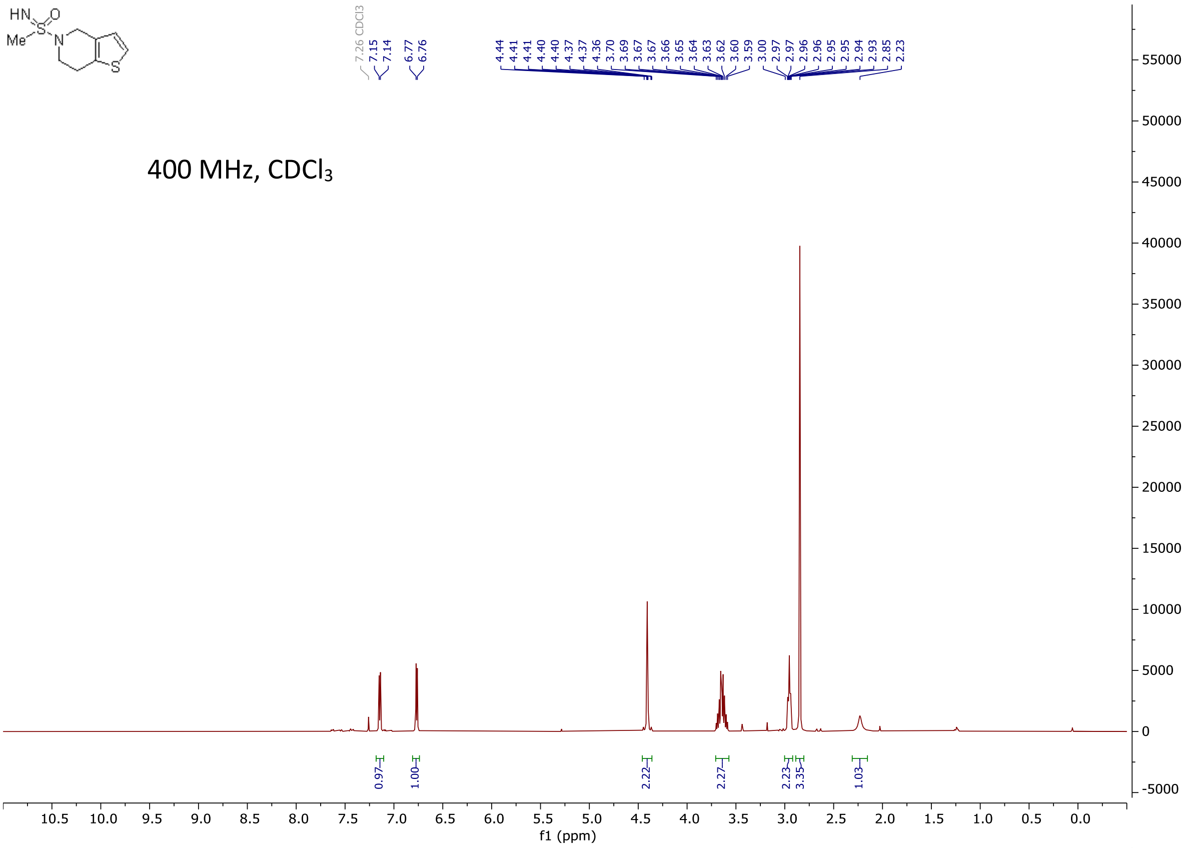

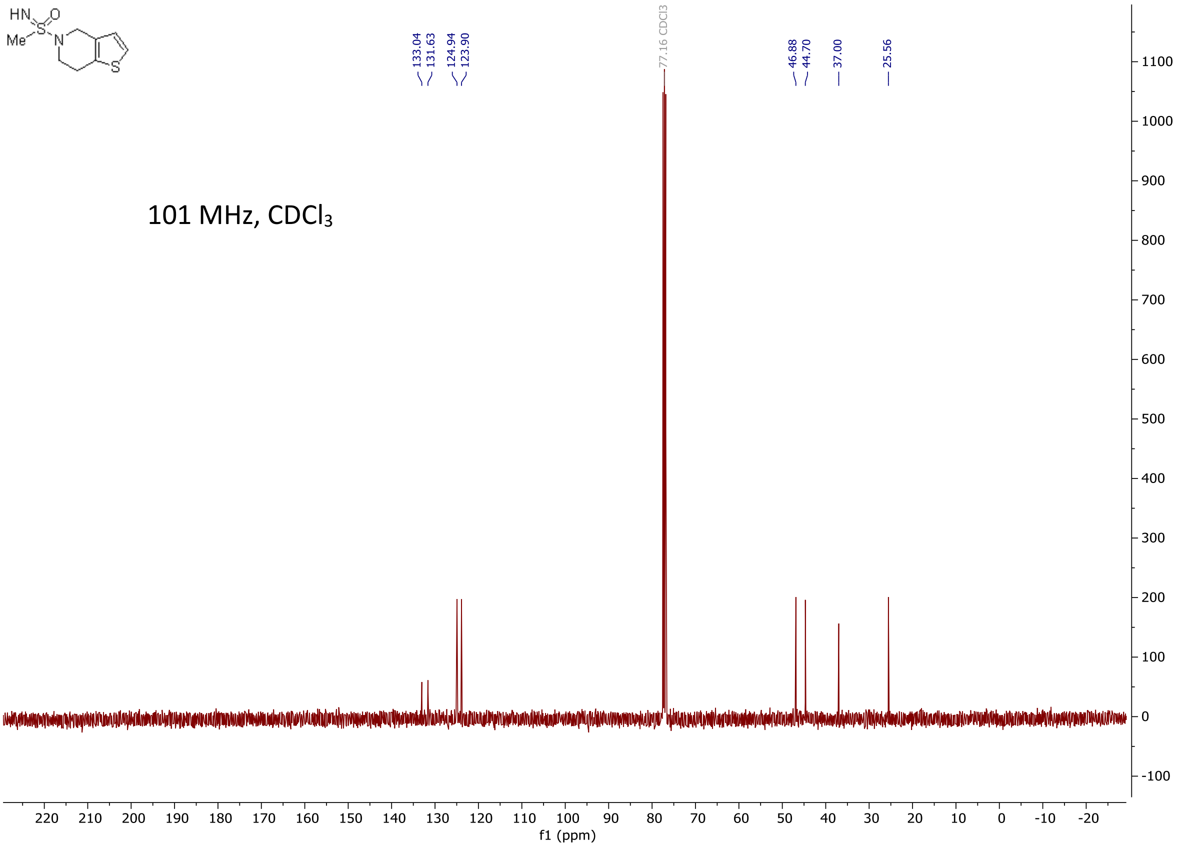

N-(3,4-dimethoxybenzyl)-*N*-methylmethanesulfonimidamide (2r)

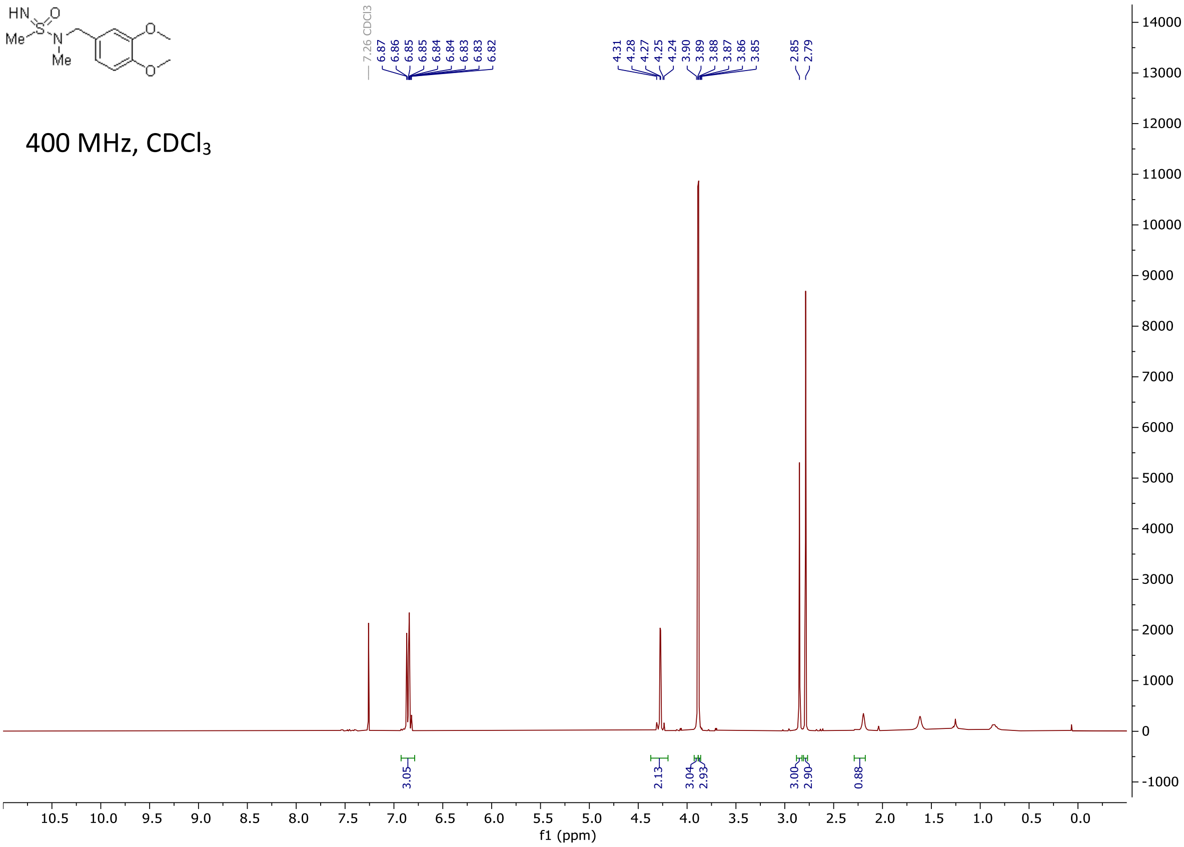

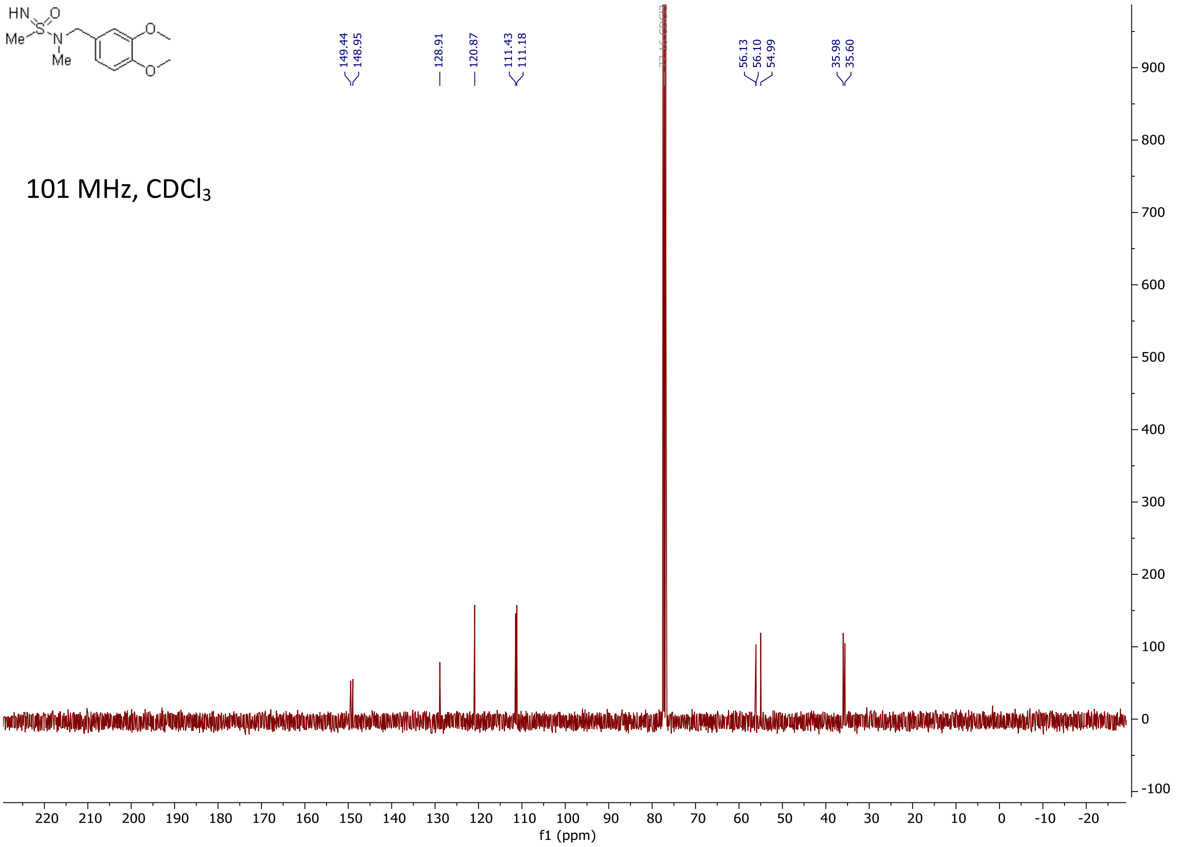

N'-benzylmethanesulfonimidamide (2s)

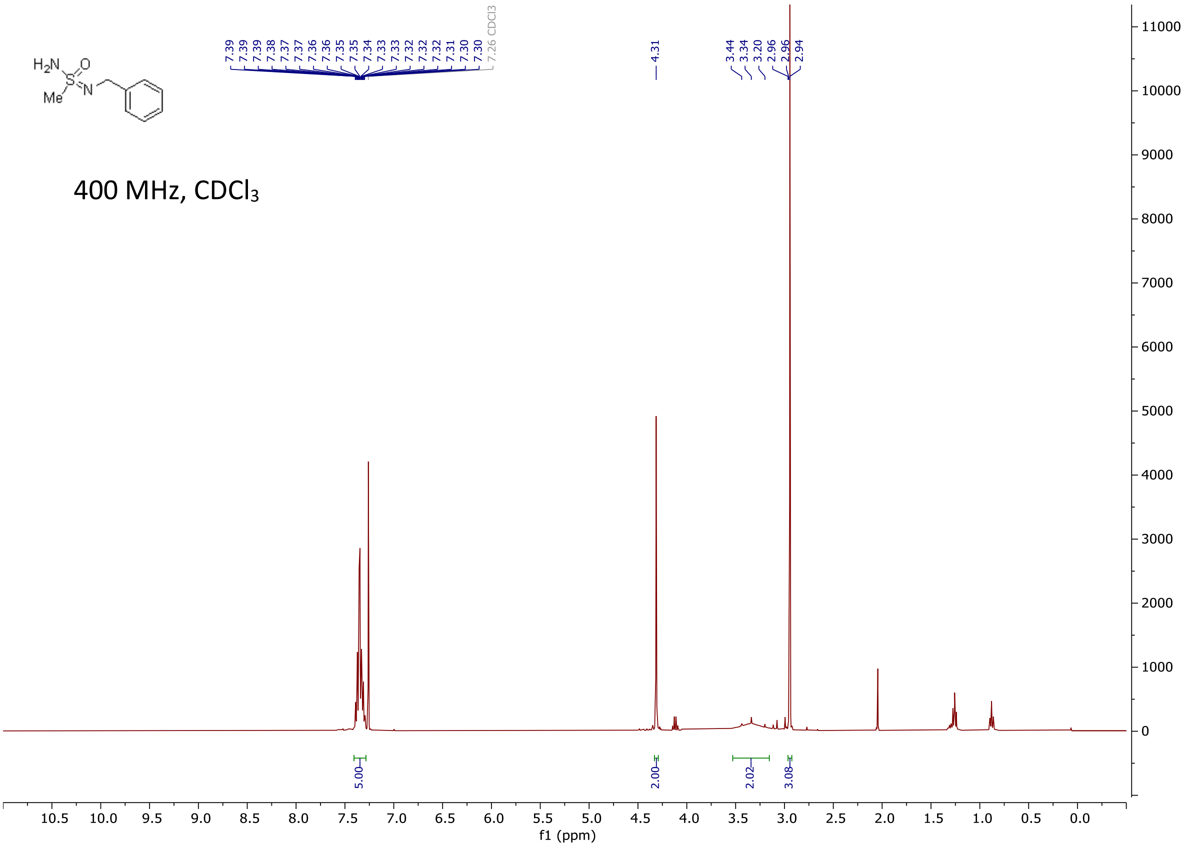
H2N O
S
Me N
400 MHz, CDCl3
7.39 7.39 7.39 7.38 7.37 7.37 7.36 7.36 7.35 7.35 7.34 7.33 7.33 7.32 7.32 7.32 7.31 7.30 7.30
7.26 CDCl3
4.31
3.44 3.34 3.20 2.96 2.96 2.94
5.00
2.00
2.02
3.08
f1 (ppm)

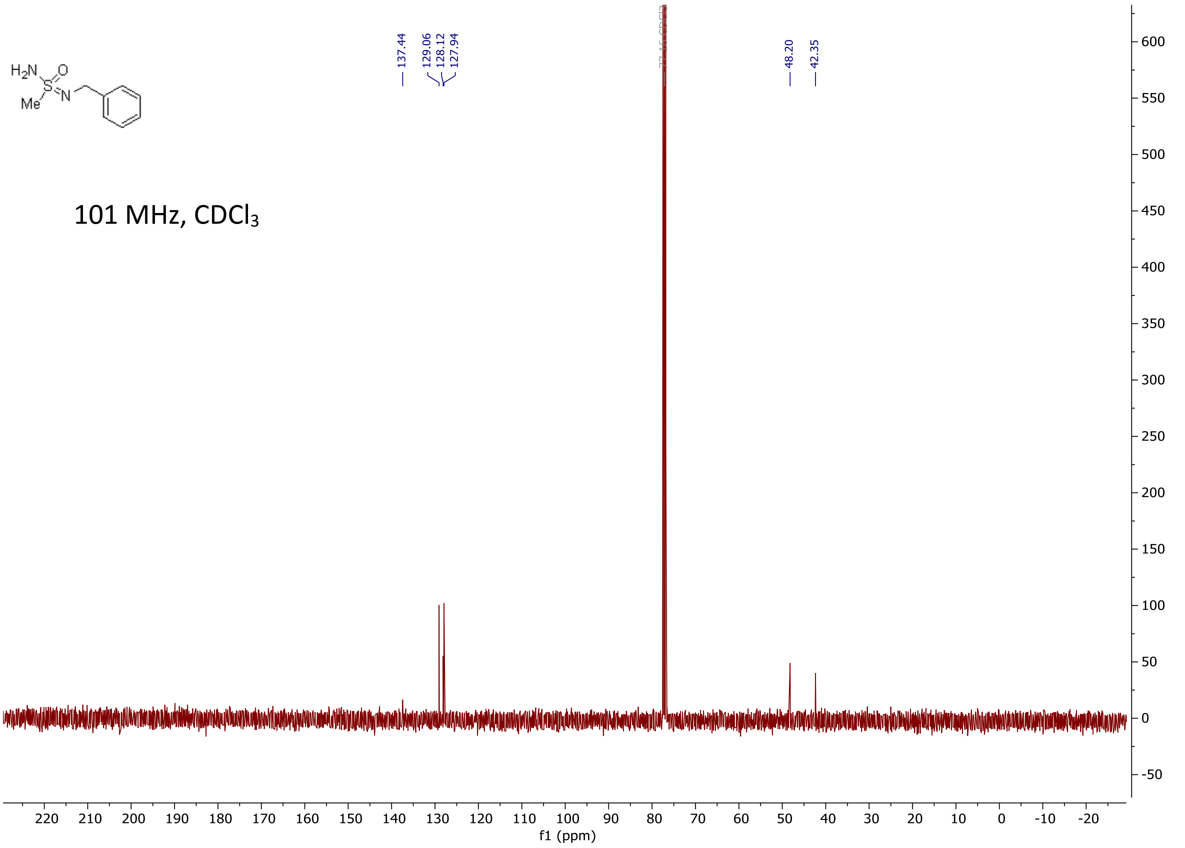
H2N O
S
Me N
101 MHz, CDCl3
137.44
129.06 128.12 127.94
48.20
42.35
f1 (ppm)

4-(ethylsulfonimidoyl)morpholine (2t)

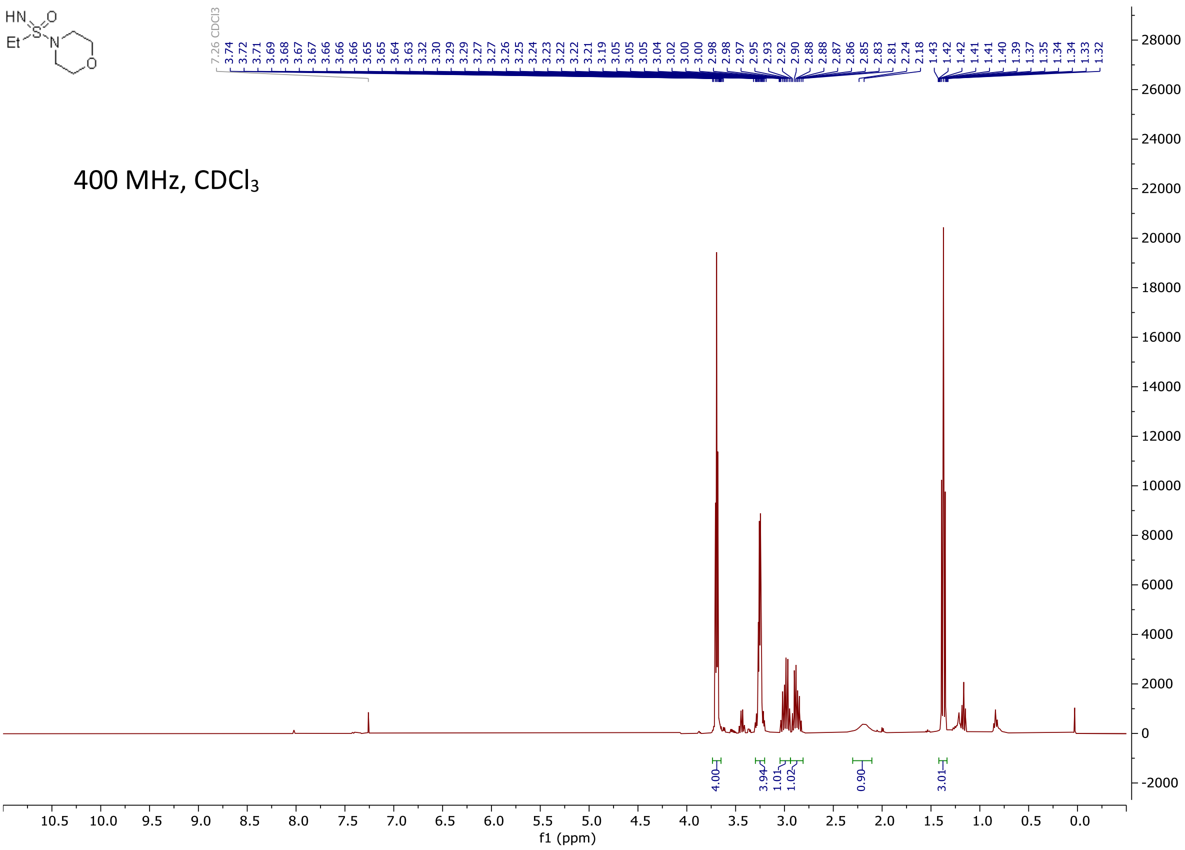

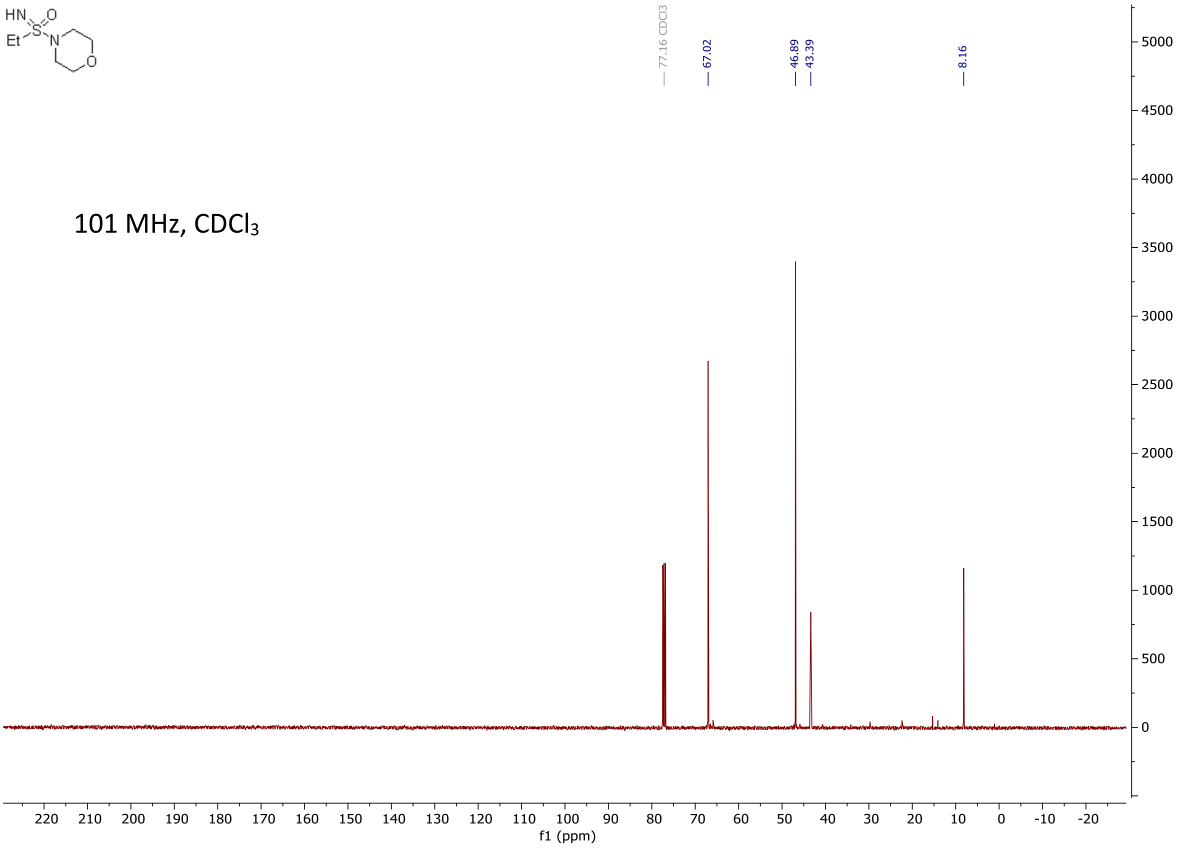

4-(S-benzylsulfonimidoyl)morpholine (2u)

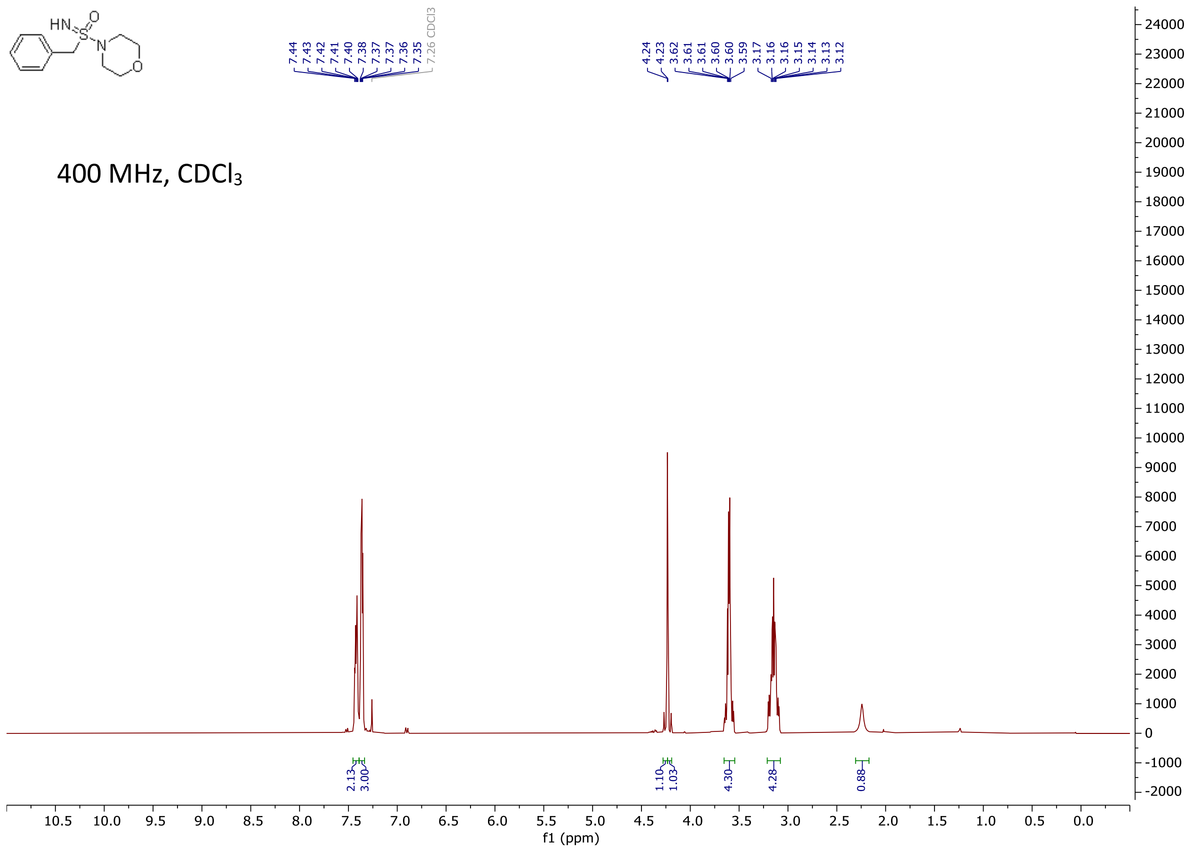

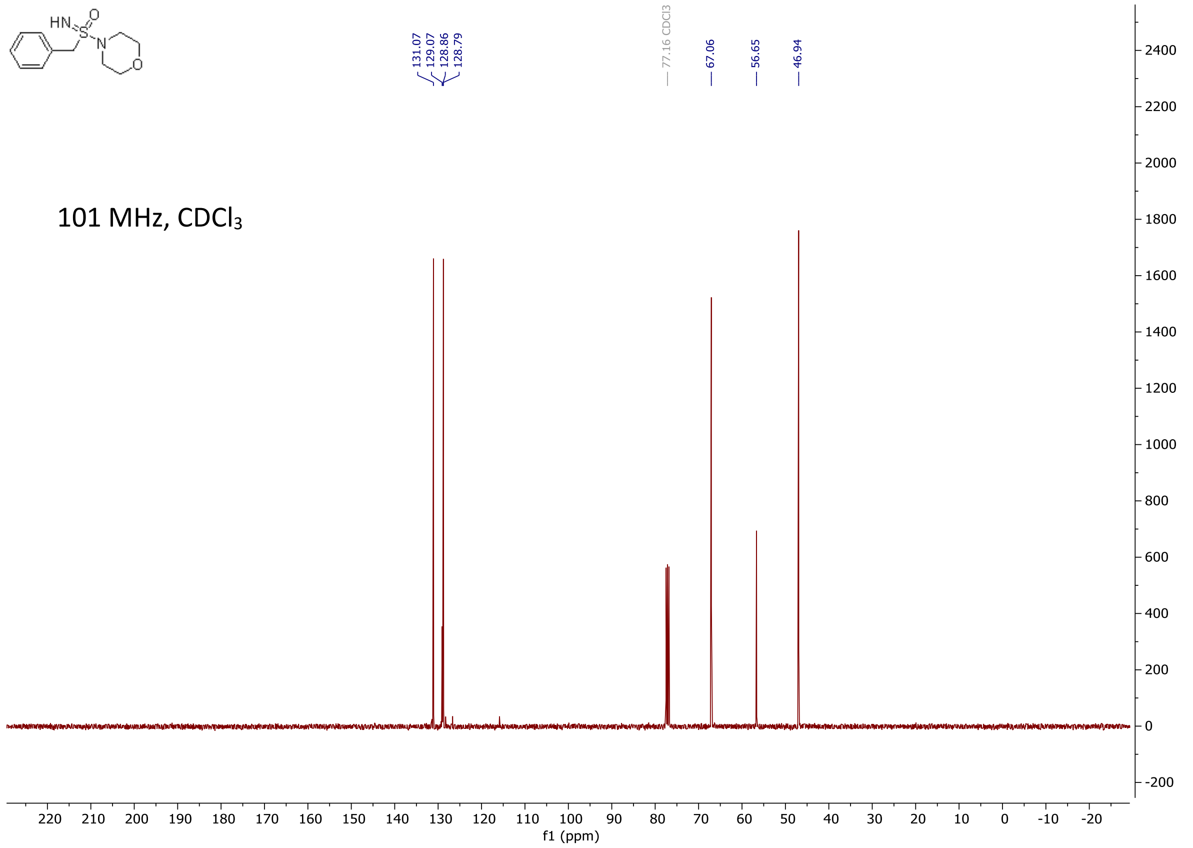

4-(allylsulfonimidoyl)morpholine (2v)

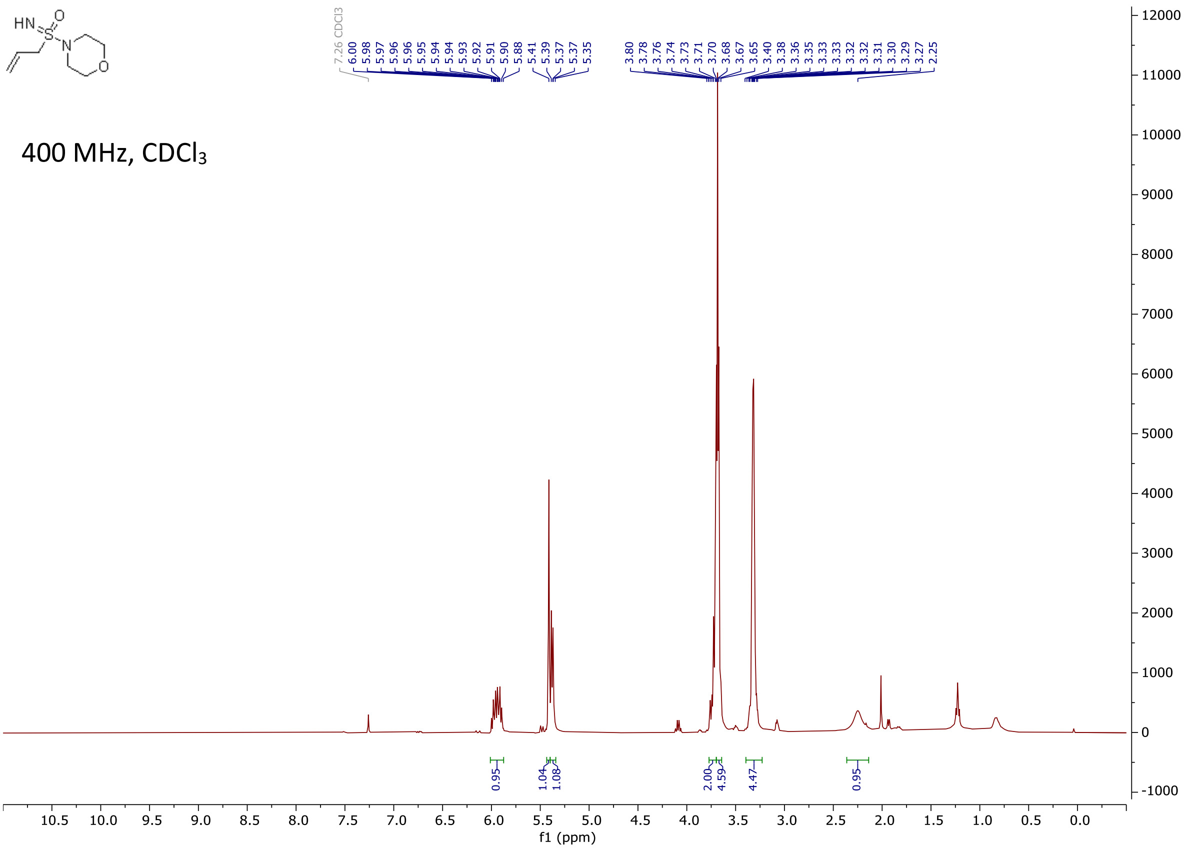

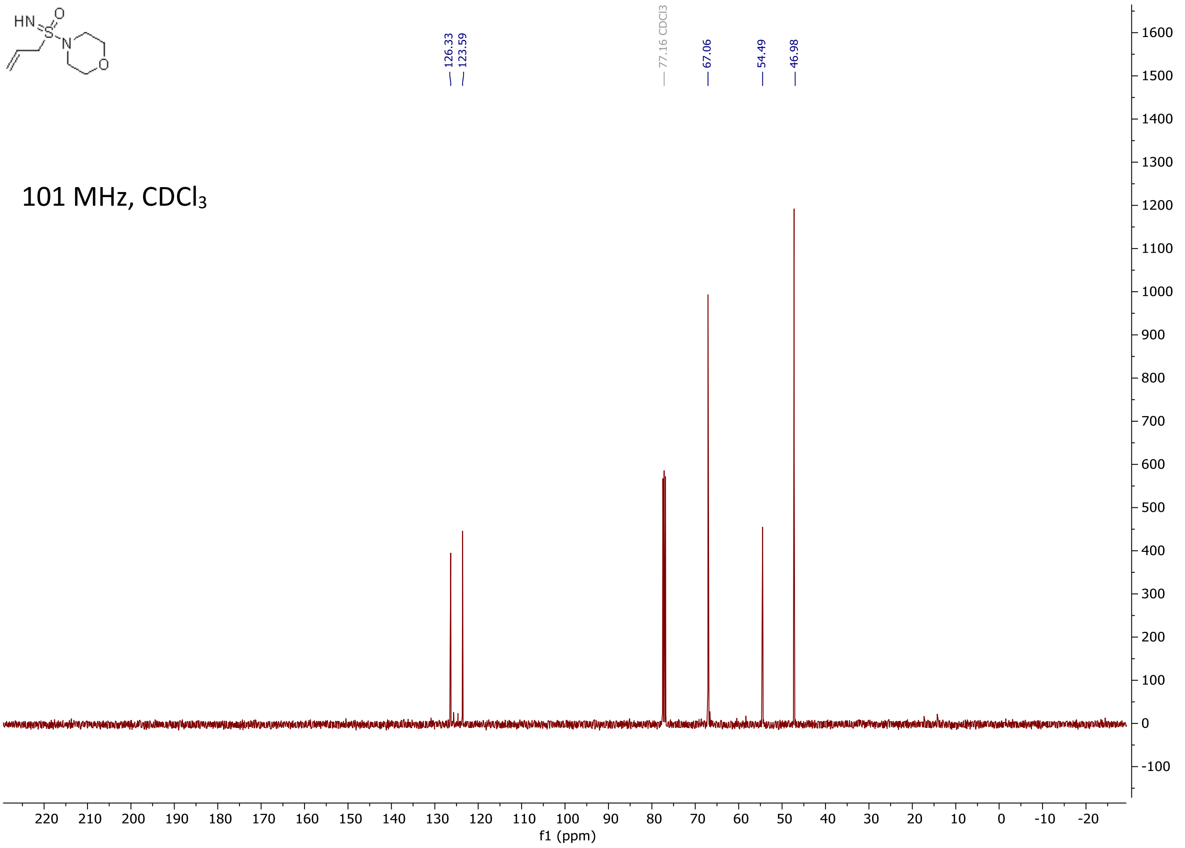

N'-cyclopropylmethanesulfonimidamide (SI-3)

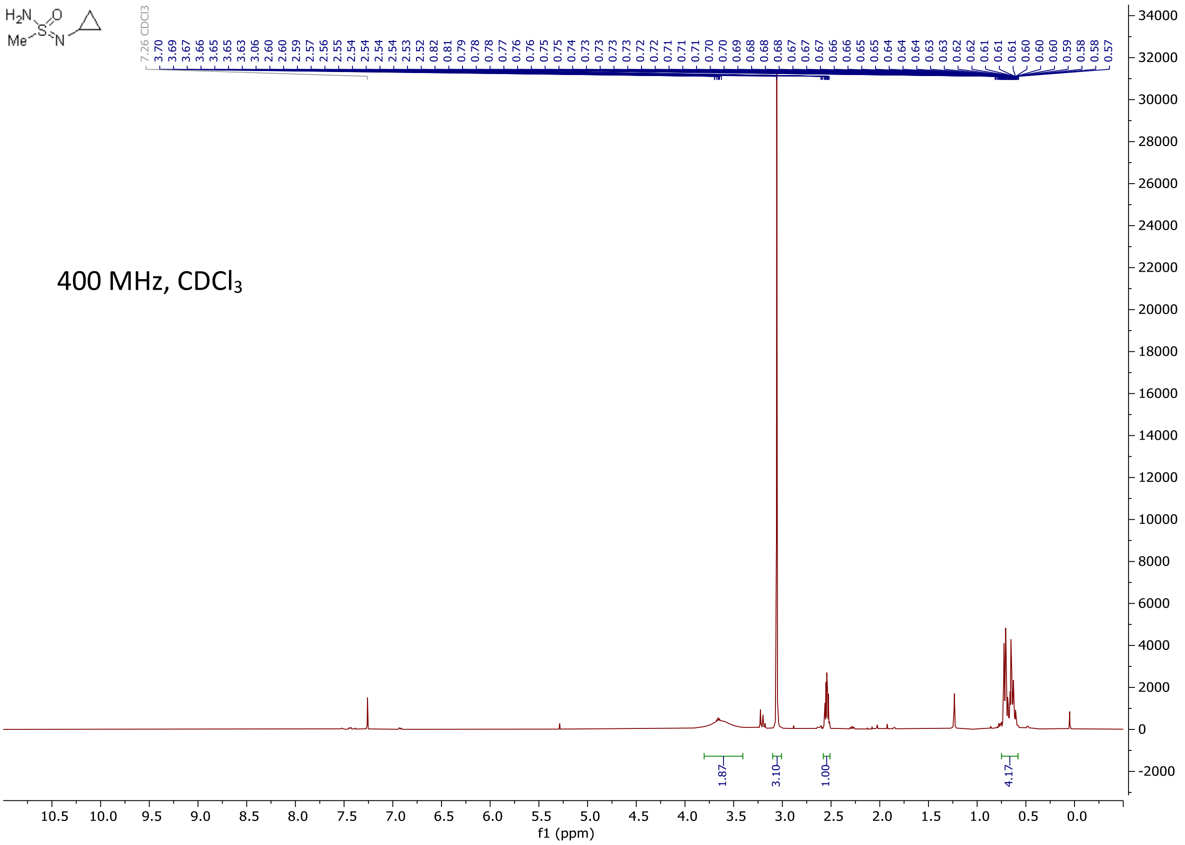

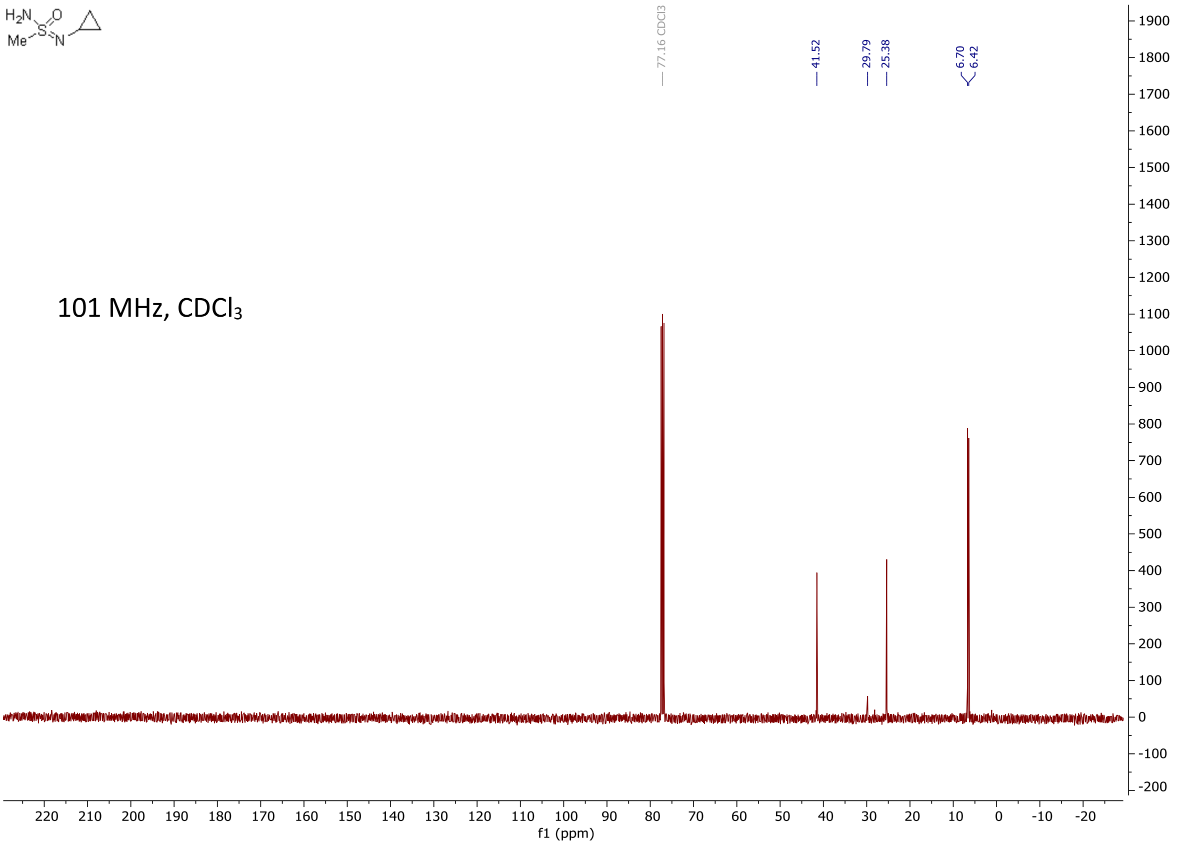

N'-butylmethanesulfonimidamide (SI-4)

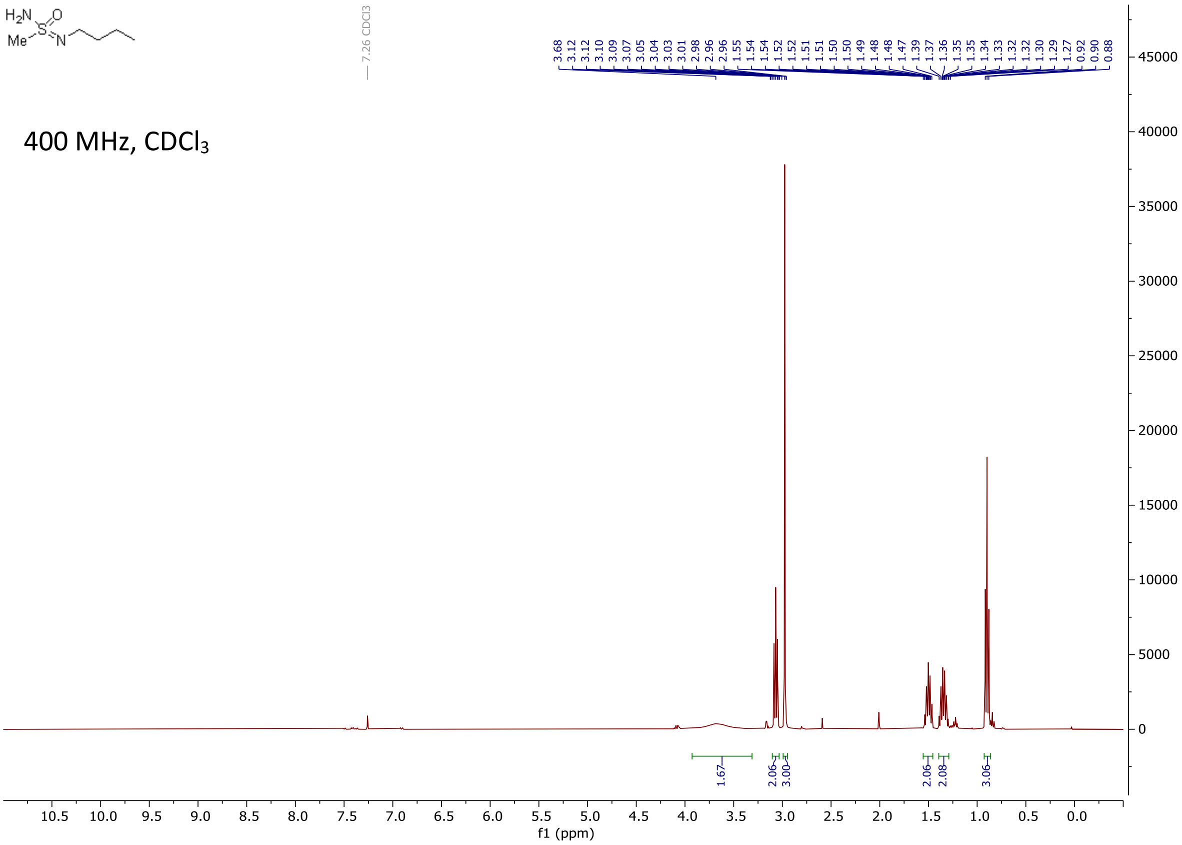
H2N O
Me S N
400 MHz, CDCl3
7.26 CDCl3
3.68 3.12 3.12 3.10 3.09 3.07 3.05 3.04 3.03 3.01 2.98 2.96 2.96 1.55 1.54 1.54 1.52 1.52 1.51 1.51 1.50 1.50 1.49 1.48 1.48 1.47 1.39 1.37 1.36 1.35 1.35 1.34 1.33 1.32 1.32 1.30 1.29 1.27 0.92 0.90 0.88
1.67 2.06 3.00 2.06 2.08 3.06
f1 (ppm)

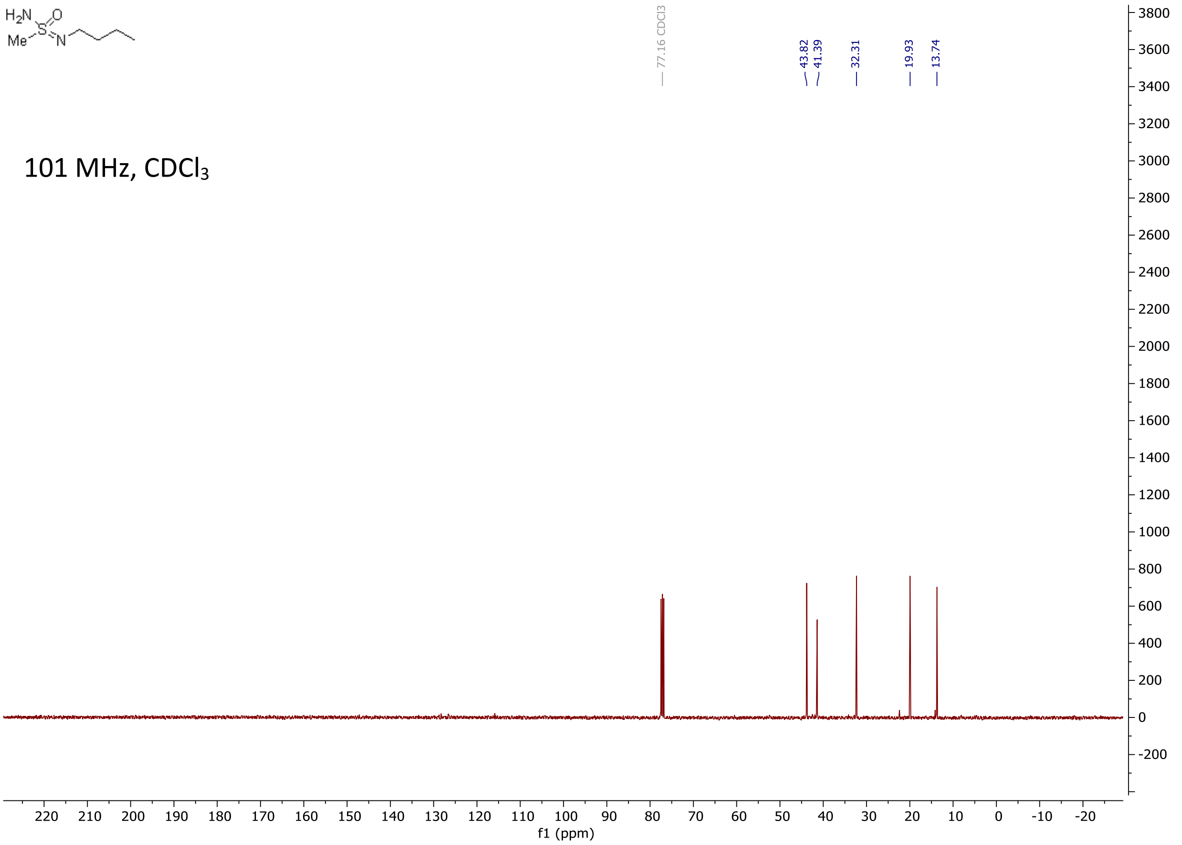
H2N O
Me S N
101 MHz, CDCl3
77.16 CDCl3
43.82
41.39
32.31
19.93
13.74
f1 (ppm)

2-Morpholinobenzo[c][1,2]thiazine 2-oxide (3a)

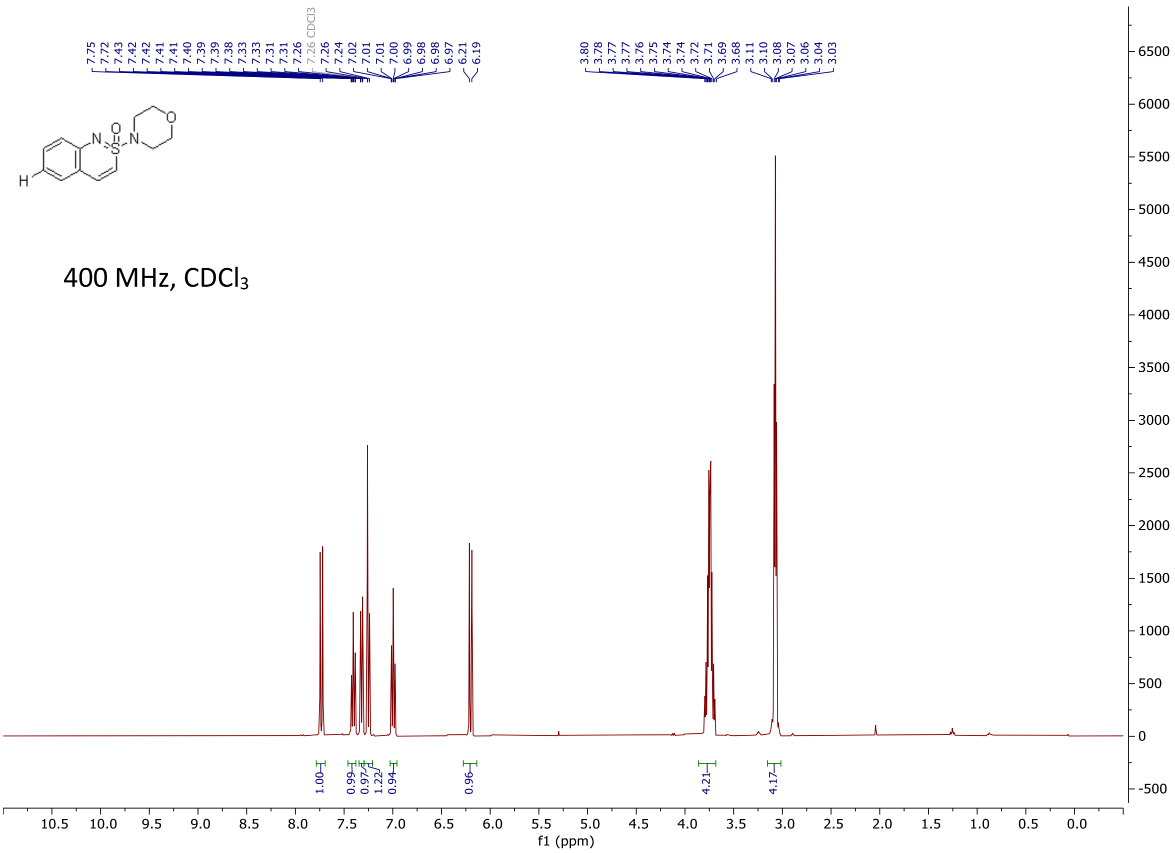

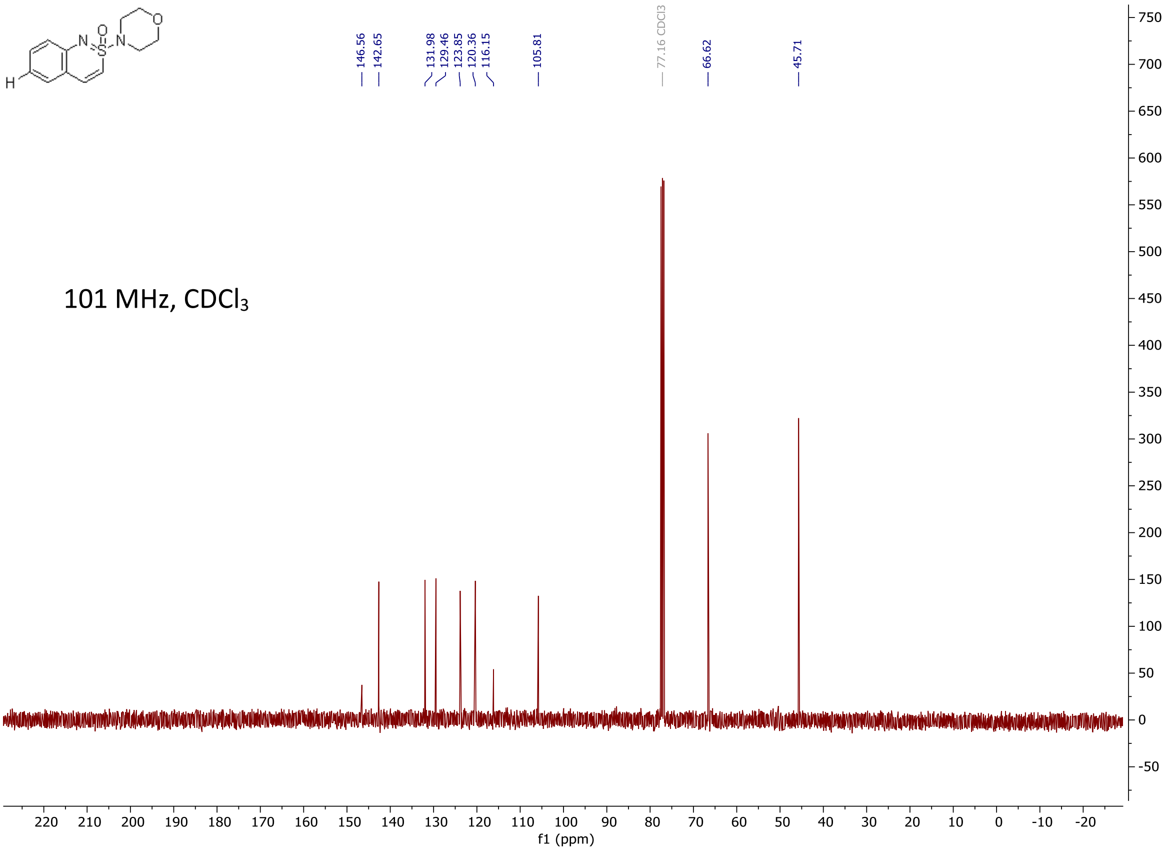

6-Methoxy-2-morpholinobenzo[c][1,2]thiazine 2-oxide (3b)

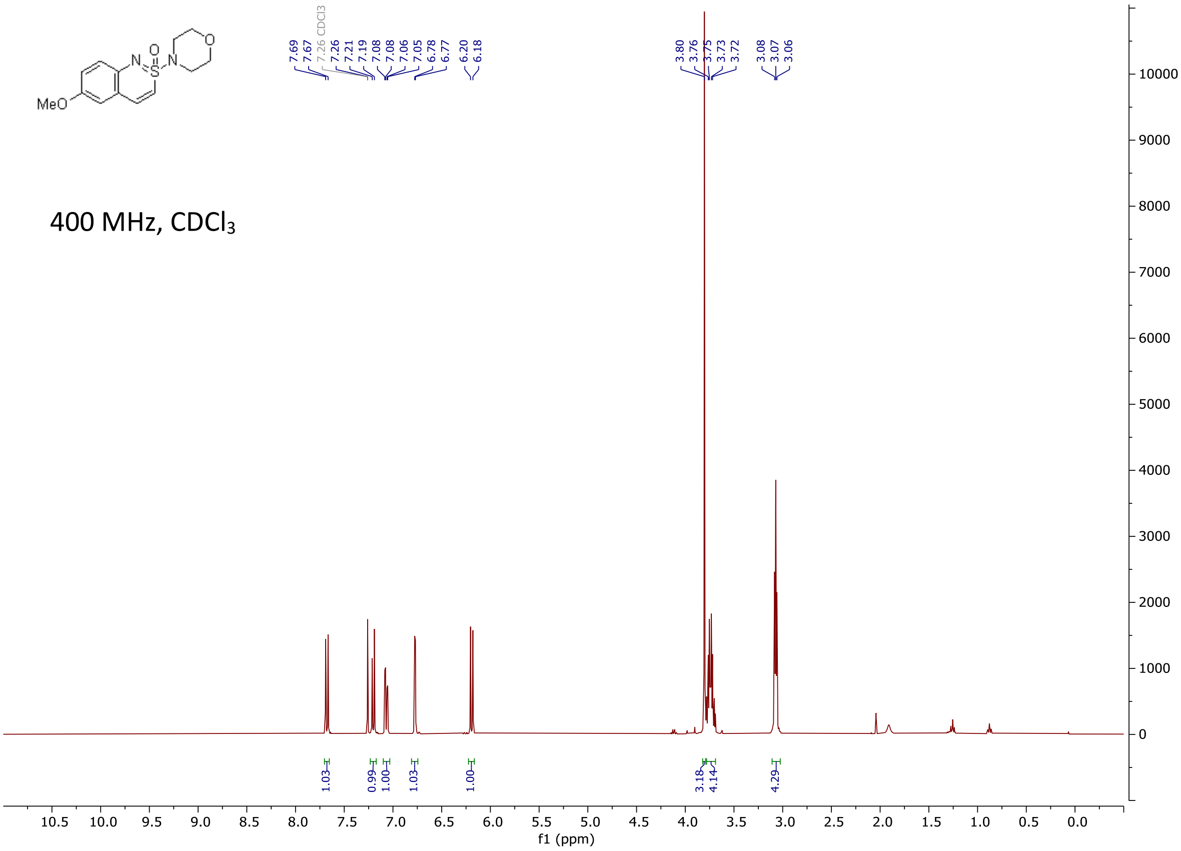

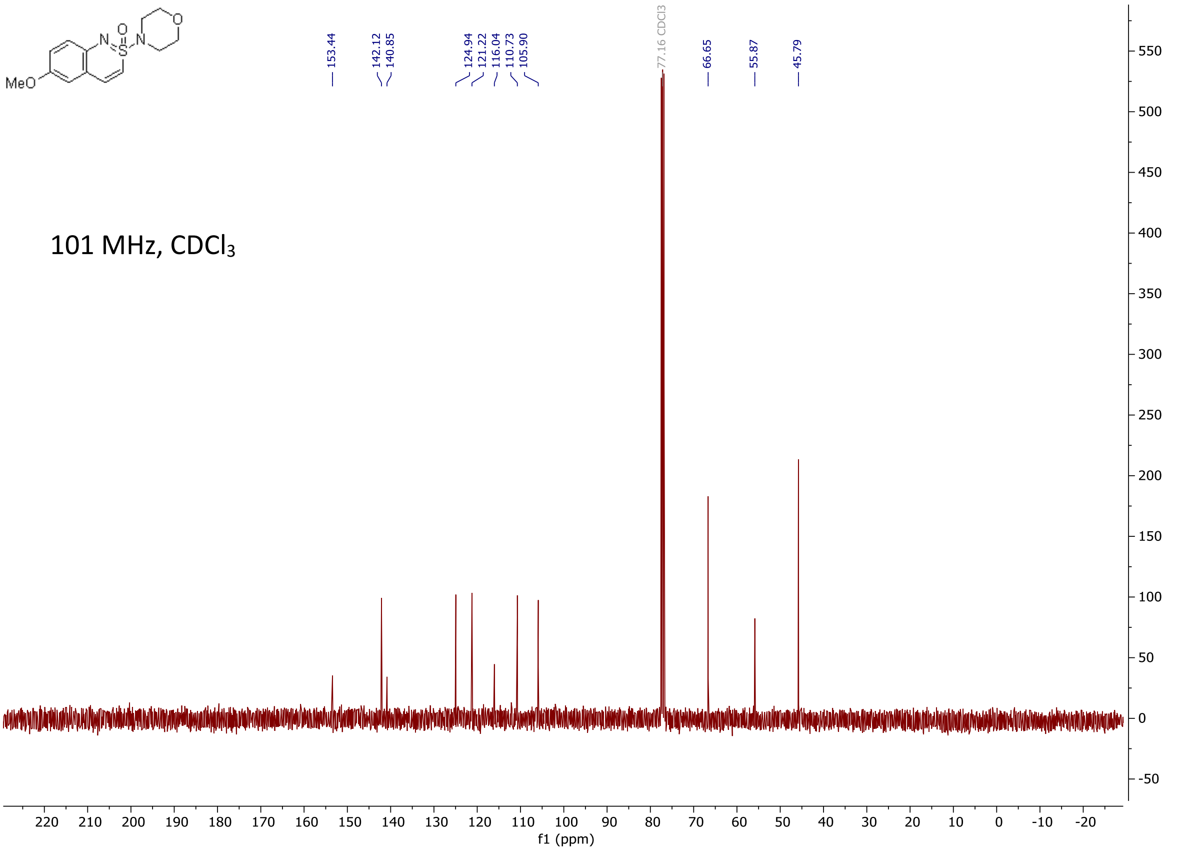

6-Chloro-2-morpholinobenzo[c][1,2]thiazine 2-oxide (3c)

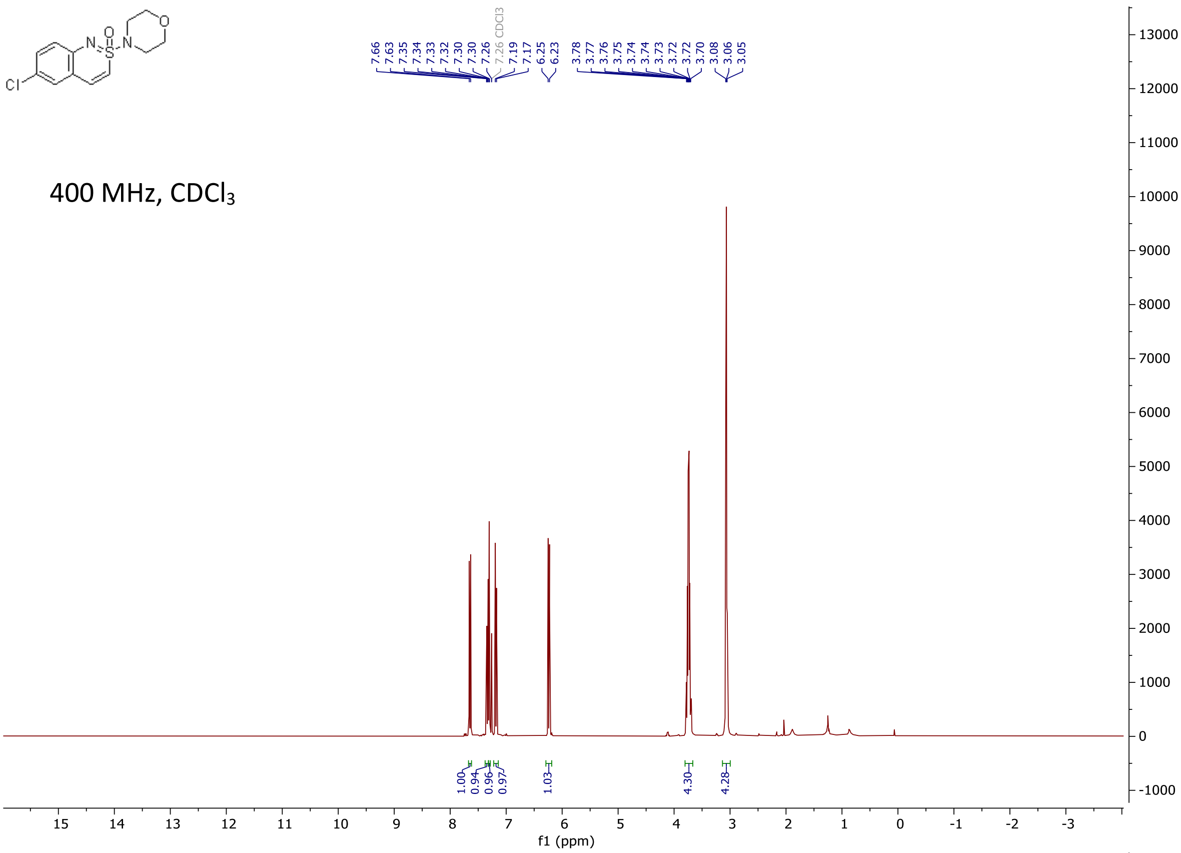

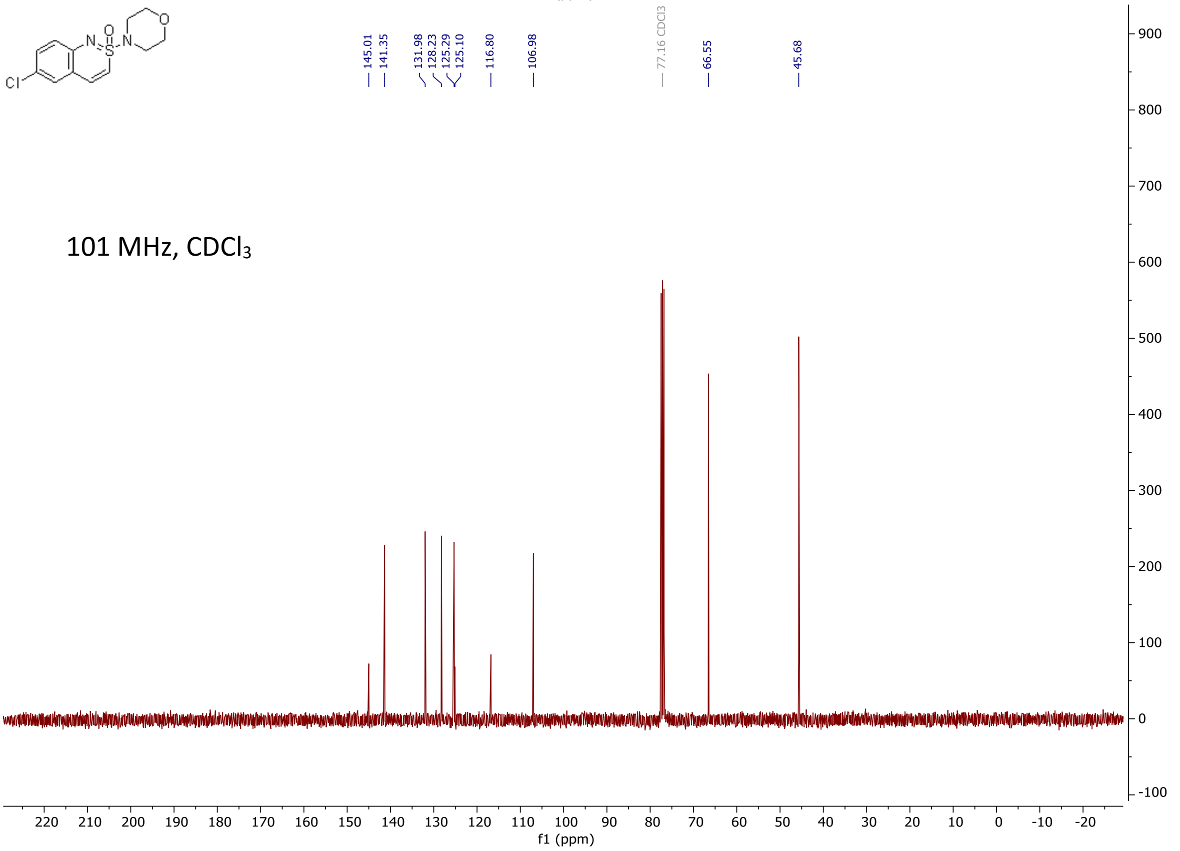

2-Morpholino-6-nitrobenzo[c][1,2]thiazine 2-oxide (3d)

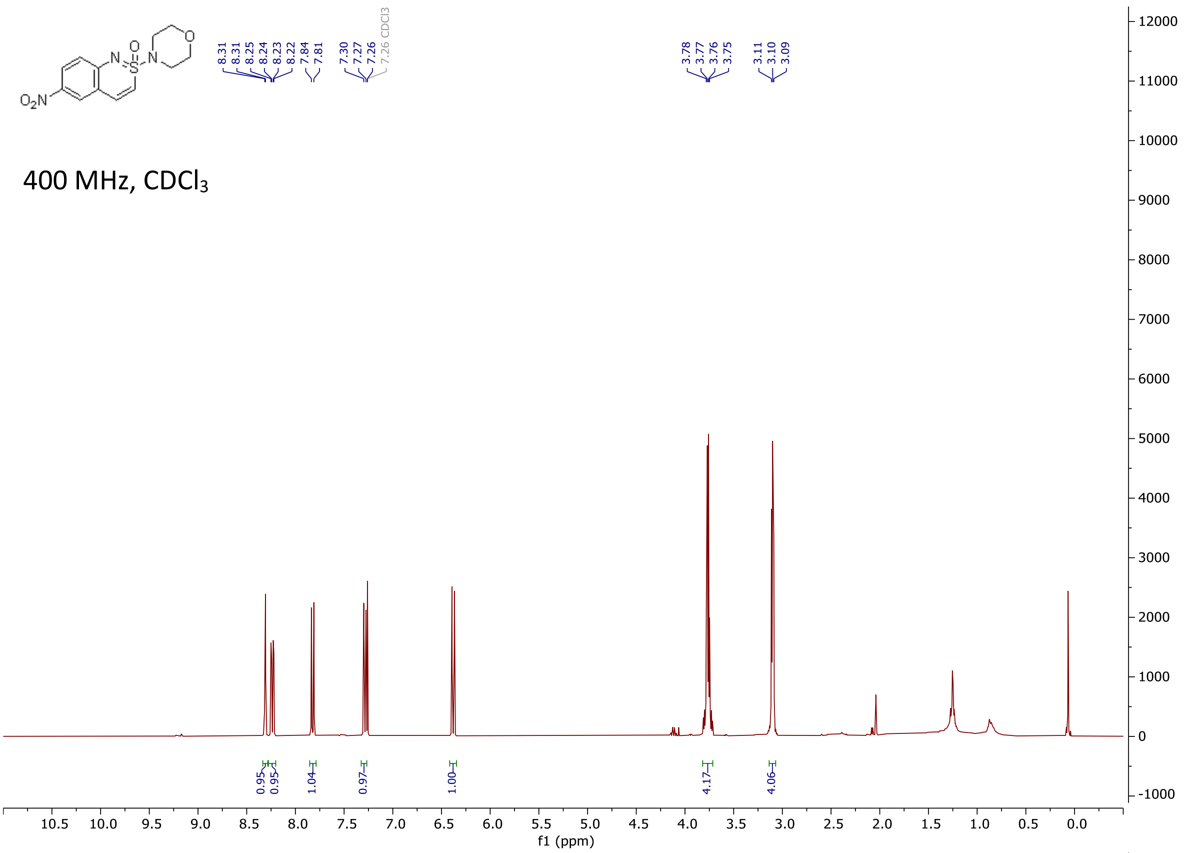

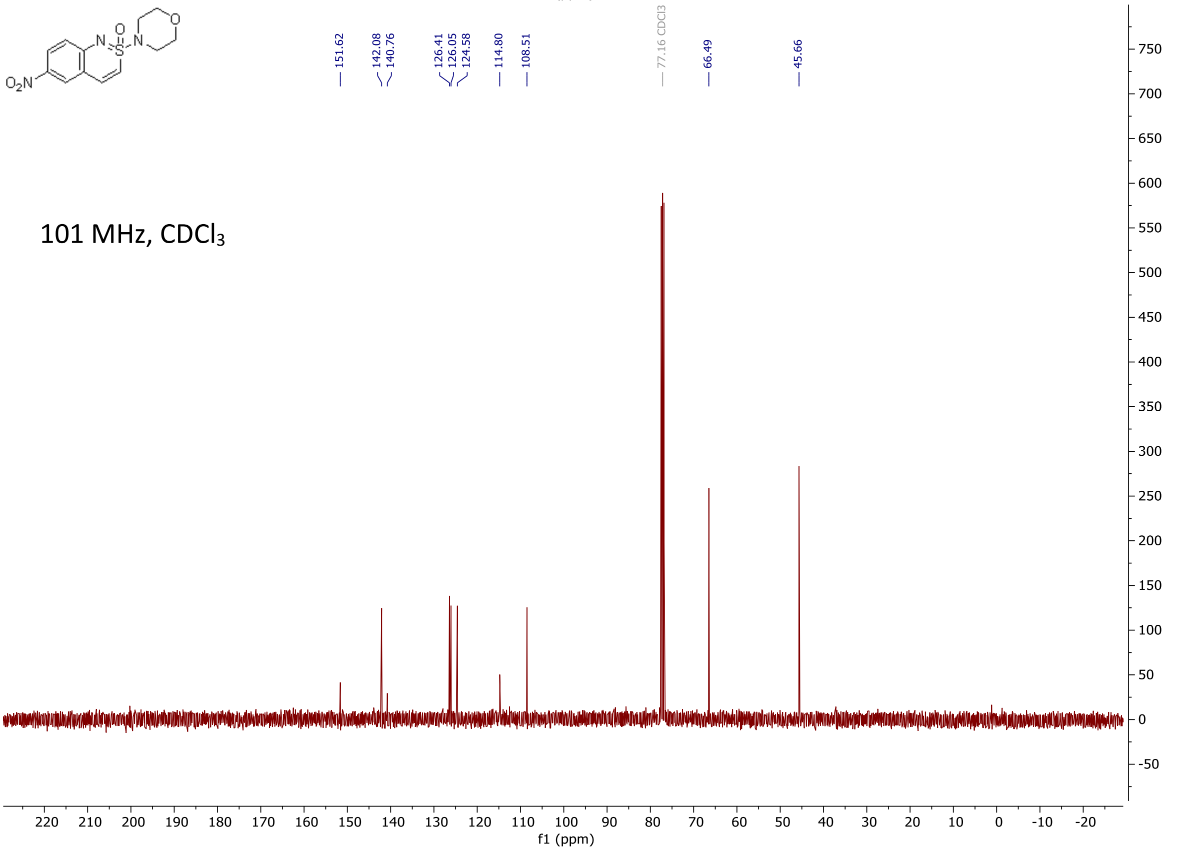

6-Fluoro-2-morpholinobenzo[c][1,2]thiazine 2-oxide (3e)

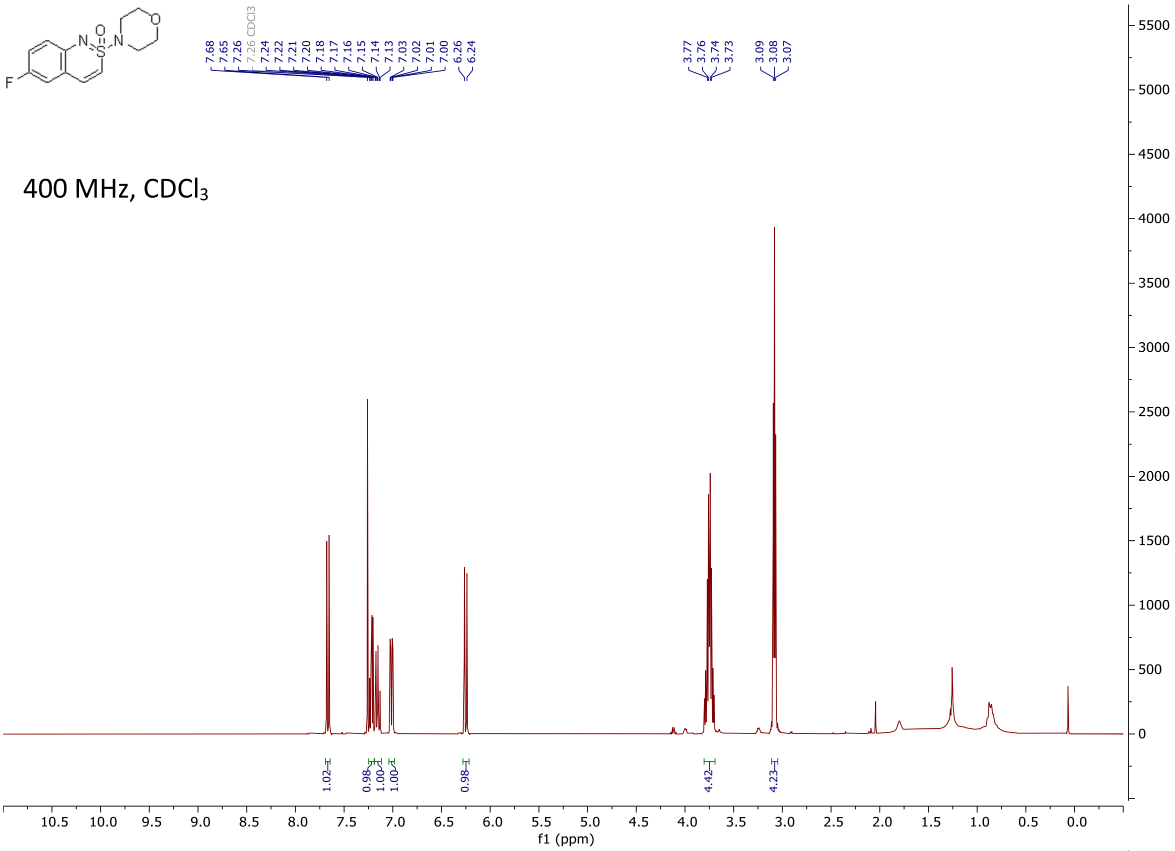

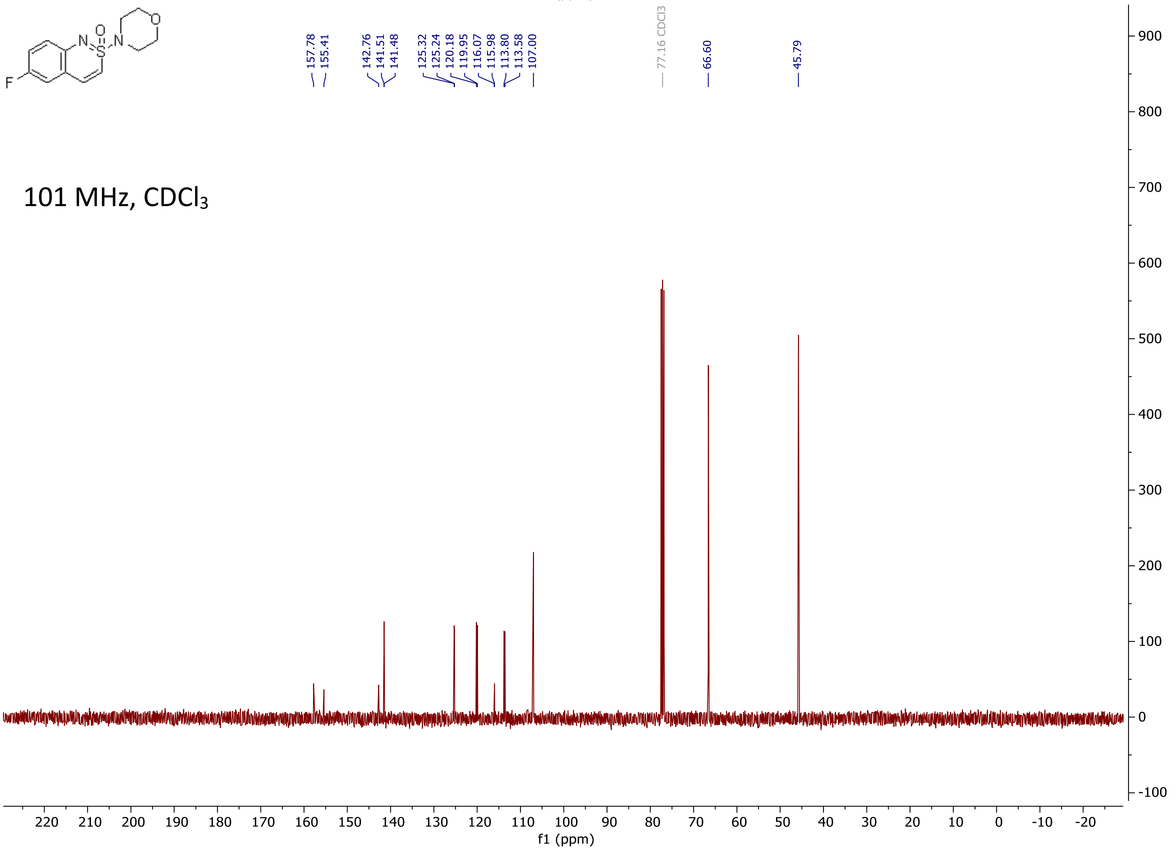

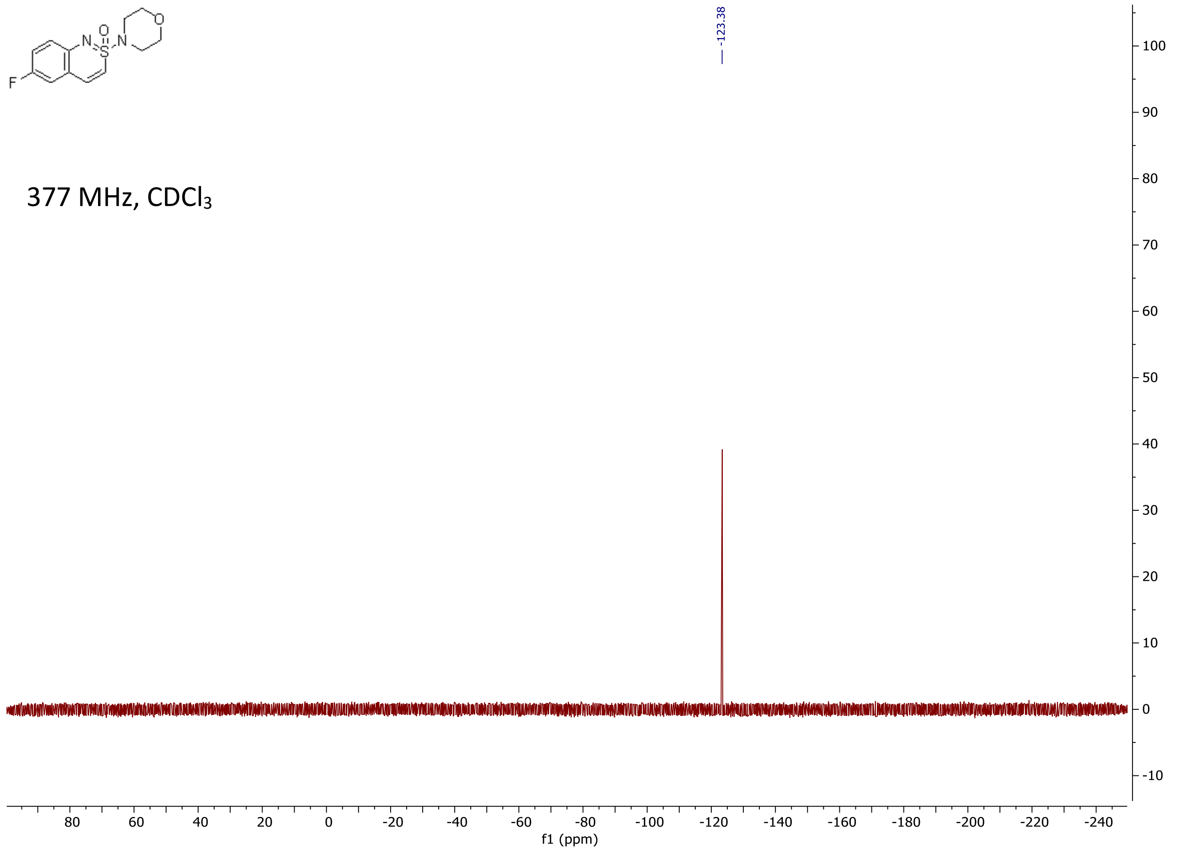

7-Fluoro-2-morpholinobenzo[c][1,2]thiazine 2-oxide (3f)

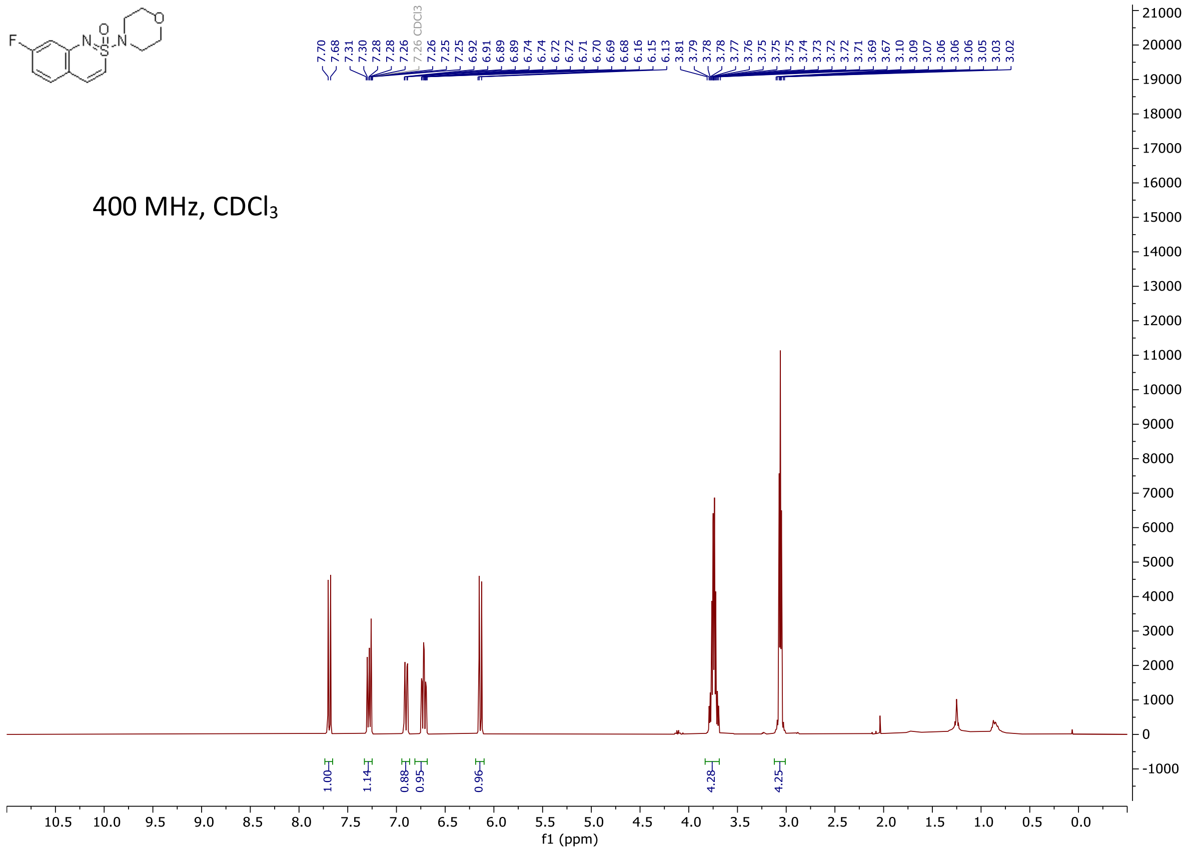

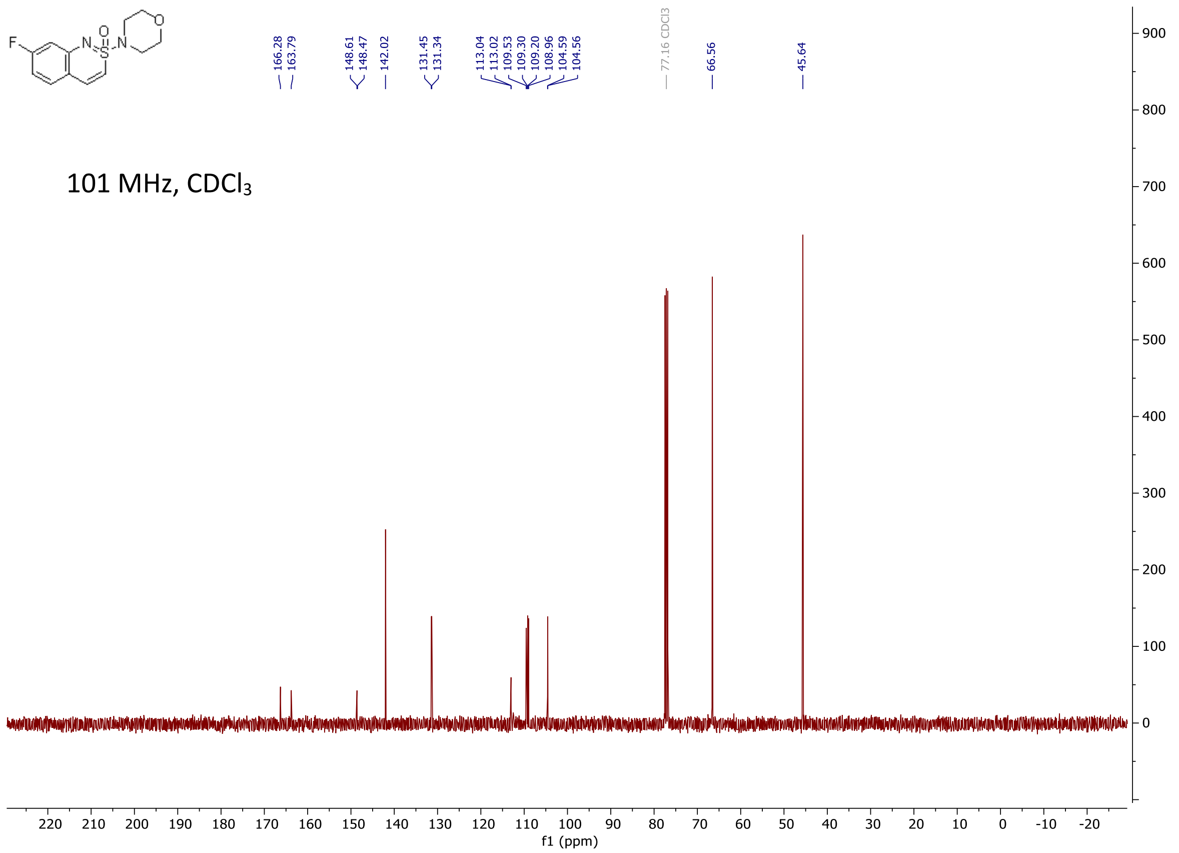
101 MHz, CDCl3
166.28
163.79
148.61
148.47
142.02
131.45
131.34
113.04
113.02
109.53
109.30
109.20
108.96
104.59
104.56
77.16 CDCl3
66.56
45.64
f1 (ppm)

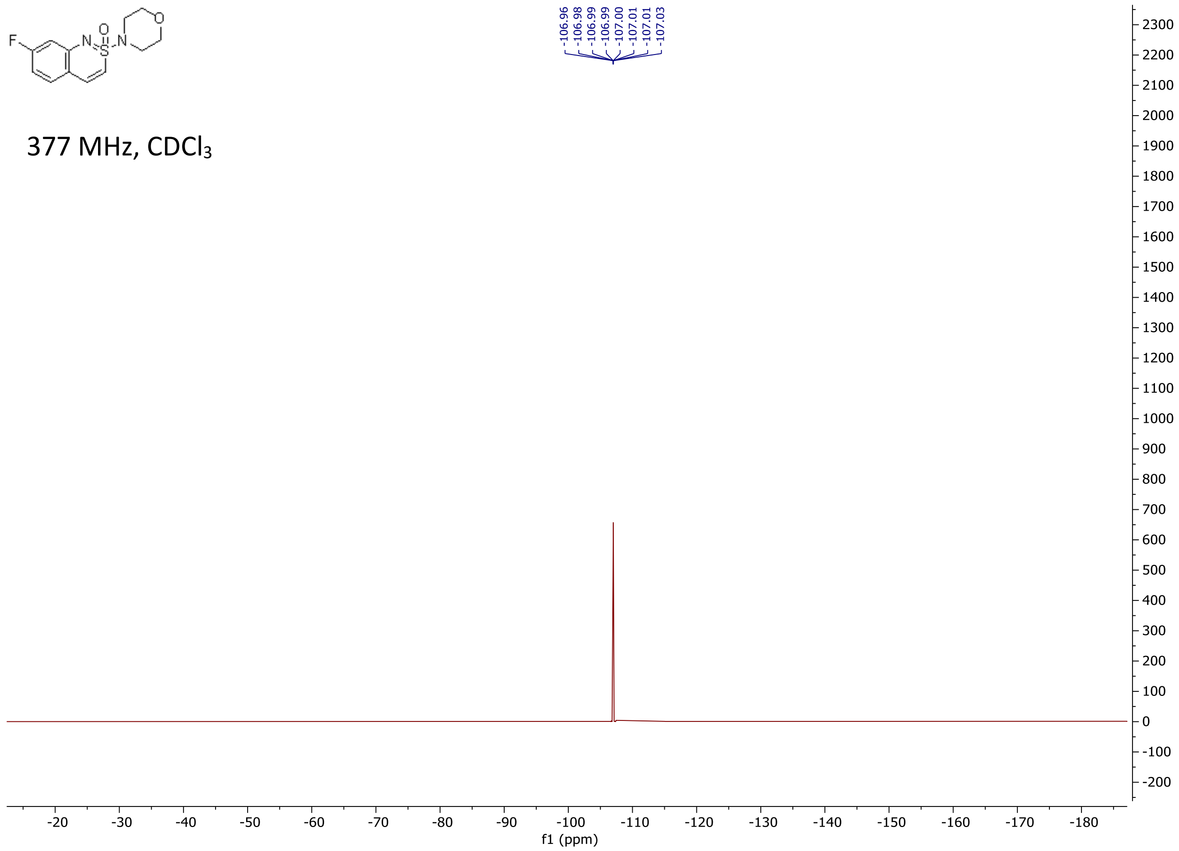
377 MHz, CDCl3
-106.96
-106.98
-106.99
-106.99
-107.00
-107.01
-107.01
-107.03
f1 (ppm)

5-Fluoro-2-morpholinobenzo[c][1,2]thiazine 2-oxide (3g)

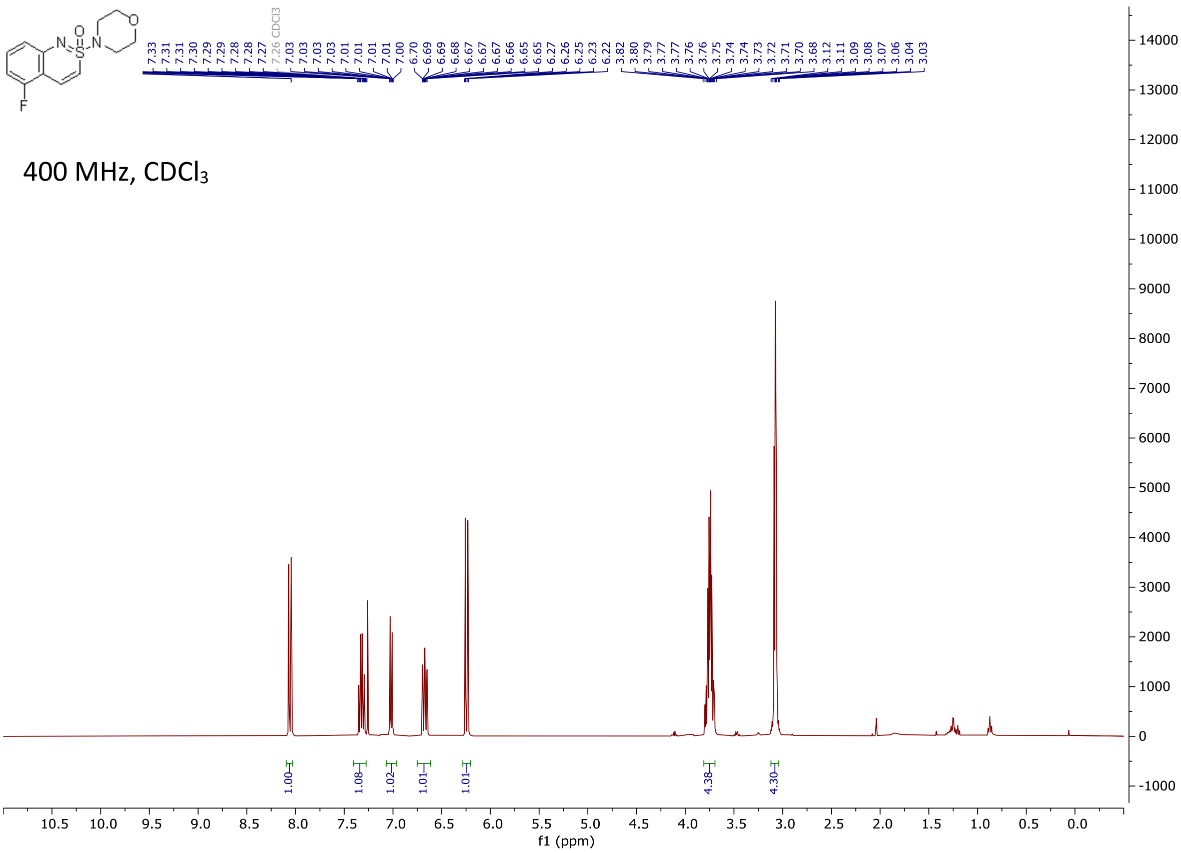

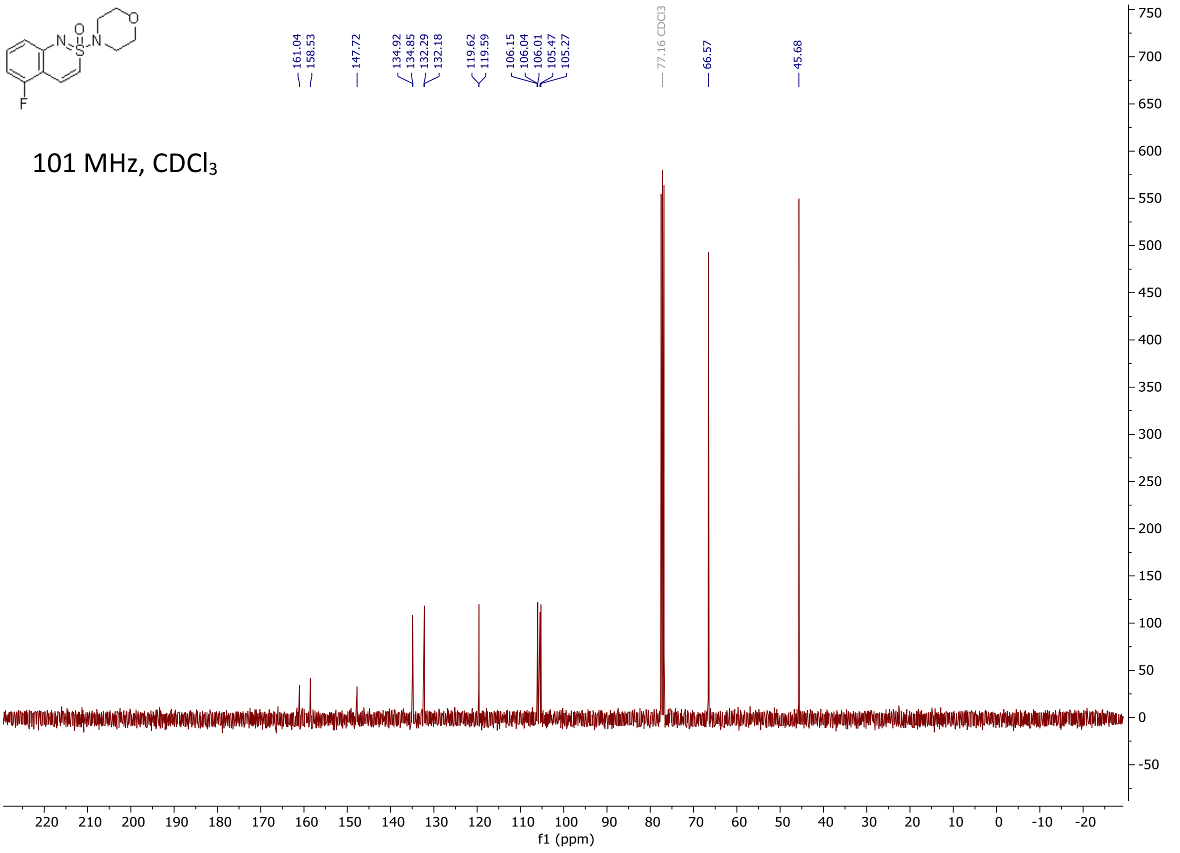

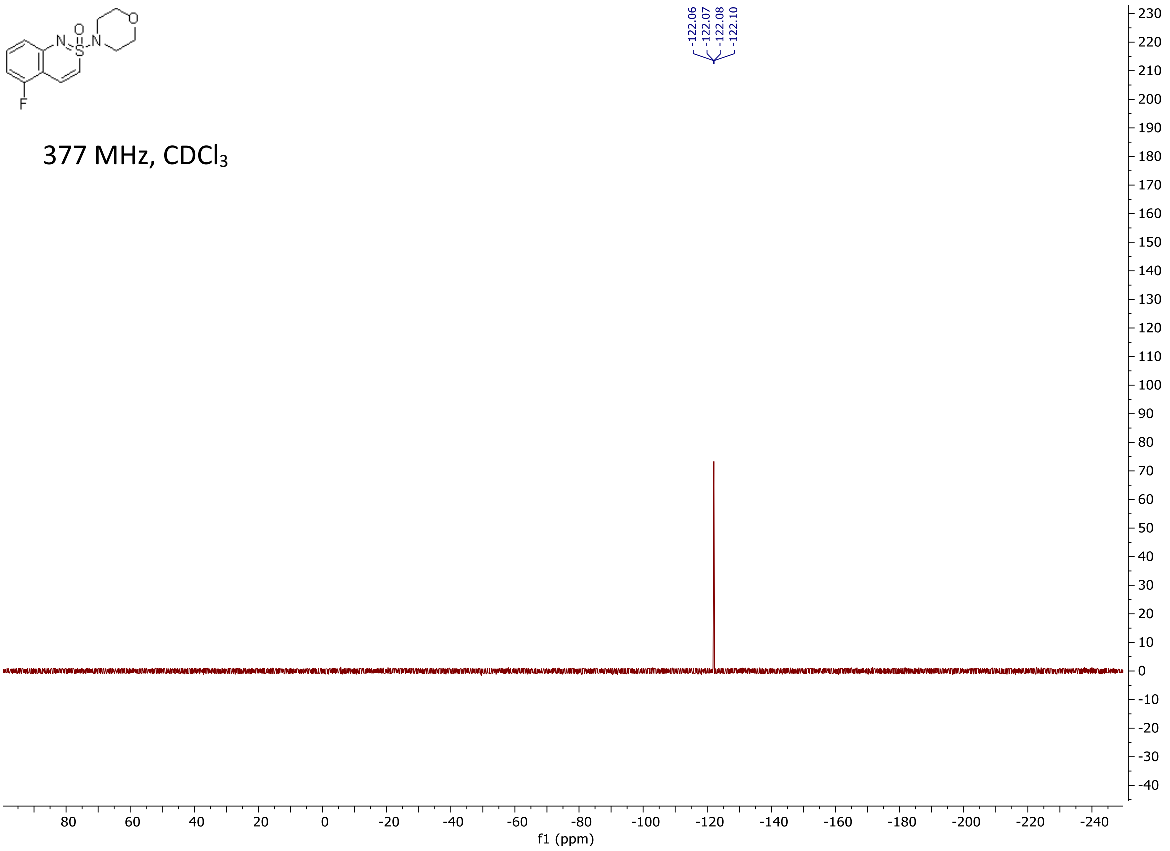

8-methyl-2-morpholinobenzo[*c*][1,2]thiazine 2-oxide (3h)

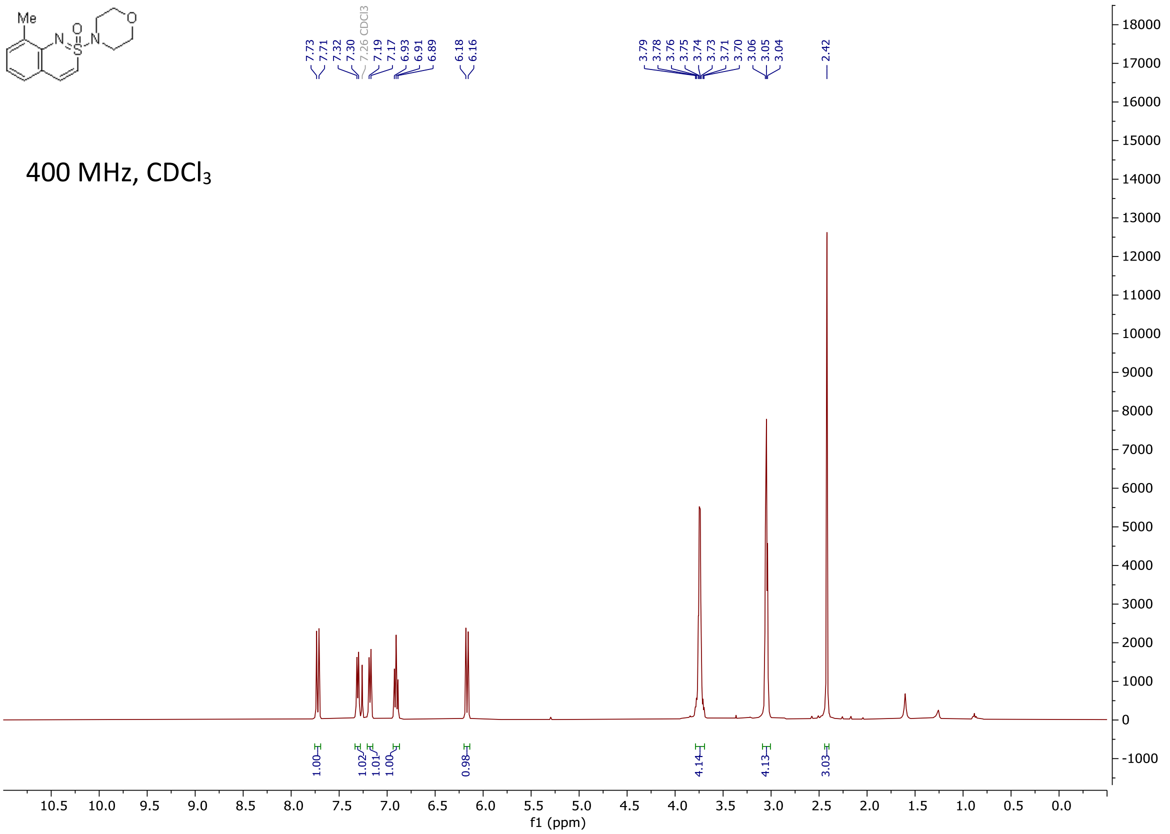

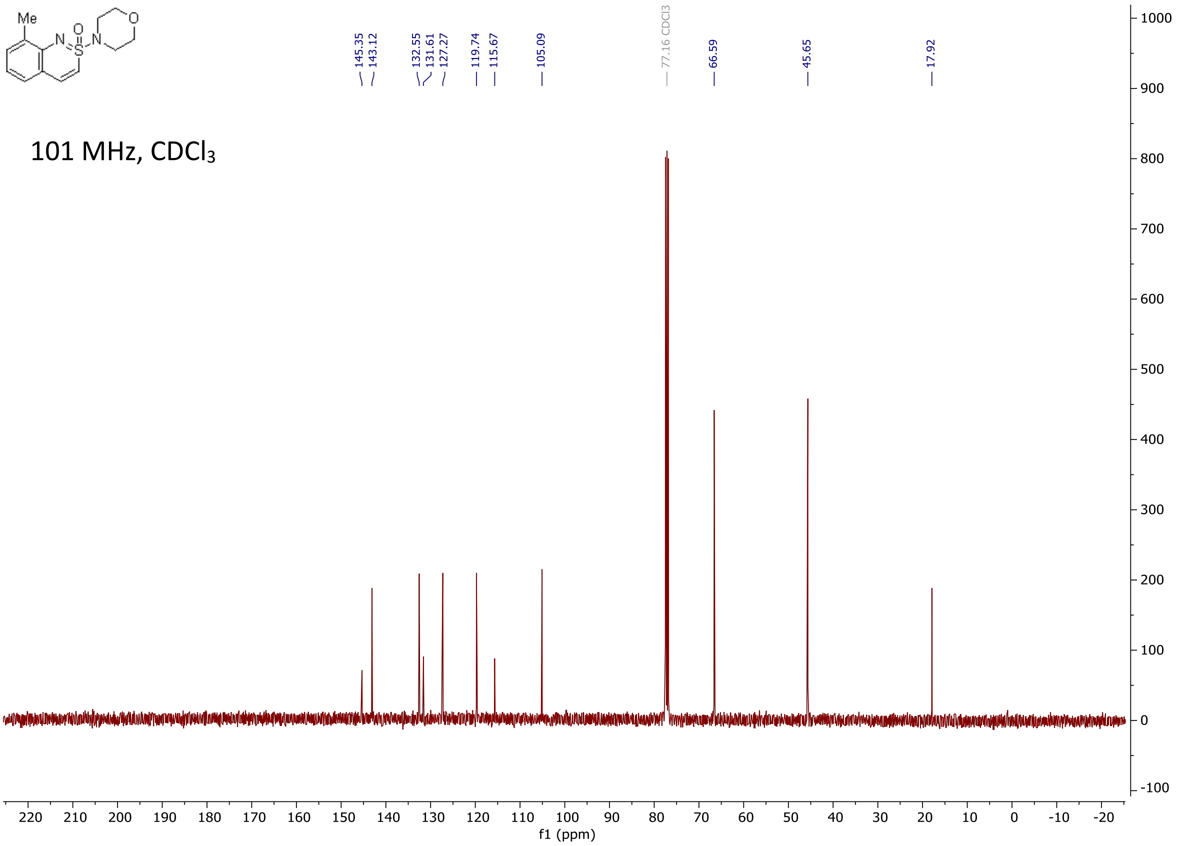

2-Morpholino-[1,3]dioxolo[4',5':4,5]benzo[1,2-*c*][1,2]thiazine 2-oxide (3i)

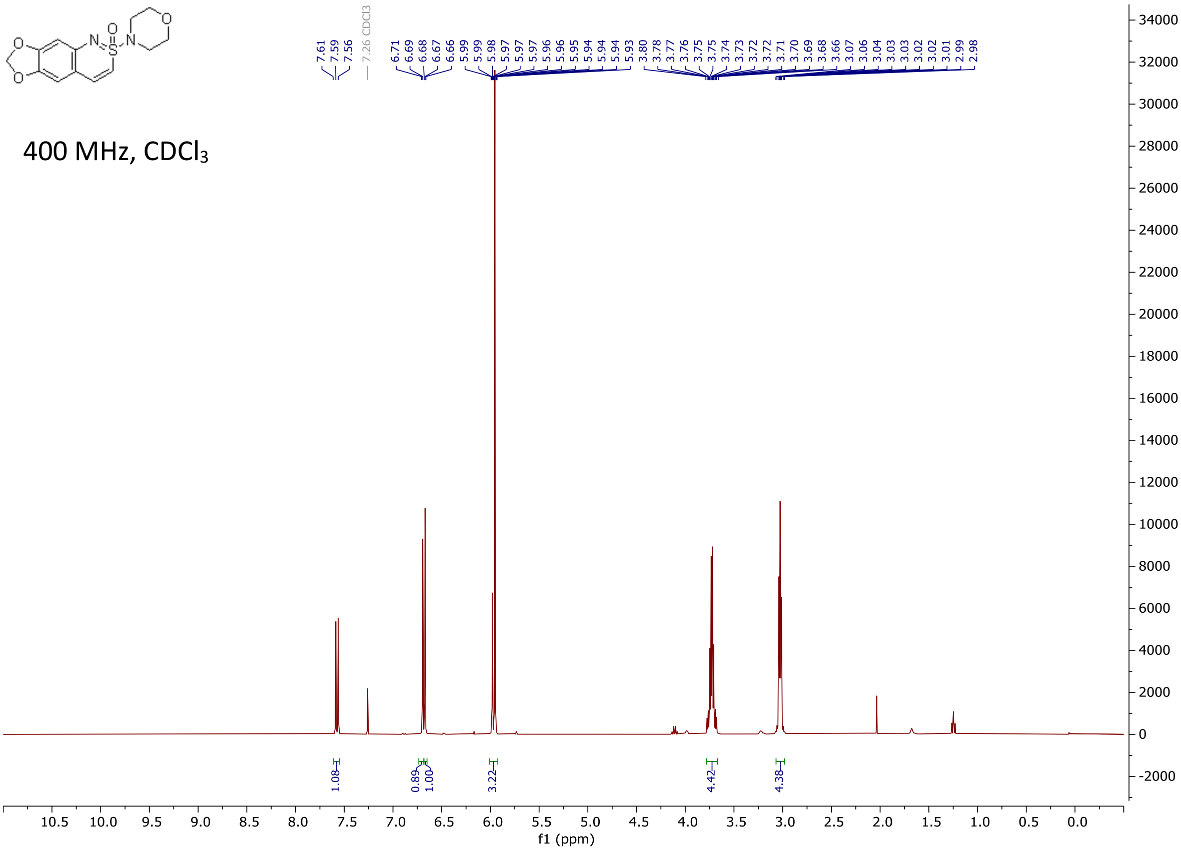

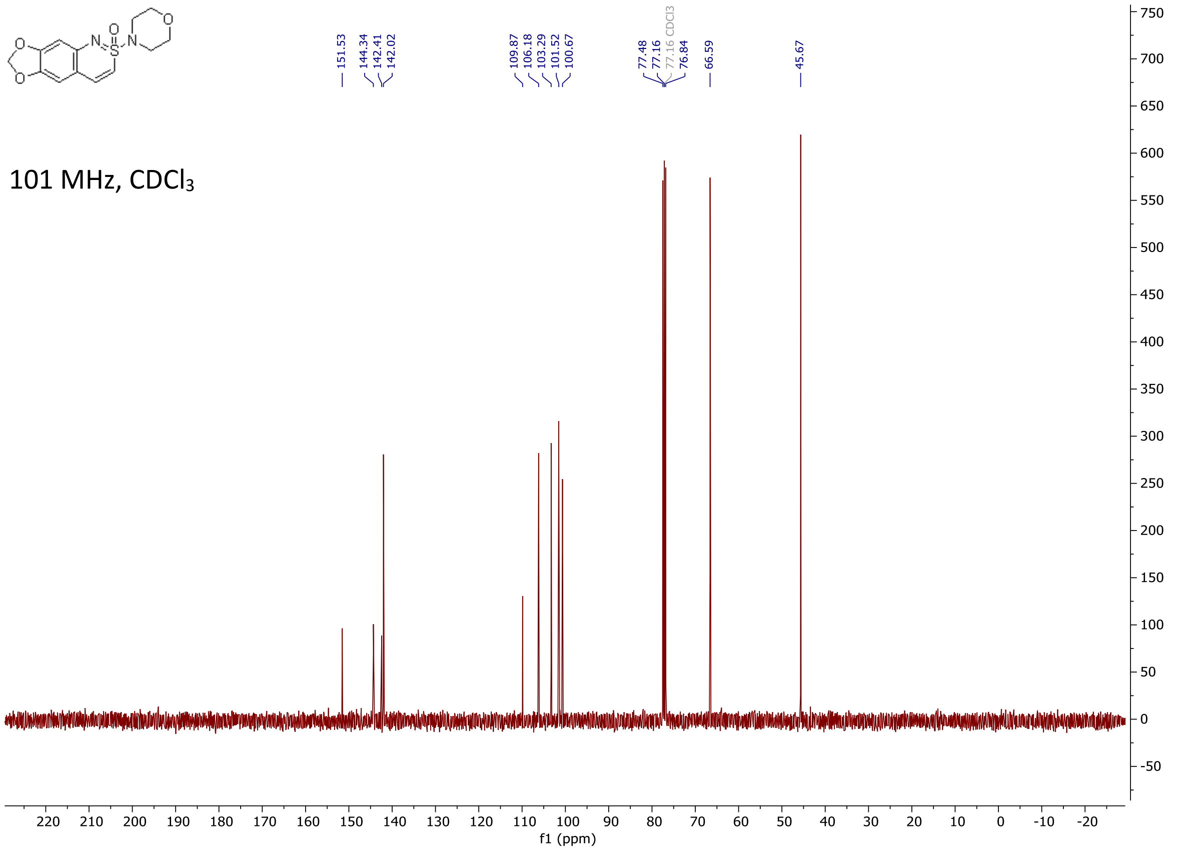

2-Morpholinopyrido[3,4-c][1,2]thiazine 2-oxide (3j)

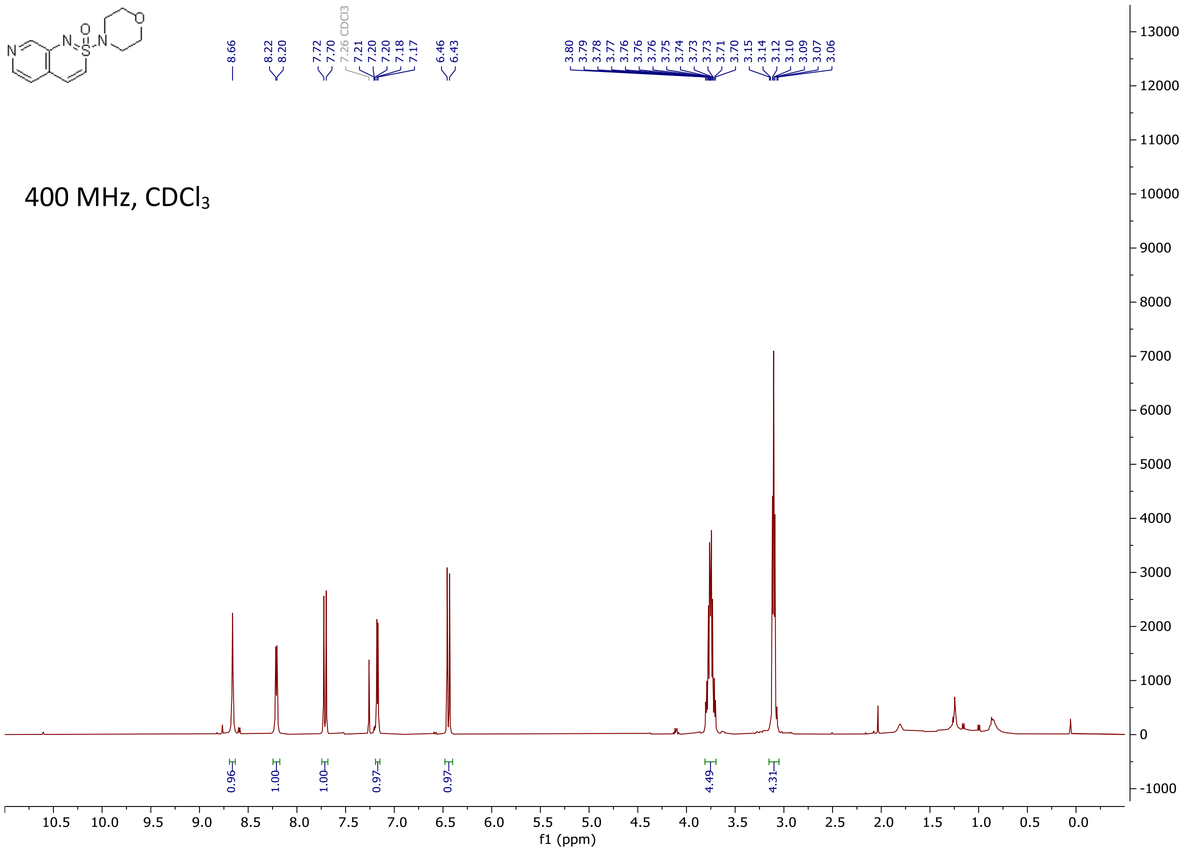

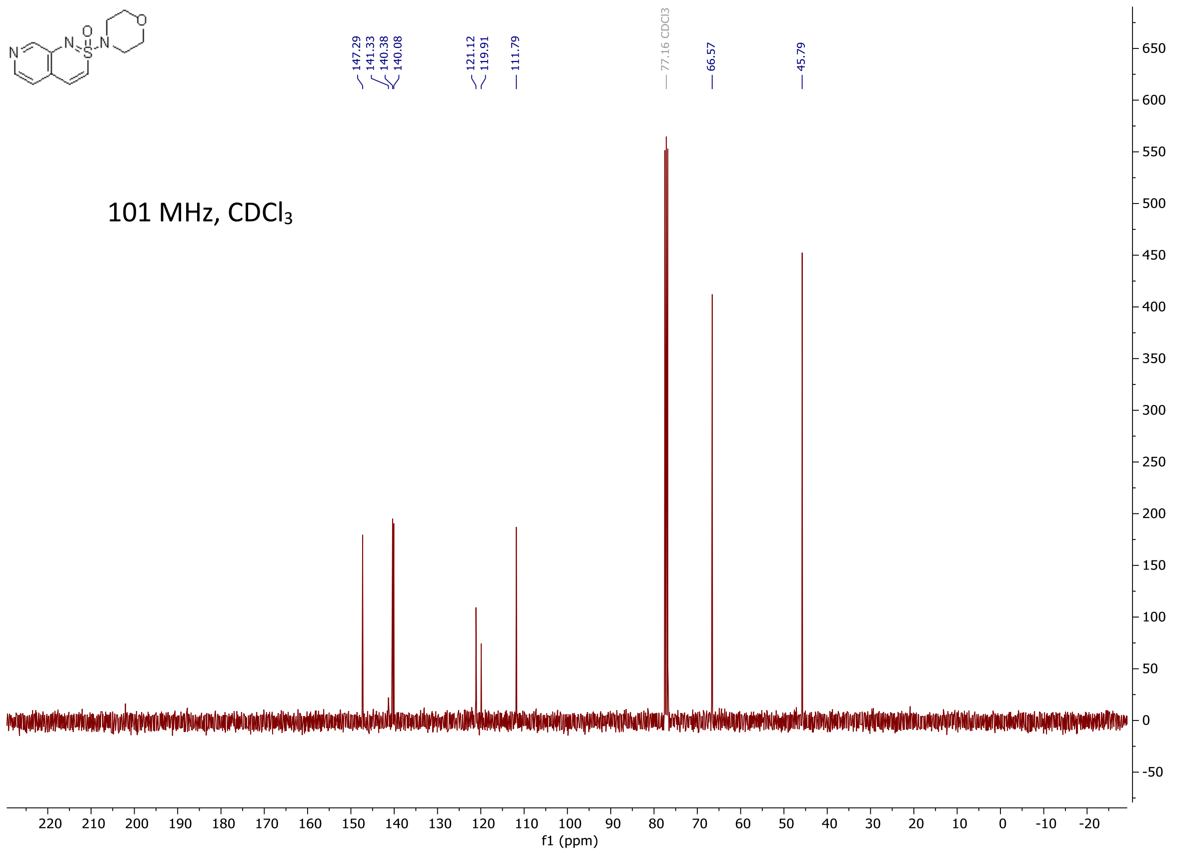

2-Morpholinopyrido[3,2-*c*][1,2]thiazine 2-oxide (3k)

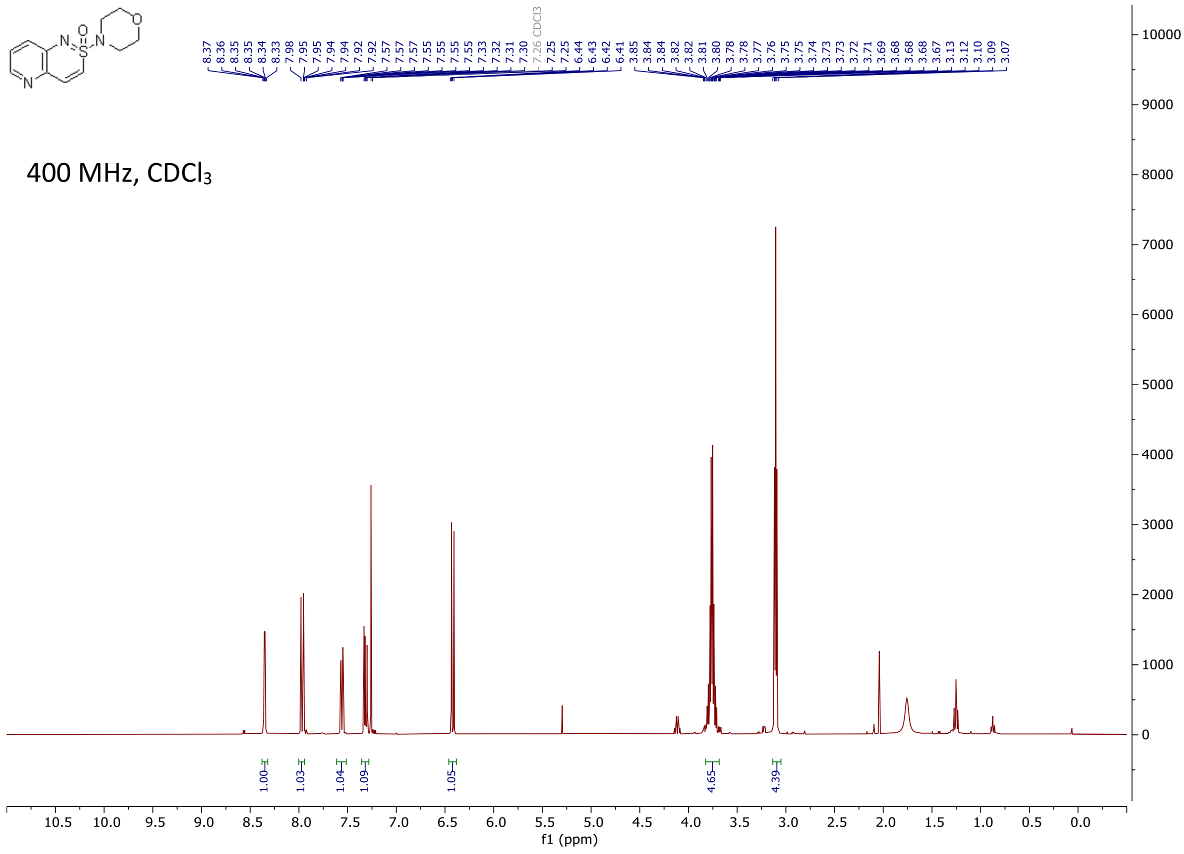

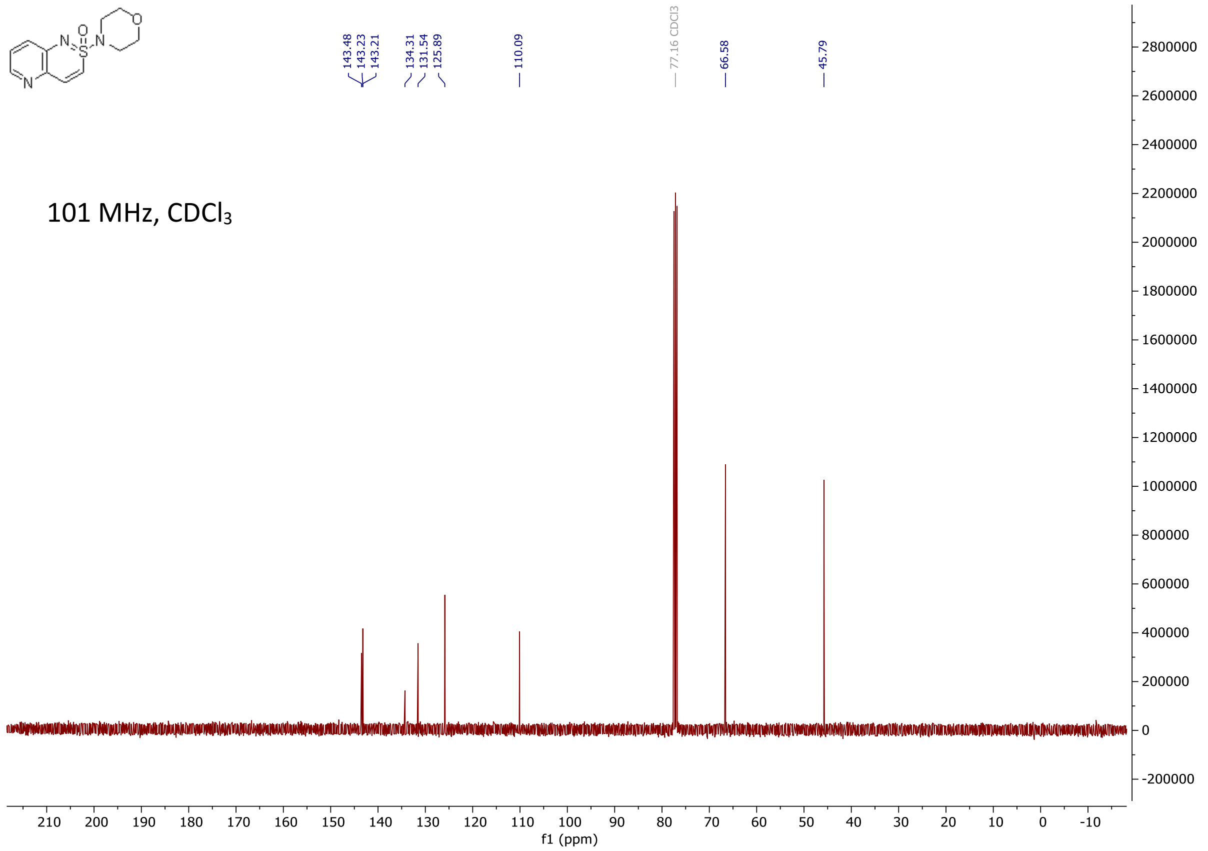

2-Morpholinothieno[3,2-c][1,2]thiazine 2-oxide (3l)

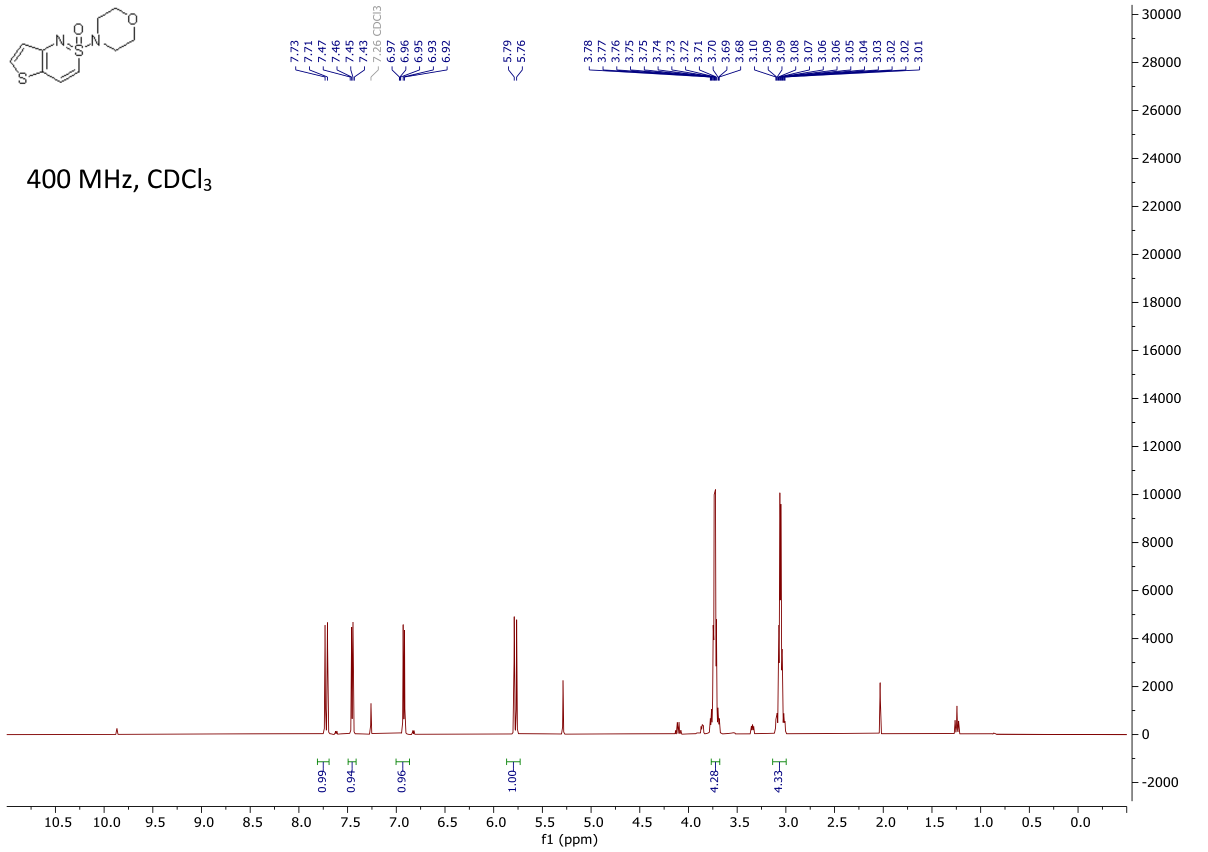

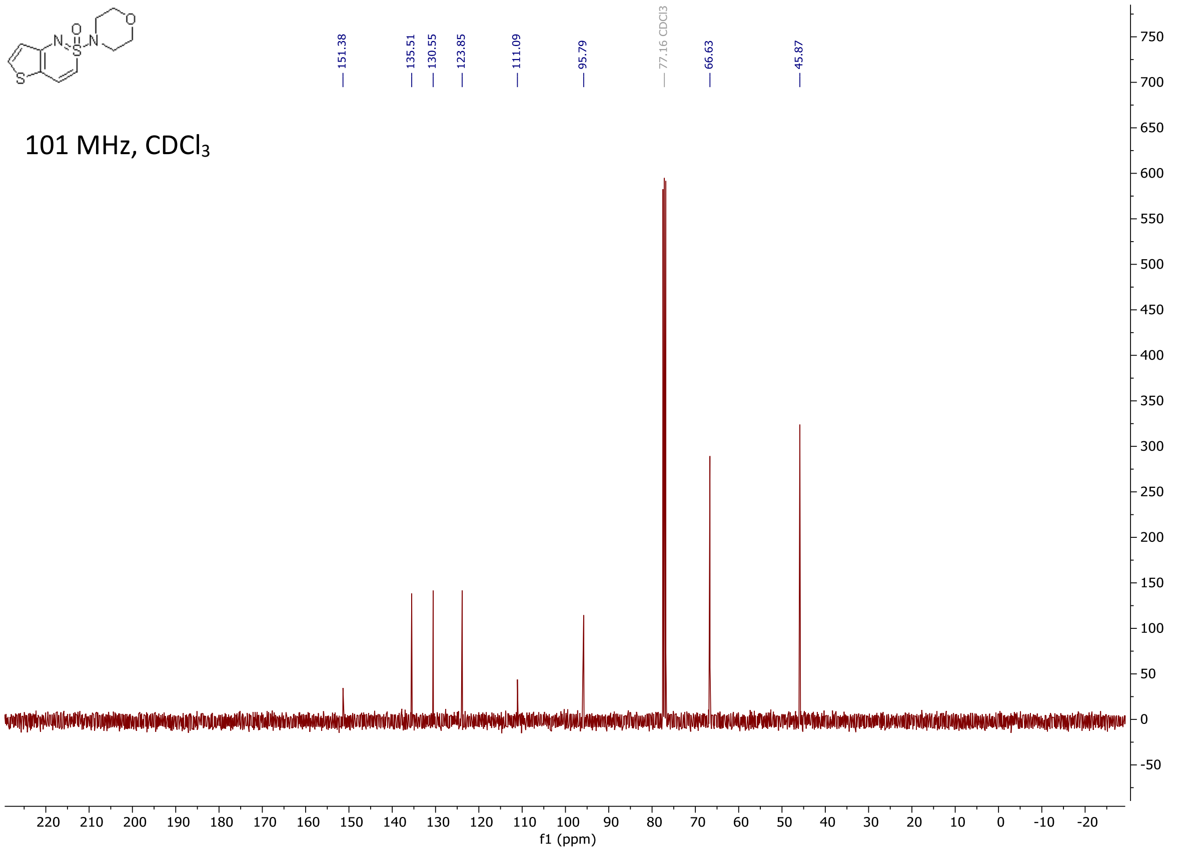

2-Morpholino-4-phenylbenzo[c][1,2]thiazine 2-oxide (3m)

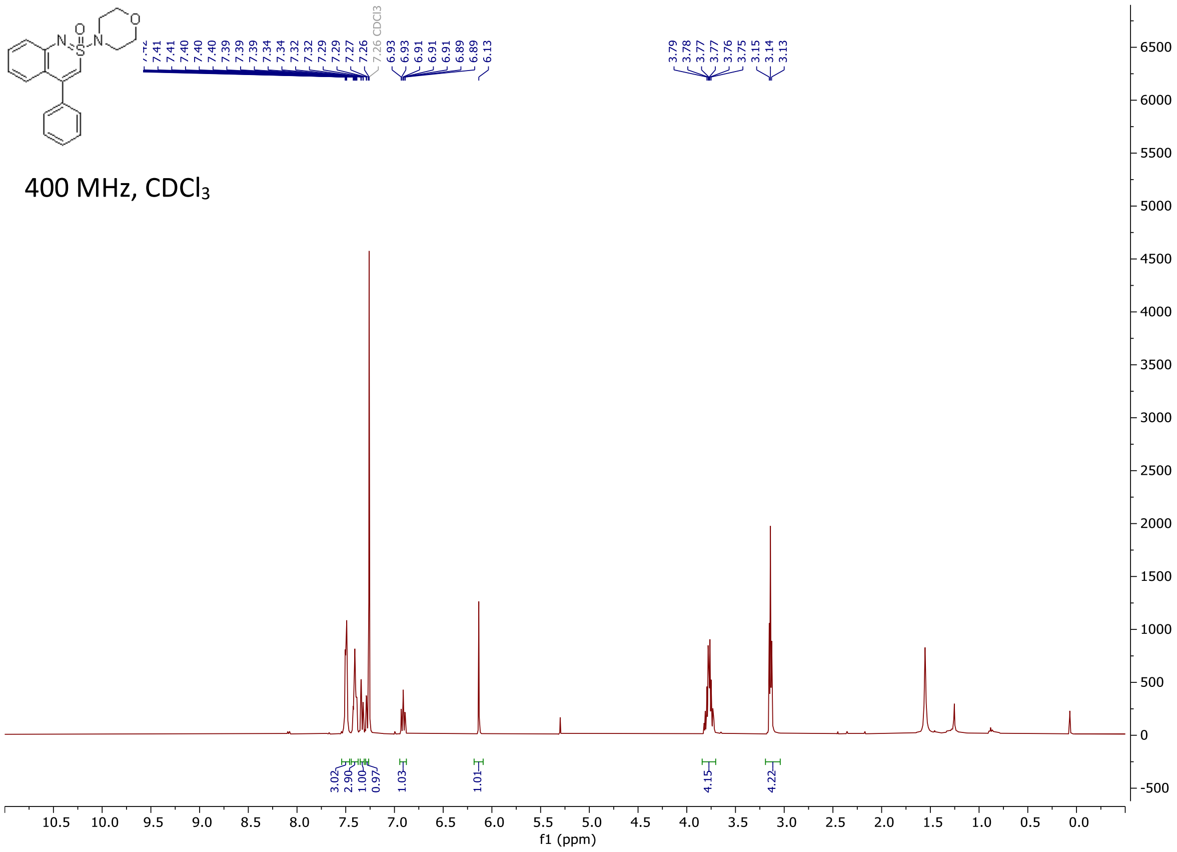

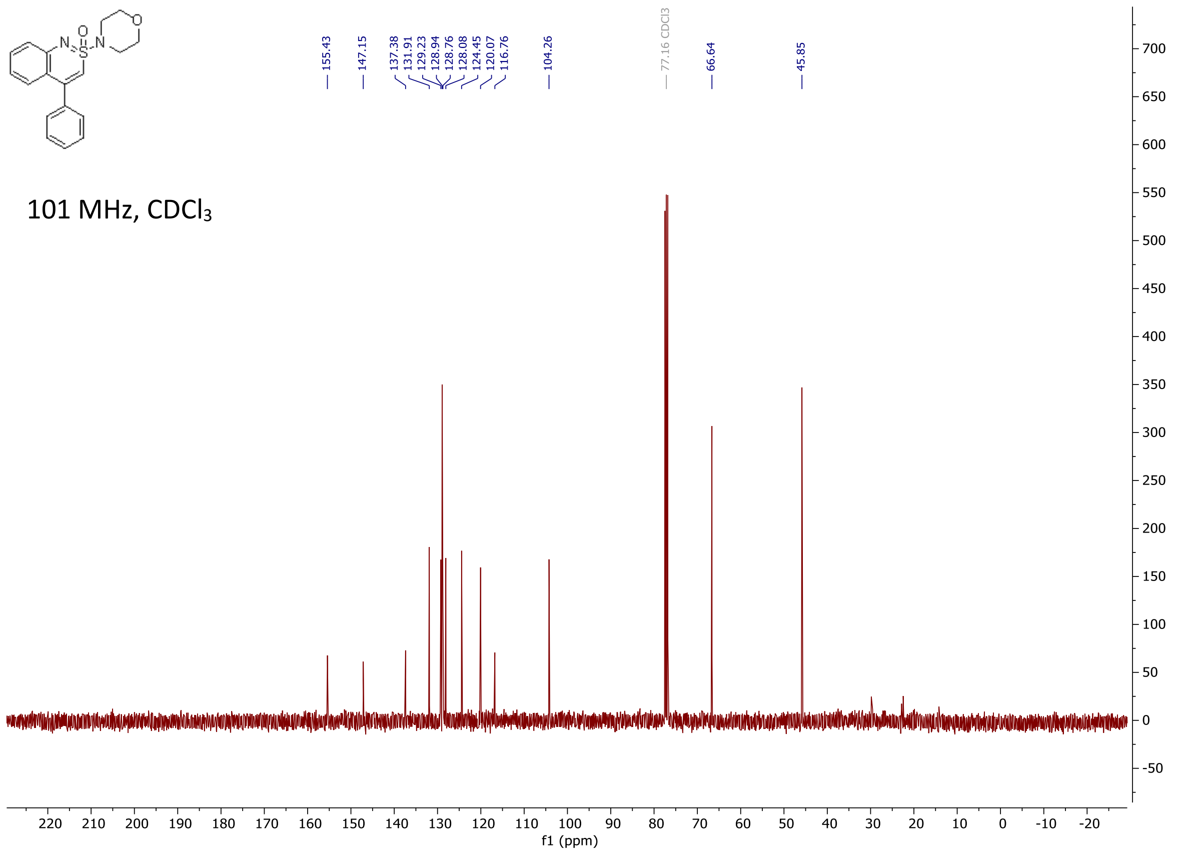

2-(1,4-dioxa-8-azaspiro[4.5]decan-8-yl)benzo[c][1,2]thiazine 2-oxide (3o)

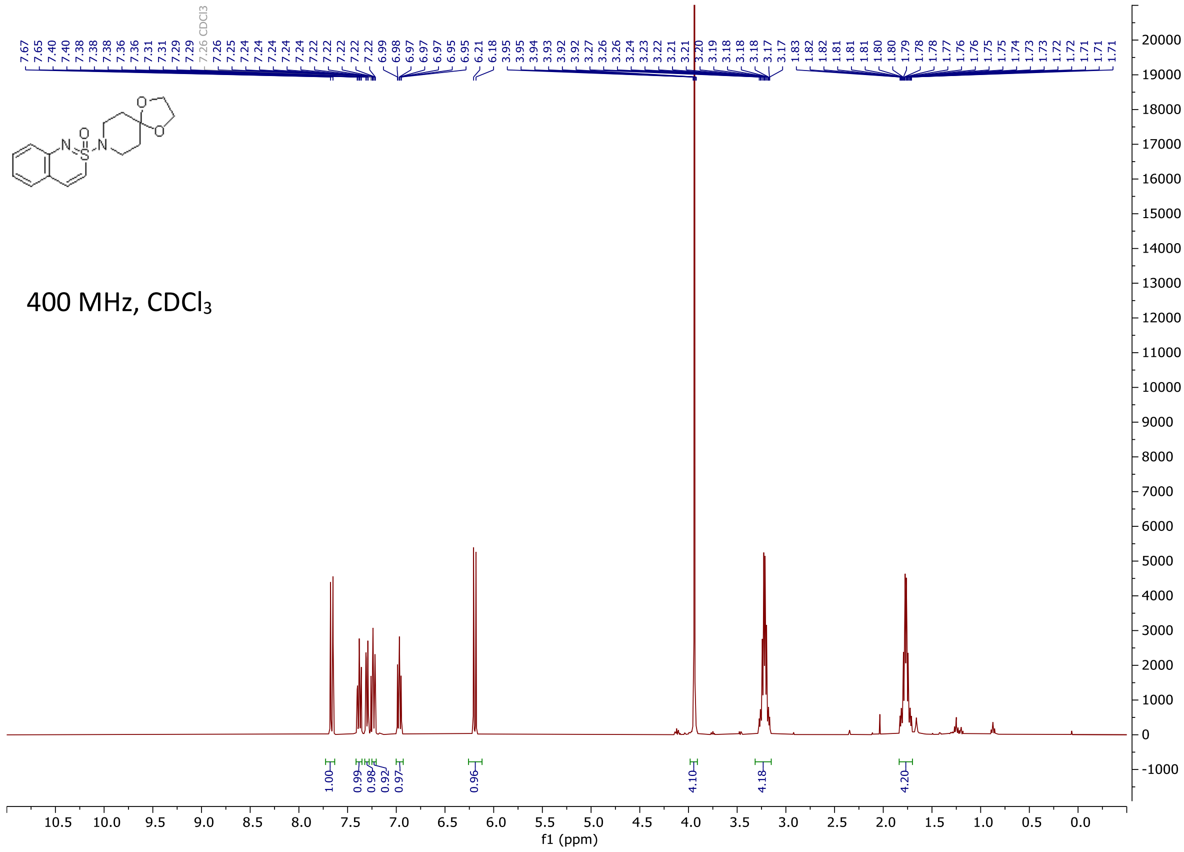

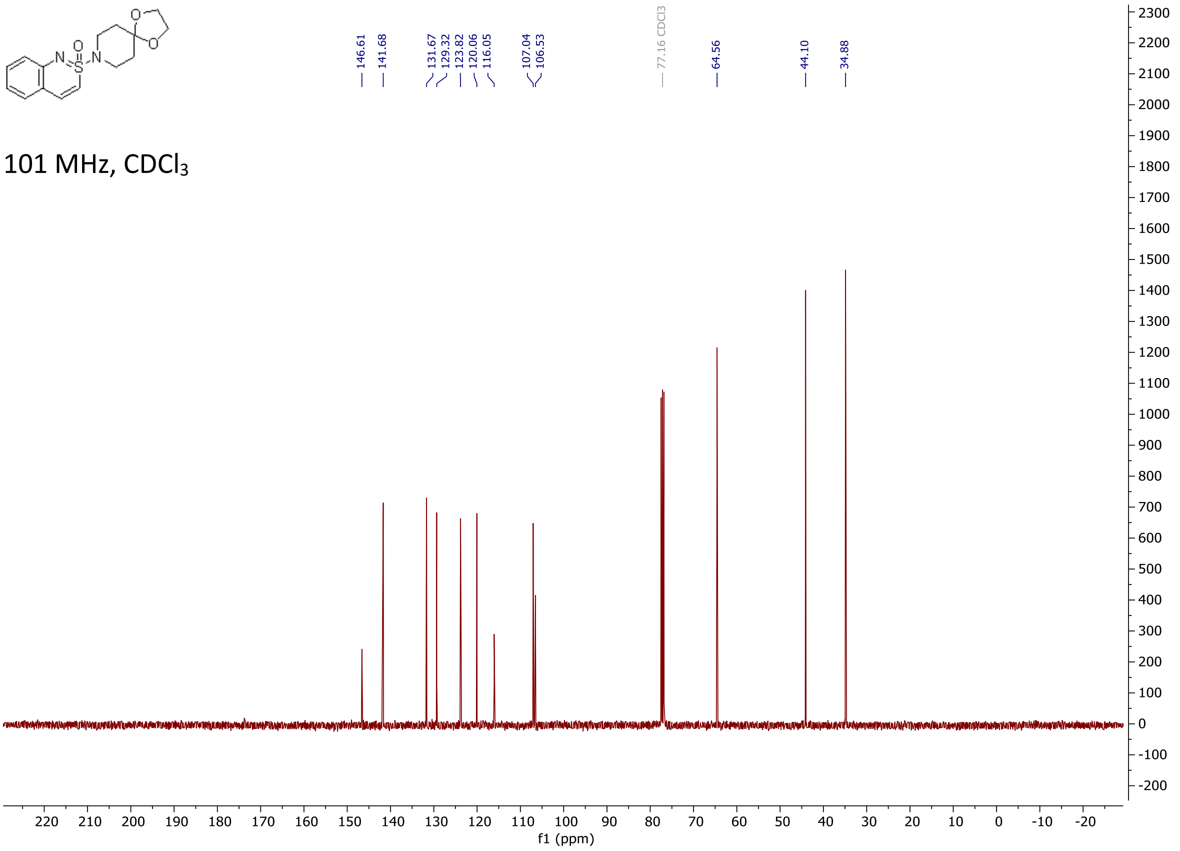

2-(4-(pyrimidin-2-yl)piperazin-1-yl)benzo[c][1,2]thiazine 2-oxide (3p)

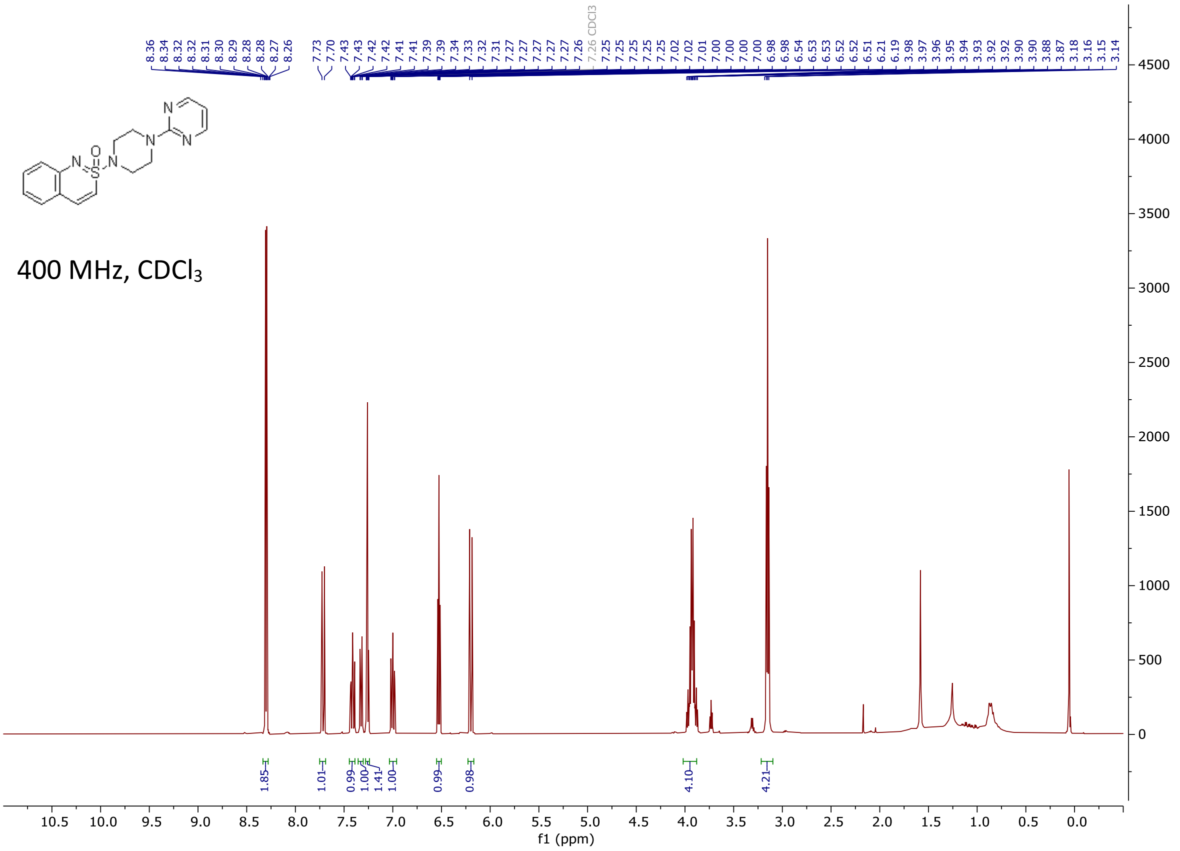

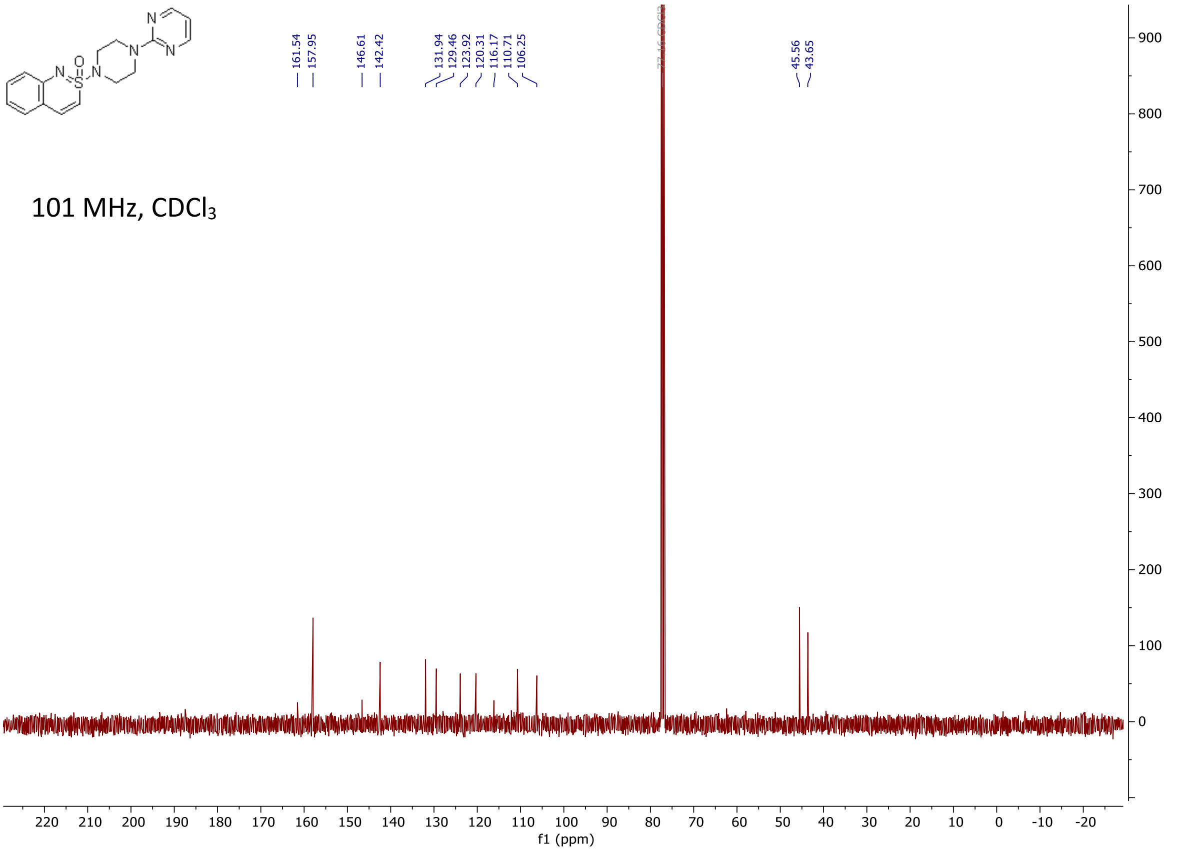

2-(6,7-dihydrothieno[3,2-c]pyridin-5(4H)-yl)benzo[c][1,2]thiazine 2-oxide (3q)

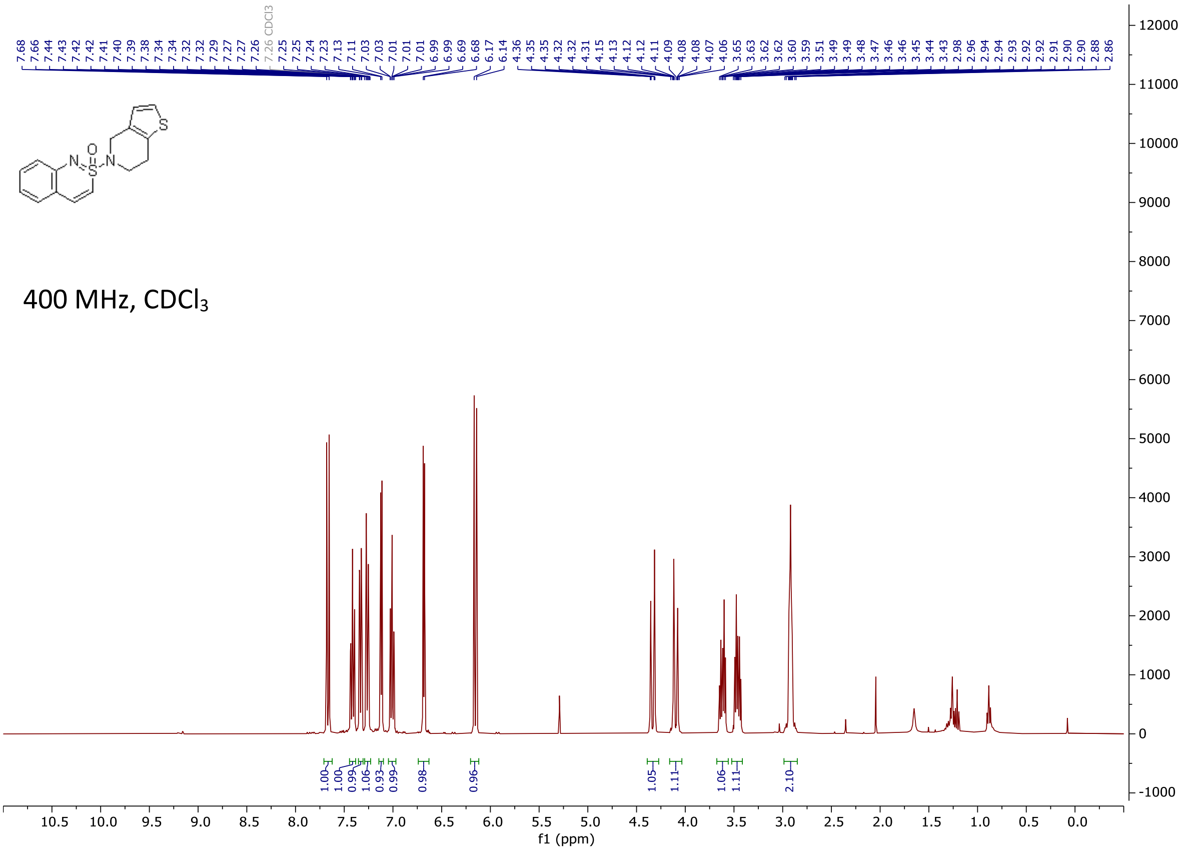

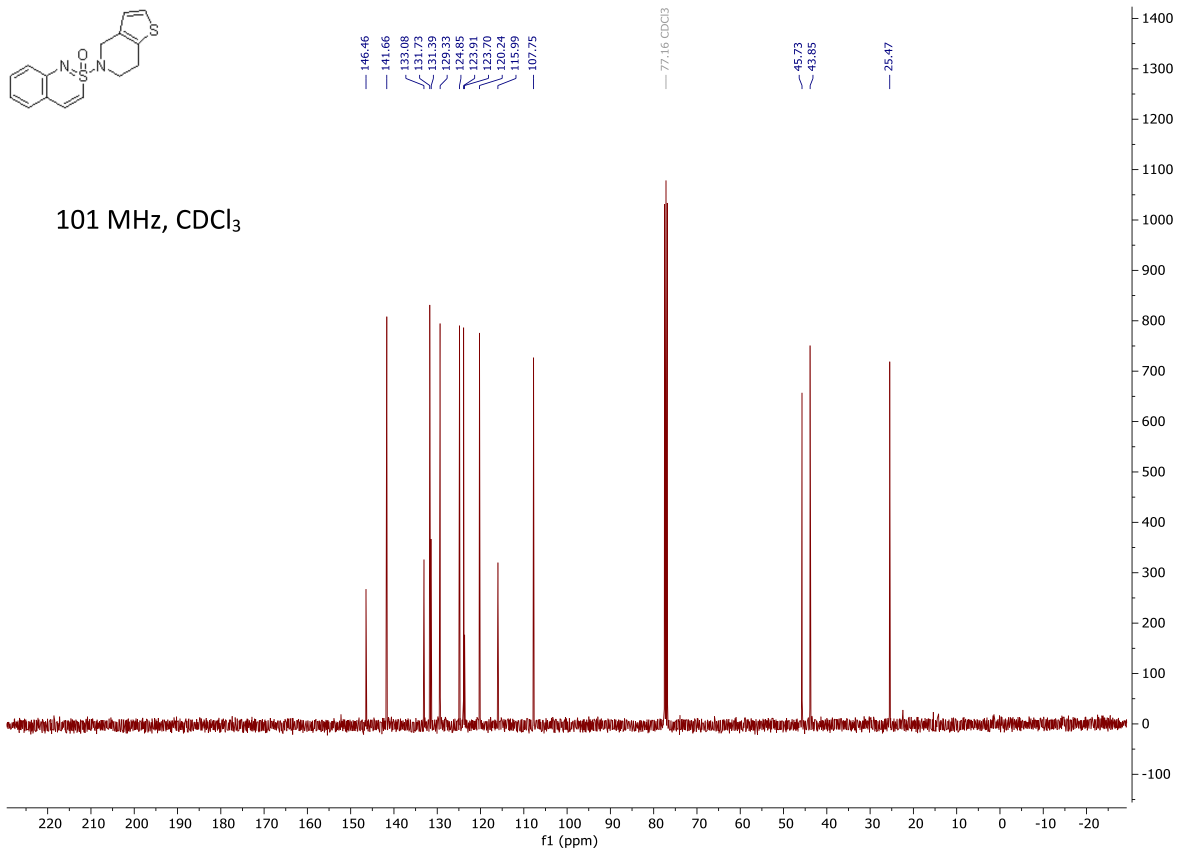

2-((3,4-dimethoxybenzyl)(methyl)amino)-2l4-benzo[c][1,2]thiazine 2-oxide (3r)

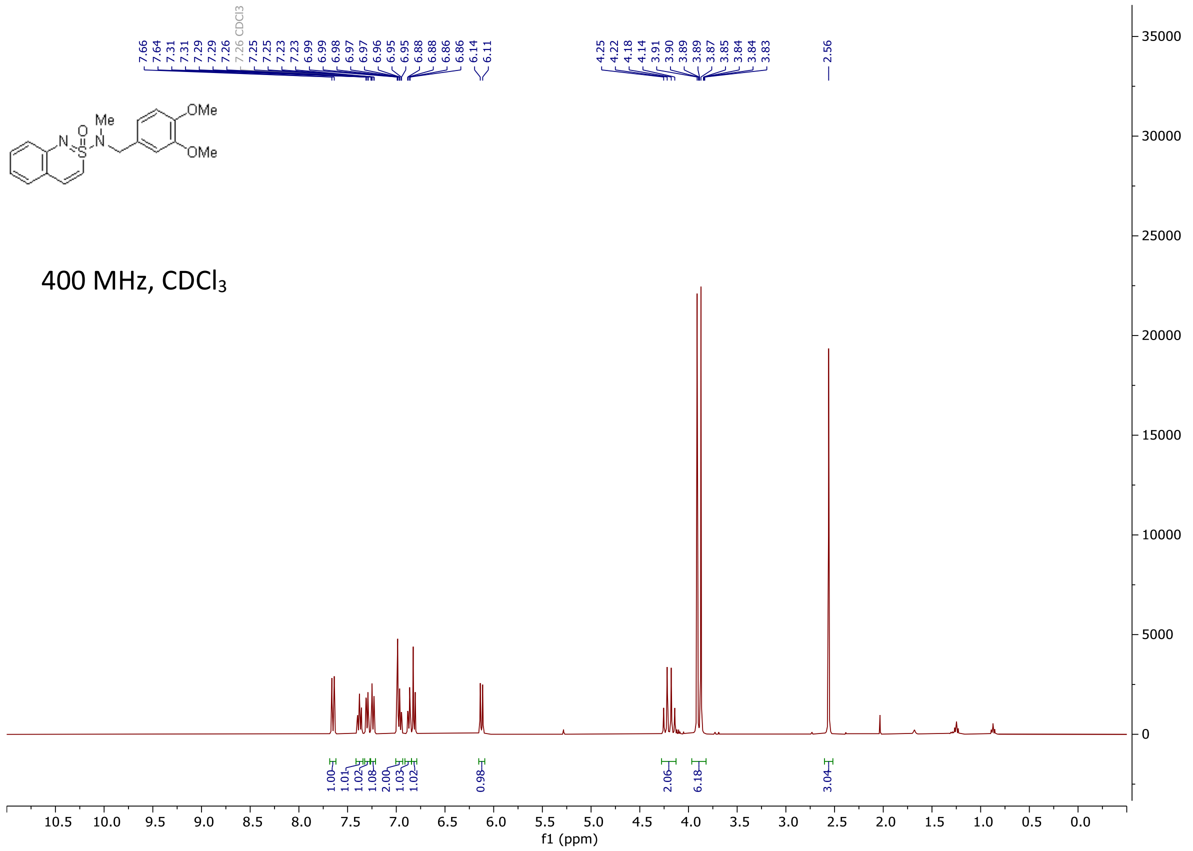

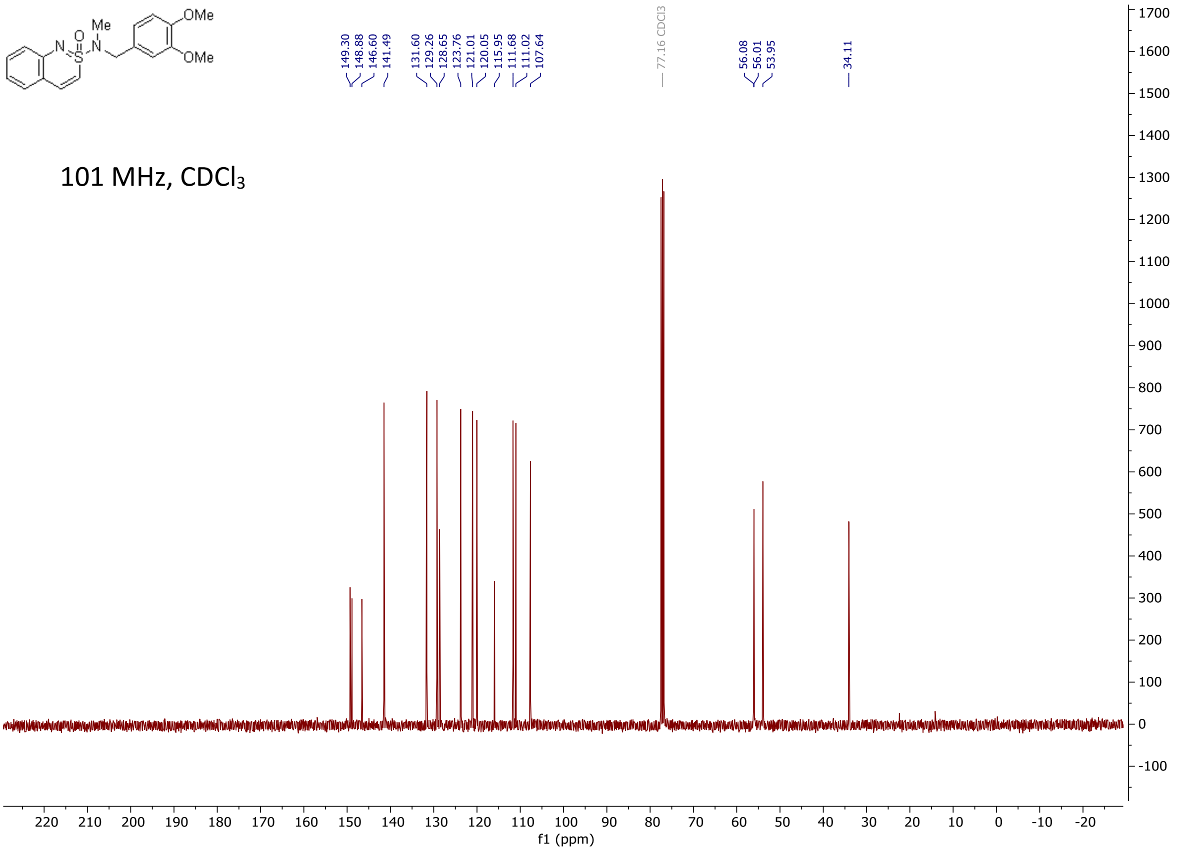

3-methyl-2-morpholinobenzo[c][1,2]thiazine 2-oxide (3t)

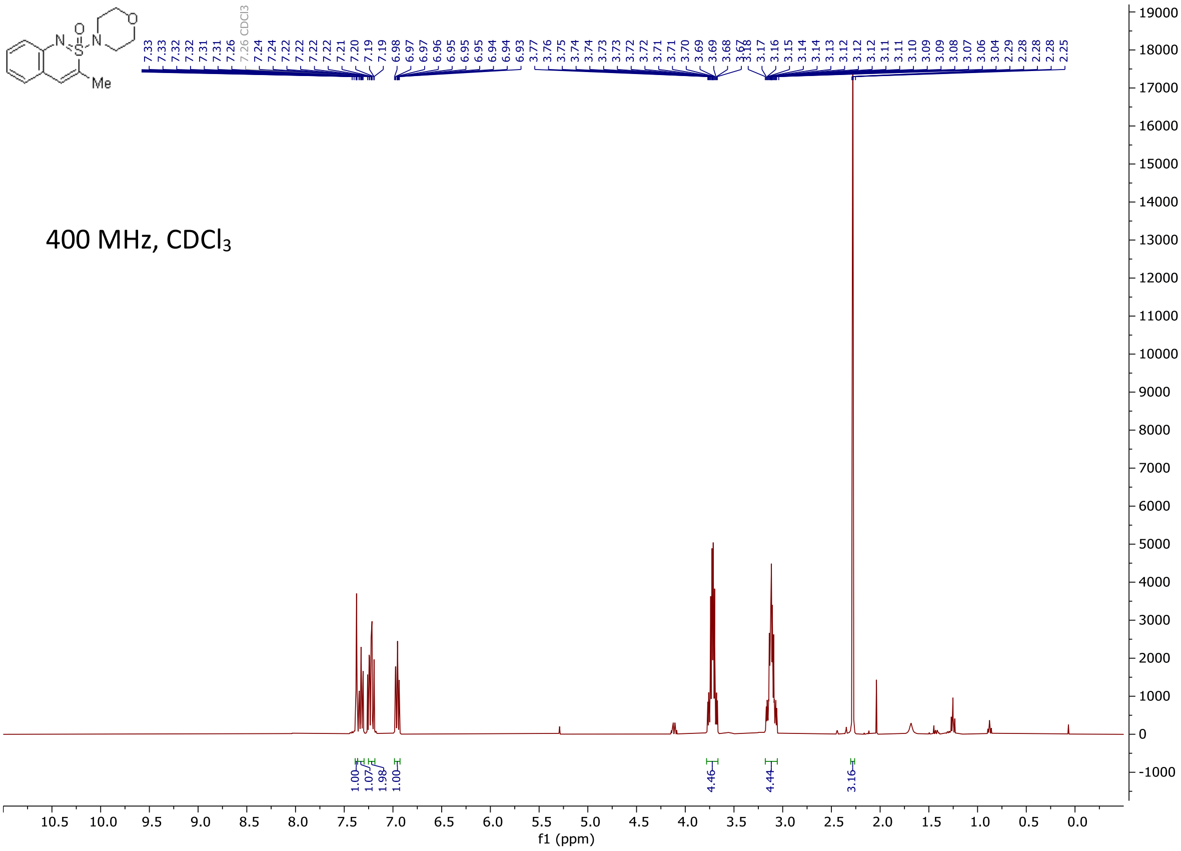

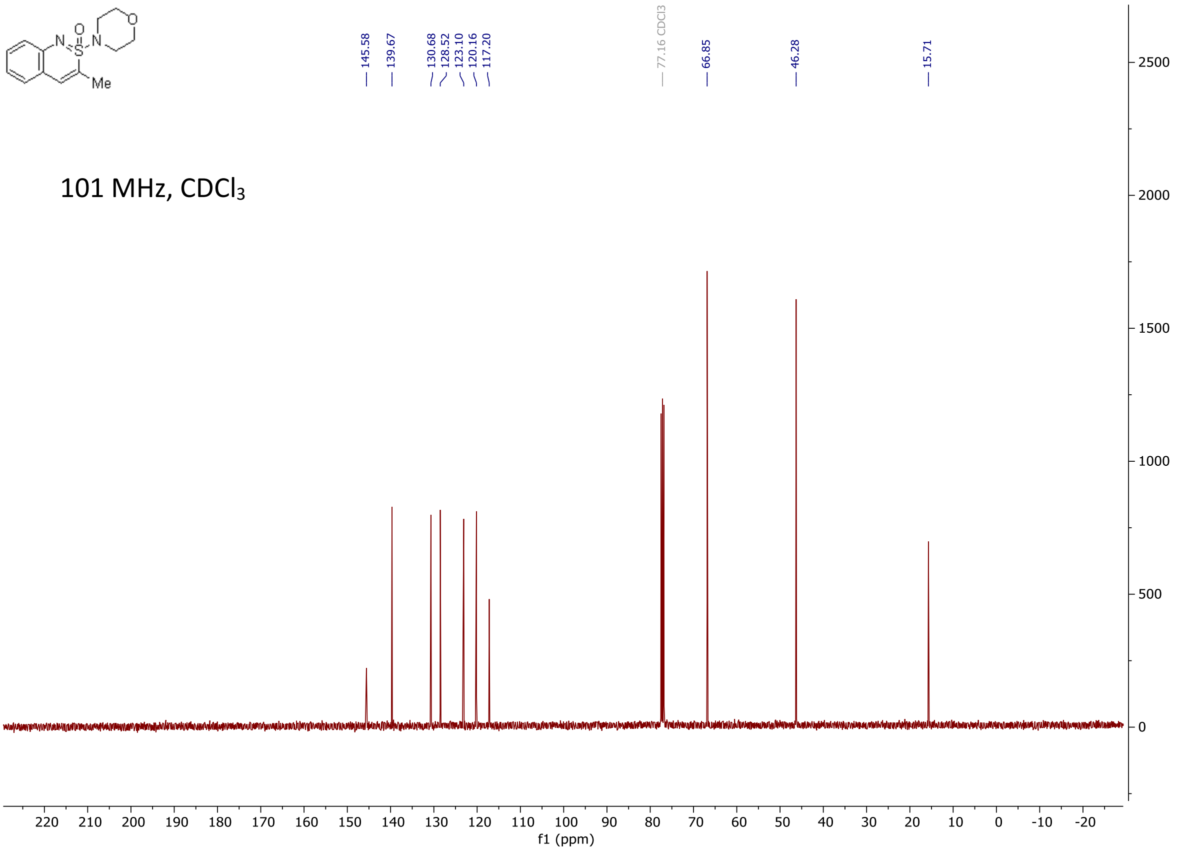

2-morpholino-3-phenylbenzo[c][1,2]thiazine 2-oxide (3u)

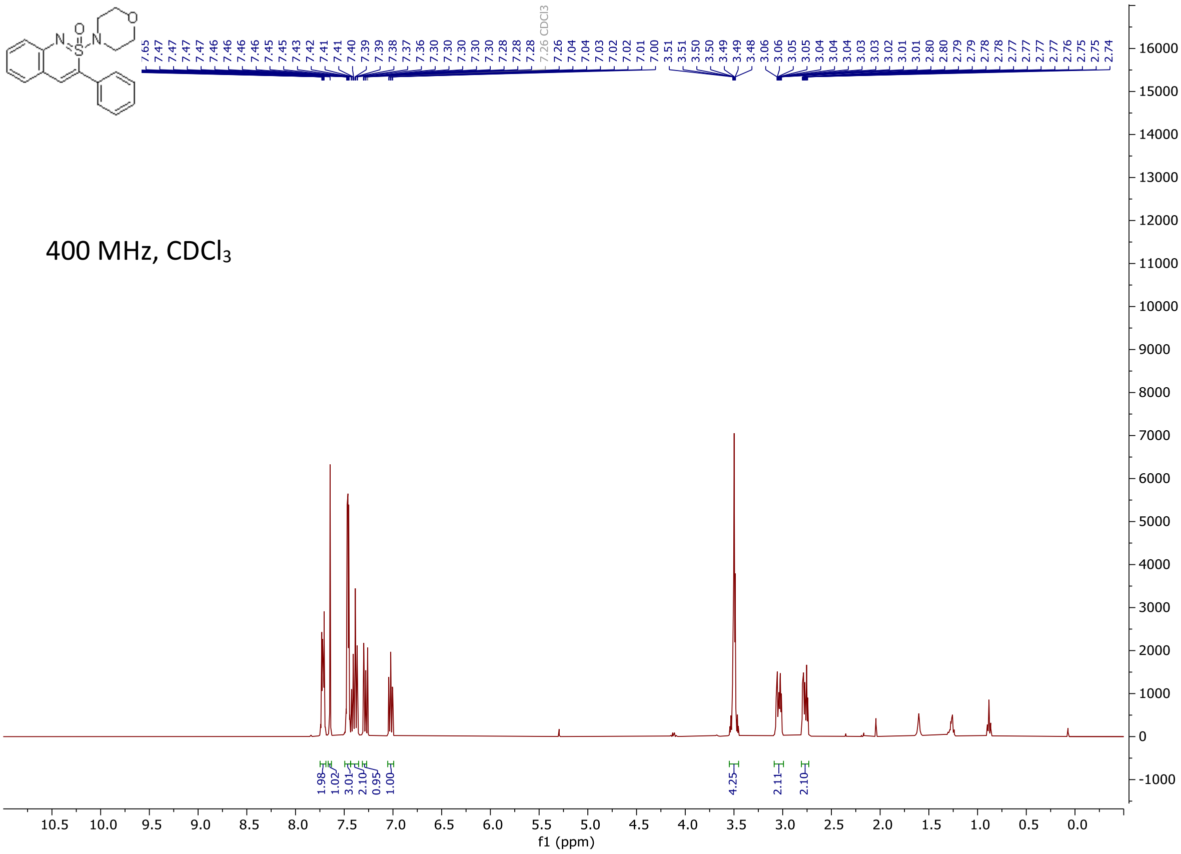

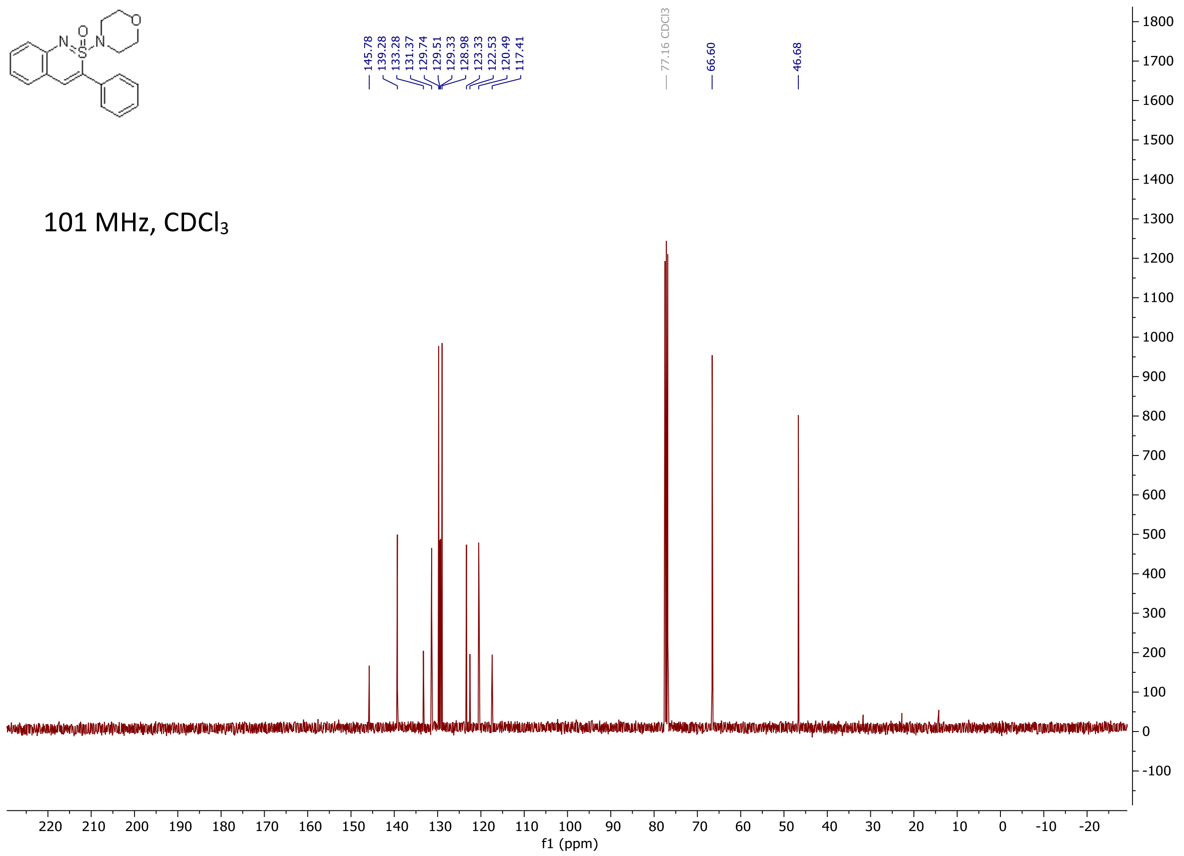

2-morpholino-3-vinylbenzo[c][1,2]thiazine 2-oxide (3v)

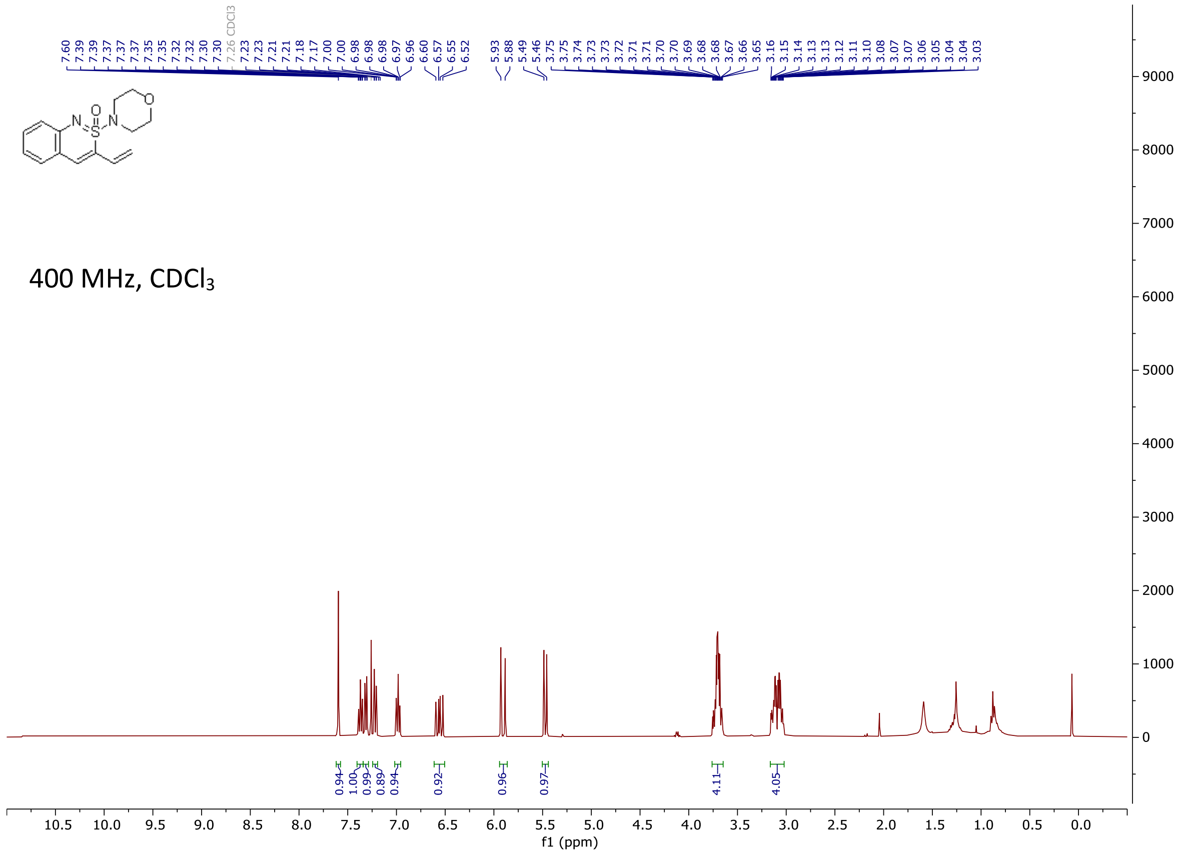

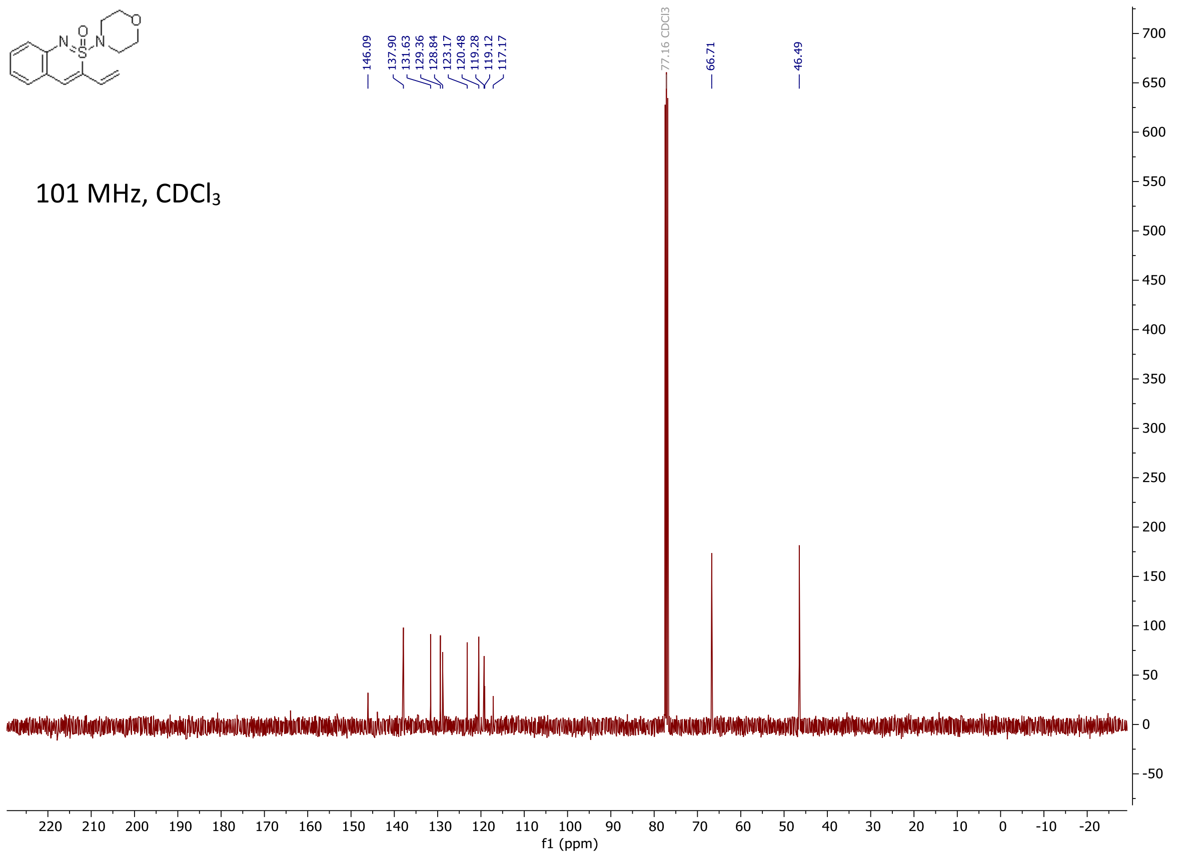

4-amino-2-morpholino-3-phenylbenzo[c][1,2]thiazine 2-oxide (3w)

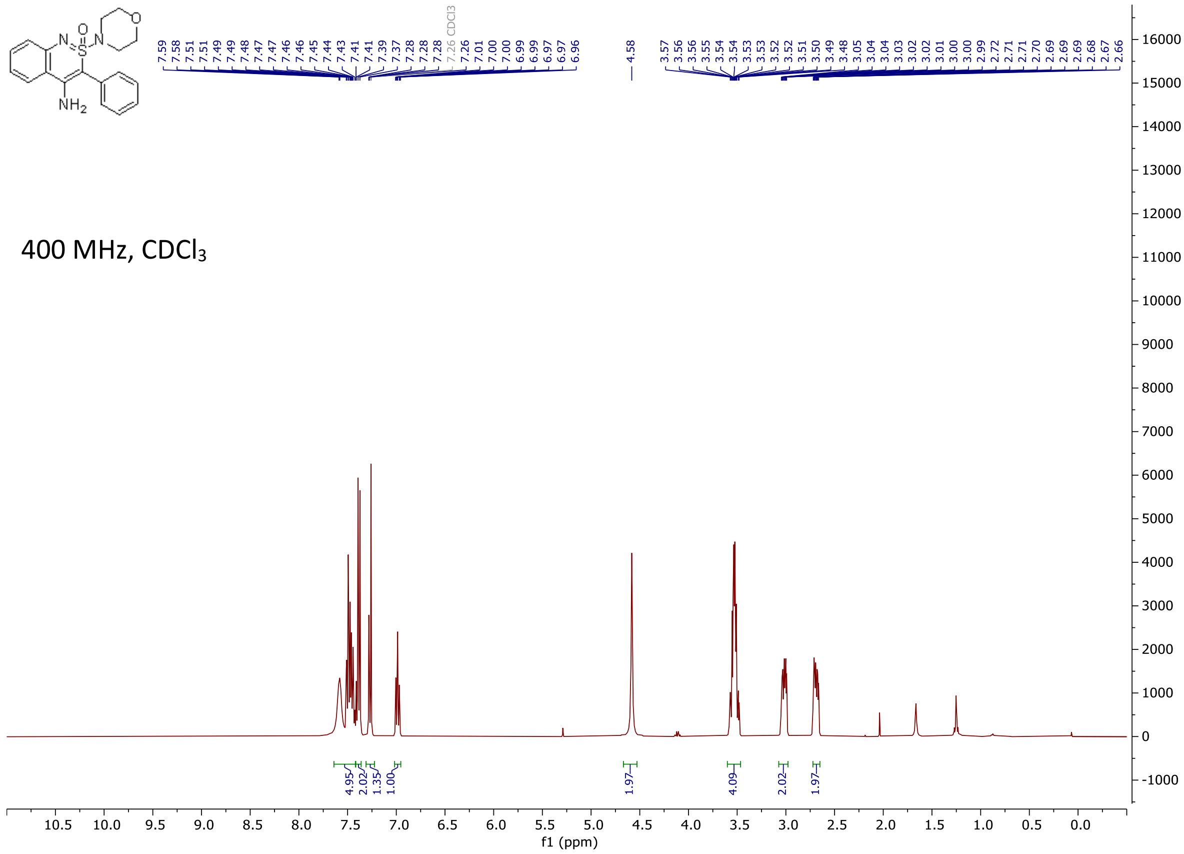

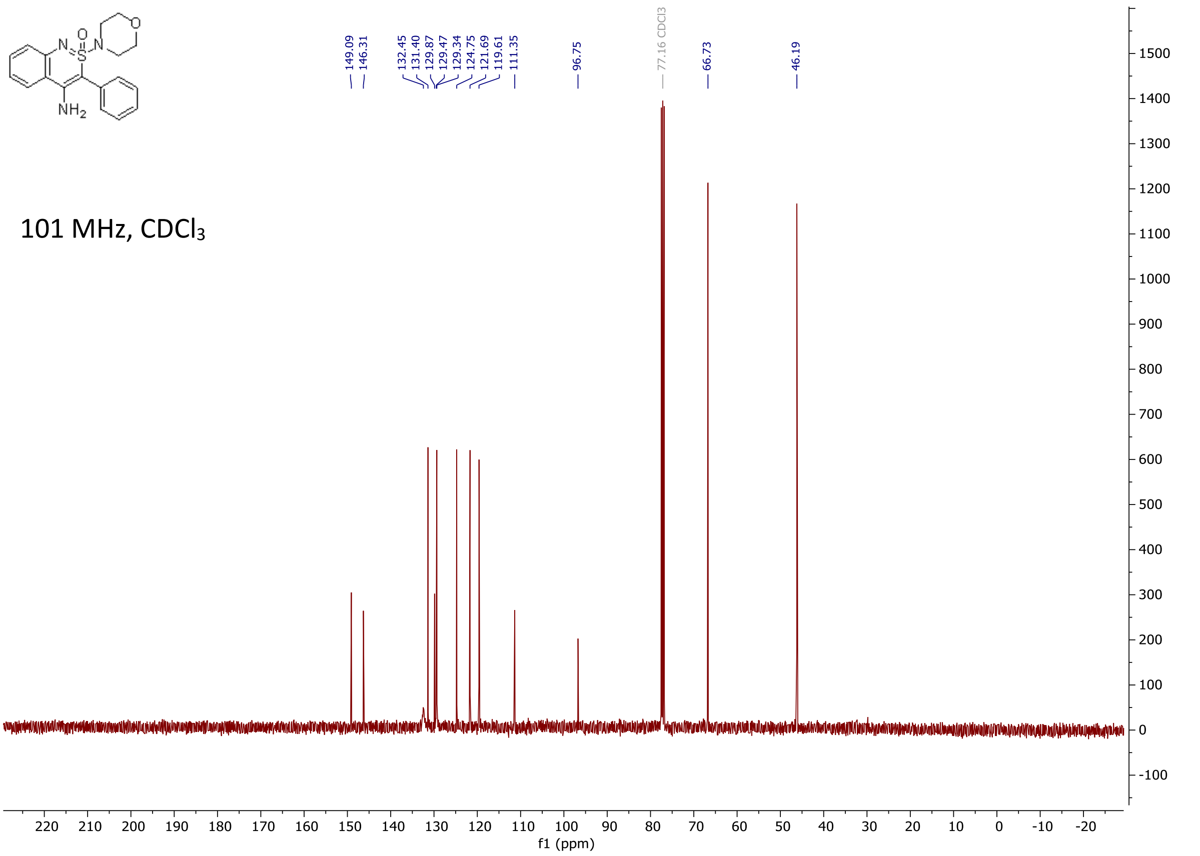

2-((methyl(morpholino)(oxo)-λ⁶-sulfaneylidene)amino)benzaldehyde (Int-1)

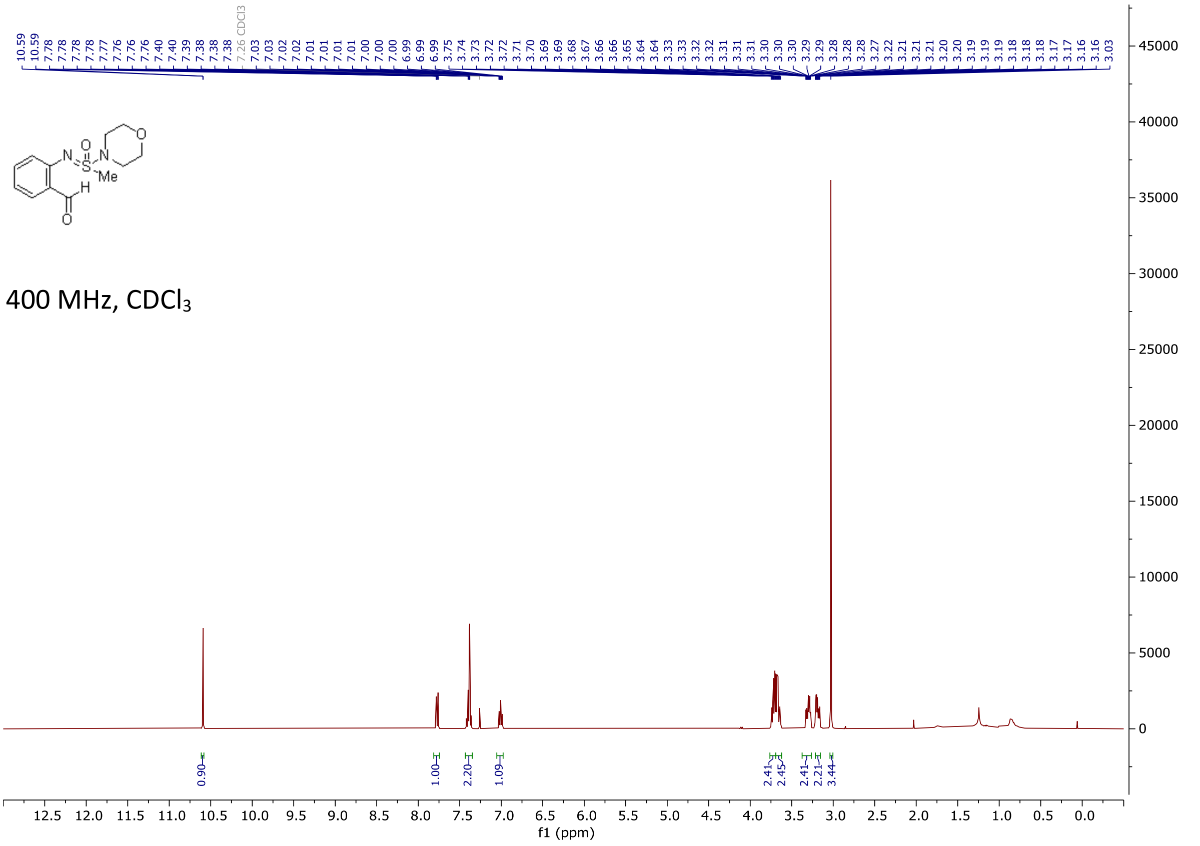

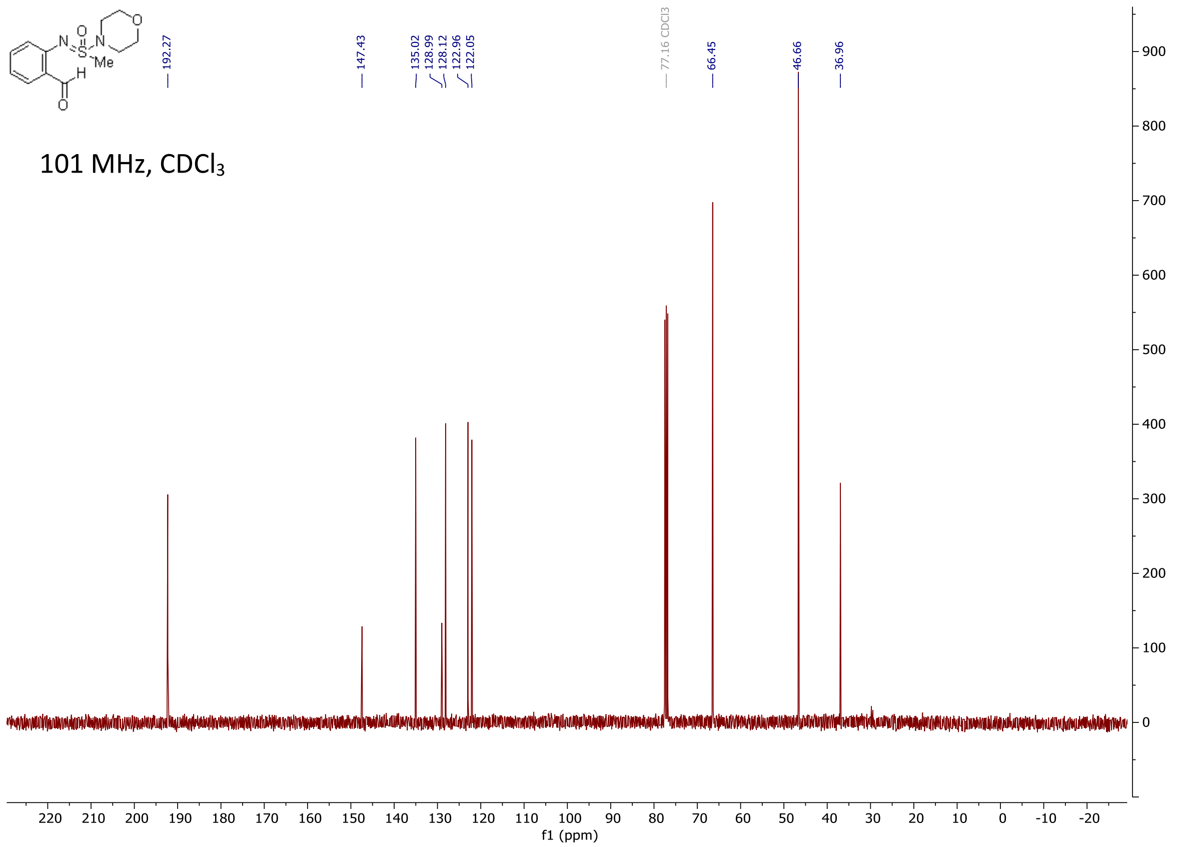

2-((methyl(morpholino)(oxo)-l6-sulfaneylidene)amino)benzonitrile (SI-5)

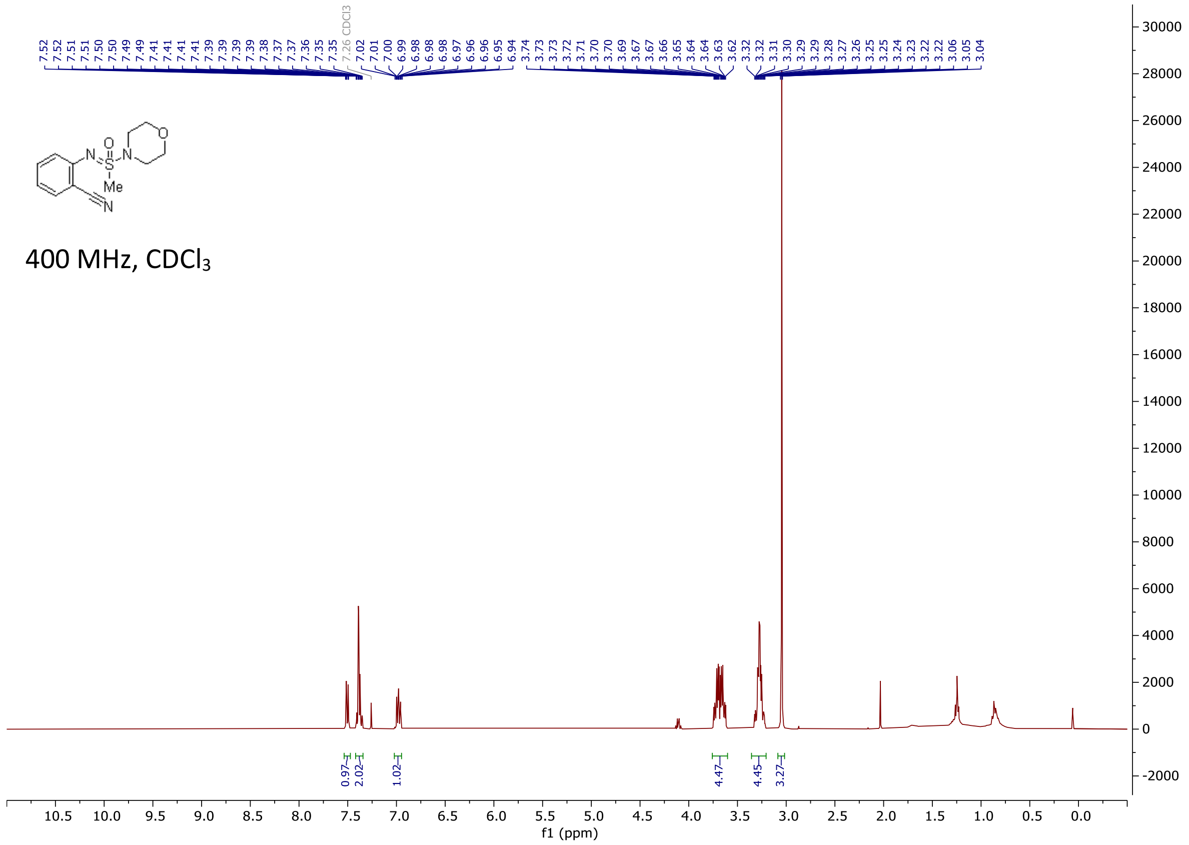

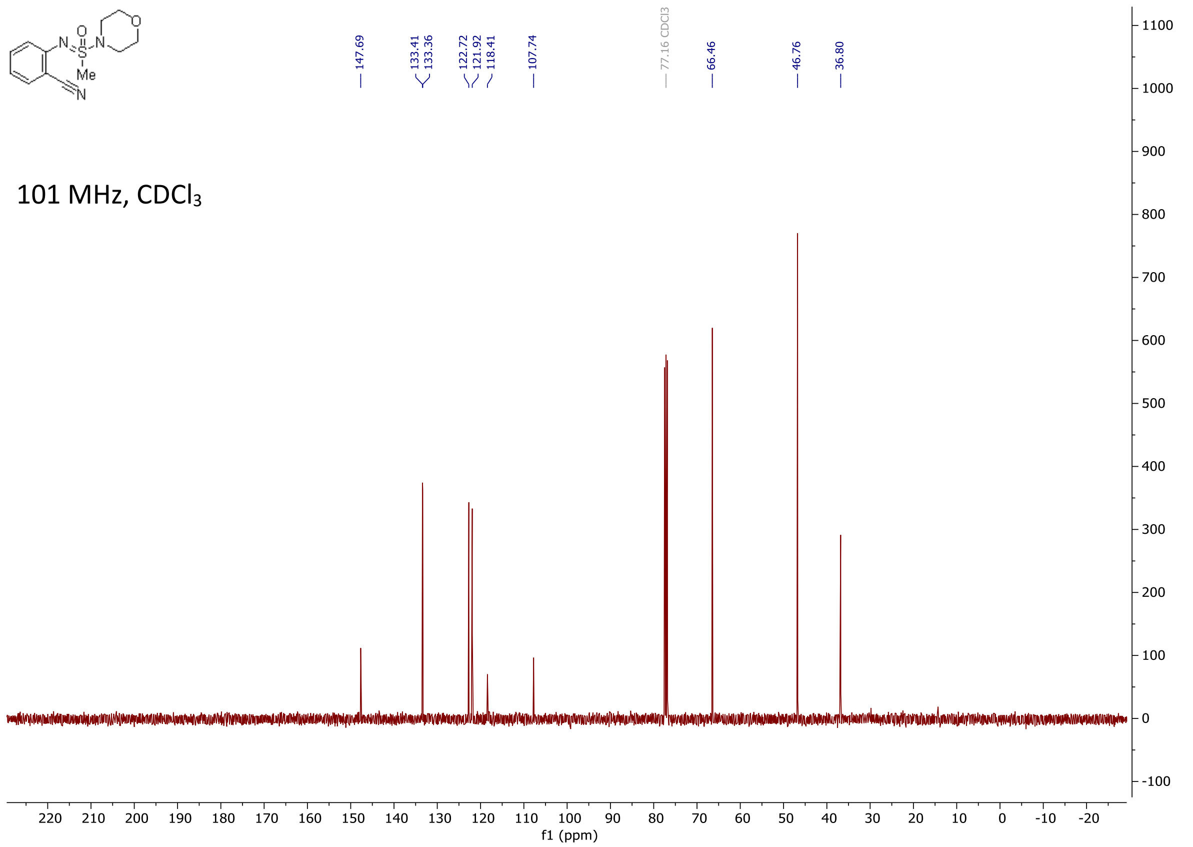

***tert*-butyl(imino)(methyl)- λ⁶-sulfanone (4)**

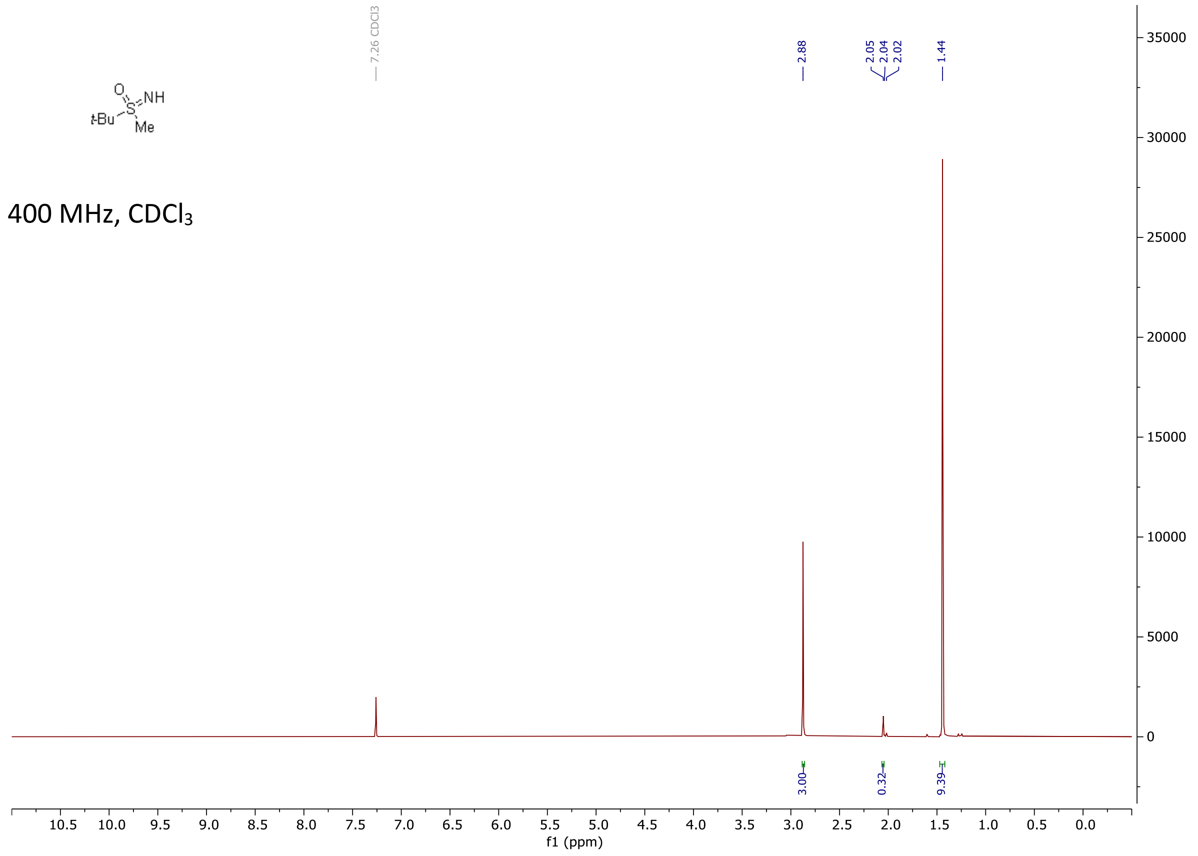

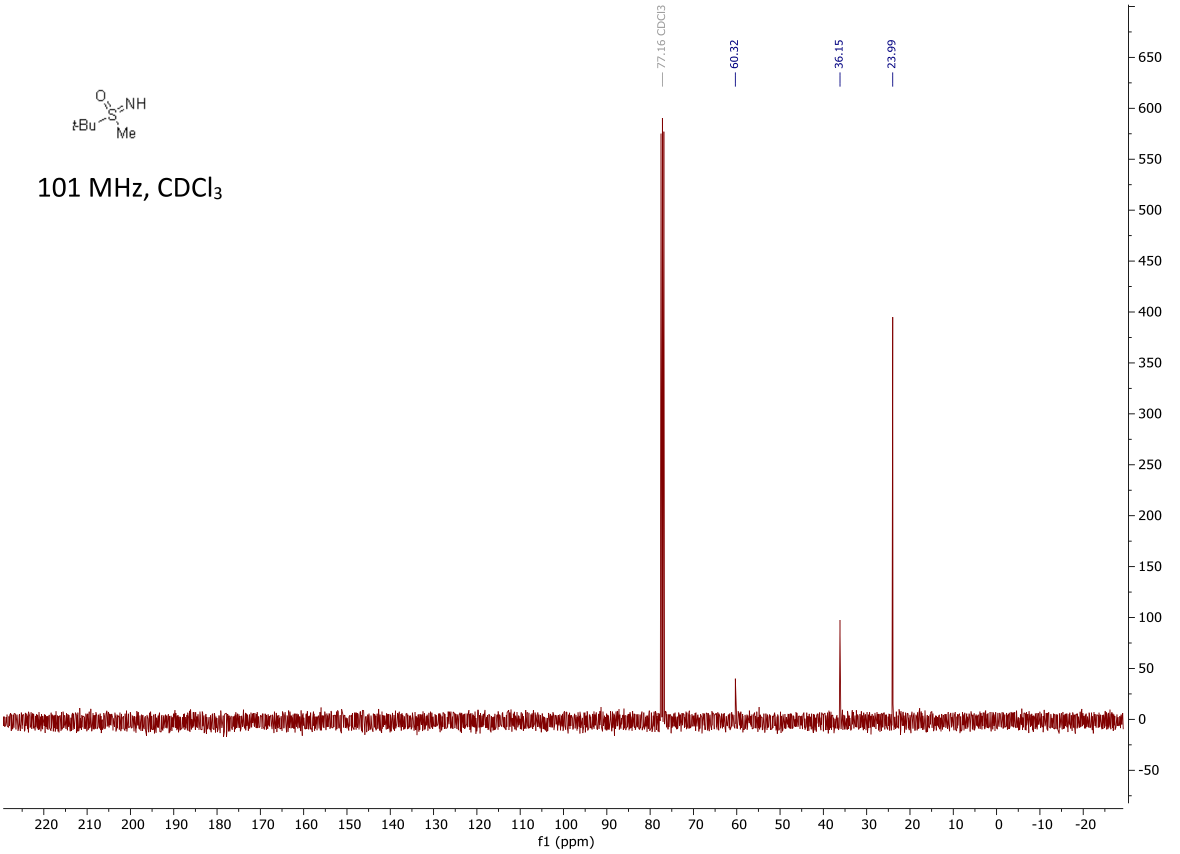

2-(*tert*-butyl)-2 λ[4]-benzo[*c*][1,2]thiazine 2-oxide (5)

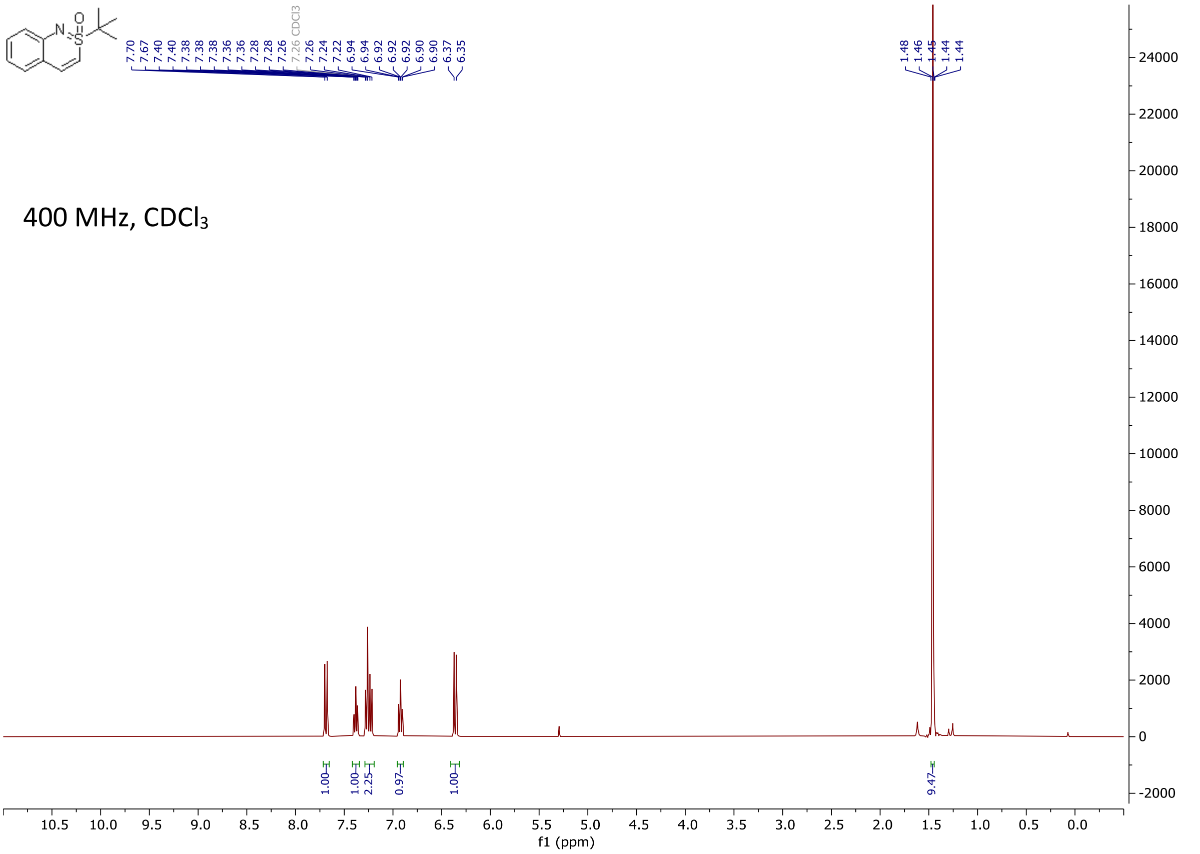

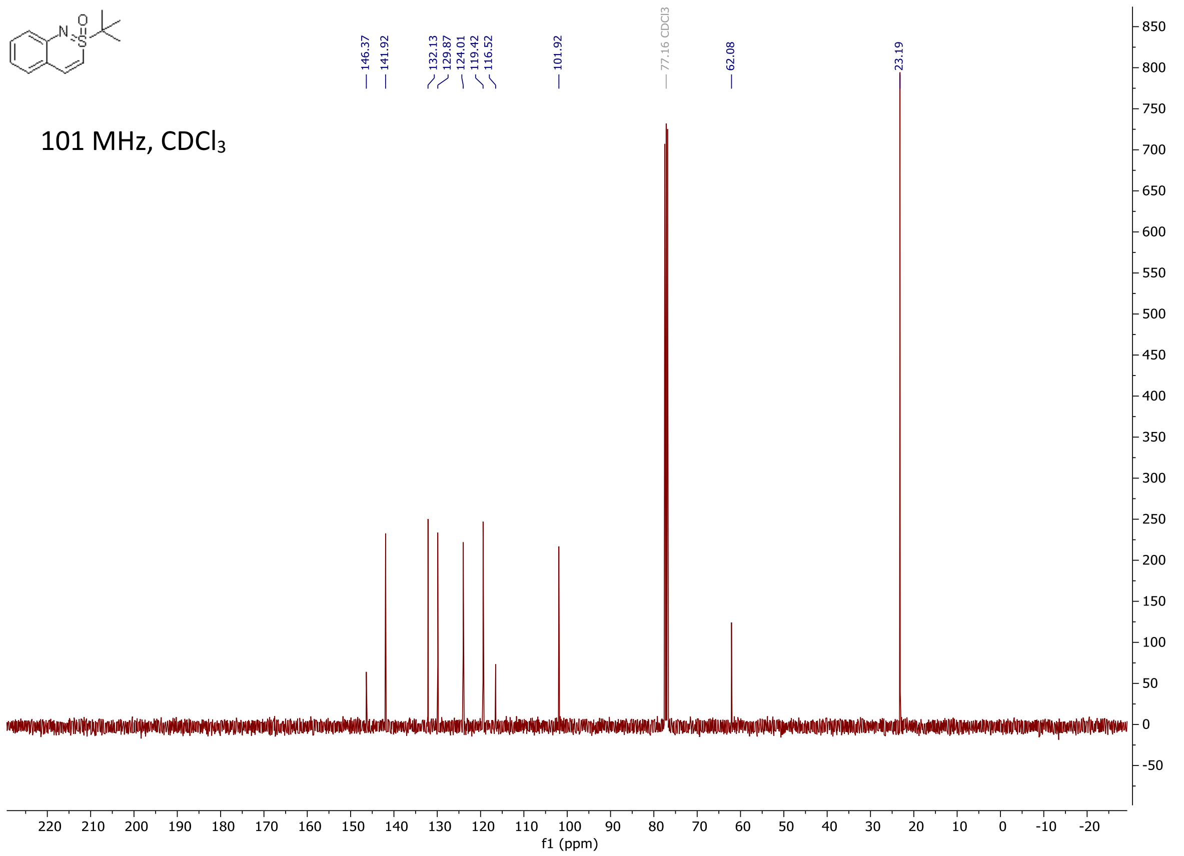

1*H*-benzo[*c*][1,2]thiazine 2-oxide (6)

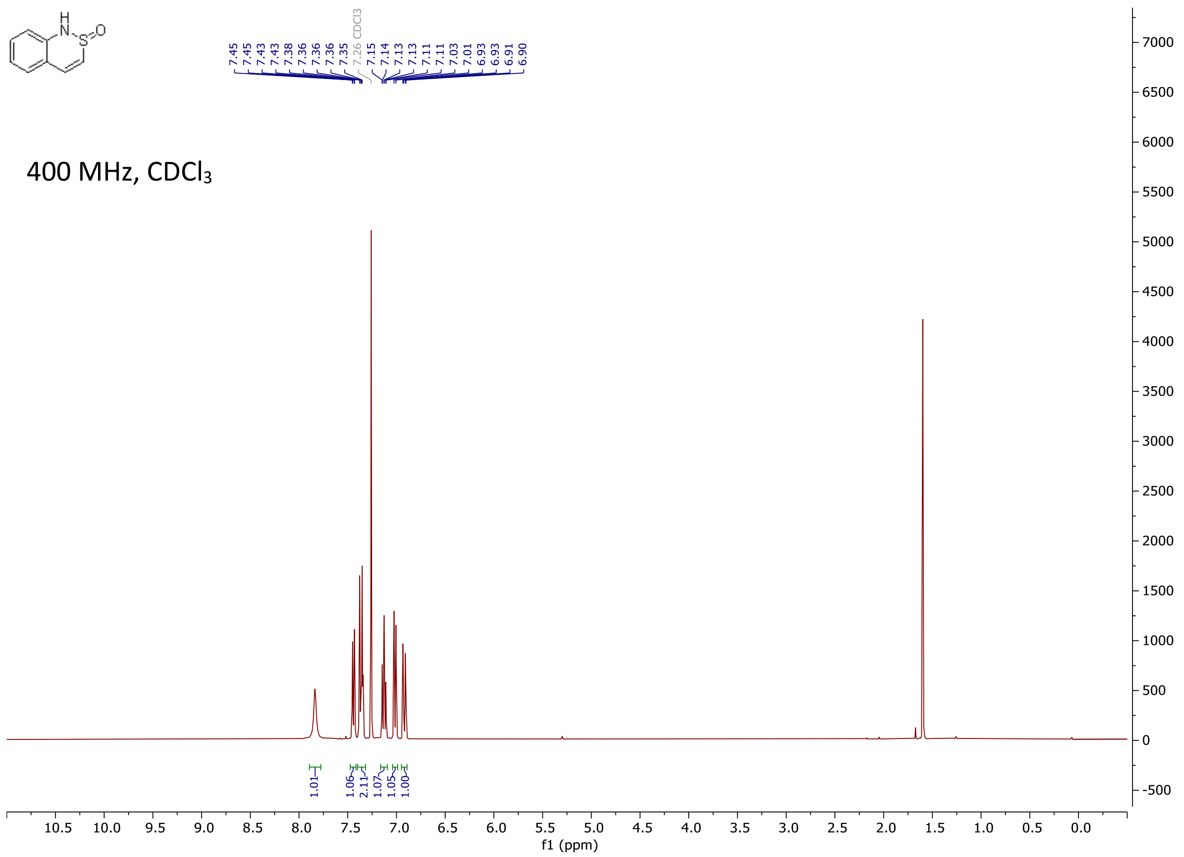

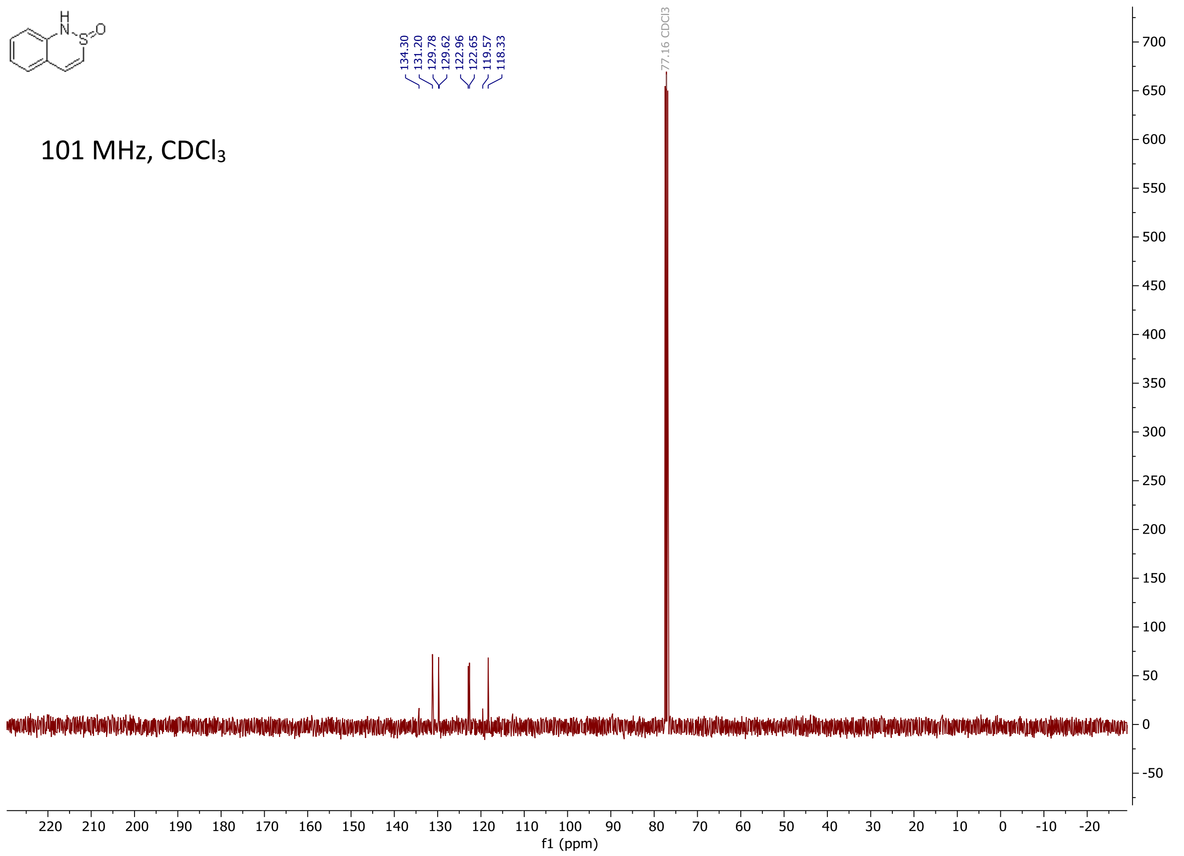

1H-benzo[*c*][1,2]thiazine 2,2-dioxide (7)

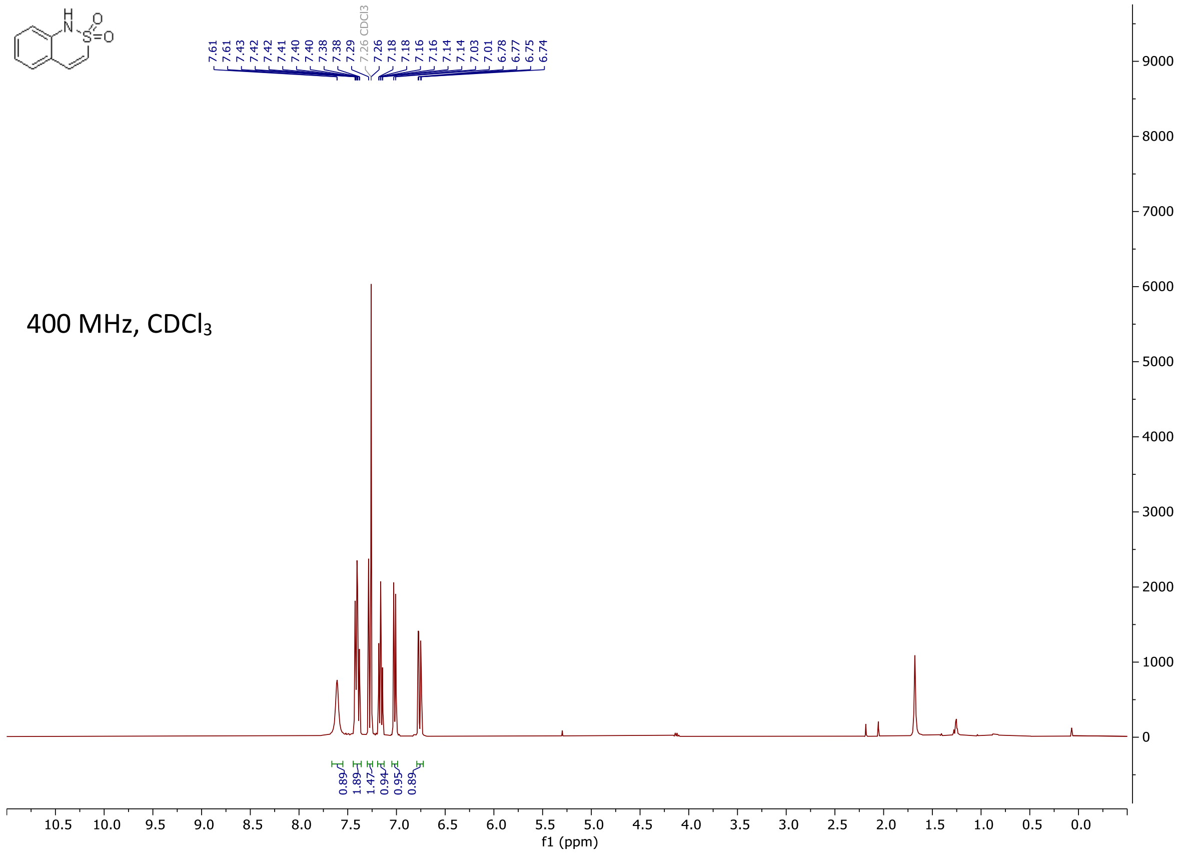

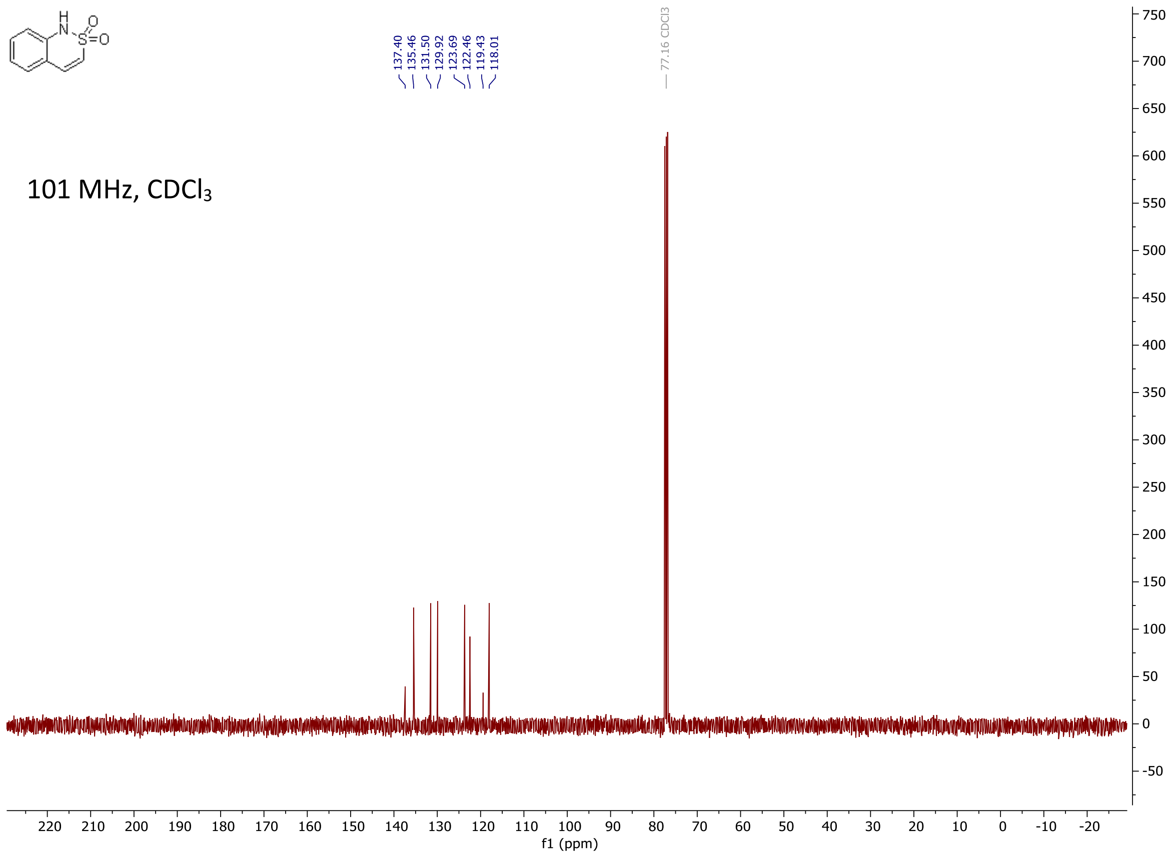

2-(butylamino)-2λ^4-benzo[*c*][1,2]thiazine 2-oxide (9a)

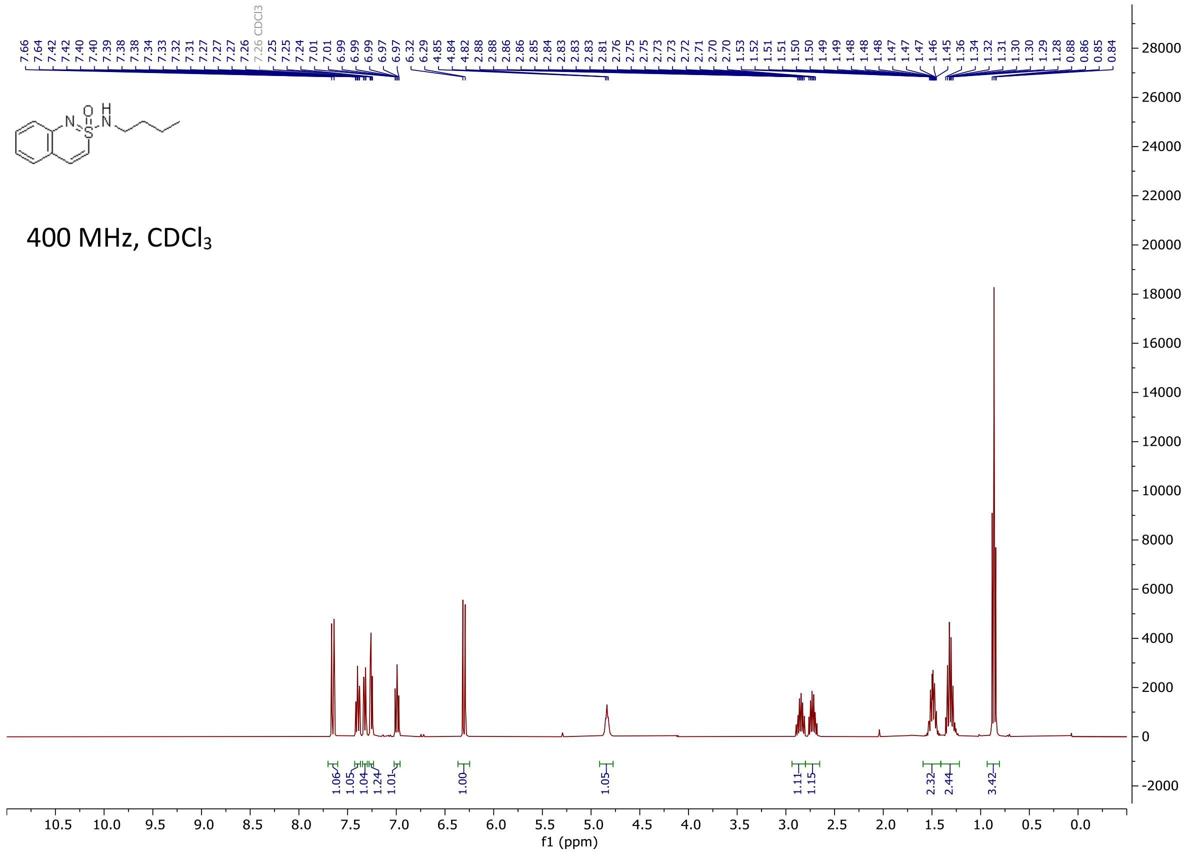

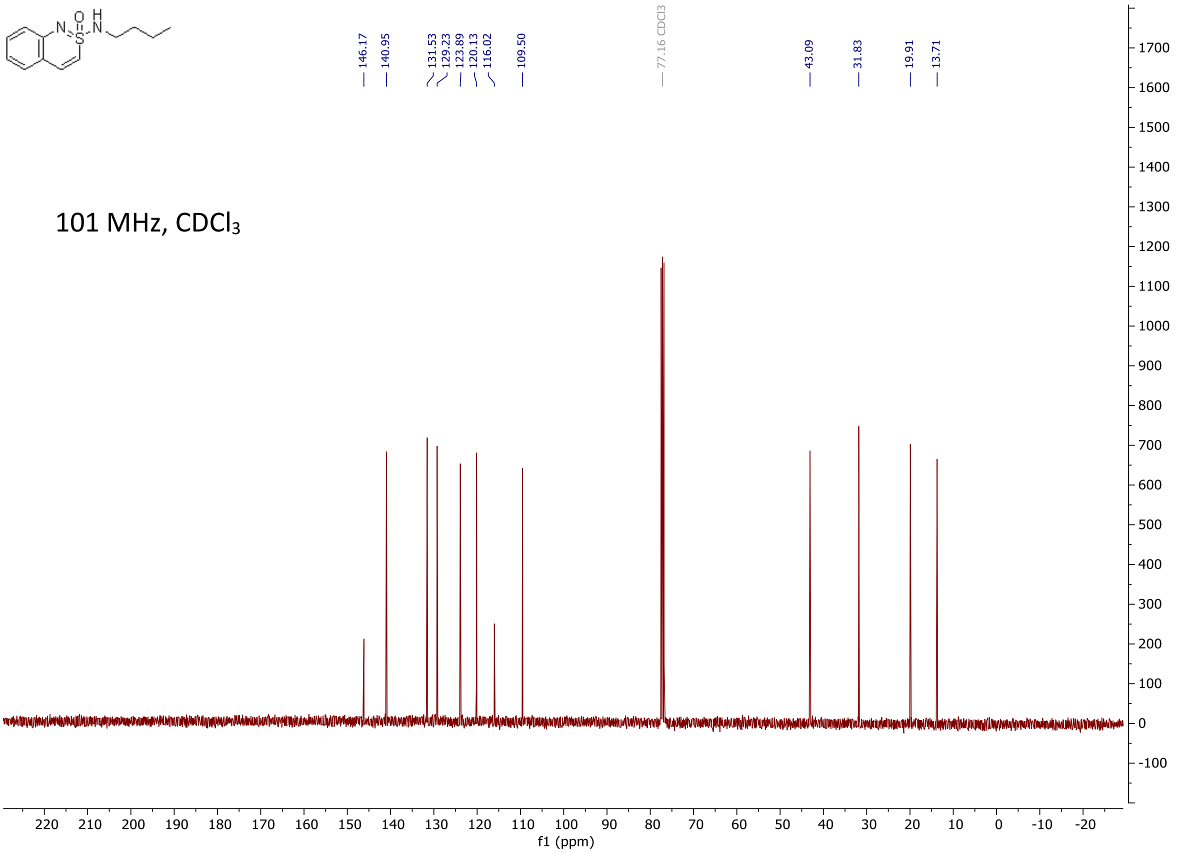

2-(benzylamino)-2λ^4-benzo[*c*][1,2]thiazine 2-oxide (9b)

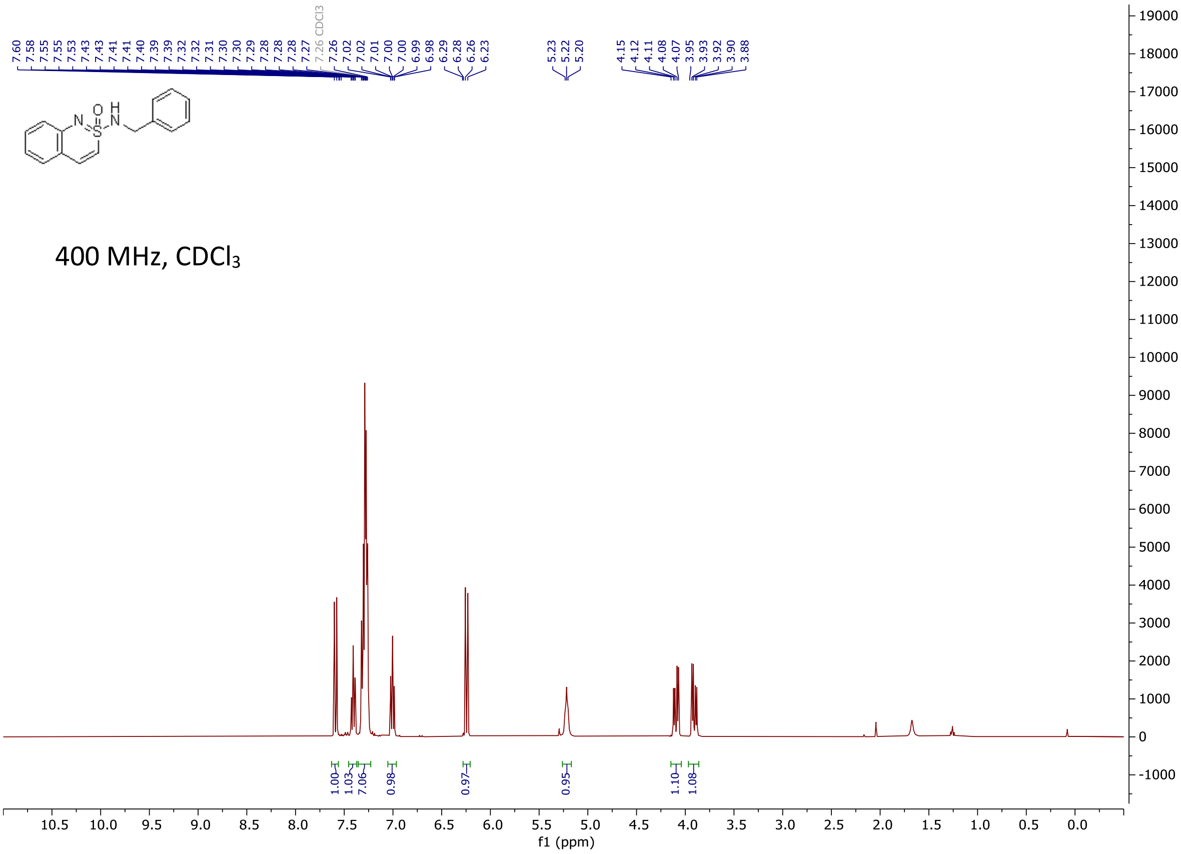

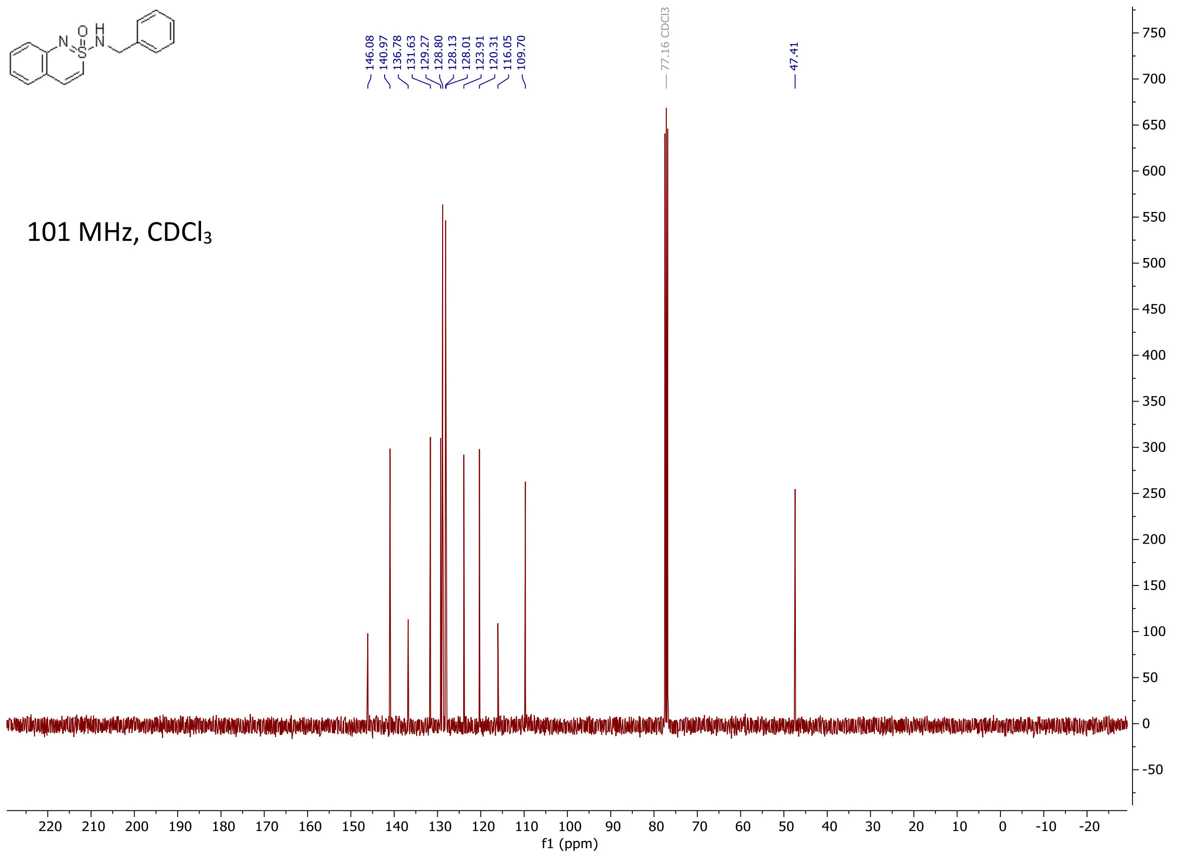

(*S*)-2-(((*S*)-1-phenylethyl)amino)-2λ[4]-benzo[*c*][1,2]thiazine 2-oxide (9c)

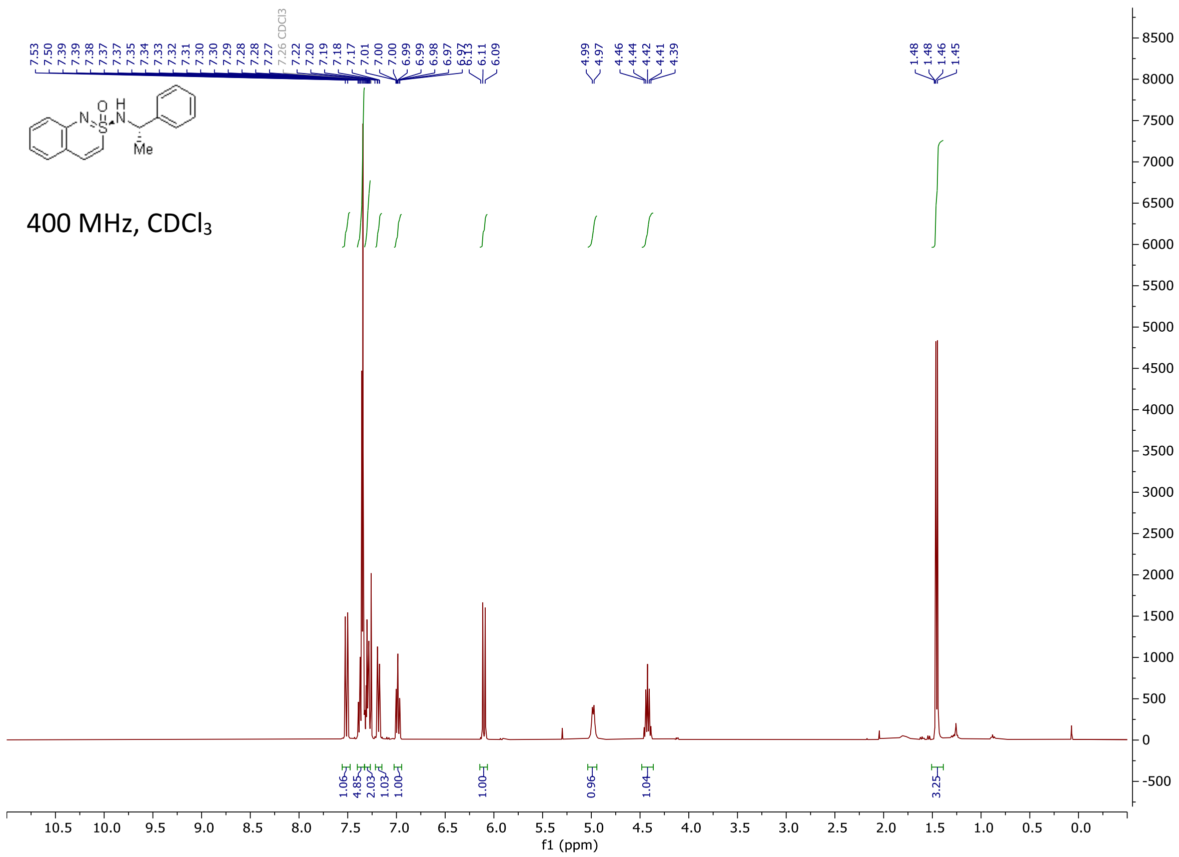

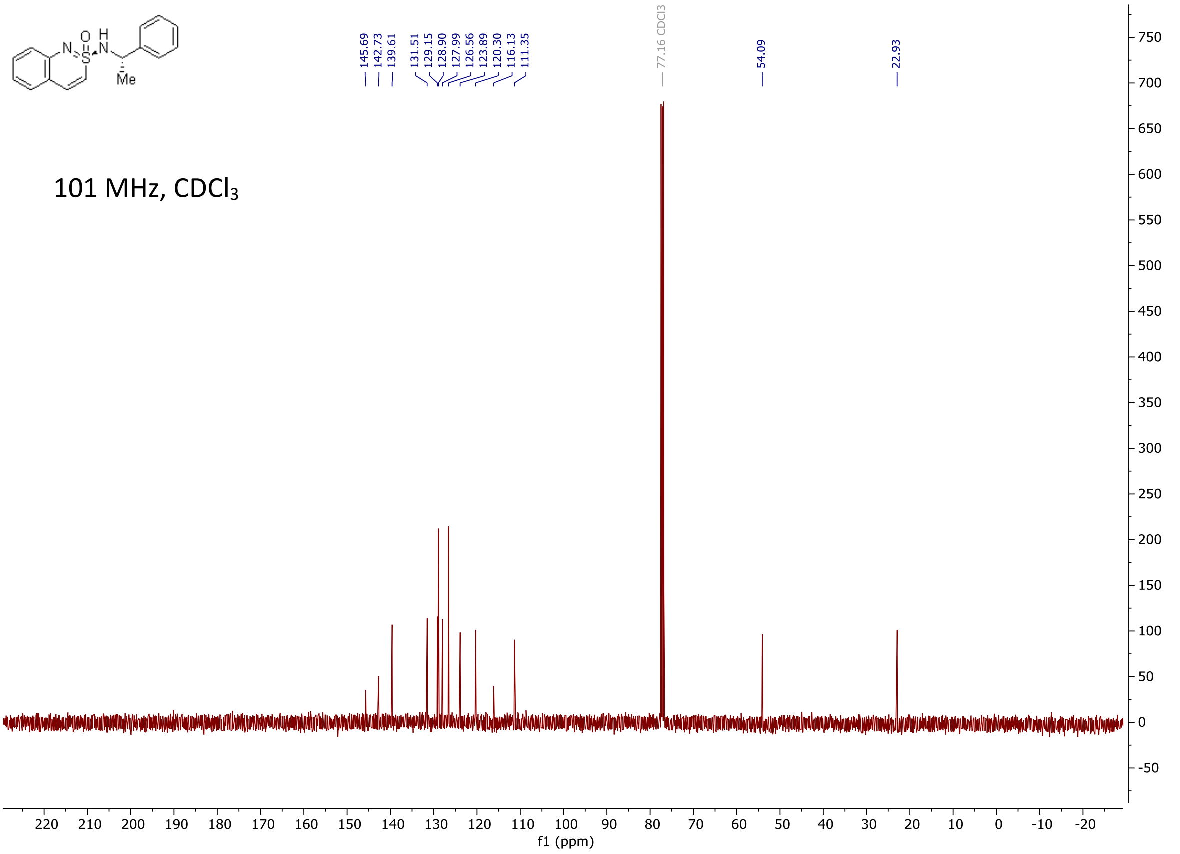

(*R*)-2-(((*S*)-1-phenylethyl)amino)-2λ[4]-benzo[*c*][1,2]thiazine 2-oxide (9c’)

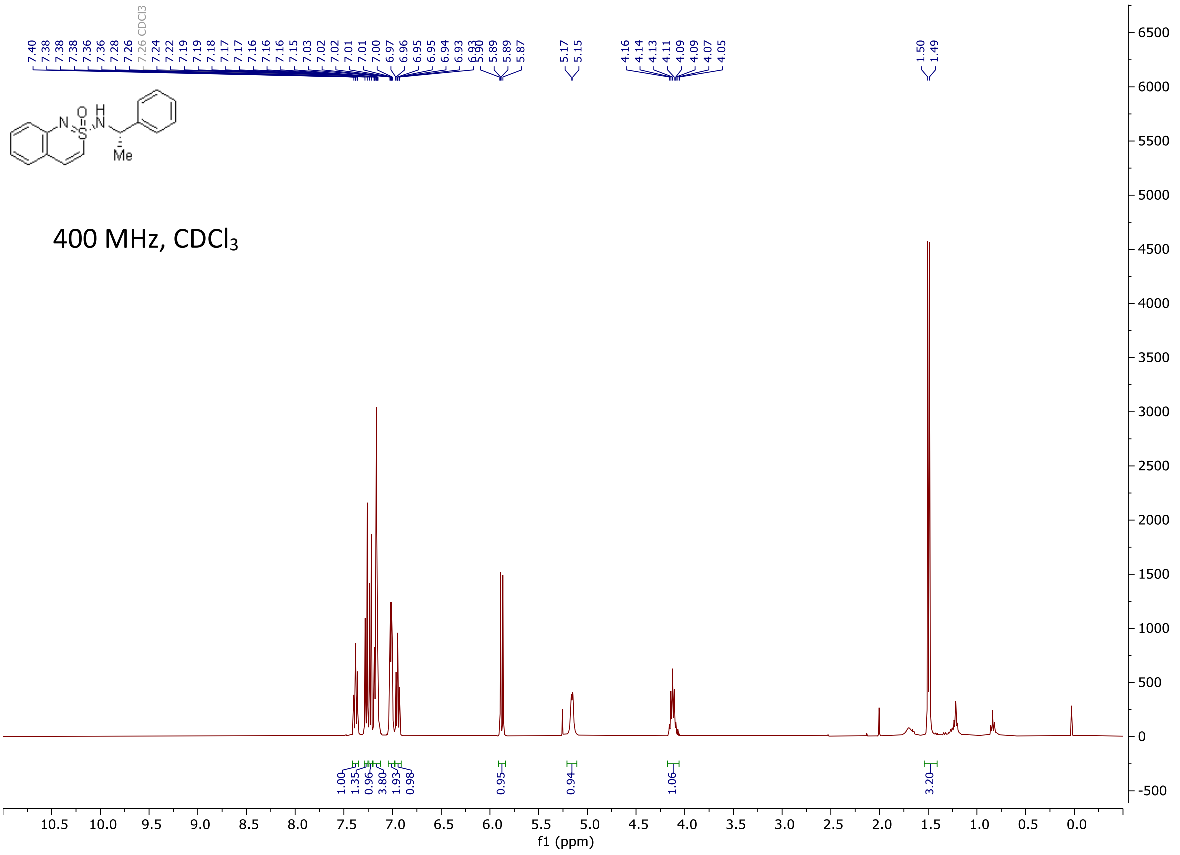

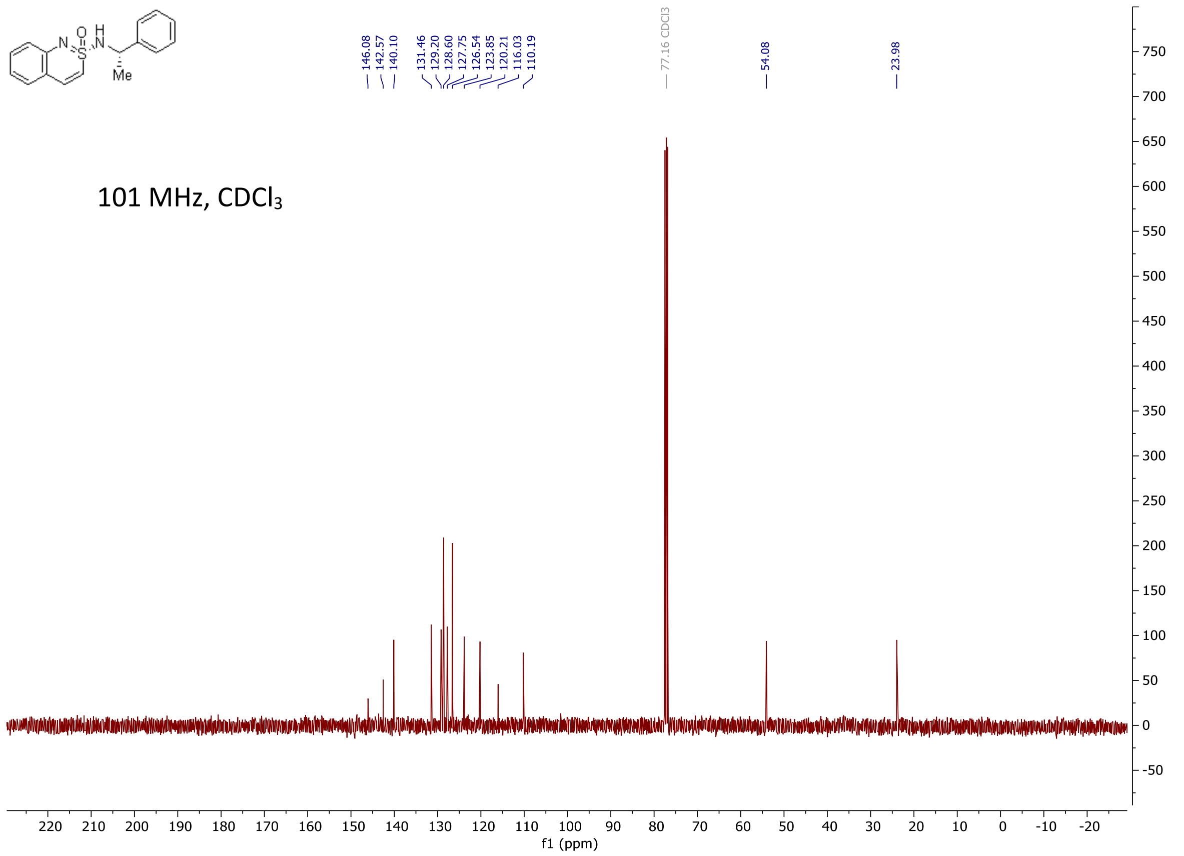

2-(tert-butylamino)-2 λ[4]-benzo[*c*][1,2]thiazine 2-oxide (9d)

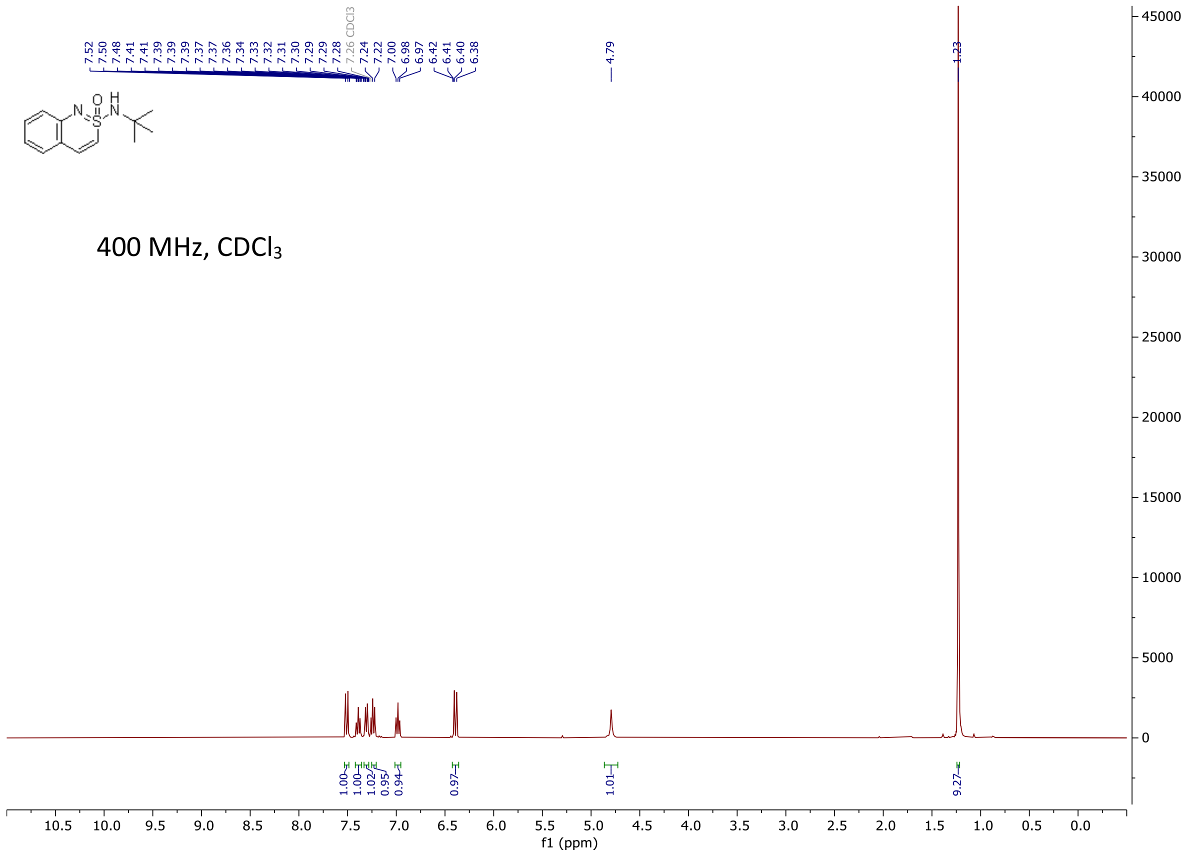

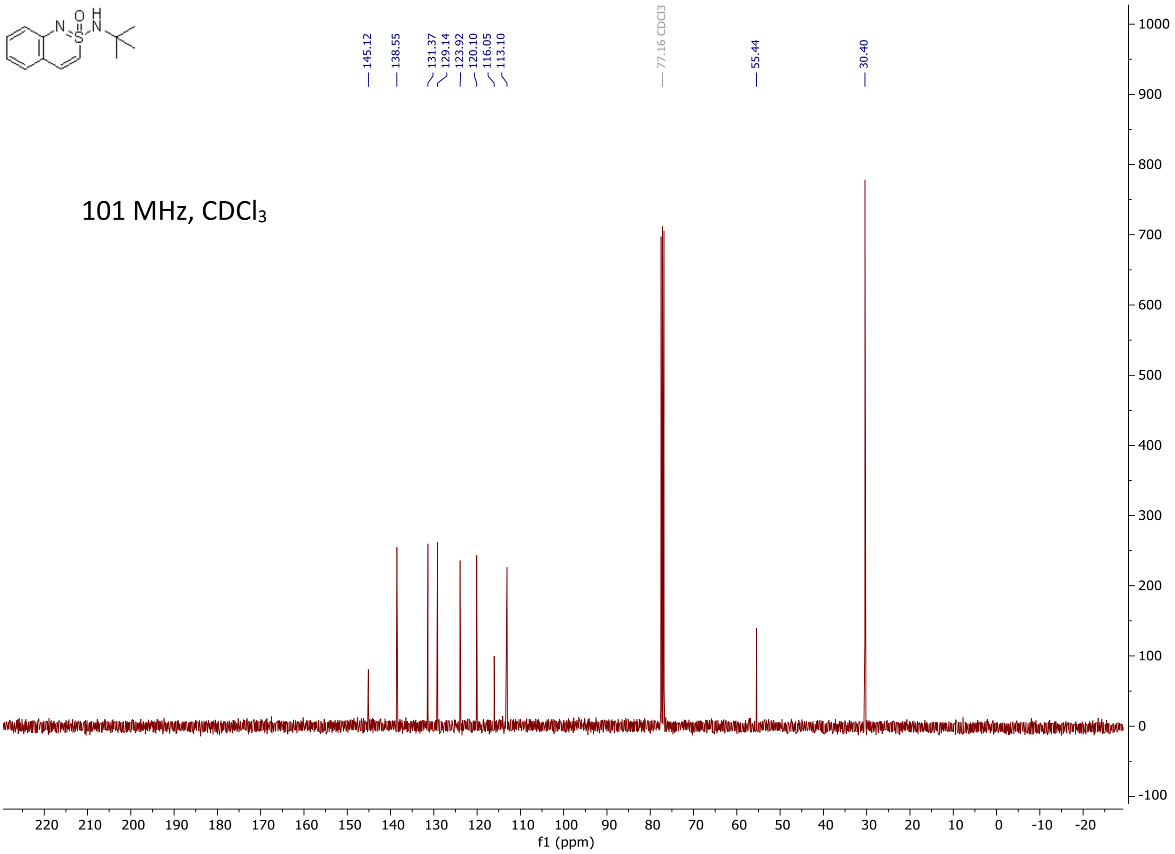

2-(phenylamino)-2λ[4]-benzo[*c*][1,2]thiazine 2-oxide (9e)

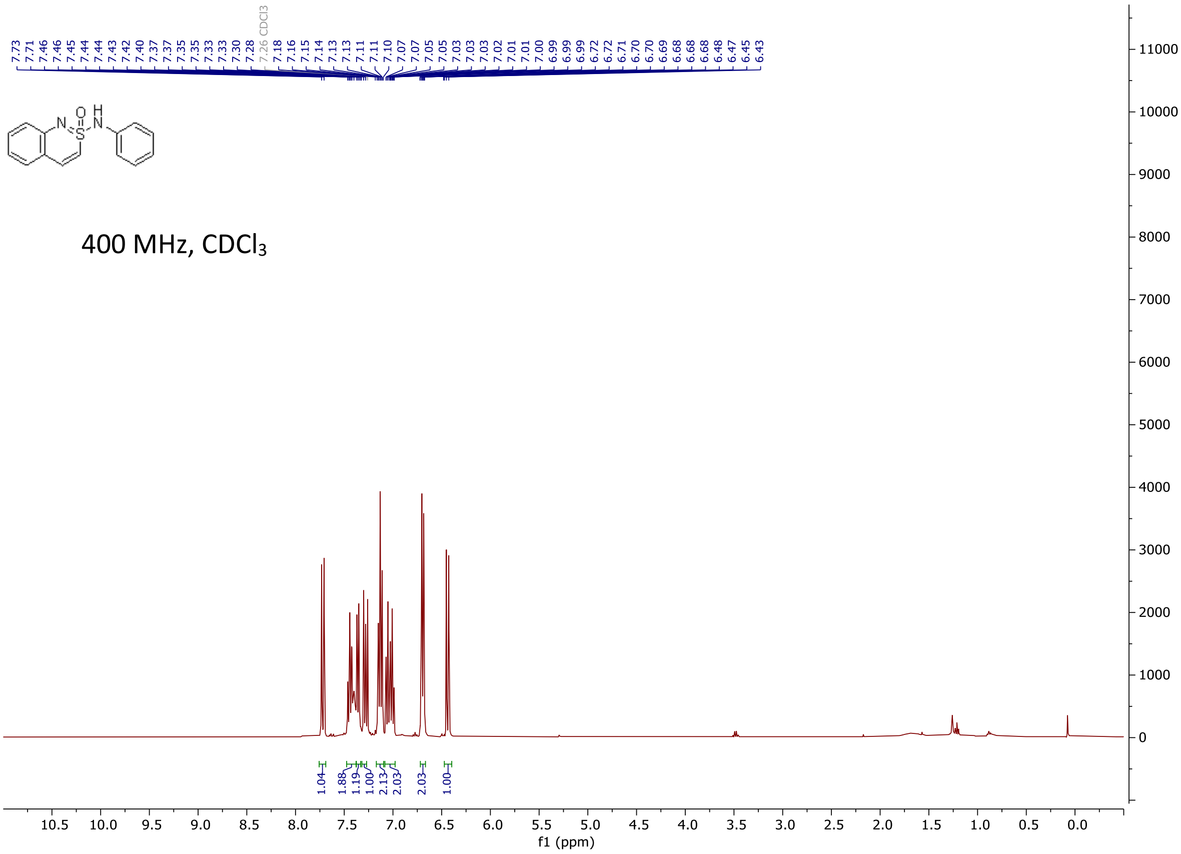

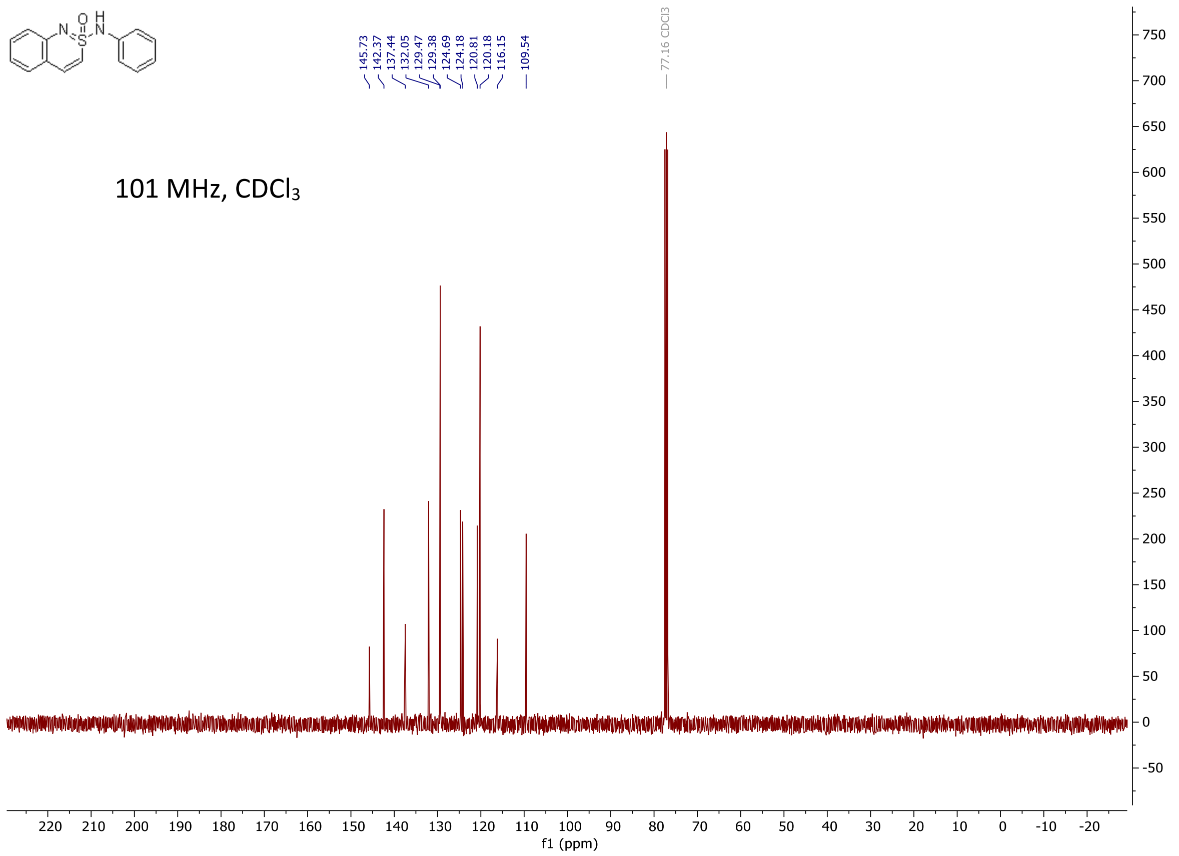

2-amino-2λ⁴-benzo[*c*][1,2]thiazine 2-oxide (9f)

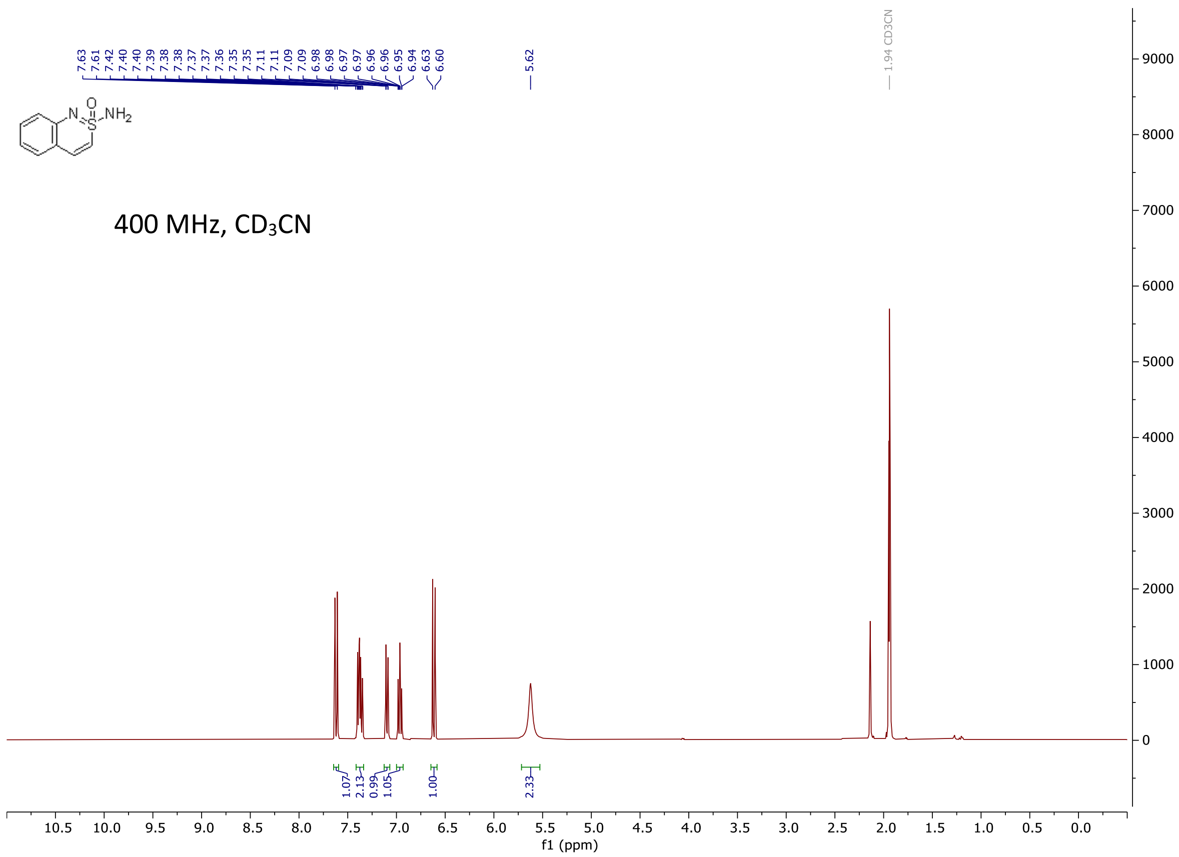

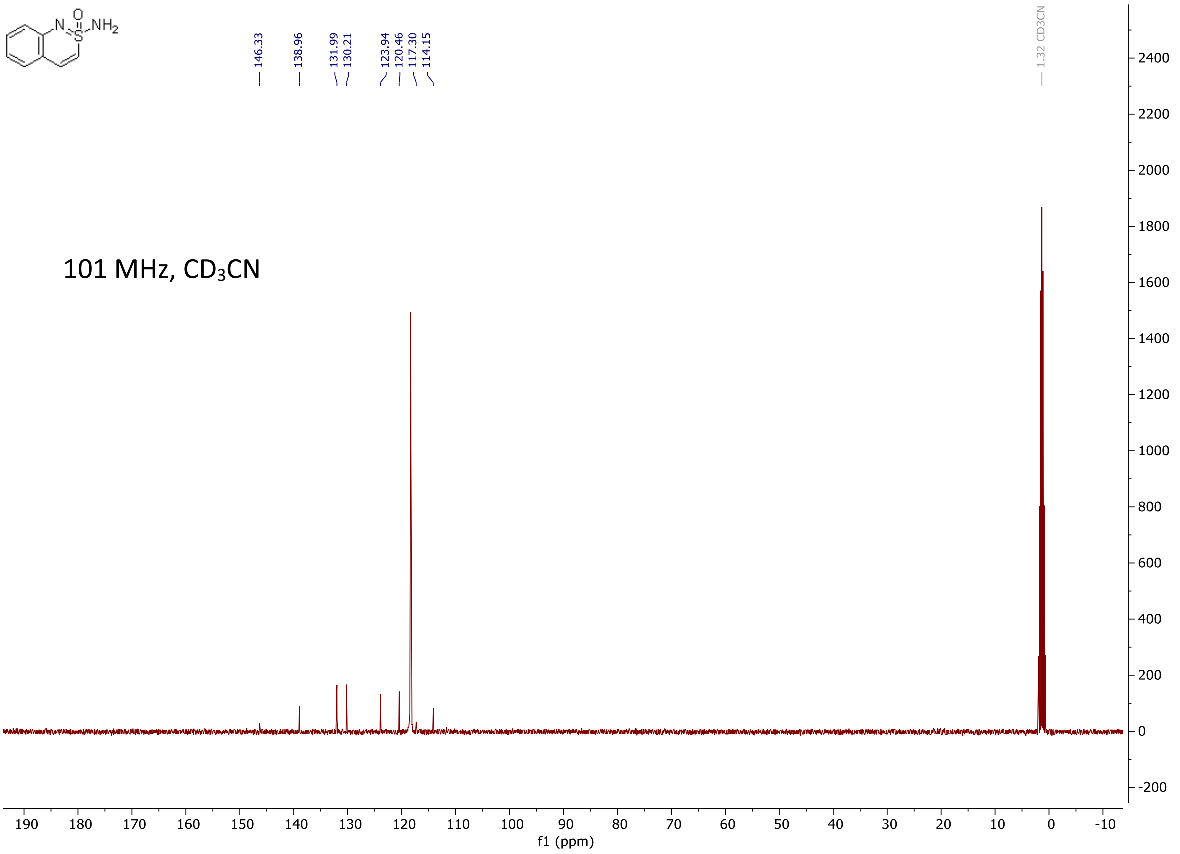

2-fluoro-2λ[4]-benzo[*c*][1,2]thiazine 2-oxide (10)

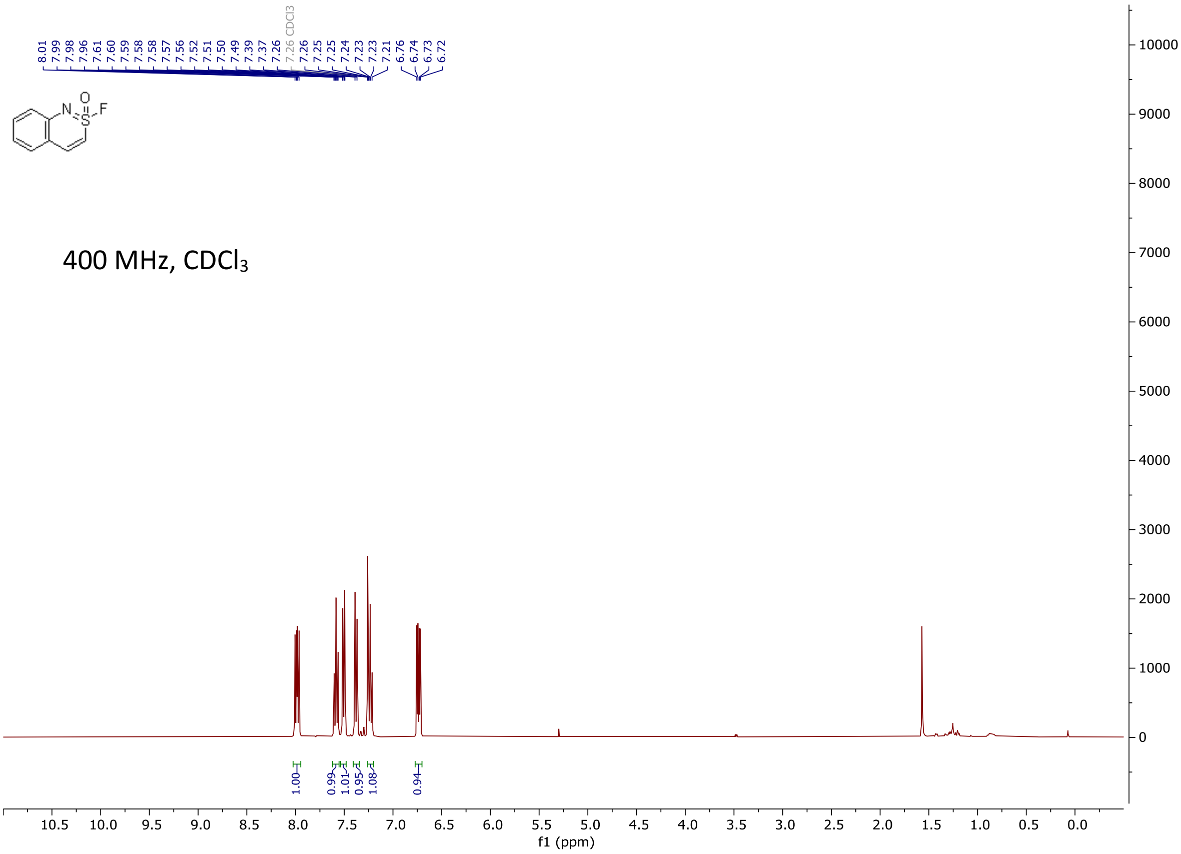

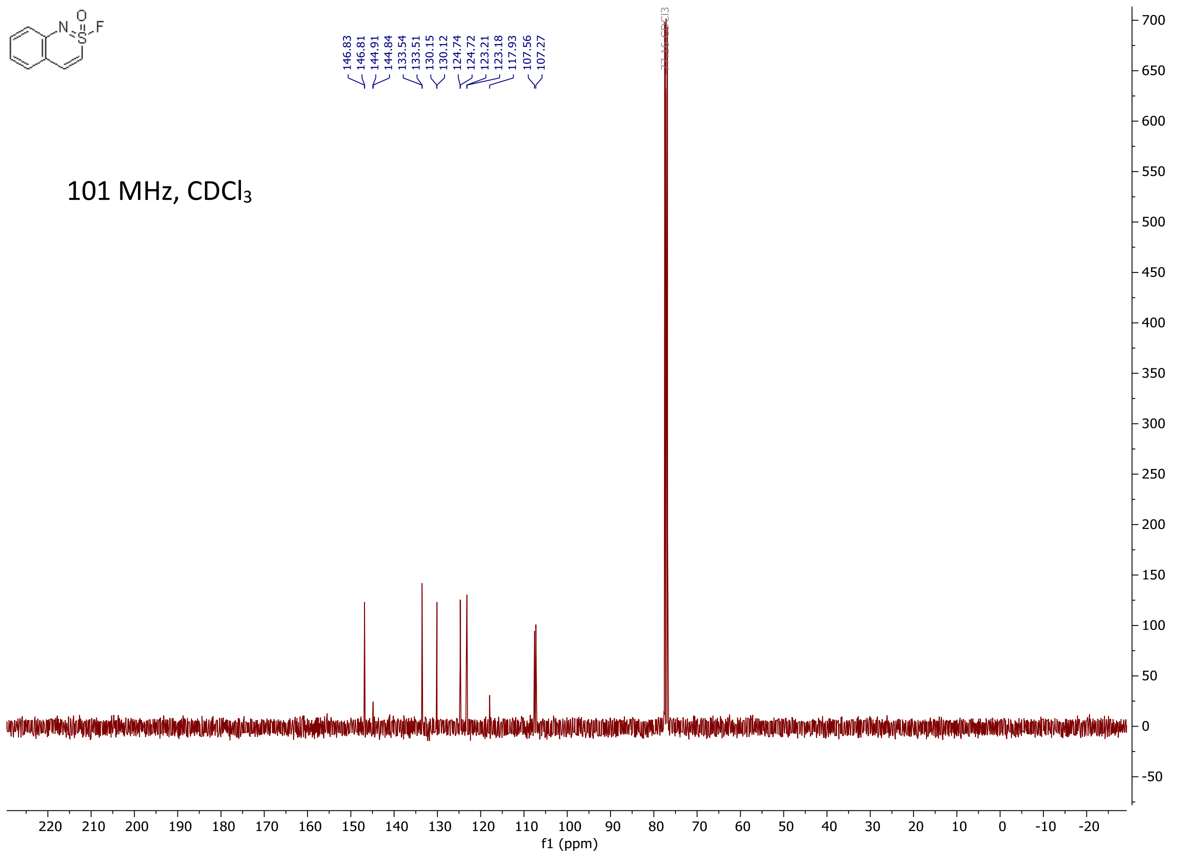
101 MHz, CDCl3
146.83
146.81
144.91
144.84
133.54
133.51
130.15
130.12
124.74
124.72
123.21
123.18
117.93
107.56
107.27
f1 (ppm)

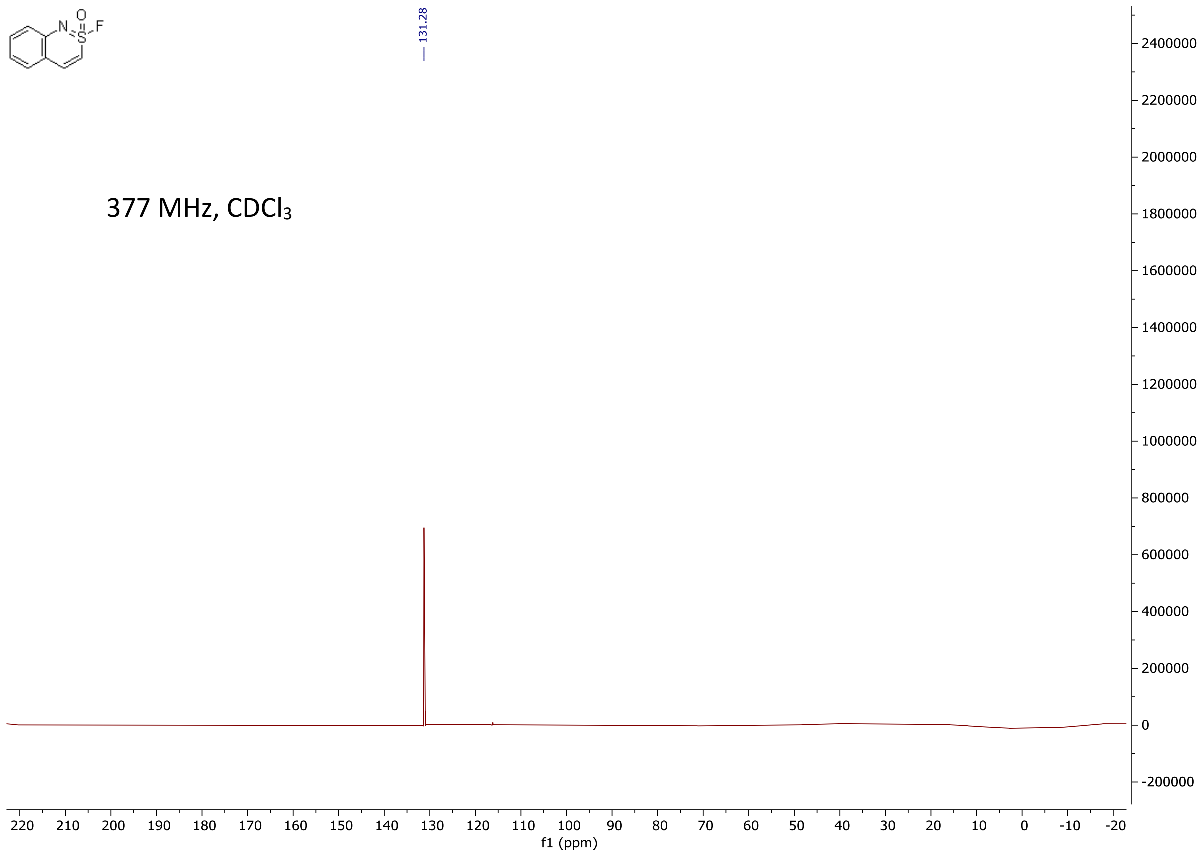
377 MHz, CDCl3
131.28
f1 (ppm)

2-phenoxy-2λ[4]-benzo[*c*][1,2]thiazine 2-oxide (11)

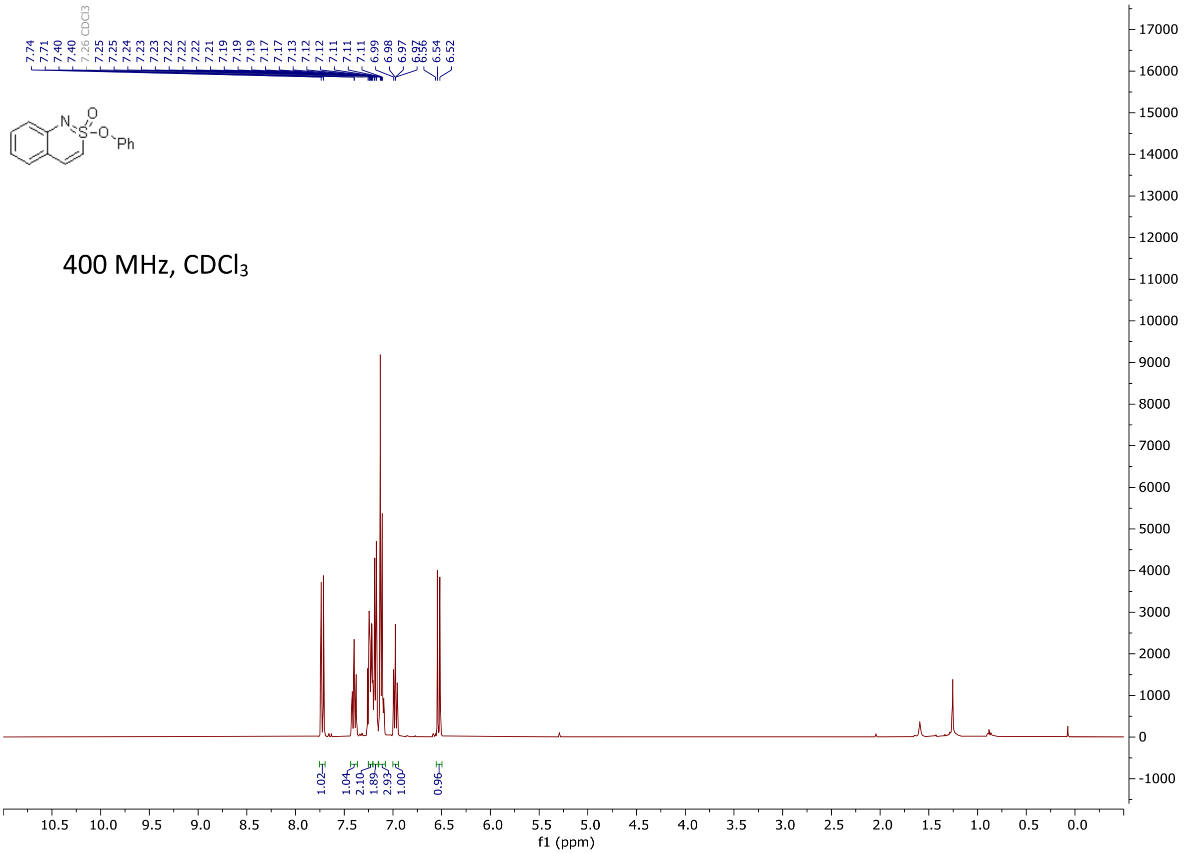

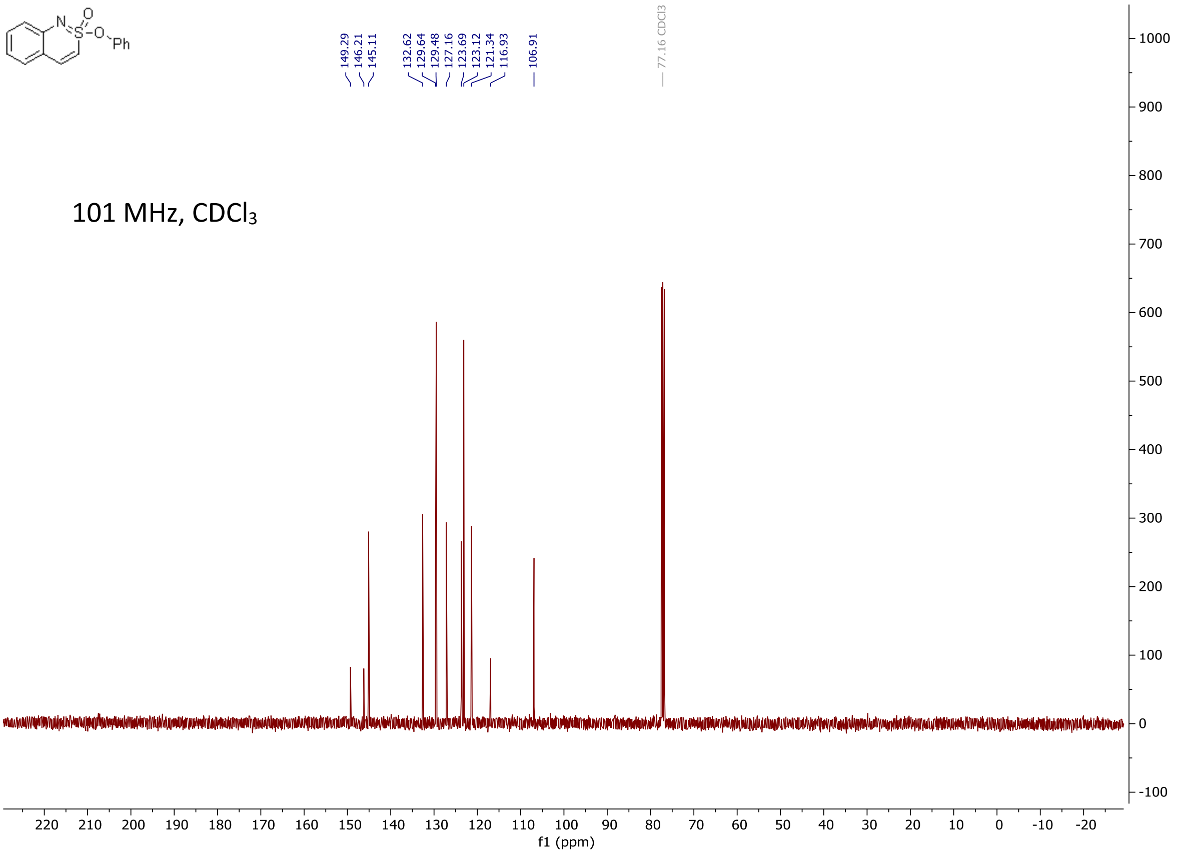

N-(((2-formylphenyl)imino)(methyl)(morpholino)-λ^6-sulfaneylidene)-4-nitrobenzenesulfonamide (13)

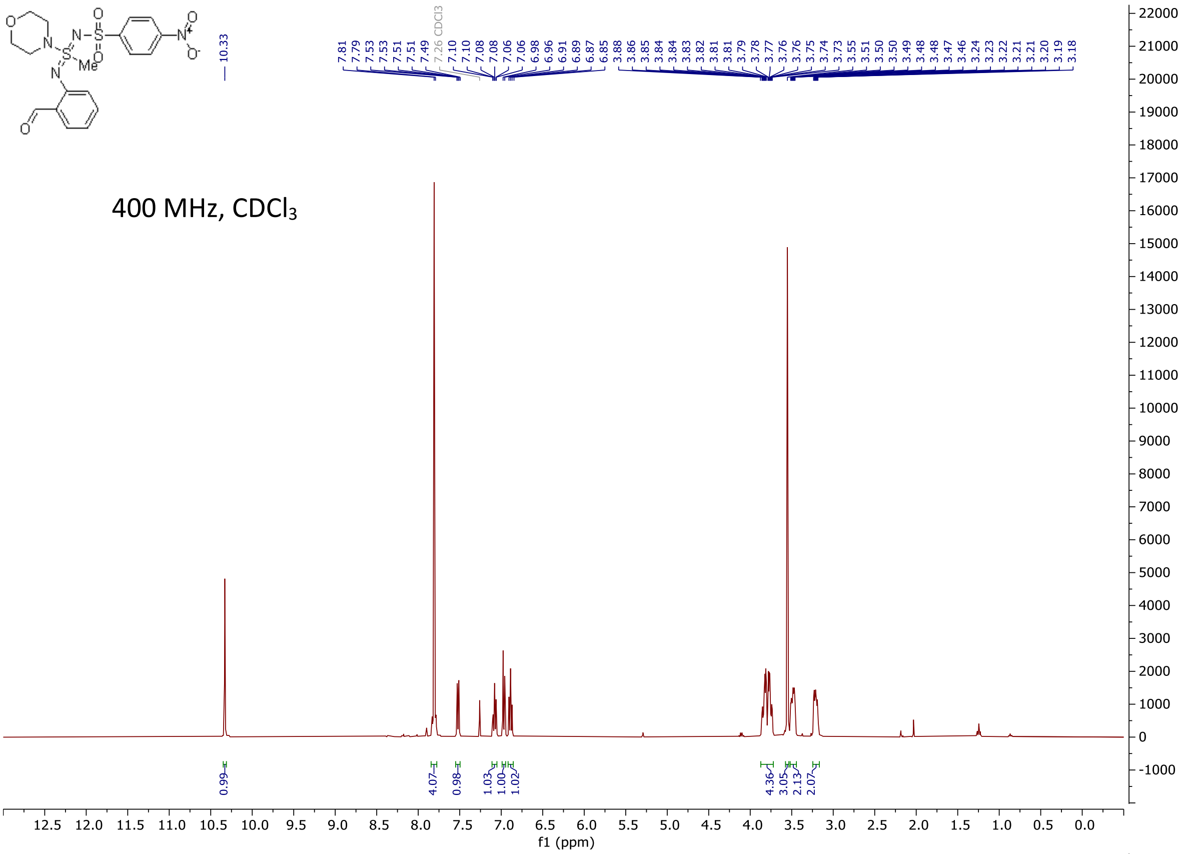

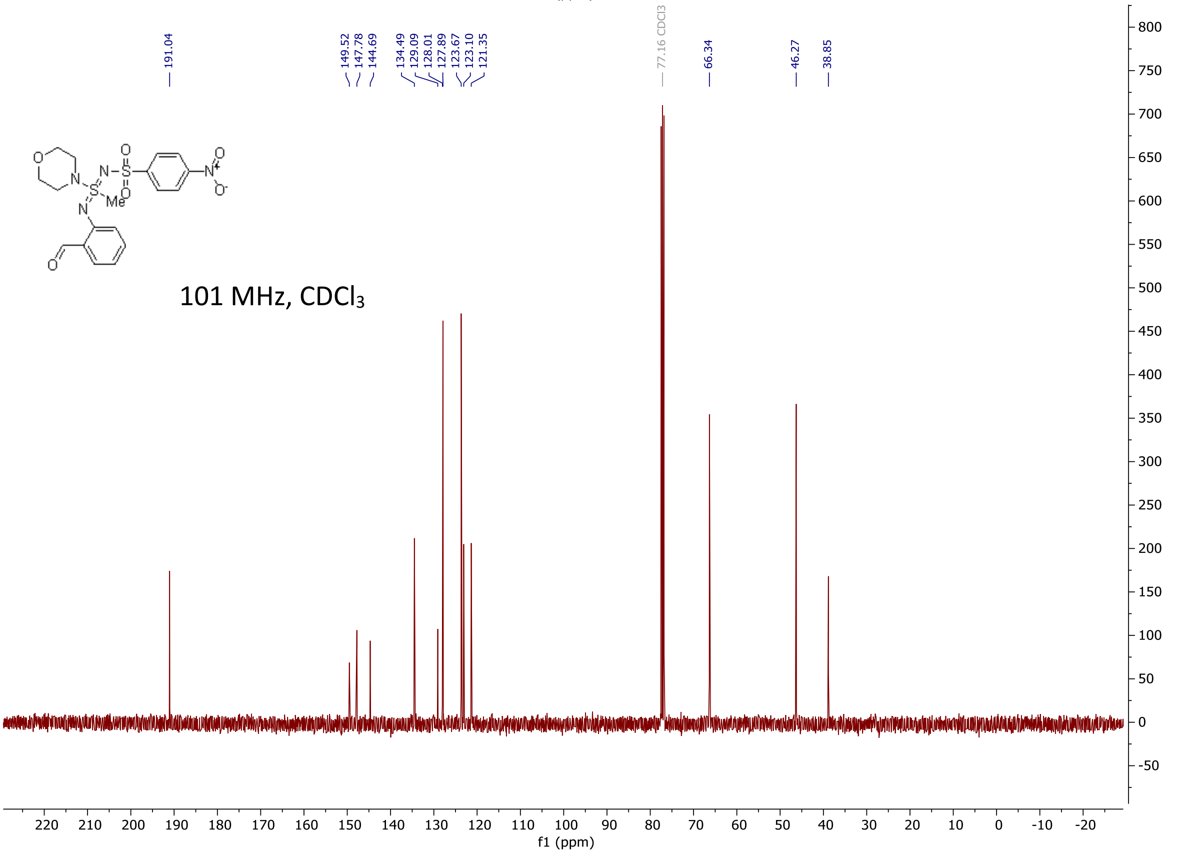

N-(2-morpholino-2λ[6]-benzo[*c*][1,2]thiazin-2-ylidene)-4-nitrobenzenesulfonamide (14)

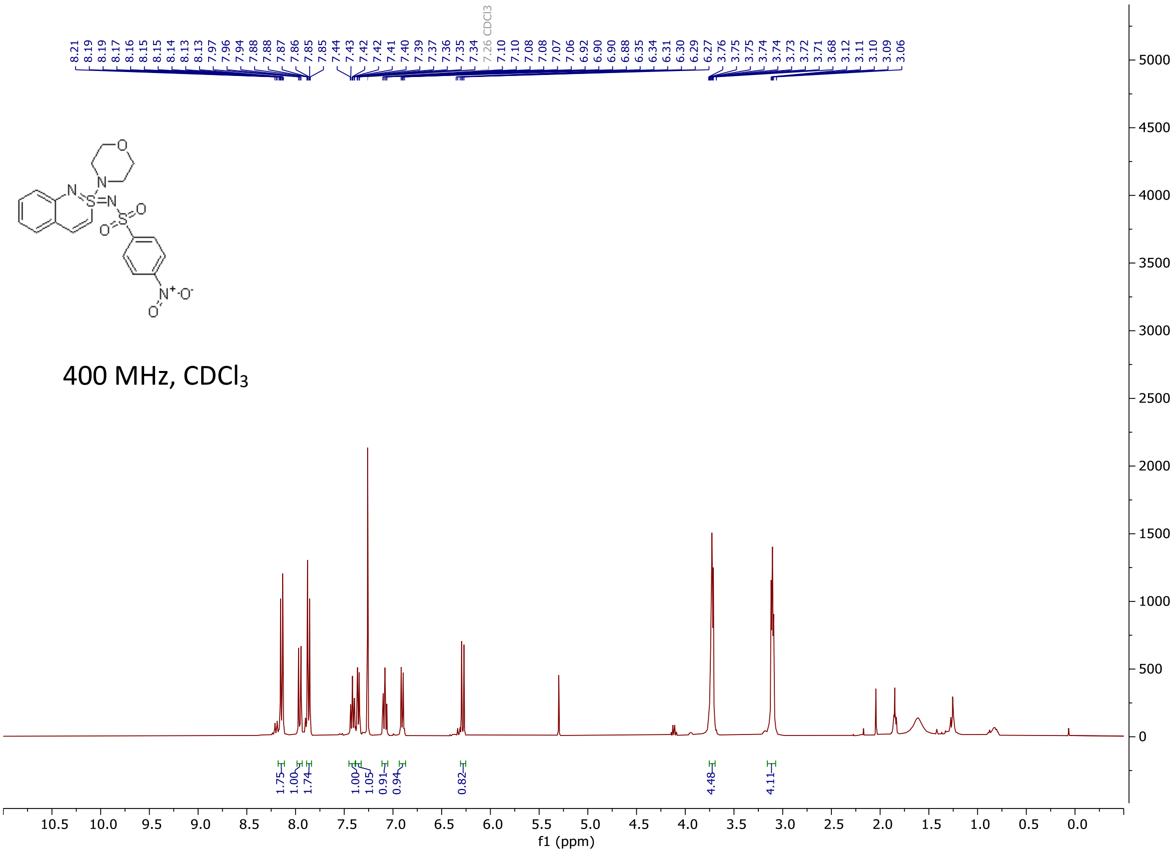

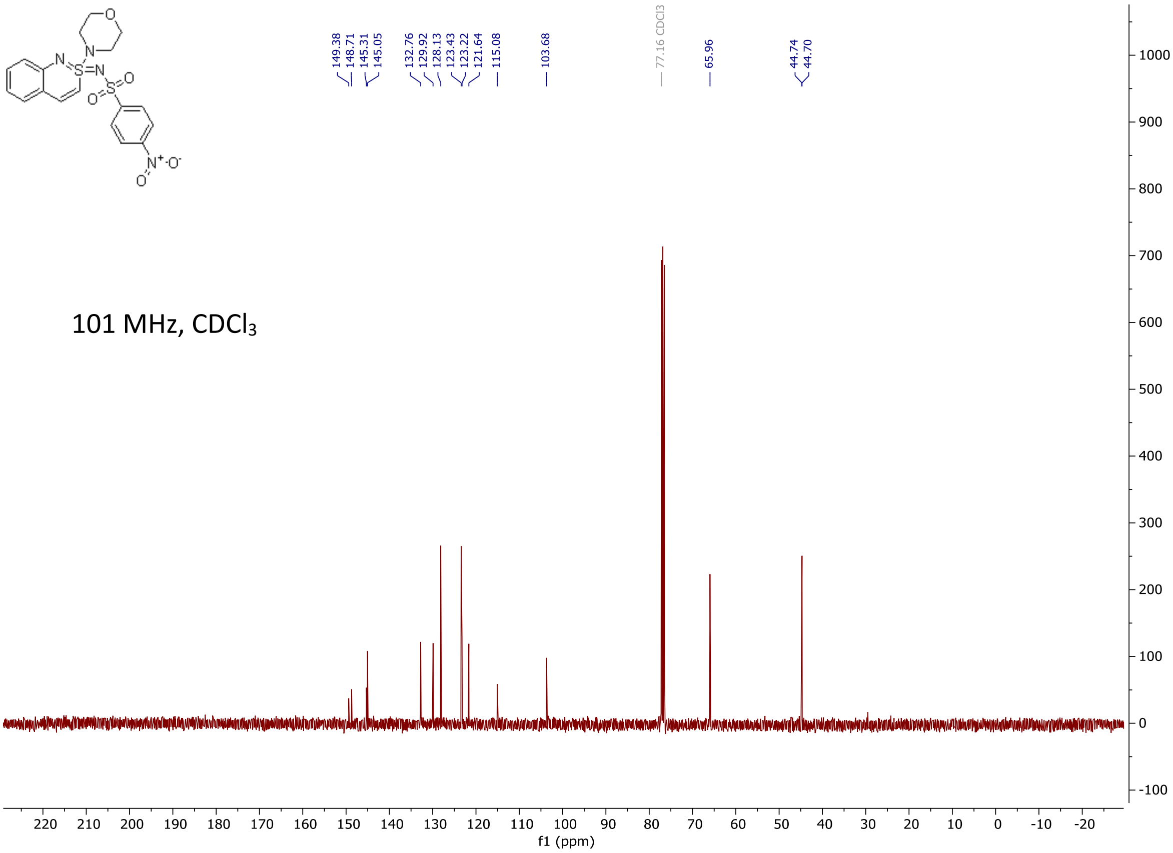

6-bromo-2-morpholinobenzo[*c*][1,2]thiazine 2-oxide (15)

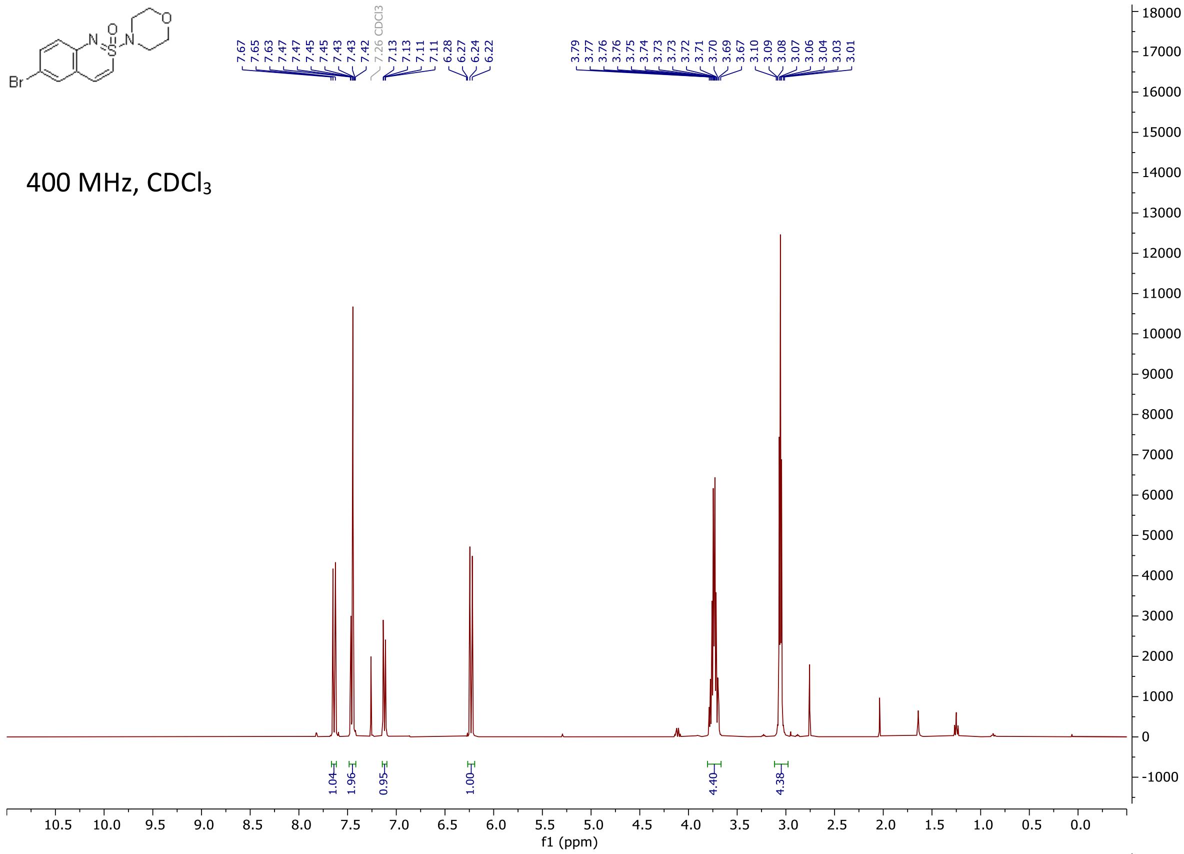

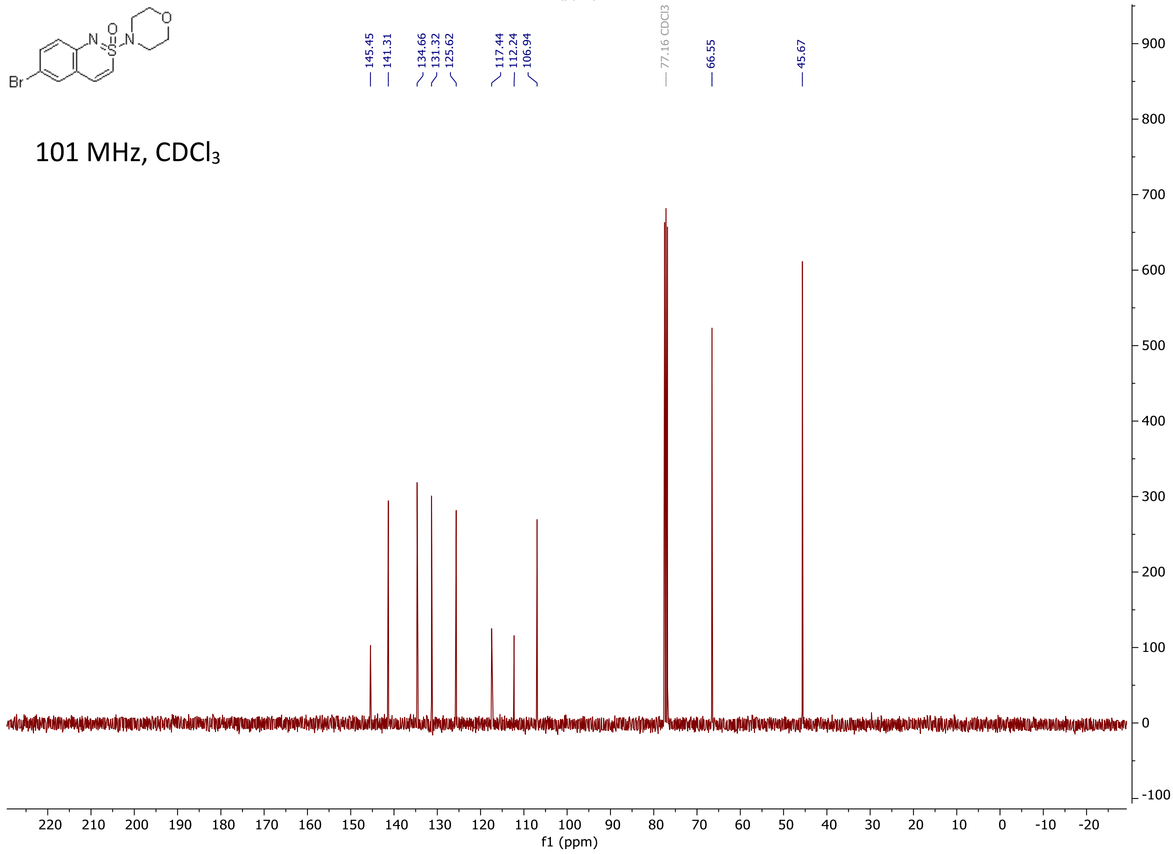

8-bromo-6-methoxy-2-morpholinobenzo[*c*][1,2]thiazine 2-oxide (16a)

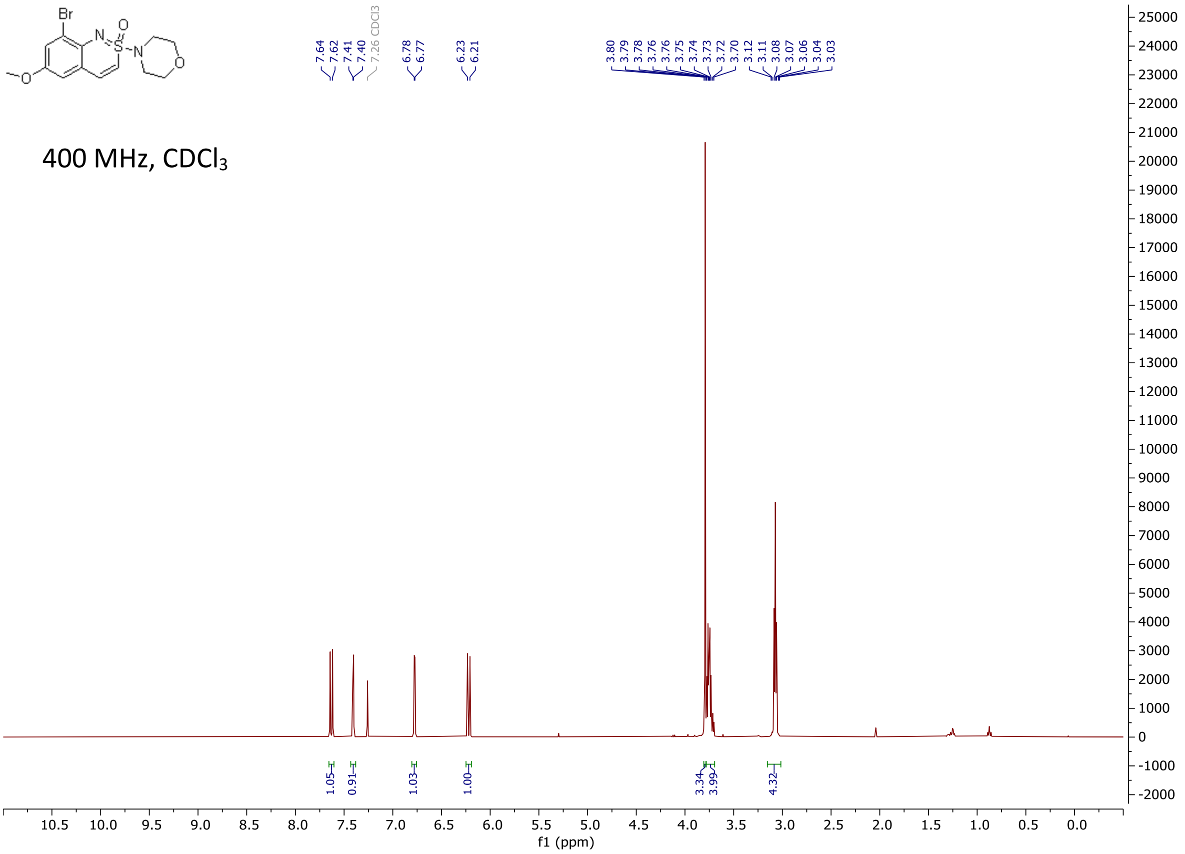

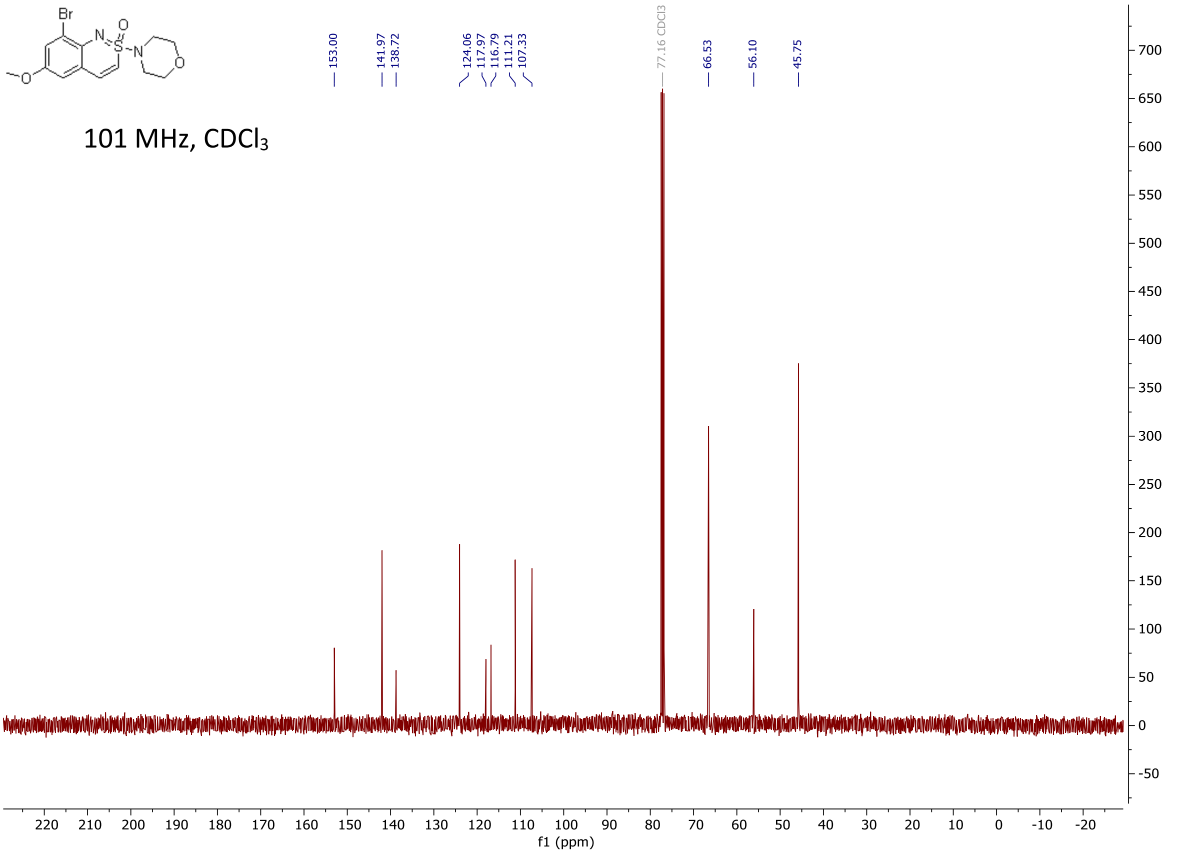

3-bromo-6-methoxy-2-morpholinobenzo[c][1,2]thiazine 2-oxide (16b)

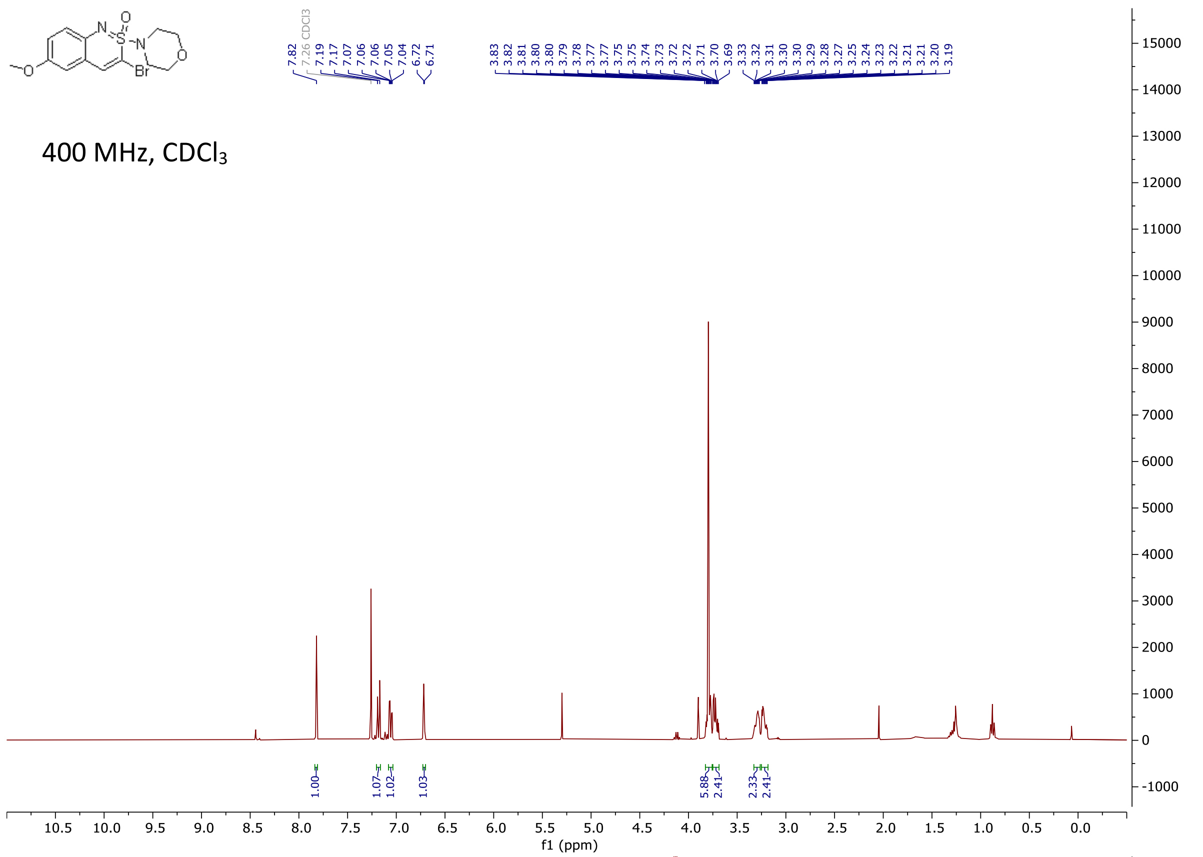

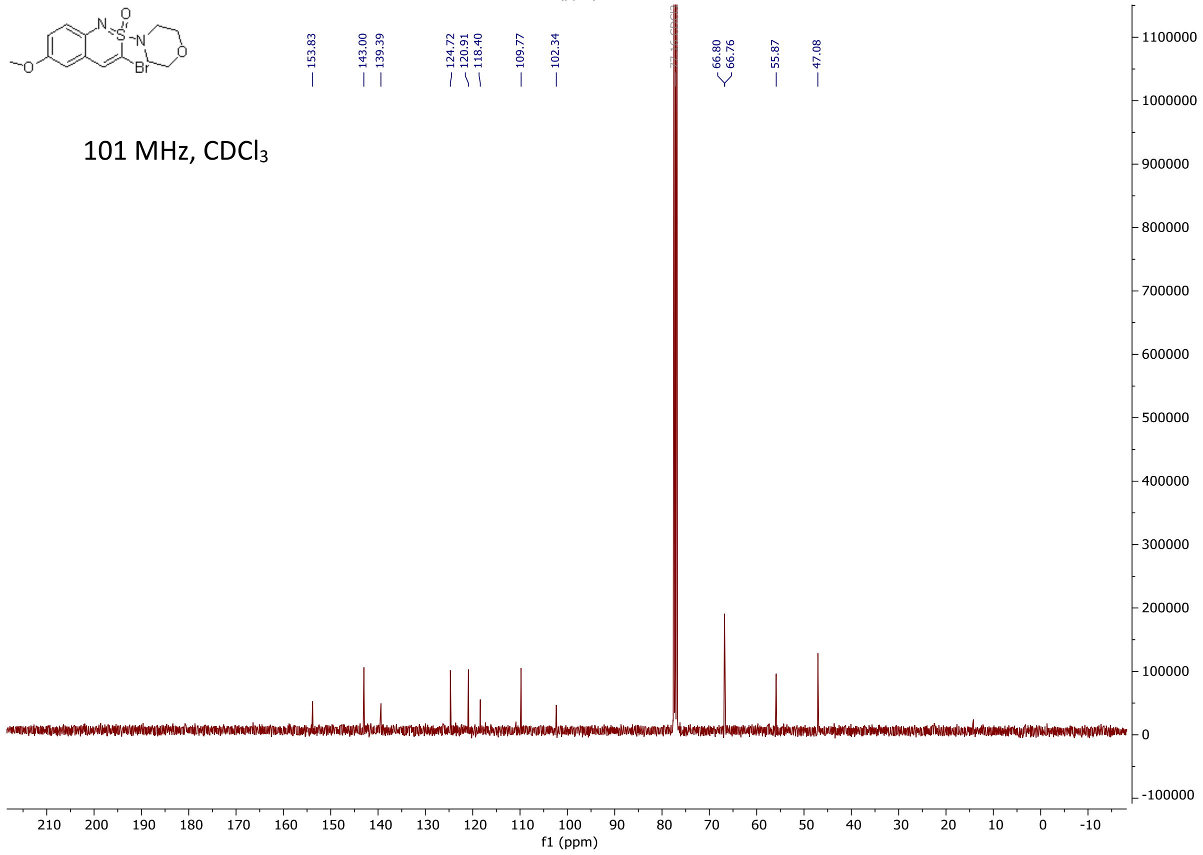

5,8-dibromo-6-methoxy-2-morpholinobenzo[*c*][1,2]thiazine 2-oxide (16c)

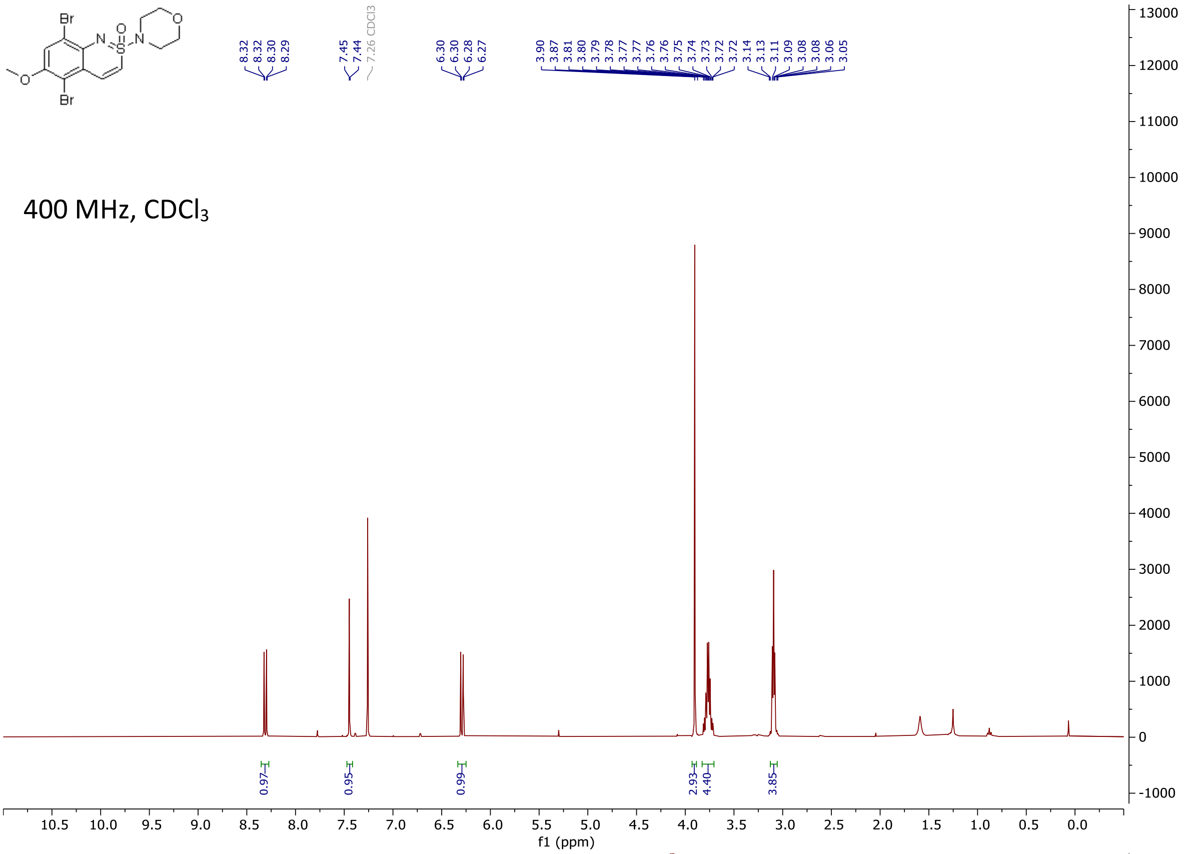

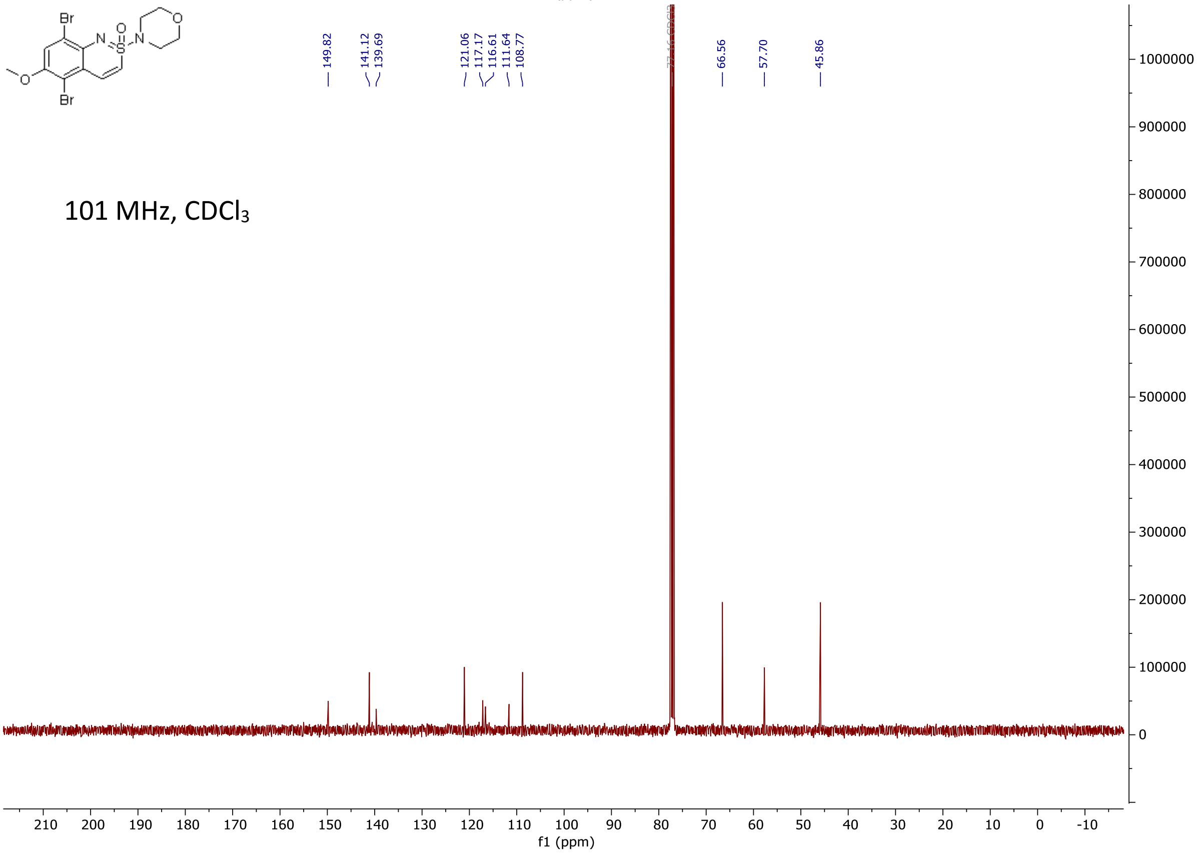

3,8-dibromo-6-methoxy-2-morpholinobenzo[*c*][1,2]thiazine 2-oxide (16d)

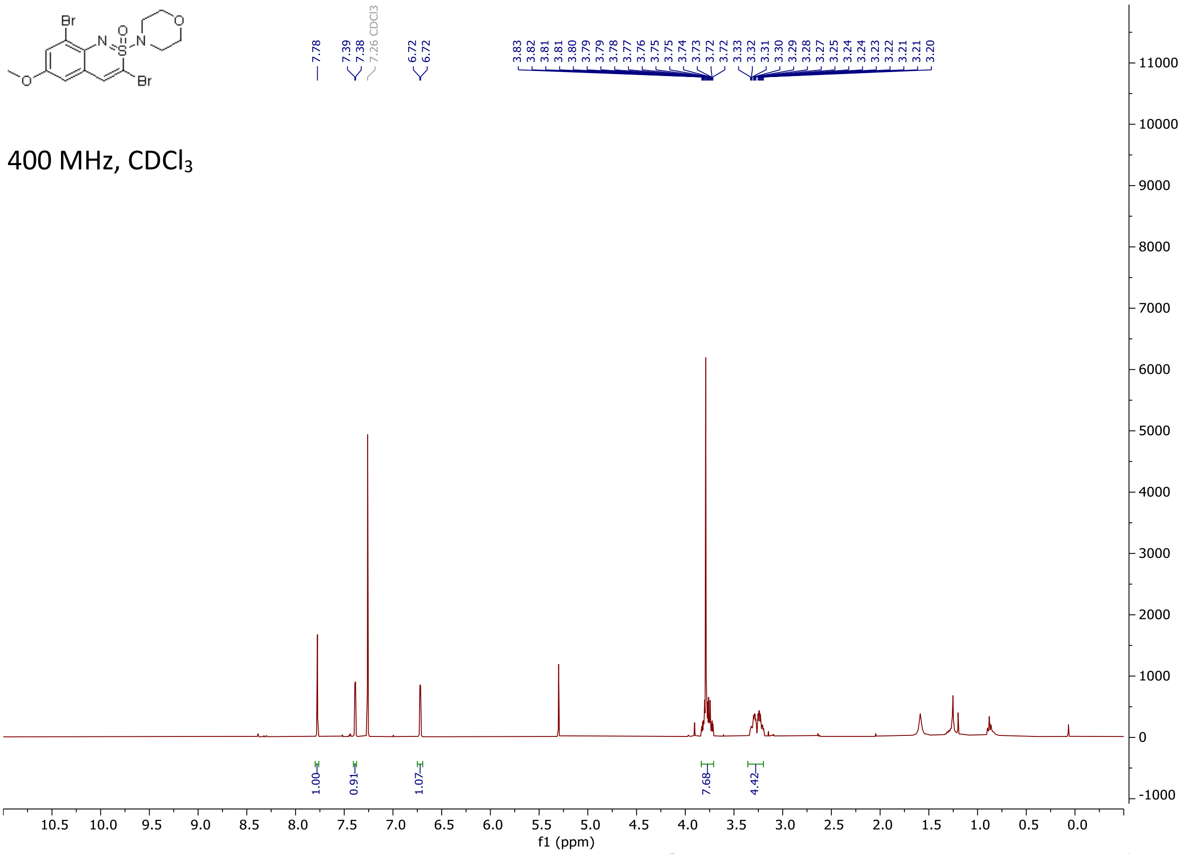

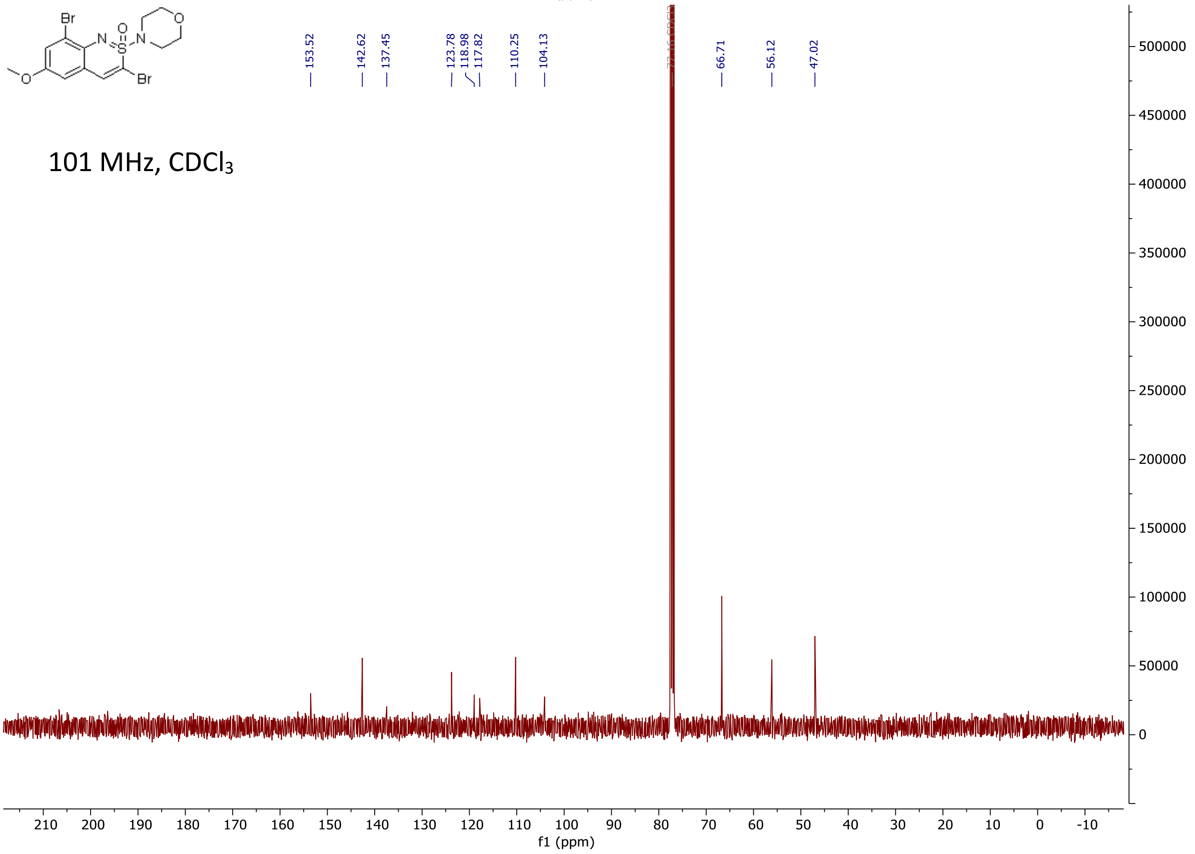

2-morpholino-3-(triisopropylsilyl)benzo[c][1,2]thiazine 2-oxide (17)

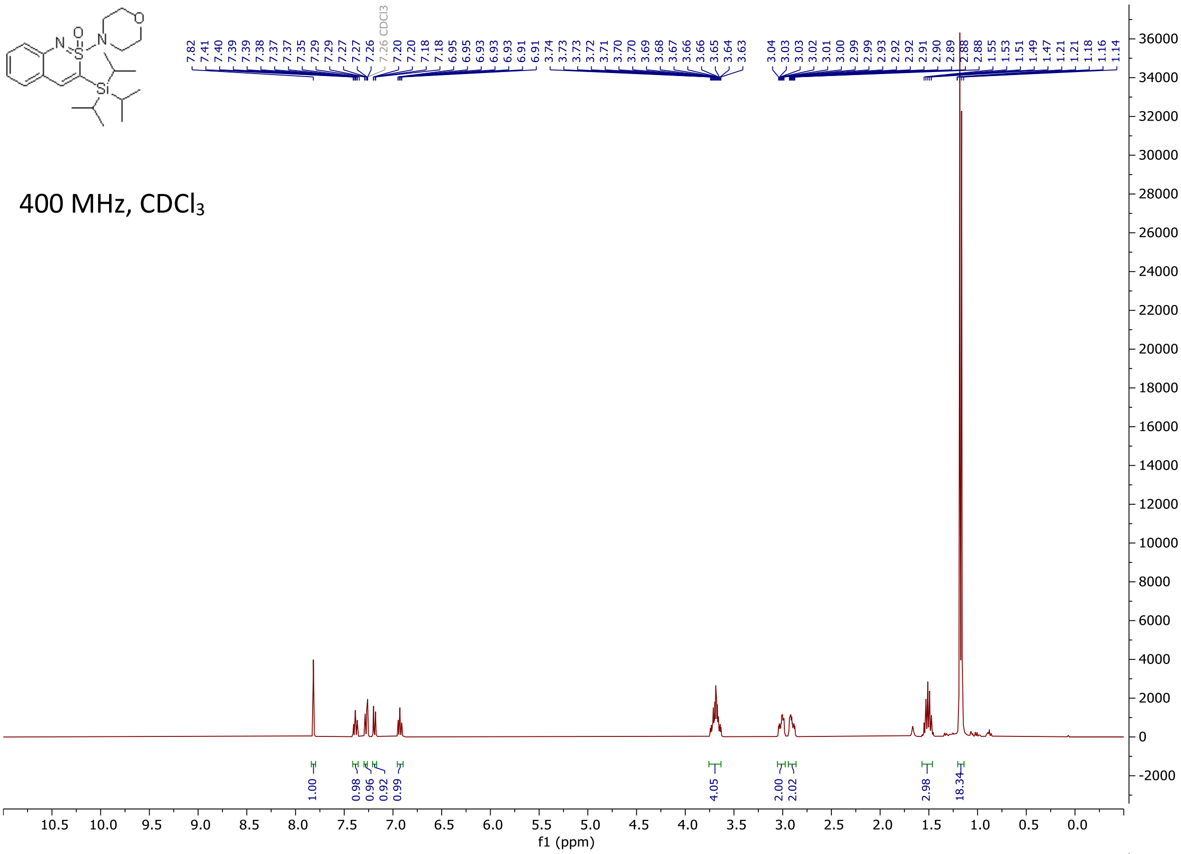

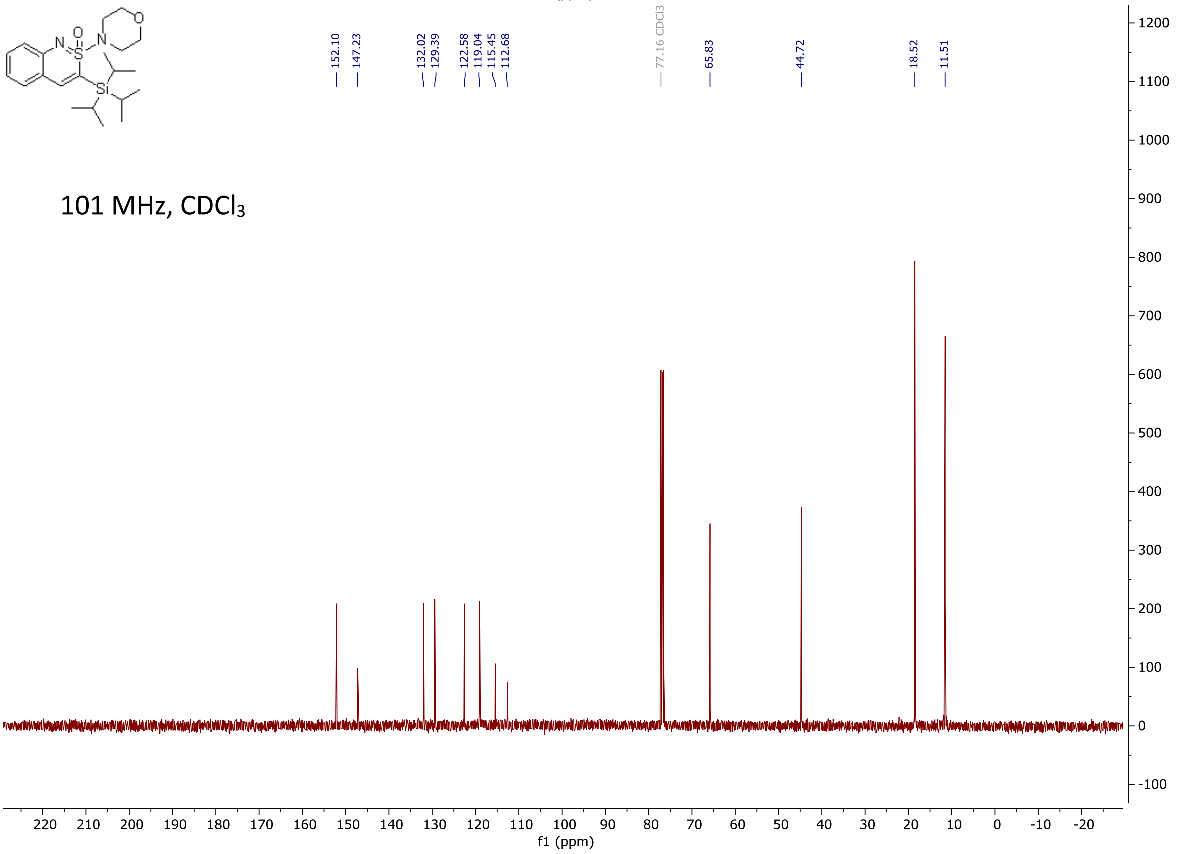

6. HPLC-Data

(*S*)-2-(((*S*)-1-phenylethyl)amino)-2λ[4]-benzo[*c*][1,2]thiazine 2-oxide (9c)

Column: Chiralpak IC; **Solvent:** *n*-hexane/IPA (85:15); **flowrate:** 0.8 mL/min

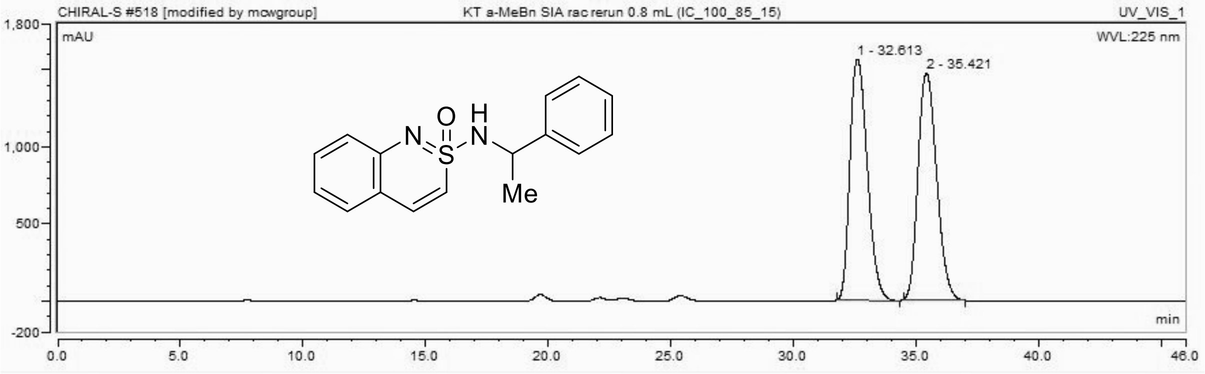

No.	Peakname	Ret.Time min	Area mAU*min	Amount	Type	Height mAU	Rel.Area %	Resolution
1	n.a.	32.613	#########	n.a.	BMB*	1562.857	49.80	2.06
2	n.a.	35.421	#########	n.a.	BMB*	1473.320	50.20	n.a.
Total:			#########	0.0000		3036.177	100.00	

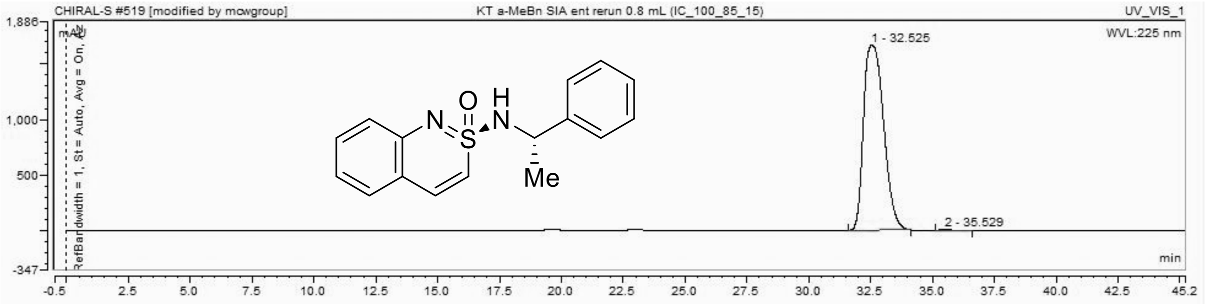

No.	Peakname	Ret.Time min	Area mAU*min	Amount	Type	Height mAU	Rel.Area %	Resolution
1	n.a.	32.525	#########	n.a.	BMB*	1671.072	99.81	2.37
2	n.a.	35.529	2.9874	n.a.	BMB*	5.022	0.19	n.a.
Total:			#########	0.0000		1676.094	100.00	

(*R*)-2-(((*S*)-1-phenylethyl)amino)-2λ[4]-benzo[*c*][1,2]thiazine 2-oxide (9c')

Column: Chiralpak IC; **Solvent:** *n*-hexane/IPA (85:15); **flowrate:** 0.8 mL/min

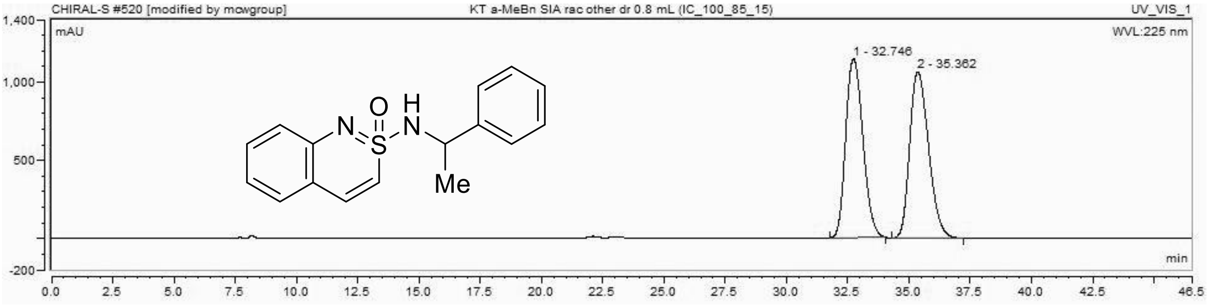

No.	Peakname	Ret.Time min	Area mAU*min	Amount	Type	Height mAU	Rel.Area %	Resolution
1	n.a.	32.746	931.1006	n.a.	BMB*	1142.745	49.44	1.92
2	n.a.	35.362	952.0343	n.a.	BMB*	1060.039	50.56	n.a.
Total:			#########	0.0000		2202.784	100.00	

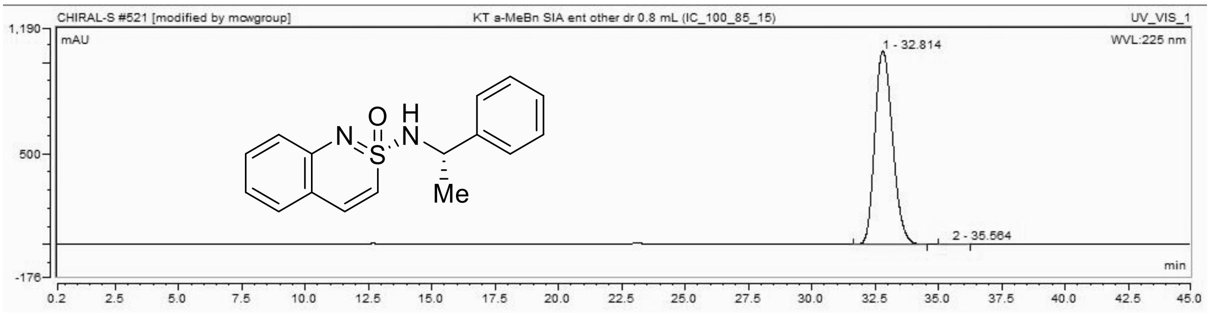

No.	Peakname	Ret.Time min	Area mAU*min	Amount	Type	Height mAU	Rel.Area %	Resolution
1	n.a.	32.814	864.9149	n.a.	BMB*	1062.088	99.78	2.25
2	n.a.	35.564	1.9285	n.a.	BMB*	2.846	0.22	n.a.
Total:			866.8434	0.0000		1064.934	100.00	

(R)-2-amino-2 λ⁴-benzo[*c*][1,2]thiazine 2-oxide ((R)-9f)

Column: Chiralpak IC; **Solvent:** *n*-hexane/IPA (70:30); **flowrate:** 0.7 mL/min

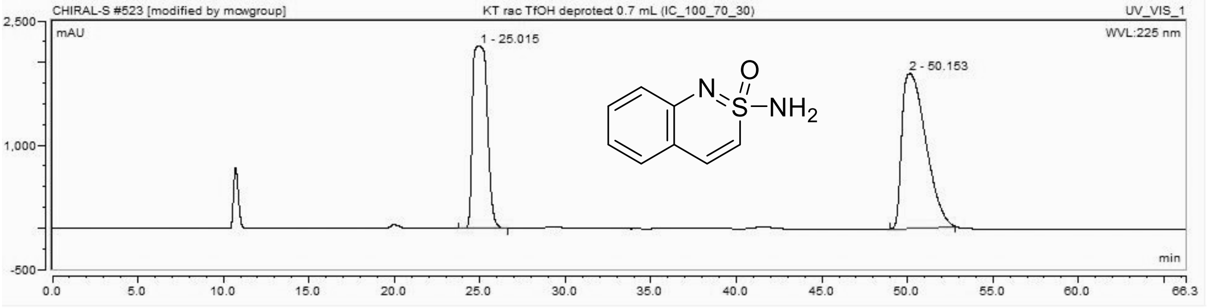

No.	Peakname	Ret.Time min	Area mAU*min	Amount	Type	Height mAU	Rel.Area %	Resolution
1	n.a.	25.015	#########	n.a.	BMB*	2201.862	42.06	11.84
2	n.a.	50.153	#########	n.a.	BMB*	1868.286	57.94	n.a.
Total:			#########	0.0000		4070.148	100.00	

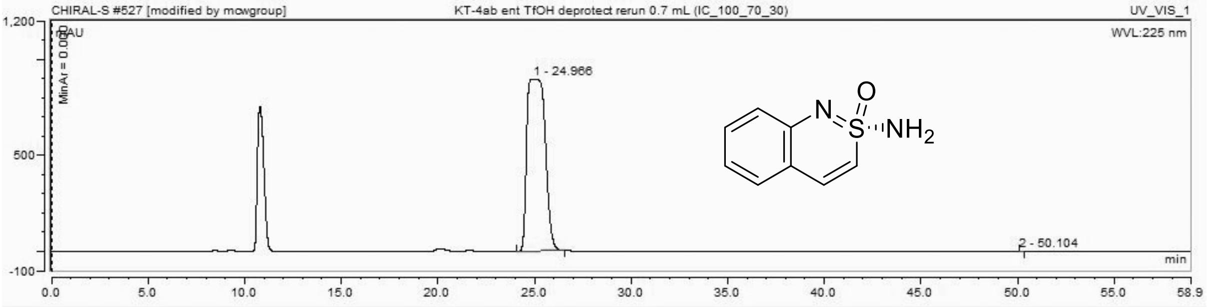

No.	Peakname	Ret.Time min	Area mAU*min	Amount	Type	Height mAU	Rel.Area %	Resolution
1	n.a.	24.966	976.9598	n.a.	BMB*	894.033	100.00	n.a.
2	n.a.	50.104	0.0193	n.a.	BMB*	0.037	0.00	n.a.
Total:			976.9791	0.0000		894.070	100.00	
